# Amphioxus functional genomics and the origins of vertebrate gene regulation

**DOI:** 10.1038/s41586-018-0734-6

**Published:** 2018-11-21

**Authors:** Ferdinand Marlétaz, Panos N. Firbas, Ignacio Maeso, Juan J. Tena, Ozren Bogdanovic, Malcolm Perry, Christopher D. R. Wyatt, Elisa de la Calle-Mustienes, Stephanie Bertrand, Demian Burguera, Rafael D. Acemel, Simon J. van Heeringen, Silvia Naranjo, Carlos Herrera-Ubeda, Ksenia Skvortsova, Sandra Jimenez-Gancedo, Daniel Aldea, Yamile Marquez, Lorena Buono, Iryna Kozmikova, Jon Permanyer, Alexandra Louis, Beatriz Albuixech-Crespo, Yann Le Petillon, Anthony Leon, Lucie Subirana, Piotr J. Balwierz, Paul Edward Duckett, Ensieh Farahani, Jean-Marc Aury, Sophie Mangenot, Patrick Wincker, Ricard Albalat, Èlia Benito-Gutiérrez, Cristian Cañestro, Filipe Castro, Salvatore D’Aniello, David E. K. Ferrier, Shengfeng Huang, Vincent Laudet, Gabriel A. B. Marais, Pierre Pontarotti, Michael Schubert, Hervé Seitz, Ildiko Somorjai, Tokiharu Takahashi, Olivier Mirabeau, Anlong Xu, Jr-Kai Yu, Piero Carninci, Juan Ramon Martinez-Morales, Hugues Roest Crollius, Zbynek Kozmik, Matthew T. Weirauch, Jordi Garcia-Fernàndez, Ryan Lister, Boris Lenhard, Peter W. H. Holland, Hector Escriva, Jose Luis Gómez-Skarmeta, Manuel Irimia

**Affiliations:** 1 Department of Zoology, University of Oxford, Oxford, UK; 2 Molecular Genetics Unit, Okinawa Institute of Science and Technology Graduate University, Onna-son, Japan; 3 Centro Andaluz de Biología del Desarrollo (CABD), CSIC-Universidad Pablo de Olavide-Junta de Andalucía, Seville, Spain; 4 Genomics and Epigenetics Division, Garvan Institute of Medical Research, Sydney, New South Wales Australia; 5 St Vincent’s Clinical School, Faculty of Medicine, University of New South Wales, Sydney, New South Wales Australia; 6 Australian Research Council Centre of Excellence in Plant Energy Biology, School of Molecular Sciences, The University of Western Australia, Crawley, Western Australia Australia; 7 Institute of Clinical Sciences, Faculty of Medicine, Imperial College London, London, UK; 8 Computational Regulatory Genomics, MRC London Institute of Medical Sciences, London, UK; 9 Centre for Genomic Regulation (CRG), Barcelona Institute of Science and Technology (BIST), Barcelona, Spain; 10 Universitat Pompeu Fabra (UPF), Barcelona, Spain; 11 Biologie Intégrative des Organismes Marins, BIOM, Observatoire Océanologique, CNRS and Sorbonne Université, Banyuls sur Mer, France; 12 Department of Genetics, Microbiology and Statistics, Faculty of Biology, and Institut de Biomedicina (IBUB), University of Barcelona, Barcelona, Spain; 13 Department of Molecular Developmental Biology, Faculty of Science, Radboud Institute for Molecular Life Sciences, Radboud University, Nijmegen, The Netherlands; 14 Institute of Molecular Genetics of the Czech Academy of Sciences, Prague, Czech Republic; 15 Institut de Biologie de l’ENS, IBENS, Ecole Normale Supérieure, Paris, France; 16 Inserm, U1024, Paris, France; 17 CNRS, UMR 8197, Paris, France; 18 Genoscope, Institut de biologie François-Jacob, Commissariat à l’Energie Atomique (CEA), Université Paris-Saclay, Evry, France; 19 Génomique Métabolique, Genoscope, Institut de biologie François Jacob, Commissariat à l’Energie Atomique (CEA), CNRS, Université Evry, Université Paris-Saclay, Evry, France; 20 Department of Genetics, Microbiology and Statistics, Faculty of Biology and Institut de Recerca de la Biodiversitat (IRBio), University of Barcelona, Barcelona, Spain; 21 Department of Zoology, University of Cambridge, Cambridge, UK; 22 Interdisciplinary Centre of Marine and Environmental Research (CIIMAR/CIMAR) and Faculty of Sciences (FCUP), Department of Biology, University of Porto, Porto, Portugal; 23 Biology and Evolution of Marine Organisms, Stazione Zoologica Anton Dohrn Napoli, Naples, Italy; 24 The Scottish Oceans Institute, Gatty Marine Laboratory, University of St Andrews, St Andrews, UK; 25 State Key Laboratory of Biocontrol, School of Life Sciences, Sun Yat-sen University, Guangzhou, China; 26 Laboratoire de Biométrie et Biologie Evolutive (UMR 5558), CNRS and Université Lyon 1, Villeurbanne, France; 27 IRD, APHM, Microbe, Evolution, PHylogénie, Infection, IHU Méditerranée Infection and CNRS, Aix Marseille University, Marseille, France; 28 Sorbonne Université, CNRS, Laboratoire de Biologie du Développement de Villefranche-sur-Mer, Institut de la Mer de Villefranche-sur-Mer, Villefranche-sur-Mer, France; 29 UMR 9002 CNRS, Institut de Génétique Humaine, Université de Montpellier, Montpellier, France; 30 Biomedical Sciences Research Complex, School of Biology, University of St Andrews, St Andrews, UK; 31 School of Medical Sciences, Faculty of Biology, Medicine and Health, University of Manchester, Manchester, UK; 32 INSERM U830, Équipe Labellisée LNCC, SIREDO Oncology Centre, Institut Curie, PSL Research University, Paris, France; 33 School of Life Sciences, Beijing University of Chinese Medicine, Beijing, China; 34 Institute of Cellular and Organismic Biology, Academia Sinica, Taipei, Taiwan; 35 RIKEN Center for Life Science Technologies (Division of Genomic Technologies) (CLST DGT), Yokohama, Japan; 36 Laboratory for Transcriptome Technology, RIKEN Center for Integrative Medical Sciences, Yokohama, Japan; 37 Center for Autoimmune Genomics and Etiology, Divisions of Biomedical Informatics and Developmental Biology, Cincinnati Children’s Hospital Medical Center, Cincinnati, OH USA; 38 Department of Pediatrics, University of Cincinnati College of Medicine, Cincinnati, OH USA; 39 Harry Perkins Institute of Medical Research, Nedlands, Western Australia Australia; 40 Sars International Centre for Marine Molecular Biology, University of Bergen, Bergen, Norway

**Keywords:** Molecular evolution, Functional genomics, Epigenomics

## Abstract

Vertebrates have greatly elaborated the basic chordate body plan and evolved highly distinctive genomes that have been sculpted by two whole-genome duplications. Here we sequence the genome of the Mediterranean amphioxus (*Branchiostoma lanceolatum*) and characterize DNA methylation, chromatin accessibility, histone modifications and transcriptomes across multiple developmental stages and adult tissues to investigate the evolution of the regulation of the chordate genome. Comparisons with vertebrates identify an intermediate stage in the evolution of differentially methylated enhancers, and a high conservation of gene expression and its *cis*-regulatory logic between amphioxus and vertebrates that occurs maximally at an earlier mid-embryonic phylotypic period. We analyse regulatory evolution after whole-genome duplications, and find that—in vertebrates—over 80% of broadly expressed gene families with multiple paralogues derived from whole-genome duplications have members that restricted their ancestral expression, and underwent specialization rather than subfunctionalization. Counter-intuitively, paralogues that restricted their expression increased the complexity of their regulatory landscapes. These data pave the way for a better understanding of the regulatory principles that underlie key vertebrate innovations.

## Abstract (web summary)

Genomic, epigenomic and transcriptomic data derived from the Mediterranean amphioxus (*Branchiostoma lanceolatum*) provide insights into the evolution of the genomic regulatory landscape of chordates.

## Main

All vertebrates share multiple morphological and genomic novelties^1^. The most prominent genomic difference between vertebrates and non-vertebrate chordates is the reshaping of the gene complement that followed the two rounds of whole genome duplication (WGD)—the 2R hypothesis—that occurred at the base of the vertebrate lineage^2,3^. These large-scale mutational events are hypothesized to have facilitated the evolution of vertebrate morphological innovations, at least in part through the preferential retention of ‘developmental’ gene families and transcription factors after duplication^3,4^. However, duplicate genes and their associated regulatory elements were initially identical and could not drive innovation without regulatory and/or protein-coding changes.

To date, the effect of vertebrate WGDs on gene regulation have remained poorly understood—both in terms of the fates of duplicate genes and the acquisition of the unique genomic traits that are characteristic of vertebrates. These traits include numerous features that are often associated with gene regulation, such as unusually large intergenic and intronic regions^5,6^, high global 5-methylcytosine (5mC) content and 5mC-dependent regulation of embryonic transcriptional enhancers^7^. To investigate these traits, appropriate species must be used for comparisons. Previous studies have largely focused on phylogenetic distances that are either too short (such as human versus mouse) or too long (such as human versus fly or nematode), resulting in limited insights. In the first case, comparisons among closely related species (for example, between mammals^8–11^)—for which the orthology of non-coding regions can be readily determined from genomic alignments—have allowed fine-grained analyses of the evolution of transcription-factor binding. In the second case, three-way comparisons of human, fly and nematode by the modENCODE consortium revealed no detectable conservation at the *cis*-regulatory level^12^ and very little conservation of gene expression^13^. Moreover, the genomes of flies and nematodes are highly derived^14–16^. Thus, we lack comprehensive functional genomic data from a slow-evolving, closely related outgroup that would enable an in-depth investigation of the origins of the vertebrate regulatory genome and of the effect of WGDs on gene regulation.

Unlike flies, nematodes and most non-vertebrates, amphioxus belongs to the chordate phylum. Therefore, although amphioxus lacks the specializations and innovations of vertebrates, it shares with them a basic body plan and has multiple organs and structures homologous to those of vertebrates^1^. For these reasons, amphioxus has widely been used as a reference outgroup to infer ancestral versus novel features during vertebrate evolution. Here, we undertook a comprehensive study of the transcriptome and regulatory genome of amphioxus to investigate how the unique functional genome architecture of vertebrates evolved.

## Functional genome annotation of amphioxus

**Fig. 1:**
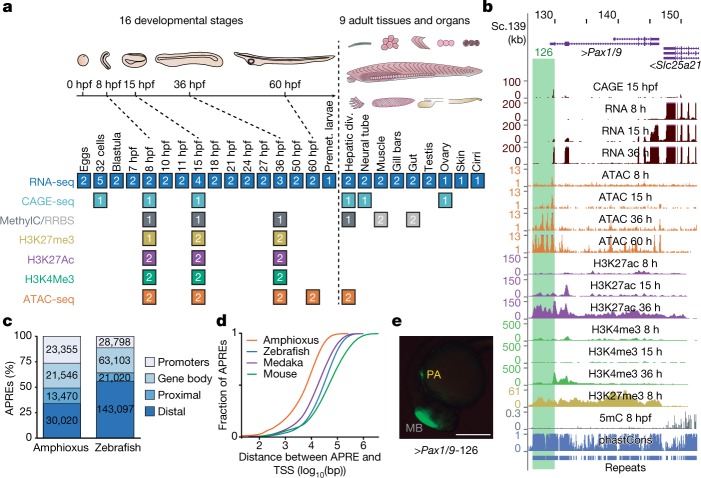
Functional genome annotation of amphioxus. **a**, Summary of the 94 amphioxus samples generated in this study, comprising eight functional-genomic datasets. The number of biological replicates is indicated for each sample type. div., diverticulum; MethylC/RRBS, methylC sequencing and reduced representation bisulfite sequencing; Premet., premetamorphic. **b**, Genome browser excerpt showing a selection of available tracks, including gene annotation, sequence conservation (using phastCons), repeats and several epigenomic and transcriptomic datasets. Green rectangle highlights the APRE tested in **e**. **c**, Numbers and proportions of amphioxus and zebrafish APREs according to their genomic location. Promoters, within 1-kbp upstream and 0.5-kbp downstream of an annotated TSS; gene body, within an orthology-supported gene; proximal, within 5-kbp upstream of (but not overlapping with) a TSS; distal, not in the aforementioned categories. **d**, Cumulative distributions of the distance between each APRE and the closest annotated TSS in each species. **e**, Lateral view of a representative transgenic zebrafish 26-hpf embryo showing GFP expression driven by an amphioxus APRE associated with *Pax1/9* (‘*Pax1/9*-126’, highlighted in **b**) in pharyngeal arches (PA; *n* = 4/4). Positive-control enhancer was expressed in the midbrain (MB). Scale bar, 250 μm.

**Extended Data Fig. 1:**
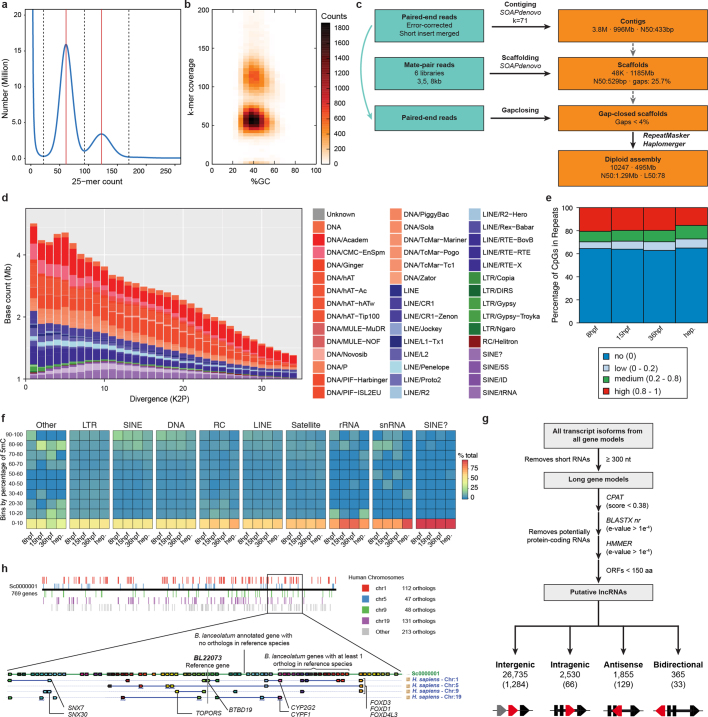
Summary of genomic assembly and repeat annotation. **a**, Spectrum of 25-mers in Illumina sequencing data that shows the bimodal distribution that is characteristic of highly polymorphic species. **b**, Heat map showing *k*-mer decomposition (*y* axis) across GC content (*x* axis). Both peaks show comparable GC content, which is consistent with them representing haploid versus diploid *k*-mers. **c**, Flow chart of the steps followed to obtain the *B. lanceolatum* assembly. **d**, Repeat landscape and its evolutionary history, shown by the proportion of repetitive elements with a given divergence (K2P) to their consensus in the repeat library (repeatScout). **e**, Percentage of methylated CpG dinucleotides within repetitive elements, at three developmental stages and in the adult hepatic diverticulum. **f**, Distribution of average levels of 5mC of different repeat families. Colour key indicates the percentage of repeats in each family with corresponding levels of average methylation. **g**, Computational pipeline to identify long non-coding RNAs (lncRNAs). Categories: antisense, lncRNA overlaps with a protein-coding gene in the reverse strand; intragenic, lncRNA overlaps with a protein-coding gene in the same strand; bidirectional, within 1 kbp of a TSS of a protein-coding gene in the antisense strand, probably a product of a bidirectional promoter; intergenic, lncRNA does not overlap with any protein-coding gene. The total number in each category is indicated, with the number of those that are multi-exonic in parentheses. **h**, Quadruple conserved synteny between amphioxus and human. Top, amphioxus scaffold Sc0000001 aligned against the four human chromosomes with which it shares the highest number of orthologues (chr1, chr5, chr9 and chr19). In this scaffold, 277 out of 551 genes have clear orthologues in human, and 203 of these have orthologues on at least one of the four mentioned chromosomes. The black horizontal line represents the amphioxus scaffold, and each vertical coloured box an orthologous gene on the corresponding human chromosome. Bottom, modified view from Genomicus that is centred on the *BL22073* gene and spans Sc0000001: 7,736,434–8,850,041. On the top line, each amphioxus gene with at least one orthologue in the nine reference species is represented with an oriented coloured box. Human genes located in the four ohnologous chromosomes are aligned underneath, in boxes of colours that correspond to those of their amphioxus pro-orthologues. The Genomicus server dedicated to amphioxus can be accessed at http://genomicus.biologie.ens.fr/genomicus-amphioxus.

**Extended Data Fig. 2:**
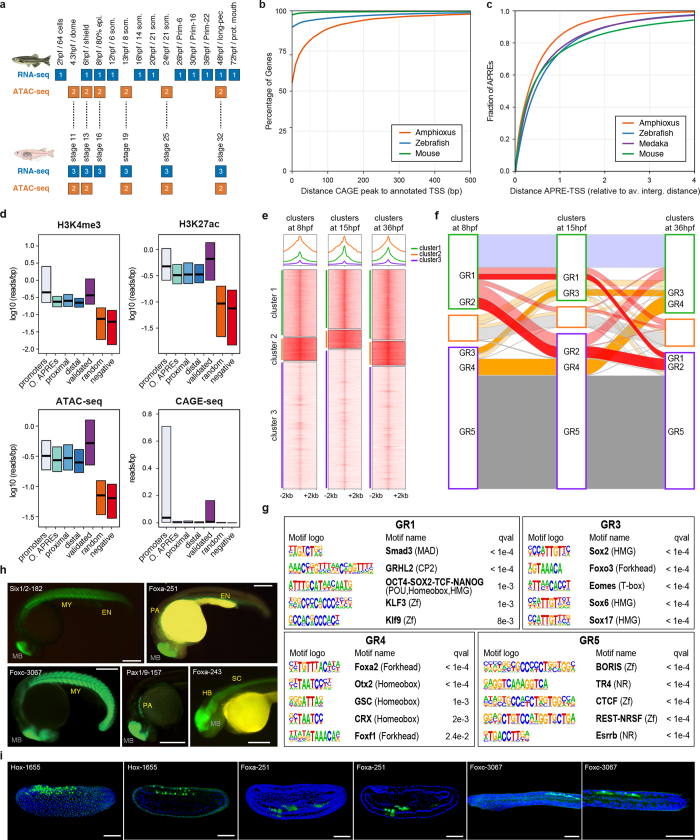
Dynamics of chromatin marks on APREs and reporter assays. **a**, Summary of the zebrafish and medaka RNA-seq and ATAC-seq datasets generated for this study. Dashed lines indicate equivalent developmental stages in the two species, based on aprevious study^70^. The number of biological replicates is indicated for each experiment. Zebrafish 24-hpf ATAC-seq data are from a previous study^67^. **b**, Cumulative distribution of the distance between CAGE-seq peaks and the closest annotated TSSs for genes with expression cRPKM > 5 in any of the samples covered by CAGE-seq (see Fig. 1a). Only CAGE-seq peaks within 1 kbp of an annotated TSS were tested (amphioxus: 10,435 peaks; zebrafish, 23,326 peaks; and mouse, 23,443 peaks). **c**, Cumulative distribution of distances between each APRE and the closest annotated TSS normalized by the average intergenic distance of the species (amphioxus, 83,471; zebrafish, 252,774; medaka, 174,139; and mouse, 216,857 APREs, as per Fig. 1c). **d**, Signal **d**istribution of different marks within functional-genomic regions in amphioxus. log_10_ of read counts of H3K4me3, H3K27ac and ATAC-seq, and raw read counts of CAGE-seq in promoters of homology-supported, protein-coding genes (*n* = 26,501), other APREs (‘O. APREs’, all APREs that do not overlap a TSS from any gene model; *n* = 48,341), proximal APREs (*n* = 24,622), distal APREs (*n* = 11,881), previously validated enhancers (*n* = 43; Supplementary Table 9), random regions (*n* = 88,413) and negative regions (excluding ATAC-seq peaks, *n* = 88,413). For region designation, see Fig. 1c. For clarity, whiskers and outliers are not displayed. **e**, *k*-means clustering of APREs based on H3K27ac signal in three developmental stages. Cluster 1 and 3 APREs were considered as active and inactive, respectively. Average H3K27ac profiles are represented in the top panels. The number of APREs per cluster and stage are provided in Supplementary Data 2, dataset 8. **f**, Alluvial plot that shows the dynamics of each APRE among the clusters described in **e**. APREs that remained active (cluster 1 in all stages) along the three developmental stages are represented in blue, constitutively inactive APREs (cluster 3 in all stages) in dark grey and dynamic APREs in red or orange (if inactivated or activated, respectively, during development). Five groups of APREs of special interest are highlighted with stronger colours and named GR1–GR5. **g**, Representative enriched DNA motifs found in each of the groups described in **f**. GR1 APREs were enriched in early motifs (for example, Smad3 and Oct4, Sox2 and Nanog); GR3 APREs in motifs of transcription factors involved in the generation of the three germ layers (for example, Foxo3, Sox6 and Sox17); GR4 APREs in tissue-specific transcription factors (for example, Foxa2, Otx2 and Crx); and GR5 APREs in CTCF and CTCF-like (BORIS) motifs. *q* values as provided by Homer. **h**, Lateral views of embryos from stable transgenic zebrafish lines at 24 hpf (except for Foxa-243, at 48 hpf) showing GFP expression driven by the amphioxus APREs listed in Supplementary Table 8 and highlighted in Supplementary Fig. 1. The number of independent founders with the same expression were as follows: Six1/2-182 (5/5), Foxa-243 (3/3), Foxa-251 (4/4), FoxC-3067 (6/6) and Pax1/9-157 (3/3). Midbrain expression corresponds to the positive-control enhancer included in the reporter constructs. EN, endoderm; HB, hindbrain; MY, myotomes; PA, pharyngeal arch; SC, spinal cord. Scale bar, 250 μm. **i**, Lateral v**i**ews of transient transgenic amphioxus embryos, showing GFP expression driven by the APREs highlighted in Supplementary Fig. 1a, b (Foxa-251 (*n* = 46 out of 52) and Foxc-3067 (*n* = 27 out of 35), respectively) and in a previous study^71^ (Hox-1655, *n* = 72 out of 80). For each element, left panels correspond to 3D rendering from sub-stacks and right panels to *z*-stack sagittal sections. Scale bar, 50 μm. Anterior is to the left and dorsal to the top.

We generated an exhaustive resource of genomic, epigenomic and transcriptomic data for the Mediterranean amphioxus (*B. lanceolatum*), comprising a total of 52 sample types (Fig. 1a and Supplementary Data 2, datasets 1–5). These datasets were mapped to a *B. lanceolatum* genome that was sequenced and assembled de novo, with 150× coverage, a total size of 495.4 Mbp, a scaffold N50 of 1.29 Mbp and 4% gaps (Extended Data Fig. 1a–c). To facilitate access by the research community, we integrated these resources into a UCSC Genome Browser track hub (Fig. 1b; available at http://amphiencode.github.io/Data/), together with an intra-cephalochordate sequence conservation track and a comprehensive annotation of repetitive elements (Extended Data Fig. 1d–f) and long non-coding RNAs (Extended Data Fig. 1g and Supplementary Data 2, dataset 6). To enable broader evolutionary comparisons, we reconstructed orthologous gene families for multiple vertebrate and non-vertebrate species (Supplementary Data 2, dataset 7), generated several equivalent datasets for zebrafish and medaka (Extended Data Fig. 2a), and built a dedicated server for synteny comparisons (Extended Data Fig. 1h).

A comprehensive functional annotation of the *B. lanceolatum* genome identified 88,391 putative *cis*-regulatory elements of DNA as defined by assay for transposase-accessible chromatin using sequencing (ATAC-seq) (these elements are hereafter referred to as APREs), as well as 20,569 protein-coding genes supported by orthology. We divided the APREs into promoters—around transcription start sites (TSSs), which were highly supported by cap analysis gene-expression sequencing (CAGE-seq) data, Extended Data Fig. 2b—and gene-body, proximal and distal APREs (Fig. 1c). Equivalent analyses using zebrafish data yielded 256,018 potential regulatory regions, with a significantly higher proportion of these being distal APREs (Fig. 1c; *P* < 2.2 × 10^−16^, one-sided Fisher’s exact test). A significantly larger global TSS distance in APREs was observed for all vertebrates compared to amphioxus (Fig. 1d), even after correcting for differences in average intergenic length among species (Extended Data Fig. 2c; *P* < 2.2 × 10^−16^ for all vertebrate-versus-amphioxus comparisons, one-sided Mann–Whitney tests). Amphioxus APREs showed enrichment for enhancer-associated chromatin marks (Extended Data Fig. 2d), which were highly dynamic during embryo development (Extended Data Fig. 2e-g), and consistently drove GFP expression in zebrafish or amphioxus transgenic assays (93% (14/15), Fig. 1e and Extended Data Fig. 2h, i). Moreover, 89% (32/36) of previously reported amphioxus enhancers overlapped APREs defined by our data. Therefore, a large fraction of APREs probably act as developmentally regulated transcriptional enhancers.

## Disentangling vertebrate bidirectional promoters

**Extended Data Fig. 3:**
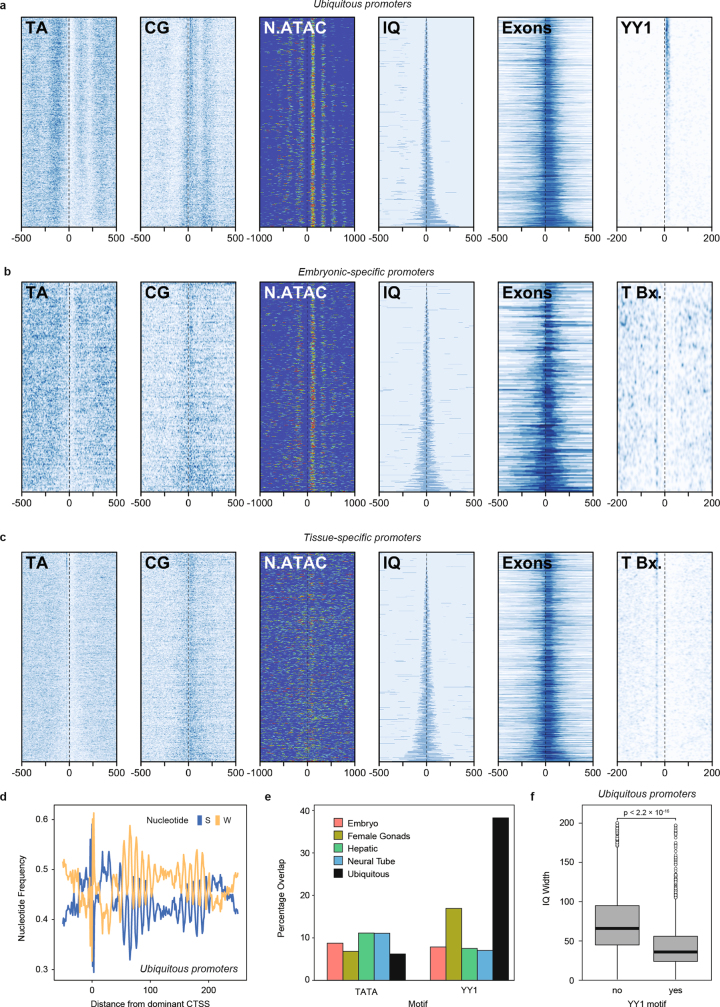
Features of amphioxus promoters derived from CAGE-seq. **a**–**c**, Heat maps showing AT and CG signal, nucleosome positioning (derived from the NucleoATAC signal), promoter width (interquantile (IQ) range), first exon length and YY1 (**a**) or TATA box (**b**, **c**) motifs around ubiquitous (**a**, *n* = 3,710), embryonic-specific (**b**, *n* = 1,451) and tissue-specific (**c**, *n* = 4,154) promoters, sorted by promoter width. Position 0 corresponds to the main TSS. **d**, Ubiquitous promoters show strong evidence for a nucleosome positioned downstream of the CAGE TSS, as judged from the 12-bp periodicity of W and S nucleotide density. **e**, Per cent of promoters of each category that have associated TATA box or YY1 motifs. Number of promoters: embryo, 1,451; female gonads, 1,494; hepatic, 2,420; neural tube, 1,734; and ubiquitous, 3,710. **f**, IQ width distribution of ubiquitous promoters (*n* = 3,710) with and without an associated YY1 motif. *P* value corresponds to two-sided Wilcoxon sum-rank tests.

**Extended Data Fig. 4:**
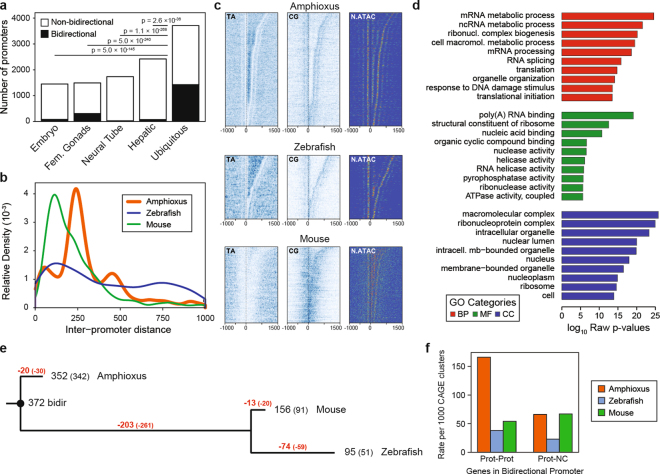
Characteristics and evolution of bidirectional promoters. **a**, Number of bidirectional and non-bidirectional promoters identified for each regulatory category. *P* values correspond to two-sided Fisher’s exact tests against ubiquitous promoters. **b**, Distribution of distance between bidirectional promoters in each species (amphioxus, 1,975; zebrafish, 549; and mouse, 876 pairs of promoters). The distance between amphioxus peaks closely corresponds to integral nucleosome spacing. **c**, Heat maps of TA, CG and nucleosome occupancy (derived from the NucleoATAC signal) around bidirectional promoter pairs in amphioxus (*n* = 1,975), mouse (*n* = 876) and zebrafish (*n* = 549), arranged by the distance between the two CAGE TSSs. In amphioxus, both TA and NucleoATAC signals indicate regions in which 0, 1 or 2 nucleosomes separate promoters. **d**, Enriched GO terms for genes associated with bidirectional promoters in amphioxus. Uncorrected *P* values correspond to two-sided Fisher’s exact tests as provided by topGO. **e**, Inferred evolutionary dynamics of 372 putatively ancestral bidirectional promoters among chordate groups. Red, number of inferred losses and disentanglements; black, number of detected bidirectional promoters by CAGE-seq (in brackets) or microsynteny (neighbouring genes in a 5′ to 5′ orientation) for each species. In parentheses, number of lost and disentangled (red) or retained (black) bidirectional promoters when considering only the cases supported by CAGE-seq. **f**, In vertebrates, disentanglement was not accompanied by a general increase in the fraction of bidirectional promoters with antisense non-coding transcription, as shown by the relative number of CAGE clusters identified as bidirectional promoters that are composed of two protein-coding genes (‘Prot-Prot’) or of one protein-coding and one non-coding or non-annotated locus (‘Prot-NC’). The total number of uniquely annotated, protein-coding-associated CAGE promoters was amphioxus, 11,789; mouse, 13,654; and zebrafish, 14,014.

Analyses of core promoters, defined by CAGE-seq, at single-nucleotide resolution revealed that amphioxus promoters display a mixture of pan-metazoan, pan-vertebrate and unique features (Extended Data Fig. 3 and Supplementary Information). These analyses also identified that 25% (3,950/15,884) of neighbouring protein-coding genes were arranged in bidirectional promoters. Bidirectional promoters were most common among ubiquitous promoters (Extended Data Fig. 4a), displayed a marked periodicity in the distance between promoters (Extended Data Fig. 4b, c) and were associated with genes that were significantly enriched in housekeeping functions (Extended Data Fig. 4d). Notably, the fraction of bidirectional promoters defined by CAGE-seq decreased progressively from amphioxus to mouse (12.83% (1,752/13,654)) and to zebrafish (7.84% (1,098/14,014)), which suggests a disentanglement of ancestral bidirectional promoters after each round of WGD (two in tetrapods and three in teleosts). Consistently, the majority of a set of 372 putatively ancestral, bidirectional promoters were lost in vertebrates—particularly in stem vertebrates (54.5%)—with only very few amphioxus-specific losses (5.3%) (Extended Data Fig. 4e, f).

## Developmental DNA demethylation of APREs

**Fig. 2:**
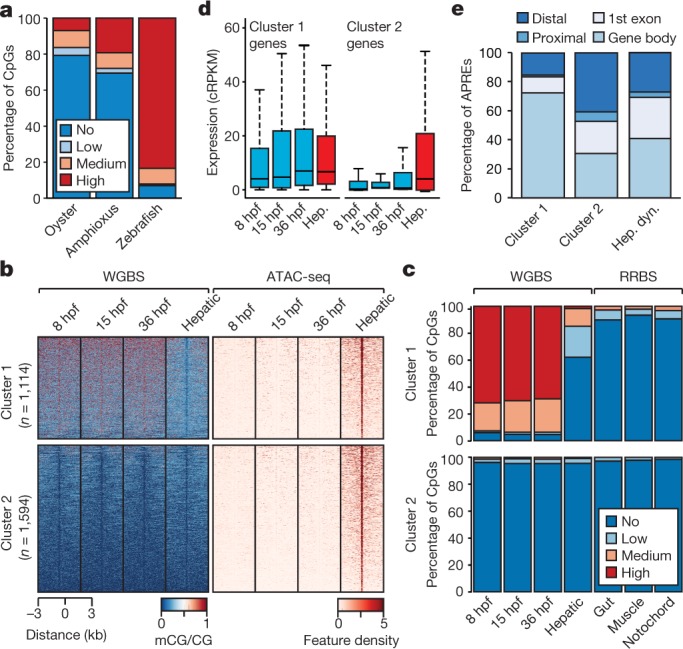
5mC patterns and dynamics in the amphioxus genome. **a**, Percentage of methylated CpG dinucleotides in oyster (mantle, *n* = 14,779,123), amphioxus (8 hpf, *n* = 19,657,388) and zebrafish (1,000-cell stage, *n* = 38,989,847) samples. Low, >0–20%; medium, 20–80%; high, >80%. **b**, *k*-means clustering (*n* = 2) of 5mC signal over hepatic-specific APREs. **c**, Percentage of methylated CpG dinucleotides as assessed by whole-genome bisulfite sequencing (WGBS) and reduced representation bisulfite sequencing (RRBS) in embryos and adult tissues in APREs from **b**. **d**, Distribution of expression levels for genes associated with APREs displaying distinct 5mC patterns in **b**. Cluster 1: 1,114 genes; cluster 2: 1,594 genes. cRPKM, corrected (per mappability) reads per kb of mappable positions and million reads. Hep, hepatic diverticulum. **e**, Genomic distribution of regions with distinct 5mC patterns from **b**. Hep. dyn., dynamic APREs active in the hepatic diverticulum.

**Extended Data Fig. 5:**
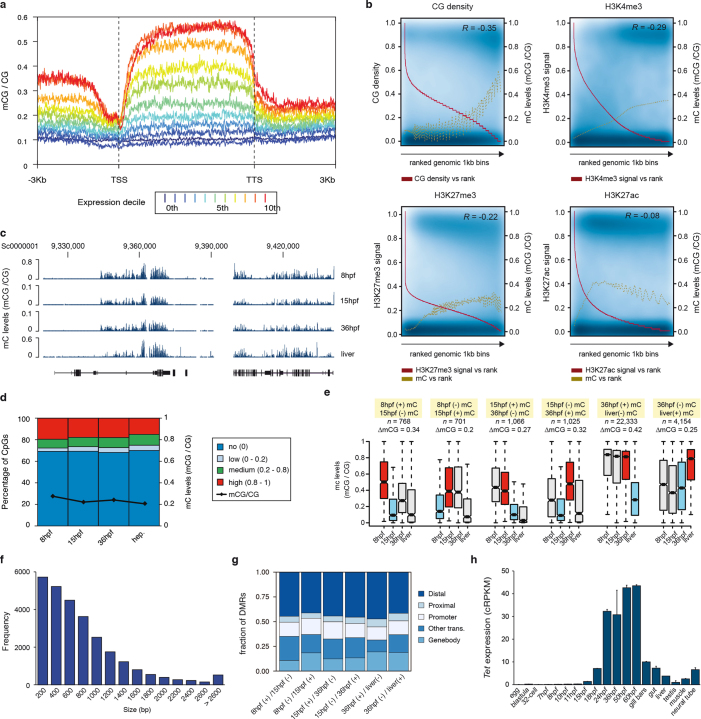
5mC dynamics in amphioxus. **a**, 5mC levels across gene bodies (*n* = 20,569) from different expression deciles (0^th^, not expressed; 10^th^, highest expression). TTS, transcription termination site. **b**, Scatter plots of levels of 5mC and CpG density, H3K4me3, H3K27me3 and H3K27ac in 1-kbp genomic bins sorted on the basis of feature rank. The red line tracks anti-correlation between feature density and rank number (a low rank number implies high feature density). The golden line represents a smoothing spline of 5mC signal versus feature rank number. Pearson correlation coefficients (*R*) are displayed in the top right corner of each panel. **c**, UCSC browser excerpt of 5mC patterns for selected regions. **d**, Percentage of methylated CpG dinucleotides in 8-hpf (*n* = 19,657,388), 15-hpf (*n* = 21,247,615), 36-hpf (*n* = 21,702,000) and hepatic (adult, *n* = 19,240,245) amphioxus samples. Black line indicates the fraction between methylated and non-methylated CpGs at each stage. **e**, Box plots of average 5mC levels in different types of differentially methylated regions (DMRs) at each stage. ΔmCG denotes the change in the fraction of methylated CpGs between the two stages used for identification of DMRs (red (hyper) and blue (hypo) boxes). The number of DMRs were as follows: 8 hpf(+)–15 hpf(−), 768; 8 hpf(−)–15 hpf(+), 701; 15 hpf(+)–36 hpf(−),1,066; 15 hpf(−)–36 hpf(+), 1,025; 36 hpf(+)–liver(−), 22,333; and 36 hpf(−)–liver(+), 4,154. The coordinates for all DMRs are provided in Supplementary Data 2, dataset 11. **f**, Distribution of DMR sizes (in bp). **g**, Genomic distribution of DMRs identified for each sample. ‘Other trans.’, DMRs that overlap with gene models that were not defined as being supported by orthology. **h**, Expression (cRPKMs) of the amphioxus *Tet* orthologue in embryos and adult tissues. Error bars represent standard error of the mean (the number of replicates for each RNA-seq dataset is provided in Fig. 1a).

Similar to other non-vertebrates^17–19^, the amphioxus genome exhibited very low levels of CpG methylation (Fig. 2a); nearly all of the 5mC occurred in gene bodies, in which the proportion of methylated CpGs correlated positively with gene-expression levels but negatively with the density of H3K27me3 and H3K4me3 histone marks and CpG dinucleotides (Extended Data Fig. 5a–c). However, as in zebrafish and frogs^7^, global levels of 5mC displayed a decrease during development (Extended Data Fig. 5d–g), coinciding with the onset of expression of the amphioxus orthologue of TET demethylase (Extended Data Fig. 5h).

**Extended Data Fig. 6:**
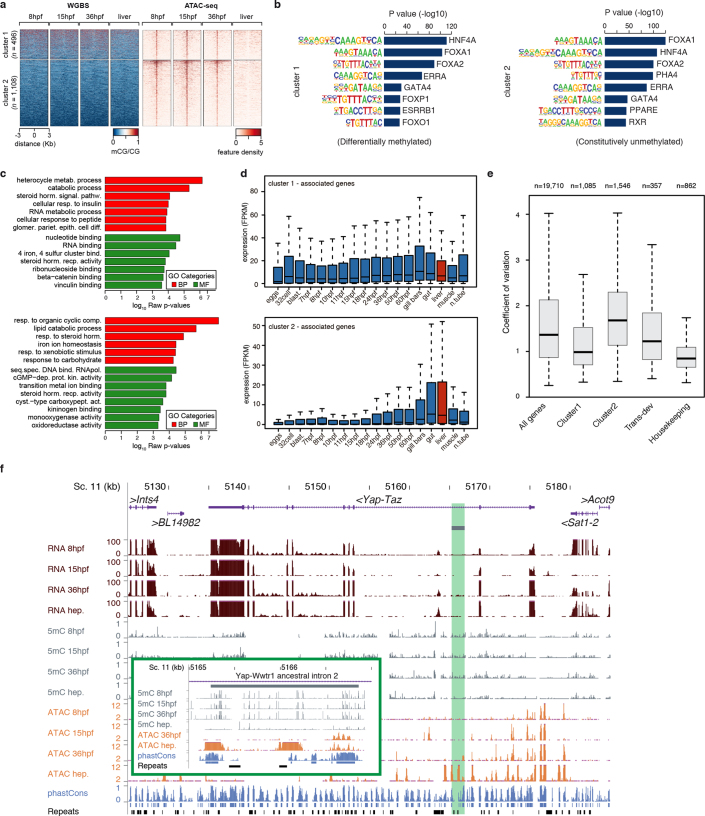
Developmental 5mC dynamics at APREs in amphioxus. **a**, *k*-means clustering (*n* = 2) of 5mC signal over embryo-specific open-chromatin regions (that is, APREs), assessed by ATAC-seq (Supplementary Table 10). **b**, The most significantly enriched transcription-factor binding-site motifs in APREs that display different developmental 5mC patterns in Fig. 2b. Uncorrected *P* values as provided by MEME. All plotted motifs had Benjamini-corrected *q* values of 0. **c**, GO enrichment for genes associated with cluster 1 (top) or cluster 2 (bottom) APREs from Fig. 2b. Uncorrected *P* values correspond to two-sided Fisher’s exact tests as calculated by topGO. **d**, Distribution of expression values (cRPKMs) across all samples for genes associated with cluster 1 (top, *n* = 1,114) or cluster 2 (bottom, *n* = 1,594) APREs from Fig. 2b. **e**, Distribution of the coefficients of variation for genes associated with cluster 1 or cluster 2 APREs from Fig. 2b, as well as all (*n* = 19,710), trans-dev (*n* = 357) and house-keeping (*n* = 862) amphioxus genes. **f**, Example of a potentially conserved (zebrafish to amphioxus) DMR associated with *yap1*, a major transcription factor of the Hippo pathway. The inset corresponds to the region highlighted in green. The two ohnologous genomic regions in zebrafish are shown in Supplementary Fig. 2. Additional cases included genes that contained APREs that are likely to regulate neighbouring liver-specific genes (‘bystander’ genes) (Supplementary Table 11). The number of replicates for each experiment displayed in each track is provided in Fig. 1a.

To assess whether these 5mC dynamics may have regulatory potential, we identified adult hepatic diverticulum-specific APREs that are inactive during development. Unlike embryo-specific APREs (Extended Data Fig. 6a), the clustering of these adult APREs on the basis of 5mC content revealed two distinct subsets, one with hepatic-specific and one with constitutive hypomethylation (Fig. 2b). Differentially methylated APREs (cluster 1) also displayed robust hypomethylation in other adult tissues (Fig. 2c), which suggests that demethylation at these APREs occurs organism-wide. Both groups of hepatic-specific APREs were enriched for binding sites of liver-specific transcription factors—such as Hnf4a—as well as broadly expressed transcription factors such as Foxa (Extended Data Fig. 6b), which is a pioneer factor that participates in 5mC removal at regulatory regions in mammals^20^.

APREs from both clusters were preferentially associated with genes with metabolic functions (Extended Data Fig. 6c). However, only APREs with hepatic-specific hypomethylation (cluster 1) were primarily associated with genes that displayed steady widespread expression (Fig. 2d and Extended Data Fig. 6d, e); these APREs were mainly located within gene bodies (Fig. 2e). These data suggest that demethylation of these APREs may contribute to their identification as adult-specific, transcriptional *cis*-regulatory elements within continuously hypermethylated gene-body contexts, which is characteristic of non-vertebrate species. Fourteen zebrafish gene families contained differentially methylated APREs in introns that are orthologous to those identified in amphioxus—amongst these are four genes that encode components of the Hippo pathway, including the transcriptional effectors Yap (*yap1* and *wwtr1*) and Tead (*tead1a* and *tead3a*) (Extended Data Fig. 6f, g).

## The hourglass model and chordate embryogenesis

**Fig. 3:**
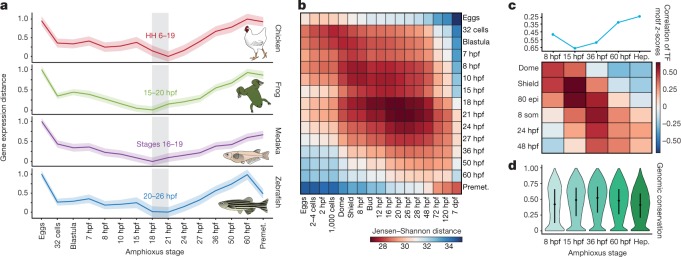
The hourglass model and chordate embryogenesis. **a**, Stages of minimal transcriptomic divergence (using the Jensen–Shannon distance metric) from four vertebrate species to each amphioxus stage. The grey box outlines the period of minimal divergence, with the corresponding vertebrate periods indicated (the range is given by the two less divergent stages). Dispersions correspond to the standard deviation computed on 100 bootstrap re-samplings of the orthologue sets (amphioxus–chicken: 5,720; amphioxus–zebrafish: 5,673; amphioxus–frog: 5,883; and amphioxus–medaka: 5,288). HH, Hamburger–Hamilton stage. **b**, Heat map of pairwise transcriptomic Jensen–Shannon distances between amphioxus (vertical) and zebrafish (horizontal) stages. A smaller distance (red) indicates higher similarity. dpf, days post-fertilization. **c**, Zebrafish and amphioxus pairwise Pearson correlation of relative enrichment *z*-scores for transcription-factor (TF) motifs in dynamic APREs, active at different developmental stages. Top, maximal correlation for each amphioxus stage against the zebrafish stages. Bottom, heat map with all pairwise correlations. 80 epi, 80% epiboly stage; 8 som, 8-somite stage. **d**, Sequence conservation levels within the cephalochordates of active APREs at each developmental stage, visualized as the distribution of average phastCons scores. The number of APREs at 8 hpf = 5,282; at 15 hpf = 17,387; at 36 hpf = 21,089; at 60 hpf = 22,674; and in hepatic diverticulum (hep) **=** 16,551. Dots correspond to the mean values and lines represent the interquartile range.

**Extended Data Fig. 7:**
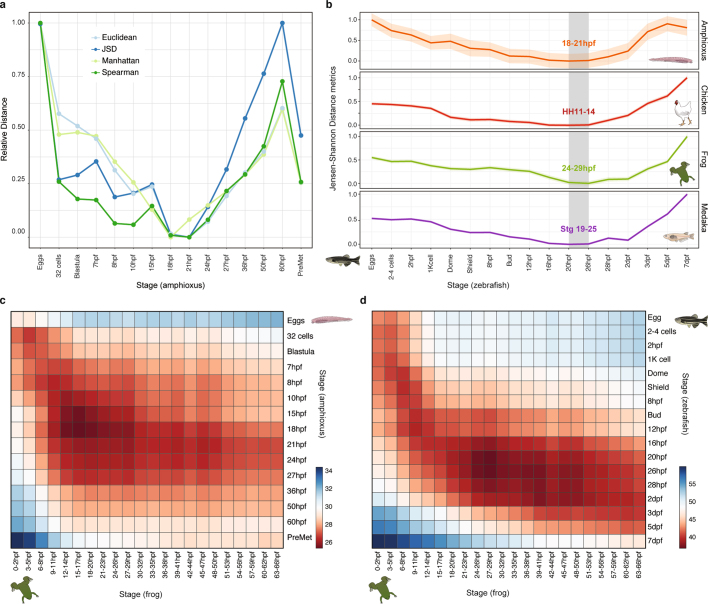
Periods of maximal transcriptomic similarity across chordate development. **a**, Stages of minimal transcriptomic distance obtained in the comparison between amphioxus and zebrafish for four alternative distance methods (Euclidean, Manhattan and Jensen–Shannon distances, and Spearman correlation). Values are normalized to minimal (0) and maximal (1) for each metric. **b**, Stages of minimal transcriptomic divergence shown as the smallest Jensen–Shannon distance between zebrafish stages and four chordate species. The shaded area surrounding the line that connects the stages is the standard deviation, derived from 100 bootstrap replicates of the orthologous gene set. The grey box outlines the ‘phylotypic’ period of minimal divergence; the corresponding periods are indicated for each species as the range provided by the two closest stages. **c**, **d**, Heat maps of pairwise transcriptomic distances (Jensen–Shannon distance metric) between pairs of chordate species, amphioxus and frog (**c**), and zebrafish and frog (**d**). In both heat maps, the smallest distance (red) indicates maximal similarity of the transcriptome. The periods of minimal divergence of the transcriptome are earlier for the amphioxus–frog comparison than for the zebrafish–frog comparison.

Previous comparative analyses among vertebrate transcriptomes^21,22^ showed a developmental period of maximal similarity in gene expression that coincides with the so-called phylotypic period, consistent with the hourglass model^23^. However, similar comparisons with tunicates and amphioxus have thus far not resolved a phylotypic period shared across all chordates^22^. Pairwise comparisons of stage-specific RNA sequencing (RNA-seq) data from developmental time courses of amphioxus against zebrafish, medaka, frog (*Xenopus tropicalis*) and chicken revealed a consistent period of highest similarity (Fig. 3a, b and Extended Data Fig. 7) that occurred slightly earlier than those reported for vertebrates; in amphioxus, this corresponds to the neurula at the 4–7-somite stage (18–21 hours post fertilization (hpf)). At the regulatory level, pairwise comparisons between the relative enrichment of transcription-factor motifs in sets of dynamic APREs that were active at each stage were also consistent with an earlier hourglass model^24^ (Fig. 3c). By contrast, at a shorter timescale, comparisons between different species of amphioxus showed that the sequence conservation for the same APREs was higher after the putative chordate phylotypic period (Fig. 3d).

## Regulatory conservation shapes chordate body plan

**Fig. 4:**
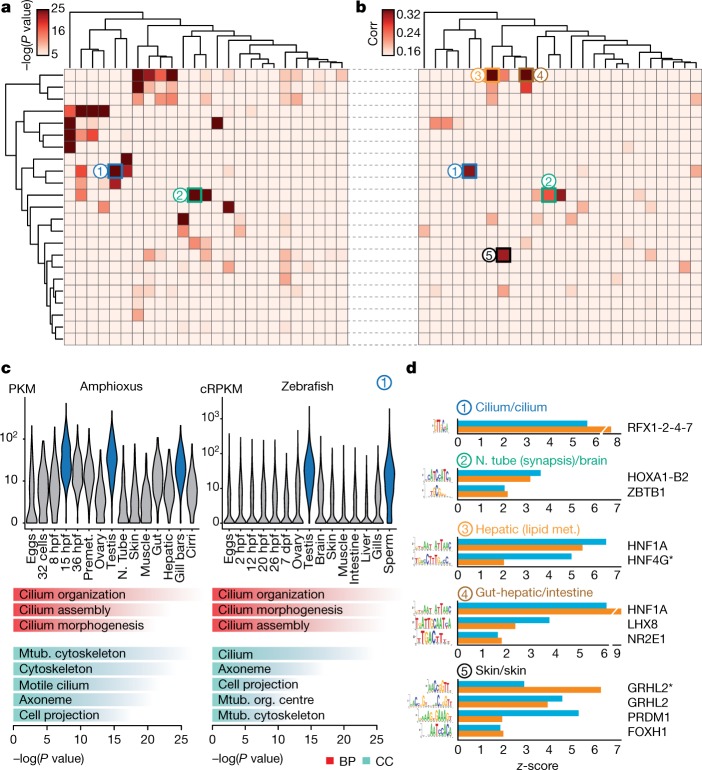
Transcriptomic and *cis*-regulatory conservation of adult chordate tissues. **a**, Heat map showing the level of raw statistical significance of orthologous gene overlap between modules produced by weighted correlation network analysis (WGCNA), from amphioxus (vertical) and zebrafish (horizontal) as derived from upper-tail hypergeometric tests. **b**, Heat map of all pairwise Pearson correlations (corr) between the modules of the two species, based on the relative *z*-scores of transcription-factor motifs for each module (242 super-families of motifs). Modules are clustered as in **a**. **c**, Distribution of expression values (cRPKMs) for all genes within the cilium modules across each sample (top), and enriched Gene Ontology terms within each module (bottom) for a pair of modules (labelled ‘1’ in **b**, **c**; 1,681 and 261 genes in zebrafish and amphioxus, respectively). BP, biological process; CC, cellular component. *P* values correspond to uncorrected two-sided Fisher’s exact tests as provided by topGO. Mtub., microtubule; N. tube, neural tube; org., organizing. **d**, Transcription-factor binding-site motifs with high *z*-scores from highly correlated pairs of modules between zebrafish (blue) and amphioxus (orange). Numbers correspond to those circles in **b**. RFX1-2-4-7 denotes RFX1, RFX2, RFX4 and RFX7; HOXA1-B2 denotes HOXA1 and HOXB2; asterisk denotes an alternative motif.

**Extended Data Fig. 8:**
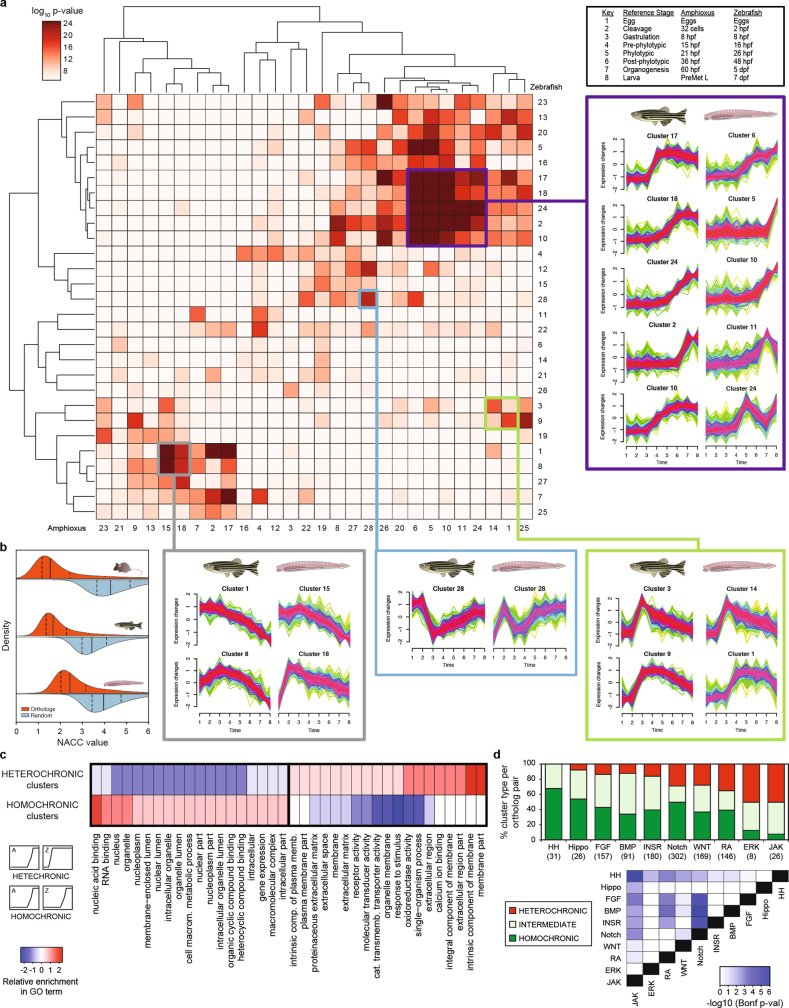
Comparison of temporal gene expression profiles in amphioxus and zebrafish. **a**, Heat map showing the significance of orthologous gene overlap between Mfuzz clusters across eight matched developmental stages in amphioxus and zebrafish as derived from an upper-tail hypergeometric test. Some clusters with highly significant overlap are highlighted, and their corresponding temporal expression profiles are shown. The profiles of all clusters for the two species are included in Supplementary Figs. 3, 4. Exact *P* values and sample sizes are provided in Supplementary Data 2, dataset 8. **b**, Distributions of NACC values for orthologous genes (in red) or random orthology assignments (blue) for each species against human. Lower NACC values imply higher conservation of relative expression. Solid lines show the median, and the dashed lines mark the interquartile range. The number of orthologue pairs were as follows: mouse, 15,109; zebrafish, 16,480; and amphioxus, 8,633. **c**, Differentially enriched GO terms among pairs of zebrafish and amphioxus Mfuzz clusters with significant orthologue overlap (*P* < 10^−10^ upper-tail hypergeometric test) with homochronic (48 pairs) and heterochronic (35 pairs) patterns. The GO enrichment of a group was calculated as the number of cluster pairs with significant enrichment for that given term (Supplementary Data 2, dataset 12). **d**, Top, per cent of zebrafish genes from each developmental pathway we studied, based on the temporal similarity of their corresponding Mfuzz cluster (homochronic, heterochronic or intermediate). Only genes belonging to clusters with significant orthologue overlap were analysed; the number of genes is provided in parenthesis below the pathway name. Bottom, pairwise comparisons between developmental pathway distributions. *P* values correspond to Bonferroni-corrected, two-sided, three-way Fisher’s exact tests.

Additional comparisons of embryo transcriptomes and neighbourhood analysis of conserved co-expression^25^ showed a high conservation of developmental and global expression patterns and of gene functions between amphioxus and vertebrates (Extended Data Fig. 8 and Supplementary Information). Further pairwise comparison of co-regulated gene modules across tissues between amphioxus and zebrafish revealed multiple pairs with highly significant levels of orthologue overlap (Fig. 4a). These included modules with conserved tissue-specific expression that were enriched for coherent Gene Ontology categories, including genes with high expression in organs with ciliated cells (for example, spermatozoa and gill bars) (labelled ‘1’ in Fig. 4a–c) as well as neural, muscle, gut, liver, skin and metabolism-related modules (Supplementary Data 1). We also found a significant positive correlation between relative motif-enrichment scores for many pairs of modules (Fig. 4b); the most-enriched transcription-factor motifs within each cluster were highly consistent between amphioxus and zebrafish (Fig. 4d).

## Higher regulatory information in vertebrate genomes

**Fig. 5:**
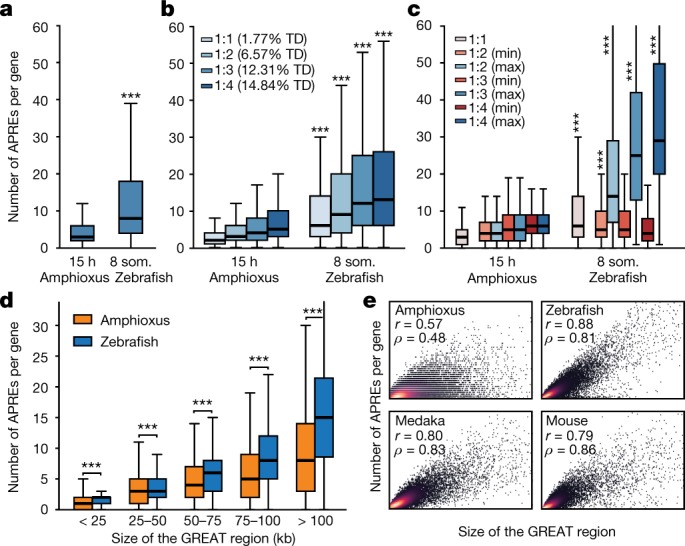
Higher regulatory complexity in vertebrate regulatory landscapes. **a**, Distribution of the number of APREs within the regulatory landscape of each gene (as estimated by GREAT), at comparable pre-phylotypic developmental stages (15 hpf for amphioxus and 8 somites for zebrafish). *n* = 6,047 and 9,239 genes for amphioxus and zebrafish, respectively. **b**, As in **a**, but with gene families separated according to the number of retained ohnologues per family in vertebrates (from 1 to 4, using mouse as a reference). The percentage of developmental regulatory genes (TD, trans-dev) in each category is indicated. **c**, As in **b**, but only the genes with the lowest (‘min’, in red) and the highest (‘max’, in blue) number of APREs are plotted for each ohnologue family. **d**, Distributions of the number of APREs per gene among subsets of amphioxus and zebrafish genes matched by GREAT-region size (± 500 bp) and binned by size as indicated. **e**, Density scatter plot of the number of APREs (*y* axis) versus the size of the GREAT region (*x* axis) per gene and species. Pearson (*r*) and Spearman (*ρ*) correlation coefficients are indicated. Sample sizes: amphioxus, 20,053; zebrafish, 20,569; medaka, 15,978; mouse, 18,838. **a**–**d**, *** *P* < 0.001; one-sided Mann–Whitney tests of the zebrafish distribution versus the equivalent amphioxus distribution. Exact *P* values and sample sizes are provided in Supplementary Data 2, dataset 8.

**Extended Data Fig. 9:**
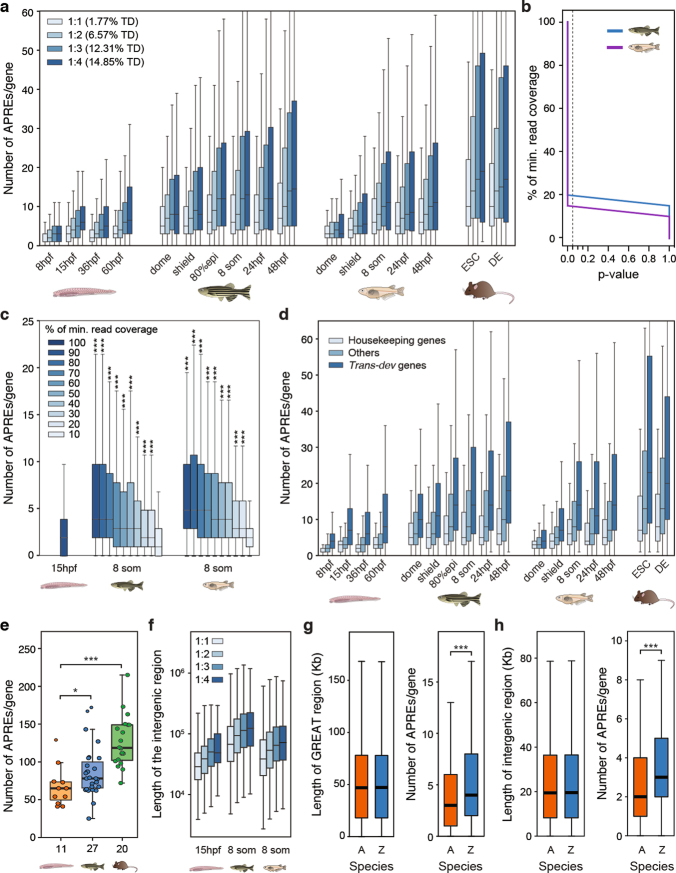
Higher regulatory content in vertebrate genomes. **a**, Distribution of the number of APREs per the regulatory landscape of a gene (as determined by GREAT^26^), at different developmental stages or cell lines of four chordate species (amphioxus, zebrafish, medaka and mouse). Orthologous gene families are split according to the number of ohnologues that are retained per family (from 1 to 4, using mouse as a reference species for the ohnologue counts). The percentage of developmental regulatory genes (trans-dev, TD) in each category is indicated. **b**, *P* values of one-sided Mann–Whitney *U* tests against the amphioxus peak-number distribution using 100% of the minimum read coverage for different levels of down-sampling of the zebrafish and medaka samples. **c**, Distribution of the number of APREs in the GREAT region of the gene, called after down-sampling the reads of the two vertebrate samples to different fractions of the sample with the minimum effective coverage in our study (~21 reads per kbp for the 36-hpf sample in amphioxus). Asterisks correspond to the significance of the *P* values of Mann–Whitney *U* tests against the amphioxus peak-number distribution using 100% of the minimum-read coverage. The number of genes per box was as follows: amphioxus, 20,569; zebrafish, 20,053; and medaka, 15,978. **d**, As in **a**, but with gene families separated according to functional categories (housekeeping, trans-dev and others). **e**, Number of APREs per regulatory landscape determined using 4C-seq, for 58 members of 11 trans-dev families. The number of genes probed in each species is indicated on the *x* axis. **f**, Distribution of the length of the intergenic regions from the genes plotted in **a** for the indicated stages. **g**, Distributions of GREAT-region sizes (left) and number of APREs per gene (right) for a subset of 10,186 pairs of genes with matched GREAT-region size distributions (±500 bp) in amphioxus and zebrafish. **h**, Distributions of intergenic-region sizes (left) and number of APREs per gene (right) for a subset of 13,941 pairs of genes with matched intergenic-region size distributions (±500 bp) in amphioxus and zebrafish. *P* values correspond to Mann–Whitney *U* tests: *0.05 > *P* value ≥ 0.01, **0.01 > *P* value ≥ 0.001, ***P value < 0.001. In **a** and **d**, all comparisons between e**a**ch **d**istribution of a vertebrate species and the equivalent distribution in amphioxus produced significant *P* values (*P* value < 0.001); for simplicity, in these panels asterisks are not shown. Exact *P* values and sample sizes are provided in Supplementary Data 2, dataset 8.

To investigate the effect of WGDs on the evolution of vertebrate gene regulation, we first asked whether the number of putative regulatory regions per gene is higher in vertebrates than in amphioxus. We observed significantly more APREs in the regulatory landscape of each gene (as defined by the ‘Genomic Regions Enrichment of Annotations Tool’ (GREAT)^26^) in zebrafish than in amphioxus (Fig. 5a). This difference is particularly evident for gene families that have retained multiple copies after WGD (known as ohnologues; Fig. 5b), for which the number of APREs is very uneven between copies, with marked regulatory expansions observed for some ohnologues (Fig. 5c). The same patterns were detected for all developmental stages of amphioxus and zebrafish, as well as for medaka and mouse genomes, and were highly robust to down-sampling of ATAC-seq coverage in vertebrates (Extended Data Fig. 9a–c). We also detected a higher number of peaks associated with regulatory genes (‘trans-dev’ genes that are involved in the regulation of embryonic development) compared to housekeeping genes in all species (Extended Data Fig. 9d), consistent with the higher frequency of retention of trans-dev genes in multiple copies after WGD^3^ (Fig. 5b). Comparison of regulatory landscapes—determined experimentally using circular chromosome conformation capture followed by sequencing (4C-seq)—for 58 genes from 11 trans-dev gene families in amphioxus, zebrafish and mouse showed similar results (Extended Data Fig. 9e).

As expected, the higher number of APREs in zebrafish was associated with larger intergenic regions in this species (Extended Data Fig. 9f). However, the differences in APRE complements were not attributable only to an increase in genome size in vertebrates, as subsets of amphioxus and zebrafish genes with matched distributions of GREAT or intergenic-region lengths also displayed a higher number of APREs in zebrafish (Extended Data Fig. 9g, h). Further investigation of matched distributions showed that these differences were particularly great in genes with large regulatory landscapes (>50 kb) (Fig. 5d). Thus, larger regions in amphioxus did not scale at the same rate as in vertebrates in terms of regulatory complexity (Fig. 5e), which is consistent with the overall lower proportion of distal APREs identified in this species (Fig. 1c, d). In summary, these analyses reveal a large increase in the number of regulatory regions during vertebrate evolution (and/or a decrease in these regions in amphioxus)—particularly of distal regulatory elements—and that this trend is enhanced for specific gene copies retained after the WGDs, pointing to unequal rates of regulatory evolution for different ohnologues.

## More-complex regulation in specialized ohnologues

**Fig. 6:**
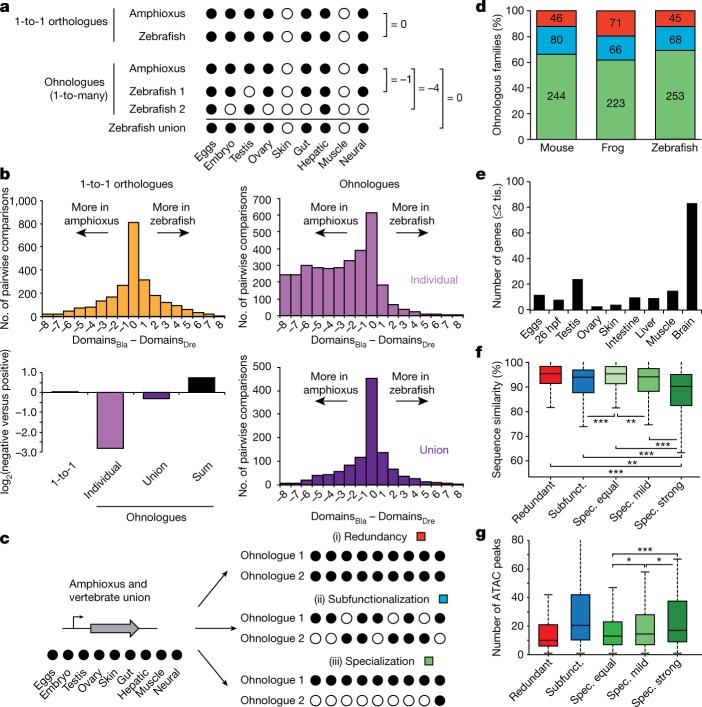
Expression specialization is the main fate after WGD. **a**, Schematic of the analysis shown in **b**. Expression is binarized for each gene across the nine homologous samples (‘on’, black dots; normalized cRPKM > 5). **b**, Distribution of the difference in positive domains between zebrafish (domains_Dre_) and amphioxus (domains_Bla_) for 1-to-1 orthologues (2,478 gene pairs; yellow), individual ohnologues (3,427 gene pairs in 1,135 families; lilac) and the union of all vertebrate ohnologues in a family (purple). Bottom left, log_2_ of the ratio between zebrafish genes with negative and positive score for each category. ‘Sum’ (black), binarization of family expression after summing the raw expression values for all ohnologues. **c**, Schemati**c** of the analyses shown in **d**, representing the three possible fates after WGD. **d**, Distribution of fates after WGD for families of ohnologues. **e**, Number of ohnologues with strong specialization in zebrafish expressed in each domain. Tis., tissue. **f**, Distribution of the percentage of nucleotide sequence similarity between human and mouse by family type. Ohnologues from specialized families are divided into ‘spec. equal’ (which maintain all expression domains), ‘spec. mild’ (which have lost some but maintained more than two expression domains) and ‘spec. strong’ (≤2 expression domains remain). Subfunct., subfunctionalized. **g**, Distribution of the number of APREs within GREAT regions for zebrafish ohnologues for each category. Only statistical comparisons within specialized families are shown. *P* values in **f** and **g** correspond to two- and one-sided Wilcoxon sum-rank tests between the indicated groups, respectively. *0.05 > *P* value ≥ 0.01, ** 0.01 > *P* value ≥ 0.001, ***P value < 0.001. Exact *P* values and sample sizes are provided in Supplementary Data 2, dataset 8.

**Extended Data Fig. 10:**
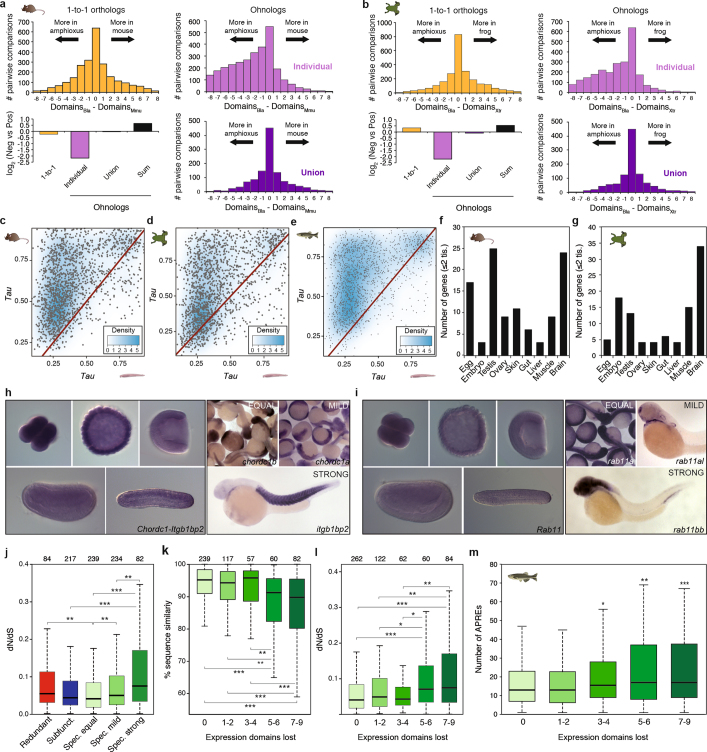
Regulatory evolution after vertebrate WGD. **a**, **b**, For each mouse (**a**) or frog (**b**) gene, the number of positive-expression domains across nine equivalent samples is subtracted from the number of domains in which the single amphioxus orthologue is expressed. The distribution of the difference in domains between the amphioxus and the vertebrate species is plotted for 1-to-1 orthologues (2,450 and 2,484 gene pairs for mouse and frog, respectively; yellow), individual ohnologues (3,011 and 2,637 gene pairs in 1,212 and 1,094 families for mouse and frog, respectively; lilac) and the union of all vertebrate ohnologues in a family (purple). Bottom left, log_2_ of the ratio between the sum of all mouse (**a**) or frog (**b**) genes with negative versus positive score for each orthology group. ‘Sum’ (black), binarization of family expression is performed after summing the raw expression values for all ohnologues. **c**–**e**, Density scattered plot of the *τ* values for pairs of mouse (**c**, *n* = 1,502), frog (**d**, *n* = 1,495) and zebrafish (**e**, *n* = 1,498) and amphioxus orthologues from multi-gene families in vertebrates. **f**, **g**, Number of ohnologues with strong specialization (≤2 remaining expression domains) in mouse (**f**) or frog (**g**) expressed in each tissue or developmental stage. **h**, **i**, Representative in situ hybridization assays in zebrafish embryos for different members of specialized families (right) and for the single amphioxus orthologue (left) (Chordc1 and Itgb1bp2 (**h**) and Rab11 (**i**)). Zebrafish image data for this paper were retrieved from the Zebrafish Information Network (ZFIN), University of Oregon, Eugene, OR 97403-5274; (http://zfin.org/, accessed May 2018) and are used with the permission of B. Thisse. Amphioxus in situ hybridization was performed once using 10 embryos per probe, all of which showed the same expression pattern. **j**, Distribution of the dN/dS ratio between human and mouse for different classes of ohnologues based on their fate after WGD. **k**, **l**, Distribution of the percentage of nucleotide sequence similarity (**k**) or dN/dS ratio (**l**) between human and mouse for ohnologues grouped by the number of expression domains lost. **m**, Distribution of the number of APREs within GREAT regions for zebrafish ohnologues grouped by the number of expression domains lost. *P* values in **j**–**m** correspond to Wilcoxon sum-rank tests. *0.5 > *P* value ≥ 0.01; **0.01 > *P* value ≥ 0.001; ***P value < 0.001.

The duplication–degeneration–complementation (DDC) model hypothesizes that the retention of duplicate genes could be driven by reciprocal loss of regulatory elements and restriction of paralogues to distinct subsets of the ancestral expression pattern^27^. In particular, the DDC model predicts that individual paralogues would each have more restricted expression than an unduplicated outgroup, but that their summation would not. To test this, we binarized the expression (‘on’ or ‘off’) of each gene in nine homologous expression domains in amphioxus, zebrafish, frog and mouse (Fig. 6a). When comparing genes that returned to single-copy status after WGDs, we detected no expression bias between amphioxus and vertebrates (Fig. 6a, b and Extended Data Fig. 10a, b). By contrast, when vertebrate ohnologues were compared to their single amphioxus orthologues, the distributions were strongly skewed and many vertebrate genes displayed far more restricted expression domains (Fig. 6b and Extended Data Fig. 10a, b; similar results were obtained by comparing *τ* values^28^, Extended Data Fig. 10c–e). The symmetrical pattern was fully recovered when the expression of all vertebrate members was combined, or when the raw expression values were summed for each member within a paralogy group (Fig. 6a, b and Extended Data Fig. 10a, b).

Although the above findings are consistent with the DDC model, they are also compatible with an alternative model in which a subset of duplicate genes becomes more ‘specialized’ in expression pattern while one or more paralogues retain the broader ancestral expression^29^. To distinguish between these alternatives, we analysed a subset of multi-gene families in which both the single amphioxus orthologue and the union of the vertebrate ohnologues—and thus probably the ancestral gene—were expressed across all nine samples that we compared. We then identified (i) gene families in which all vertebrate paralogues were expressed in all domains (termed ‘redundancy’), (ii) gene families in which none of the vertebrate members had expression across all domains (termed ‘subfunctionalization’)^27^ and (iii) gene families in which one or more vertebrate ohnologues were expressed in all domains, but at least one ohnologue was not (termed ‘specialization’) (Fig. 6c). We obtained very similar results for the three vertebrate species we studied (Fig. 6d): between 80 and 88% of gene families were subfunctionalized or specialized, which implies that ancestral expression domains have been lost in at least one member. Moreover, specialization was consistently more frequent than subfunctionalization as a fate for ohnologues with broad ancestral expression.

Ohnologues that have experienced strong specialization (≤2 remaining expression domains) retained expression more often in neural tissues (Fig. 6e and Extended Data Fig. 10f–i) and were generally not expressed in additional vertebrate-specific tissues (Supplementary Information). Furthermore, they showed the fastest rates of sequence evolution (Fig. 6f and Extended Data Fig. 10j–l), consistent with an optimization of their coding sequence to perform their function in a specific tissue and/or with the evolution of novel functions (neofunctionalization). Ohnologues from specialized families that have lost expression domains showed significantly more associated APREs than ohnologues with the full ancestral expression (Fig. 6g). We observed a strong positive relationship between the number of ancestral expression domains lost and the number of APREs associated with specialized ohnologues (Extended Data Fig. 10m). This implies that the specialization of gene expression after WGD does not occur primarily through loss of ancestral tissue-specific enhancers, but rather by a complex remodelling of regulatory landscapes that involves recruitment of novel, tissue-specific regulatory elements.

## Discussion

By applying functional genomics approaches to the cephalochordate amphioxus, we have deepened our understanding of the origin and evolution of chordate genomes. We identified APREs in amphioxus, the activation of which is tightly associated with differential DNA demethylation in adult tissues—a mechanism previously thought to be specific to vertebrates. Additional cases may be subsequently found in other non-vertebrate species when similar multi-omics datasets are analysed. In amphioxus, APREs of this type usually fall within gene bodies of widely expressed genes, which suggests that gene regulation by demethylation could have originated as a mechanism to allow better definition of enhancers in a hyper-methylated intragenic context. If so, this mechanism could have been co-opted into new genomic contexts—that is, distal intergenic enhancers—later in the evolution of vertebrate genomes, which are characterized by their pervasive, genome-wide hypermethylation.

We also found a consistently higher number of open chromatin regions per gene in vertebrates than in amphioxus. This pattern is observed at a genome-wide level, but is particularly evident for distal APREs and in gene families that retain multiple ohnologues after WGD; these families are enriched for regulatory genes with large regulatory landscapes. Finally, we detected a large degree of specialization in expression for retained ohnologues, with the vast majority of multi-gene families with broad ancestral expression having at least one member that restricted its expression breadth. Through this mechanism, vertebrates have increased their repertoire of tightly regulated genes, which has potentially contributed to tissue-specific evolution. Gene-expression specialization was accompanied by faster evolution of protein-coding sequences, and by an increase–rather than a decrease—in the number of regulatory elements. Taken together, these observations indicate that the two rounds of WGD not only caused an expansion and diversification of gene repertoires in vertebrates, but also allowed functional and expression specialization of the extra copies by increasing the complexity of their gene regulatory landscapes. We suggest that these changes to the gene regulatory landscapes underpinned the evolution of morphological specializations in vertebrates.

## Methods

No statistical methods were used to predetermine sample size. The experiments were not randomized and investigators were not blinded to allocation during experiments and outcome assessment.

### Animal husbandry and embryo staging

Amphioxus gametes were obtained by heat stimulation as previously described^30,31^. Embryos were obtained by in vitro fertilization in filtered seawater and cultured at 19 °C. Staging was done based on previous publications^32,33^; correspondence between developmental stages and hpf are provided in Supplementary Table 1. All protocols used for vertebrate species (zebrafish and medaka) have been approved by the Institutional Animal Care and Use Ethic Committee (PRBB–IACUEC, for CRG) or the Ethics Committee of the Andalusian Government (license numbers 450-1839 and 182-41106, for CABD-CSIC), and implemented according to national and European regulations. All experiments were carried out in accordance with the principles of the 3Rs (replacement, reduction and refinement).

### Genome sequencing and assembly

Genomic DNA was extracted from a single *B. lanceolatum* adult male collected in Argeles-sur-Mer, France. The genome was sequenced using a combination of Illumina libraries from a range of inserts at Genoscope (897 million reads in total, with a paired-end coverage of 150×; Supplementary Table 2). A diploid assembly was generated using SOAPdenovo assembler^34^ using a *k*-mer of 71. After gap closing, haplotypes were reconciled with Haplomerger^35^.

### Genome annotation

We generated deep coverage RNA-seq for 16 developmental stages and 9 adult tissues (4.16 billion reads in total). The bulk of strand-specific transcriptomic data was assembled de novo with Trinity^36^, aligned and assembled into loci with the PASA pipeline^37^. De novo gene models were built using Augustus^38^ and subsequently refined with EVM^39^ using PASA assemblies and aligned proteins from other species. In parallel, all strand-specific RNA-seq reads were mapped to the genome using Tophat2^40^, assembled using Cufflinks^41^ and open reading frames were predicted using Trans-decoder^42^. Models obtained using both these approaches were reconciled yielding a total 218,070 transcripts from 90,927 unified loci, of which 20,569 were protein-coding and had homologues in at least one of the other studied species (see ‘Comparative genomics’). Gene Ontology (GO) terms were assigned to amphioxus proteins based on their PFAM and Interpro domains, as well as blastp hits against human proteins (1 × 10^−6^).

Repeats were annotated and filtered with RepeatMasker using a custom library generated with RepeatModeller. Long non-coding RNAs were identified by filtering all transcripts for protein-coding potential using CPAT^43^ trained with zebrafish transcripts, and further discarding those that had a positive hit in a HMM search against the NR and PFAM databases (Extended Data Fig. 1g).

### Comparative genomics

We used OMA^44^ to reconstruct gene families and infer homology relationships based on well-established phylogenetic relationships between species^45^, and further merged families sharing Ensembl paralogues with ‘Euteleostomi’ or ‘Vertebrata’ ancestry. To define the set of high-confidence ohnologue families (Supplementary Data 2, dataset 9), we retained families with two to four copies in three out of five vertebrates (excluding teleosts) and subjected them to phylogenetic reconciliation.

To assess genome sequence conservation, reciprocal whole-genome alignments of *Branchiostoma floridae*, *Branchiostoma belcheri* and *B. lanceolatum* were performed using LASTZ and processed with phastCons^46^ to produce conservation scores. The distribution of phastCons scores in APREs was determined using ‘dynamic’ ATAC-seq peaks that showed no temporal discontinuity in activity.

### Comparative transcriptomics

To investigate the evolutionary conservation of chordate development at the molecular level, newly generated data from zebrafish, medaka and amphioxus, as well as available data from the SRA (frog and chicken), were compared (Supplementary Data 2, dataset 3 and Supplementary Table 3). Gene expression was estimated with Kallisto^47^ using Ensembl transcriptome annotations (Supplementary Table 4), and summing up transcripts per million (TPMs) from all transcript isoforms to obtain one individual gene-expression estimate per sample. We used single-copy orthologues to pair genes and used the Jensen–Shannon distance metrics after quantile normalization of TPMs to score distance between pairs of transcriptomes:

$$JS{D}_{s}=\sqrt{\frac{1}{2}\sum _{g=0}^{{n}_{{\rm{og}}}}{p}_{g}\times log\left(\frac{{p}_{g}}{\frac{1}{2}\left({p}_{g}+{q}_{g}\right)}\right)+\frac{1}{2}\sum _{g=0}^{{n}_{{\rm{og}}}}{q}_{g}\times log\left(\frac{{q}_{g}}{\frac{1}{2}\left({p}_{g}+{q}_{g}\right)}\right)}$$

Statistical robustness towards gene sampling was assessed by calculating transcriptomic distances based on 100 bootstrap replicates and estimating the standard deviation over these replicates.

To obtain groups of genes with similar dynamics of expression during development, genes were clustered based on their cRPKMs^48^ using the Mfuzz package^49^. For this purpose, eight comparable stages were selected in amphioxus and zebrafish on the basis of conserved developmental landmarks such as fertilization, gastrulation and organogenesis (Supplementary Table 5). The statistical significance of the orthologous gene overlap between pairs of clusters was assessed using upper-tail hypergeometric tests.

Modules of co-expressed genes across stages and adult tissues were inferred using WGCNA^50^ with default parameters in amphioxus (17 samples) and zebrafish (27 samples) (Supplementary Table 6). The statistical significance of the orthologous gene overlap between pairs of clusters was assessed using upper-tail hypergeometric tests. The numbers of transcription-factor binding-site motifs detected in APREs in the basal regions of genes from any given cluster were standardized using *z*-scores.

To have a general assessment of the extent of conservation or divergence in gene expression among chordates at adult stages, we used neighbourhood analysis of conserved co-expression (NACC)^25^, a method developed to compare heterogeneous, non-matched sample sets across species. NACC relies on comparisons of average distances between pairs of orthologous (genes A and B), the 20 genes with the closest transcriptomic distance ($\bar{A}$ and $\bar{B}$) and their reciprocal orthologues in the other species ($\overline{AB}$ and $\overline{BA}$), and is calculated as follows:

$$NACC=\frac{1}{2}\left[\left(\overline{AB}-\bar{A}\right)+\left(\overline{BA}-\bar{B}\right)\right]$$

NACC calculations were performed for each family that contained a single amphioxus member and up to eight members in zebrafish and were also performed with randomized orthology relationships as a control.

### Regulatory profiling

#### ATAC-seq

For amphioxus, medaka and zebrafish, ATAC-seq was performed in two biological replicates by directly transferring embryos in the lysis buffer, following the original protocol^51,52^. ATAC-seq libraries were sequenced to produce an average of 66, 83 and 78 million reads for amphioxus, zebrafish and medaka, respectively. Reads were mapped with Bowtie2 and nucleosome-free pairs (insert < 120 bp) retained for peak-calling using MACS2^53^, and the irreducible discovery rate was used to assess replicability. Nucleosome positioning was calculated from aligned ATAC-seq data using NucleoATAC^54^

#### Chromatin immunoprecipitation with sequencing (ChIP–seq)

Embryos of undetermined gender were fixed in 2% formaldehyde and ChIP was performed as previously described for other species^55^. Chromatin was sonicated and incubated with the corresponding antibody (H3K4me3: ab8580, H3K27ac: ab4729 and HeK27me3: ab6002, from Abcam). An average of 30 million reads per library was generated. Reads were mapped with Bowtie2 and peaks called with MACS2^53^, assuming default parameters.

#### 4C-seq

Embryos of undetermined gender were fixed in 2% formaldehyde and chromatin was digested with DpnII and Csp6. Specific primers targeted the TSSs of the studied genes and included Illumina adapters. An average 5 million reads were generated for each of the two biological replicates. After mapping, reads were normalized per digestion fragment cut and interactions were identified using peakC^56^ with low-coverage regions excluded.

#### MethylC-seq and RRBS

Genomic DNA was extracted as previously described^57^, sonicated, purified and end-repaired. Bisulfite conversion was performed with the MethylCode Bisulfite Conversion Kit (Thermo Fisher Scientific). After Illumina library construction, an average of 73 million reads per sample were sequenced. RRBS libraries were prepared similarly to those for MethyC-seq, but with restriction digestion with MspI instead of sonication and PCR amplification. An average of 46 million reads per sample was generated. Reads were mapped to an in silico, bisulfite-converted *B. lanceolatum* reference genome^7,58^. Differentially methylated regions in the CpG context were identified as previously described^7^. Differential transcription-factor motif enrichment was obtained with DiffBind from Bioconductor.

#### CAGE-seq

Libraries were constructed using the non-amplifying non-tagging Illumina CAGE protocol^59^. Mouse CAGE-seq data were obtained from FANTOM5^60^. Reads were aligned using Bowtie. Nearby individual CAGE TSSs were combined using the distance-based clustering method in CAGEr^61^ to produce tag clusters, which summarize expression at individual promoters. Tag clusters were clustered across samples to produce comparable promoter regions, referred to as ‘consensus clusters’. The consensus clusters were then grouped by expression patterns using a self-organizing map^62^. We investigated the relative presence and enrichment of the following features: TATA box, YY1 motif, GC and AT content, SS and WW dinucleotides, first exons and nucleosome positioning signal. Heat maps were plotted for visualization by scanning either for exact dinucleotide matches or for position weight matrix matches at 80% of the maximum score. Position weight matrices for TATA and YY1 were taken from the JASPAR vertebrate collection.

### *Cis*-regulatory comparisons

Depending on the analysis, an APRE was associated with a specific gene if it was located within: (i) the ‘basal’ region of the gene (−5 kb to +1 kb of the TSS; for comparisons of enriched motif composition) or (ii) the GREAT region of the gene (up to ±1 Mb of the TSS unless another basal region was found; for comparing the number of APREs per gene)^26^. Stratification of gene sets by GREAT or intergenic-region size between amphioxus and zebrafish was done using the function stratify from the matt suite^63^, with a range of ±500 bp.

The DNA-binding specificity of each transcription factor was predicted on the basis of the binding domain similarity to other transcription-factor family members, as previously performed^64^. Transcription-factor motifs from CIS-BP version 1.02^64^ were downloaded and clustered using GimmeMotifs^65^ (*P* ≤ 0.0001). Two hundred and forty-two clusters of motifs were assigned to one or more orthologous groups in both amphioxus and zebrafish and used for all analyses (Supplementary Data 2, dataset 10). These motifs were detected in APREs using the tools gimme threshold and gimme scan from GimmeMotifs^65^.

### Effect of WGDs on gene expression

Gene expression was binarized (1 if the normalized cRPKM > 5, and 0 otherwise) across nine comparable samples in amphioxus and three vertebrate species (mouse, frog and zebrafish) (Supplementary Table 7). Then, for each amphioxus gene and vertebrate orthologue, the expression bias was measured by subtracting the number of positive-expression domains in amphioxus from that of vertebrates (Fig. 6a). The amphioxus gene-expression pattern was also compared to the union of the ohnologues, as well as the pattern after binarizing the expression for the sum of cRPKM values of all family members. The analysis was restricted to families with a single member in amphioxus

Next, we selected those ohnologue families for which the ancestral expression included the nine studied domains, as inferred from having expression in the single amphioxus orthologue and in the union of the family. For each gene family, we then defined (Fig. 6c): (i) redundancy (all vertebrate paralogues were expressed in all domains), (ii) subfunctionalization (none of the vertebrate members had expression across all domains^27^), and (iii) specialization (one or more vertebrate ohnologues were expressed in all domains, but at least one ohnologue was not). Members of the later type were subdivided into ‘strong’ and ‘mild’ specialization if they retained ≤ 2 or more expression domains. We examined the transcript sequence similarity as well as the dN/dS between human and mouse (retrieved from Biomart), and the number of APREs associated with genes from different categories. Finally, we computed the *τ* tissue-specificity index as previously described^28^, to assess more broadly the tissue specificity of ohnologues.

### Transgenic assays in zebrafish and amphioxus

Enhancer reporter assays in zebrafish embryos were performed as previously described^66^. Selected peaks were first amplified, cloned into a PCR8/GW/TOPO vector and transferred into a detection vector (including a *gata2* minimal promoter, a GFP reporter gene and a strong midbrain enhancer (z48) as an internal control)^67^. Transgenic embryos were generated using the Tol2 transposon and transposase method^68^. Three or more independent stable transgenic lines were generated for each construct as reported in Supplementary Table 8. For amphioxus reporter assays, selected peaks were amplified and transferred into a detection vector (including the *Branchiostoma* minimal actin promoter, a GFP reporter gene and piggyBac terminal repeats). Transgenic embryos were generated by the piggyBac transposase method.

### In situ hybridization

Gene fragments that were synthetically designed or amplified by PCR from cDNA were sub-cloned into pBluescript II SK and used as templates for probe synthesis using the DIG labelling kit (Roche) and T3 RNA polymerase. Embryos at different developmental stages were fixed in PFA 4% dissolved in MOPS–EGTA buffer and in situ hybridization carried out as previously described^69^, using BCIP/NBT as a chromogenic substrate.

### Reporting summary

Further information on research design is available in the Nature Research Reporting Summary linked to this paper.

### Code availability

Custom code is available at https://gitlab.com/groups/FunctionalAmphioxus.

## Online content

Any methods, additional references, Nature Research reporting summaries, source data, statements of data availability and associated accession codes are available at 10.1038/s41586-018-0734-6.

## Supplementary information

Supplementary Information This file contains Supplementary Text and Data, Supplementary References, Supplementary Figures 1-9 and full guides for Supplementary Datasets 1-17 and Supplementary Tables 1-19.

Reporting Summary

Supplementary Data 1 This file contains the detailed annotation and expression patterns for each module of co-regulated genes identified by WGCNA in amphioxus and zebrafish. Section descriptions: **1** - Amphioxus and zebrafish module annotation and comparisons. **a**, Name assigned to each module (color) based on gene expression and/or GO enrichment. **b**, Same heatmaps as in Fig. 4a,b including the names of each module. **2 -** Clustered heatmap of TF-motifs vs amphioxus & zebrafish modules. **a**, WGCNA modules from the two species are plotted against all motif clusters. The values visualized are the z-scores of each motif in each module. Modules and motifs are clustered based on the correlation of the visualized z-scores. **b**, Key for TF motif super-families in a, ordered by size of the motif name. **3 -** Amphioxus module RNA-seq expression and GO terms. For each amphioxus module, boxplots without whiskers showing the median and interquartile range of gene expression levels (using the cRPKM metrics) across RNA-seq samples (top), and significantly enriched GO categories (bottom). P-values correspond to uncorrected p-values from two-sided Fisher's exact tests as calculated by topGO. Number of genes per module is provided in SI Dataset 8. **4 -** Zebrafish module RNA-seq expression and GO terms. For each zebrafish module, boxplots without whiskers showing the median and interquartile range of gene expression levels (using the cRPKM metrics) across RNA-seq samples (top), and significantly enriched GO categories (bottom). P-values correspond to uncorrected p-values from two-sided Fisher's exact tests as calculated by topGO. Number of genes per module is provided in SI Dataset 8.

Supplementary Data 2 This zipped file contains Supplementary Datasets 1-17 – see Supplementary Information document for a full Supplementary Dataset guide.

Supplementary Tables This file contains Supplementary Tables 1-19 – see Supplementary Information document for a full Supplementary Table guide.

## Data Availability

Next-generation sequencing data have been deposited in Gene Expression Omnibus (GEO) under the following accession numbers: GSE106372 (ChIP-seq), GSE106428 (ATAC-seq), GSE106429 (CAGE-seq), GSE106430 (RNA-seq), GSE102144 (MethylC-seq and RRBS) and GSE115945 (4C-seq). Raw genome sequencing data and the genome assembly have been submitted to European Nucleotide Archive (ENA) under the accession number PRJEB13665. UCSC hub and annotation files are available at http://amphiencode.github.io/.
