# Supplementary material for: Amphioxus functional genomics and the origins of vertebrate gene regulation
Source: Nature. 2018 Nov 21;564(7734):64–70. doi: 10.1038/s41586-018-0734-6 (PMC6292497; doi:10.1038/s41586-018-0734-6)
Supplement: Supplementary file 1 — This file contains Supplementary Text and Data, Supplementary References, Supplementary Figures 1-9 and full guides for Supplementary Datasets 1-17 and Supplementary Tables 1-19. [file 41586_2018_734_MOESM1_ESM.pdf]

nature SUPPLEMENTARY INFORMATION
https://doi.org/10.1038/s41586-018-0734-6
In the format provided by the authors and unedited.

Amphioxus functional genomics and the origins of vertebrate gene regulation

Ferdinand Marlétaz[1,2,41], Panos N. Firbas[3,41], Ignacio Maeso[3,41]*, Juan J. Tena[3,41], Ozren Bogdanovic[4,5,6,41], Malcolm Perry[7,8,41], Christopher D. R. Wyatt[9,10], Elisa de la Calle-Mustienes[3], Stephanie Bertrand[11], Demian Burguera[9,12], Rafael D. Acemel[3], Simon J. van Heeringen[13], Silvia Naranjo[3], Carlos Herrera-Ubeda[12], Ksenia Skvortsova[4], Sandra Jimenez-Gancedo[3], Daniel Aldea[11], Yamile Marquez[9], Lorena Buono[3], Iryna Kozmikova[14], Jon Permanyer[9], Alexandra Louis[15,16,17], Beatriz Albuixech-Crespo[12], Yann Le Petillon[11], Anthony Leon[11], Lucie Subirana[11], Piotr J. Balwierz[7,8], Paul Edward Duckett[4], Ensieh Farahani[3], Jean-Marc Aury[18], Sophie Mangenot[18], Patrick Wincker[19], Ricard Albalat[20], Èlia Benito-Gutiérrez[21], Cristian Cañestro[20], Filipe Castro[22], Salvatore D'Aniello[23], David E. K. Ferrier[24], Shengfeng Huang[25], Vincent Laudet[11], Gabriel A. B. Marais[26], Pierre Pontarotti[27], Michael Schubert[28], Hervé Seitz[29], Ildiko Somorjai[30], Tokiharu Takahashi[31], Olivier Mirabeau[32], Anlong Xu[25,33], Jr-Kai Yu[34], Piero Carninci[35,36], Juan Ramon Martinez-Morales[3], Hugues Roest Crollius[15,16,17], Zbynek Kozmik[14], Matthew T. Weirauch[37,38], Jordi Garcia-Fernàndez[12], Ryan Lister[6,39], Boris Lenhard[7,8,40], Peter W. H. Holland[1], Hector Escriva[11]*, Jose Luis Gómez-Skarmeta[3]* & Manuel Irimia[9,10]*

[1]Department of Zoology, University of Oxford, Oxford, UK. [2]Molecular Genetics Unit, Okinawa Institute of Science and Technology Graduate University, Onna-son, Japan. [3]Centro Andaluz de Biología del Desarrollo (CABD), CSIC-Universidad Pablo de Olavide-Junta de Andalucía, Seville, Spain. [4]Genomics and Epigenetics Division, Garvan Institute of Medical Research, Sydney, New South Wales, Australia. [5]St Vincent's Clinical School, Faculty of Medicine, University of New South Wales, Sydney, New South Wales, Australia. [6]Australian Research Council Centre of Excellence in Plant Energy Biology, School of Molecular Sciences, The University of Western Australia, Crawley, Western Australia, Australia. [7]Institute of Clinical Sciences, Faculty of Medicine, Imperial College London, London, UK. [8]Computational Regulatory Genomics, MRC London Institute of Medical Sciences, London, UK. [9]Centre for Genomic Regulation (CRG), Barcelona Institute of Science and Technology (BIST), Barcelona, Spain. [10]Universitat Pompeu Fabra (UPF), Barcelona, Spain. [11]Biologie Intégrative des Organismes Marins, BIOM, Observatoire Océanologique, CNRS and Sorbonne Université, Banyuls sur Mer, France. [12]Department of Genetics, Microbiology and Statistics, Faculty of Biology, and Institut de Biomedicina (IBUB), University of Barcelona, Barcelona, Spain. [13]Department of Molecular Developmental Biology, Faculty of Science, Radboud Institute for Molecular Life Sciences, Radboud University, Nijmegen, The Netherlands. [14]Institute of Molecular Genetics of the Czech Academy of Sciences, Prague, Czech Republic. [15]Institut de Biologie de l'ENS, IBENS, Ecole Normale Supérieure, Paris, France. [16]Inserm, U1024, Paris, France. [17]CNRS, UMR 8197, Paris, France. [18]Genoscope, Institut de biologie François-Jacob, Commissariat à l'Energie Atomique (CEA), Université Paris-Saclay, Evry, France. [19]Génomique Métabolique, Genoscope, Institut de biologie François Jacob, Commissariat à l'Energie Atomique (CEA), CNRS, Université Evry, Université Paris-Saclay, Evry, France. [20]Department of Genetics, Microbiology and Statistics, Faculty of Biology and Institut de Recerca de la Biodiversitat (IRBio), University of Barcelona, Barcelona, Spain. [21]Department of Zoology, University of Cambridge, Cambridge, UK. [22]Interdisciplinary Centre of Marine and Environmental Research (CIIMAR/CIMAR) and Faculty of Sciences (FCUP), Department of Biology, University of Porto, Porto, Portugal. [23]Biology and Evolution of Marine Organisms, Stazione Zoologica Anton Dohrn Napoli, Naples, Italy. [24]The Scottish Oceans Institute, Gatty Marine Laboratory, University of St Andrews, St Andrews, UK. [25]State Key Laboratory of Biocontrol, School of Life Sciences, Sun Yat-sen University, Guangzhou, China. [26]Laboratoire de Biométrie et Biologie Evolutive (UMR 5558), CNRS and Université Lyon 1, Villeurbanne, France. [27]IRD, APHM, Microbe, Evolution, PHylogénie, Infection, IHU Méditerranée Infection and CNRS, Aix Marseille University, Marseille, France. [28]Sorbonne Université, CNRS, Laboratoire de Biologie du Développement de Villefranche-sur-Mer, Institut de la Mer de Villefranche-sur-Mer, Villefranche-sur-Mer, France. [29]UMR 9002 CNRS, Institut de Génétique Humaine, Université de Montpellier, Montpellier, France. [30]Biomedical Sciences Research Complex, School of Biology, University of St Andrews, St Andrews, UK. [31]School of Medical Sciences, Faculty of Biology, Medicine and Health, University of Manchester, Manchester, UK. [32]INSERM U830, Équipe Labellisée LNCC, SIREDO Oncology Centre, Institut Curie, PSL Research University, Paris, France. [33]School of Life Sciences, Beijing University of Chinese Medicine, Beijing, China. [34]Institute of Cellular and Organismic Biology, Academia Sinica, Taipei, Taiwan. [35]RIKEN Center for Life Science Technologies (Division of Genomic Technologies) (CLST DGT), Yokohama, Japan. [36]Laboratory for Transcriptome Technology, RIKEN Center for Integrative Medical Sciences, Yokohama, Japan. [37]Center for Autoimmune Genomics and Etiology, Divisions of Biomedical Informatics and Developmental Biology, Cincinnati Children's Hospital Medical Center, Cincinnati, OH, USA. [38]Department of Pediatrics, University of Cincinnati College of Medicine, Cincinnati, OH, USA. [39]Harry Perkins Institute of Medical Research, Nedlands, Western Australia, Australia. [40]Sars International Centre for Marine Molecular Biology, University of Bergen, Bergen, Norway. [41]These authors contributed equally: Ferdinand Marlétaz, Panos N. Firbas, Ignacio Maeso, Juan J. Tena, Ozren Bogdanovic, Malcolm Perry. *e-mail: nacho.maeso@gmail.com; hescriva@obs-banyuls.fr; jlgomska@upo.es; mirimia@gmail.com

© 2018 Springer Nature Limited. All rights reserved.

Supplementary Materials for:

Amphioxus functional genomics reveals the evolution of vertebrate regulatory traits

Ferdinand Marletaz, Panos N. Firbas, Ignacio Maeso*, Juan J. Tena, Ozren Bogdanovic, Malcolm Perry, Christopher D.R. Wyatt, Elisa de la Calle-Mustienes, Stephanie Bertrand, Demian Burguera, Rafael D. Acemel, Simon J. van Heeringen, Silvia Naranjo, Carlos Herrera-Ubeda, Ksenia Skvortsova, Sandra Jimenez-Gancedo, Daniel Aldea, Yamile Marquez, Lorena Buono, Iryna Kozmikova, Jon Permanyer, Alexandra Louis, Beatriz Albuixech-Crespo, Yann Le Petillon, Anthony Leon Florian, Lucie Subirana, Piotr Balwierz, Paul Edward Duckett, Ensieh Farahani, Jean Marc Aury, Sophie Mangenot, Patrick Wincker, Ricard Albalat, Èlia Benito-Gutiérrez, Cristian Cañestro, Filipe Castro, Salvatore D'Aniello, David E.K. Ferrier, Shengfeng Huang, Vincent Laudet, Gabriel A.B. Marais, Pierre Pontarotti, Michael Schubert, Hervé Seitz, Ildiko Somorjai, Tokiharu Takahashi, Olivier Mirabeau, Anlong Xu, Jr-Kai Yu, Piero Carninci, Juan Ramon Martinez-Morales, Hugues Roest Crollius, Zbynek Kozmik, Matt T. Weirauch, Jordi Garcia-Fernàndez, Ryan Lister, Boris Lenhard, Peter W.H. Holland, Hector Escriva*, Jose Luis Gómez-Skarmeta*, Manuel Irimia*

* Corresponding authors:

IM, nacho.maeso@gmail.com
HE, hescriva@obs-banyuls.fr
JLGS, jlgomska@upo.es
MI, mirimia@gmail.com

SUPPLEMENTARY INFORMATION

1) BACKGROUND ON THE EUROPEAN AMPHIOXUS

1.1) History and phylogenetic position

Cephalochordates, usually known as amphioxus or lancelets, were first described by Peter Simon Pallas from a specimen collected at the coast of Cornwall [1], and were classified as mollusks (*Limax lanceolatus*). Later, in 1834, Gabriel Costa was the first zoologist to consider amphioxus a close vertebrate relative, and renamed it to *Branchiostoma lubricum*. Two years later, William Yarrell described the presence of a notochord and referred to it as amphioxus (i.e. *Amphioxus lanceolatus*) for the first time. In addition to the notochord, which is present in both the embryo and the adult and extends rostral to the cerebral vesicle, amphioxus presents a large number of anatomical features that are shared with vertebrates. Some examples are the dorsal hollow neural tube, the ventral gut, the pharynx perforated with gill slits, segmented muscles and gonads, post-anal tail, pronephric kidney and homologues of the thyroid gland and adenohypophysis (the endostyle and pre-oral pit, respectively). However, they lack several vertebrate-specific characters, such as migratory neural crest cells and placodes, an endoskeleton and a morphologically segmented hindbrain [2]. Phylogenetically, amphioxus species are the living representatives of cephalochordates, the earliest diverging evolutionary lineage within chordates, which also includes urochordates and vertebrates [3]. These two characteristics, a bodyplan conserved with vertebrates and their phylogenetic position, led researchers to use amphioxus as a model organism to study the origin and evolution of vertebrates.

1.2) Distribution, ecology and life cycle

Cephalochordates are benthic filter-feeding marine animals distributed all around the world in temperate and tropical seas that live burrowed in coarse, sandy sediments mostly in shallow waters close to the seashore. They represent a small phylum comprising only around 35 species [4] distributed into three genera, *Branchiostoma* and *Epigonichthys,* which diverged about 35 million years ago (MYA), and *Asymmetron* [5], which diverged from the *Branchiostoma*-*Epigonichthys* clade around 42 MYA [6]. They have separate sexes (gonochoric animals) and reproduce through external fertilization after releasing the gametes into the water column at

sunset during their breeding season, which normally spans from May to July, depending on the latitude.

Kowalevsky was the first biologist who described the embryonic development of the European amphioxus, *B. lanceolatum* [7], which has been reported at different locations in the Mediterranean Sea and the Atlantic coasts of Europe and North Africa [4,8]. While its early development, from fertilization to the gastrula stage, resembles that of other invertebrate deuterostomes, starting at late gastrulation and continuing through neurula stages, its development shares many features with that of vertebrates, particularly with regards to neurulation. The transparent embryos develop rapidly, becoming planktonic larvae after 3 days. These larvae are highly asymmetric since the mouth forms on the left side, the gill slits on the right-ventral side and the right series of somites are located half a segment more posterior with respect to the left series. During metamorphosis, which occurs 1 to 3 months post-fertilization in *B. lanceolatum*, the body is completely reorganized giving rise to a nearly symmetric juvenile. The lifespan of *B. lanceolatum* has been estimated to be around 8 years, compared to 2 to 3 years in *B. floridae* [9,10].

1.3) *Branchiostoma* genomes

Using Feulgen Densitometry, the *B. lanceolatum* genome size was first calculated as 17% of the human genome size (around 550 Mega-base pairs (Mbp)) [11]. This genome size has been confirmed, with slight differences, in distinct *Branchiostoma* species after sequencing of their entire genomes. *B. floridae* has a genome size of 520 Mbp and *B. belcheri* has a smaller genome of 426 Mbp [12,13]. In line with these values, the *B. lanceolatum* assembly reported here has a total of 495.4 Mbp. Amphioxus genomes are compacted into small chromosomes, which are morphologically very similar to each other. However, different diploid chromosome numbers have been found in different amphioxus species. Nogusa, in 1957, first described the diploid karyotype of *B. belcheri,* as possessing 32 chromosomes [14], but more recent studies described a total of 36 chromosomes [15,16]. The diploid chromosome number of *B. floridae* was shown to be 38 [17], which is the same number reported for *B. lanceolatum* [18].

Amphioxus diverged during chordate evolution before the two rounds of whole genome duplication (WGD) (also referred to as 2R) that occurred at the base of vertebrates [19]. Moreover,

it has been shown that, compared to those of vertebrates, the amphioxus genome has experienced fewer gene losses and possesses only a limited number of duplicated genes [2,20-23]. These characteristics make the amphioxus genome one of the best proxies for the ancestral chordate genome in comparative analyses. For example, the amphioxus genome has a set of non-coding elements conserved with vertebrates [24,25] and a high level of conservation of both macrosynteny [12] and microsynteny [26]. Moreover, it has recently been suggested that conserved and divergent features in the 3D structure of amphioxus and vertebrate chromatin may help to explain important evolutionary novelties in the vertebrate lineage [27].

2) DATA COLLECTION AND EXPERIMENTAL PROCEDURES

2.1) Genomic DNA samples of *B. lanceolatum* for genome-wide sequencing

DNA was extracted from a 5.9 cm long male, with full gonads, collected in Argeles-sur-Mer, France, as described in [28]. This specimen was kept for three days at 17°C in filtered seawater with 10µg/ml kanamycin, which was changed daily. Isolation of genomic DNA was performed with the Genomic-tip 500/G and Genomic DNA buffer set (Qiagen), following the manufacturer's instructions. A total amount of 387 µg of genomic DNA was obtained from this single individual. Paired-end and mate-pair Illumina sequencing libraries were prepared at Genoscope and sequenced on HiSeq2000 and HiSeq2500 instruments. Two sets of mate-pair libraries with multiple average insert sizes were built using the TruSeq and Nextera mate-pair construction kits (Illumina) (Supplementary Table 2).

2.2) MethylC-seq library preparation

Gametes were obtained as previously described by heat stimulation [28,29]. Embryos were obtained by *in vitro* fertilization in filtered seawater and grown at 19°C. Staging was done based on Hirakow and Kajita [30,31]; correspondence between developmental stages and hours post-fertilization (hpf) are provided in Supplementary Table 1. For MethylC-seq library preparation, genomic DNA of the 8 hpf, 15hpf, 36hpf and adult hepatic diverticulum samples was extracted as previously described [32] and sonicated to an average size of 200 bp using a Covaris sonicator. Sonicated DNA was then purified and end-repaired, followed by ligation of methylated Illumina TruSeq sequencing adapters and bisulfite conversion using the MethylCode Bisulfite Conversion Kit (Thermo Fisher Scientific). Library amplification was performed with KAPA HiFi HotStart

Uracil+ DNA polymerase (Kapa Biosystems), with 6 cycles of PCR amplification. MethylC-seq libraries were sequenced on an Illumina HiSeq1500 platform, producing an average of 73 million 100-nt single end (SE) reads per sample (Supplementary Dataset 5).

2.3) Reduced representation bisulfite sequencing (RRBS) library preparation.

RRBS libraries were prepared as those for MethyC-seq but with two modifications. Instead of sonication, digestion with the MspI enzyme (New England Biolabs) was performed. 200ng of adult amphioxus tissues (gut, notochord, muscle) in two biological replicates was digested with MspI as per manufacturer's instructions. Furthermore, bisulfite-converted DNA was amplified with KAPA HiFi HotStart Uracil+ DNA polymerase (Kapa Biosystems) for 10 cycles. RRBS libraries were sequenced on an Illumina HiSeq4000 platform, producing an average of 46 million reads per sample in 150-nt paired end (PE) mode.

2.4) RNA sequencing

2.4.1) Amphioxus

An exhaustive collection of 52 RNA-seq datasets was generated using the Illumina technology. These datasets represent a total of 16 embryonic stages and 9 adult organs, totaling 4.2 billion Illumina reads and a volume of 871 Gbp (Fig. 1a, Supplementary Dataset 3).

Different developmental stages, ranging from unfertilized eggs to pre-metamorphic larvae, were directly frozen with liquid nitrogen, and RNA was extracted using RNeasy Mini Kit (Qiagen) for two independent biological replicates. Illumina libraries were constructed from 1 µg using the TruSeq Stranded mRNA kit (Illumina, San Diego, CA) and quantified by qPCR using the KAPA Library Quantification Kit for Illumina Libraries (KapaBiosystems, Wilmington, MA). Library profiles were evaluated with an Agilent 2100 Bioanalyzer (Agilent Technologies). Libraries were subsequently sequenced by Genoscope with an Illumina HiSeq2000 machine, producing an average of 28 million 101-nt paired-end strand-specific reads. After Illumina sequencing, Genoscope applied an additional quality control to the reads that passed the Illumina quality filters. First, low-quality nucleotides ($Q < 20$) were discarded from both ends of the reads. Next, Illumina sequencing adapters and primer sequences were removed using the FastX package (http://www.genoscope.cns.fr/fastxtend/), and reads shorter than 30 nt after trimming were

discarded. Finally, read pairs that mapped to the phage phiX genome (GenBank: NC_001422.1) using SOAP2 [33] were discarded. In addition to the developmental time course, two additional replicates of embryos at 32-cell, 8hpf, 15hpf and 36hpf stages were sequenced at higher depth at the CRG Genomics facility on an Illumina HiSeq2500 machine, generating between 84 and 116 million 125-nt paired-end strand-specific reads.

Adult specimens cultured as described in section 2.1 were used to dissect different tissues. RNA from hepatic diverticulum, gill bars and gut was extracted using RNeasy Plus Mini Kit (Qiagen), neural tube with RNeasy Plus Micro Kit (Qiagen) and muscle, epidermis, male gonads, female gonads and cirri with the TRIzol® Reagent (Thermo Fisher Scientific). Illumina libraries were constructed and sequenced at the CRG Genomics facility on an Illumina HiSeq2500 machine, yielding an average of 135 million 125-nt paired-end strand-specific reads. Moreover, additional replicates of neural tube, muscle, gill bars, gut, hepatic diverticulum and male gonads were sequenced on a HiSeq2000 producing an average of 177 million 76-nt paired-ended non-strand-specific reads.

2.4.2) Zebrafish

Breeding zebrafish (*Danio rerio*) were maintained in standard conditions, at 28°C on a 14 hour light/10 hour dark cycle. A single cross with multiple male and female individuals was set to obtain a large clutch of fertilized eggs, which were cultured at 28°C. Embryos at different developmental time points, from 2hpf to 72hpf, were fixed in RNAlater® (Thermo Fisher Scientific), and RNA was extracted using RNeasy Mini Kit (Qiagen). Illumina libraries were constructed and sequenced at the CRG Genomics facility on an Illumina HiSeq2500 machine, yielding an average of 74 million 125-nt paired-end strand-specific reads. Unfertilized eggs and adult tissue data were obtained from published studies downloaded at the Short Read Archive (SRA) (Supplementary Dataset 3).

2.4.3) Medaka

Medaka (*Oryzias latipes*) wild-type strain *Cab* was maintained and embryos were staged as previously reported [34]. For total RNA extraction, collected embryos were suspended in TRIzol reagent (Intron Biotechnology) with chloroform and subsequently treated with TURBO DNA-

free (Ambion). Three biological replicates were used for RNA-seq analyses. The libraries were sequenced at BGI in an Illumina HiSeq4000, producing an average of 40 million 49-nt single-end reads for each sample.

2.4.4) Human, mouse, chicken and frog

Samples from different embryo stages and/or adult tissues from human, mouse, chicken and frog (*Xenopus tropicalis*) were obtained from published studies at the SRA (Supplementary Dataset 3).

2.5) Cap Analysis of Gene Expression followed by high throughput sequencing (CAGE-seq) samples

RNA from 32-cell, 8hpf and 15hpf embryo stages and female gonads, muscle, neural tube and hepatic diverticulum from adult individuals was obtained as described above. CAGE-seq libraries was performed on these samples using the nAnT-iCAGE (non-amplifying non-tagging Illumina CAGE) protocol [35], with 7 µg of RNA per sample. Following library preparation, samples were sequenced in a single multiplex lane on a HiSeq2500, generating an average of 15 million 50-nt single-end reads (Supplementary Dataset 1).

Mouse CAGE-seq data was obtained from FANTOM5 [36]. The following samples were used in this analysis: whole body (E11) and heart, liver, thymus and uterus (E14). These data were obtained as BED coordinates from the FANTOM data repository, through CAGEr [37]. Zebrafish CAGE-seq data was obtained from [38], comprising 12 stages of a developmental time course from unfertilized egg to Prim20 stage. This dataset was obtained through the *ZebrafishDevelopmentalCAGE* R package available at http://promshift.genereg.net/CAGEr/.

2.6) Assay for Transposase-Accessible Chromatin sequencing (ATAC-seq) samples

2.6.1) Amphioxus embryos

Embryos were grown at 19°C until 8hpf, 15hpf, 36hpf or 60hpf and 100, 30, 13 and 25 embryos were transferred in a 1.5ml tube, respectively, in duplicates. After a one-minute centrifugation at 13,000 rpm, seawater was carefully removed. 50 µl of cold lysis buffer (10 mM Tris-HCl, pH 7.4, 10 mM NaCl, 3 mM $MgCl_2$, 0.1% Igepal) were added and cells were lysed by gentle

pipetting. While 25 µl of the lysate was centrifuged at 500 g for 10 minutes at 4°C, the other 25 µl were used to count nuclei after DNA labeling with DAPI (between 20,000 to 75,000 nuclei were used per transposition reaction). The supernatant was removed, the nuclei resuspended in the reaction mix (25 µl 2x TD buffer (Illumina), 2.5 µL Tn5 transposase (Illumina), 22.5 µL nuclease free H_2O) and incubated at 37°C for 30 minutes. Following transposition, 3 µl of 3M AcoNa (pH5.3) were added to the reaction to adjust the pH, and the DNA was purified using the MinElute PCR purification Kit (Qiagen), following the manufacturer's instructions. The transposed DNA was eluted in 10 µL elution buffer preheated at 37°C. To amplify the library, the following components were combined: 10 µL of transposed DNA, 10 µL nuclease free H_2O, 2.5 µL Nextera PCR primer 1 [39] (25 µM), 2.5 µL Nextera PCR primer 2 [39] (25 µM) and 25 µL NEBNext® high-fidelity 2x PCR master mix (NEB). We used the following conditions for PCR amplification: 72°C for 5 minutes, 98°C for 30 seconds, followed by 13 cycles at 98°C for 10 seconds, 63°C for 30 seconds and 72°C for 1 minute. Following PCR amplification, 3 µl of 3M AcoNa (pH5.3) were added to the reaction to adjust the pH, and the library was purified using the MinElute PCR purification Kit (Qiagen), following the manufacturer's instructions using 20 µL of elution buffer preheated at 37°C. Sequencing was performed at the BGI and CRG, producing an average of 60 million 50-nt paired-end reads per sample (Supplementary Dataset 2).

2.6.2) Adult amphioxus tissues (hepatic diverticulum)

Unripe adults were maintained in filtered seawater for one week at 19°C in the dark with one water change per day. For each replicate, a complete hepatic caecum/diverticulum was dissected in filtered seawater and transferred into a 1.5 ml tube containing 300 µl of cold lysis buffer with a high detergent concentration (10 mM Tris-HCl, pH 7.4, 10 mM NaCl, 3 mM $MgCl_2$, 0.5% Igepal). To efficiently lyse the cells, the hepatic diverticulum was cut into small pieces with sterile scissors in the lysis buffer and tissue homogenization was performed with a sterile Eppendorf micro-pestle followed by gentle pipetting. The subsequent steps were performed as for the embryonic samples using 25µl of the obtained lysate (75,000 nuclei). Sequencing was performed at the CRG, producing an average of 90 million 50-nt paired-end reads per sample. Attempts were also made to generate ATAC-seq for neural tube and muscle tissues, but the reads obtained did not allow reliable detection of open chromatin regions.

2.6.3) Zebrafish and medaka embryos

ATAC-seq assays were performed following the original protocol [39] with some specific modifications for fish species, as previously described [40]. Briefly, two independent batches of 3 to 50 wild type embryos, depending on the developmental stage, and corresponding in all cases to ~75,000 individual cells, were dechorionated, deyolked and lysed. Samples were then treated with Tn5 transposase and the resulting libraries were amplified by PCR using standard ATAC-seq primers [39]. ATAC-seq libraries were sequenced on an Illumina HiSeq4000 machine, producing an average of 70 million 50-nt or 75-nt paired-end reads per sample (Supplementary Dataset 2).

2.6.4) Transgenesis in zebrafish

Enhancer reporter assays in zebrafish embryos were performed as previously described [41]. Selected ATAC-seq-positive peak sequences were amplified by PCR from *B. lanceolatum* genomic DNA with specific primers (Supplementary Table 8). PCR amplicons were first subcloned in a PCR8/GW/TOPO vector and subsequently shuttled using Gateway technology (Life Technologies) into an enhancer detection vector composed of a *gata2* minimal promoter, an enhanced GFP reporter gene and a strong midbrain enhancer (z48), which was monitored as an internal control for transgenesis [42]. Transgenic embryos were generated using the Tol2 transposon/transposase method [43], with minor modifications. In brief, one-cell embryos were injected with a 2 nl volume containing 25 ng/µl of transposase mRNA, 20 ng/µl of purified constructs and 0.05% of phenol red. Three or more independent stable transgenic lines were generated for each construct as reported in Supplementary Table 8. Overall, 10/11 tested APREs drove specific (6) or general (4) GFP expression in transgenic zebrafish assays (Fig. 1e and Extended Data Fig. 2h).

2.6.5) Transgenesis in amphioxus

Adults of *B. lanceolatum* of both genders were collected in Banyuls-sur-mer, France, prior to the summer breeding season and raised in the laboratory until spawning. The spawning of amphioxus male and females was induced by shifting of the temperature as previously described [29]. Selected APRE sequences were amplified by PCR from amphioxus genomic DNA and

subcloned into a transgenic detection vector carrying a minimal *Branchiostoma* actin promoter [44], a GFP reporter gene, and piggyBac transposon terminal repeats. Transgenic embryos were generated by the piggyBac transposon/transposase method [45]. For microinjection of amphioxus eggs, a mixture of DNA construct (200 ng/µl) with piggyBac transposase mRNA (100 ng/µl) in 15% glycerol was used. Transgenic embryos were allowed to develop until neurula or larval stage, fixed in 4% PFA overnight at 4°C, mounted in Vectashield with DAPI (Vector Laboratories), and analyzed by Leica SP5 confocal microscope. The three tested elements showed positive GFP expression. Hox-1655 distal element was active in the neural plate at the mid-neurula stage, as previously reported in zebrafish [27], Foxa-251 was limited to the endoderm at the mid-neurula stage, and Foxc-3067 was active in the somites of early larvae.

2.7) Chromatin immunoprecipitation sequencing (ChIP-seq)

We also ChIP-seq to determine the locations of specific histone modifications associated with distinct functional chromatin states for three developmental stages, focusing on H3K4me3 (active promoters), H3K27ac (active promoters and enhancers), and H3K27me3 (polycomb-repressed regulatory regions, typically associated with developmentally regulated genes). Gametes were obtained from ripe amphioxus adults as previously described by heat stimulation [28,29]. Fertilization was carried out in Petri dishes with filtered seawater and embryos were cultured at 19°C until 8hpf, 15hpf, 36hpf or 60hpf. Batches of embryos from six dishes were transferred into a 2 ml tube (6,000-12,000 embryos per tube). After a quick centrifugation at 9,000 rpm, seawater was removed and replaced by 1.5 ml of fixation solution (1.85% formaldehyde in MOPS-EGTA buffer: 0.1M MOPS pH7.5, 2mM $MgSO_4$, 1 mM EGTA, 0.5M NaCl). After 15 minutes at room temperature, 155 µl of 10% glycine was added and the embryos were allowed to settle in the tube. The solution was then gently removed and the embryos were washed five times at 4°C with cold NaPBS (0.9% NaCl, 20 mM sodium phosphate buffer at pH7.4). After a quick centrifugation at 13,000 rpm, the supernatant was removed and the fixed embryos were frozen in liquid nitrogen and subsequently stored at -80°C.

Chromatin immunoprecipitation was subsequently performed as previously described for other species [46]. Briefly, embryos were homogenized and lysed to release the chromatin, which was subsequently sonicated in a Bioruptor (Diagenode). The sonicated product was incubated first

with the specific antibody (H3K4me3: ab8580, H3K27ac: ab4729, HeK27me3: ab6002, from Abcam) diluted 1:200 and subsequently with Dynabeads (Thermofisher). After washing, pulled-down chromatin was eluted from the beads and de-crosslinked. Illumina libraries were prepared following the manufacturer's instructions and ChIP-seq libraries were sequenced at the BGI using HiSeq2500, producing an average of 30 million 50-nt single-end reads (Supplementary Dataset 4). Reads were mapped against the amphioxus reference genome using Bowtie [47], and peaks were called using the MACS2 software [48] with default parameters.

2.8) Circular chromosome conformation capture followed by sequencing (4C-seq)

For each zebrafish biological replicate, 500 embryos at 24hpf of the Tübingen strain were dechorionated using pronase and deyolked in 1 ml of Ginzburg Fish Ringers (55 mM NaCl, 1.8 mM KCl and 1.25 mM $NaHCO_3$). They were then fixed in 2% formaldehyde in 1x PBS for 15 min at room temperature. For amphioxus biological replicates, embryos (~8,000 at 8hpf and ~4,000 at 15hpf) were concentrated by centrifugation at low speed in 2-ml microtubes. They were fixed for 15 min at room temperature in 1.5 ml of MOPS-EGTA buffer (0.1 M MOPS pH 7.5, 2 mM $MgSO_4$, 1 mM EGTA and 0.5 M NaCl) containing 1.85% formaldehyde. For mouse, 10 E9.5 embryos were treated with Collagenase A (Roche, 10103578001) for 15 minutes at 37º degrees prior to fixation in 2% formaldehyde in 1x PBS during 10 min at room temperature. From here on, the protocols for the three species coincide. 155 µl of 10% glycine was added to stop fixation, followed by five washes with PBS (NaPBS in the case of amphioxus) at 4 °C. Pellets were frozen in liquid nitrogen and kept at −80 °C. Isolated cells were lysed (lysis buffer: 10 mM Tris-HCl pH 8, 10 mM NaCl, 0.3% Igepal CA-630 (Sigma-Aldrich, I8896) and 1x protease inhibitor cocktail (Complete, Roche, 11697498001)), and the DNA was digested with DpnII (New England BioLabs, R0543M) and Csp6I (Fermentas, Thermo Scientific, FD0214) as primary and secondary enzymes, respectively. T4 DNA ligase (Promega, M1804) was used for both ligation steps. Specific primers were designed around the putative transcriptional start sites of the genes with Primer3 v0.4.0. Illumina adaptors were included in the primer sequences, and eight PCRs were performed with the Expand Long Template PCR System (Roche, 11759060001) and pooled. Two libraries from different biological replicates were generated for each 4C-seq experiment (for each viewpoint and sample). These libraries were purified with a High Pure PCR Product Purification kit (Roche, 11732668001) and they were sent for deep

sequencing. An average of 4.9 million 49-nt single-end reads were obtained for each sample. Information about the 4C-seq data and mapping statistics is provided in Supplementary Dataset 13.

4C-seq data were analyzed with the following steps. First, raw sequencing data were demultiplexed and aligned against the Bl7nemr1, danRer10 and mm10 assemblies for amphioxus, zebrafish and mouse, respectively. Reads located in fragments flanked by two restriction sites of the same enzyme, in fragments smaller than 40 bp or within a window of 10 kb around the viewpoint were filtered out. Mapped reads were then converted to reads per first enzyme fragment ends and smoothened using a 51-fragment mean running window algorithm. We then used peakC [49] to identify significant interaction peaks of each gene promoter used as a viewpoint and defined the extension of each regulatory landscape. We further filtered out interactions happening in regions that were poorly covered. Briefly, for each fragment we calculate a local coverage score considering the 25 upstream and 25 downstream restriction fragments. The score is the sum of the fragments covered by at least one read. The local coverage score for a fragment to be considered an interaction should be in the top 25% of the experiment. We also required interactions to appear in regions where the isotonic regression calculated by peakC was above an empirical threshold to ensure a sufficient signal to noise ratio. This empirical threshold was set to 2.5% of the maximum value of the isotonic for each experiment. All regulatory landscape calls and the count of putative *cis*-regulatory elements (APREs) within for each ATAC-seq sample and species are provided in Supplementary Table 12.

3) EUROPEAN AMPHIOXUS REFERENCE GENOME

3.1) Genome assembly and haplotype merge

First, we estimated genome size and polymorphism by examining the k-mer frequency distribution of all 25-mers using Jellyfish for the available paired-end data [50]. This 25-mer spectrum revealed two distinct peaks that are typical of highly polymorphic genomes (Extended Data Fig. 1a,b). Genome size (G) was estimated at 509 Mbp, calculated as the total sequenced base pairs (T_b) divided by the adjusted k-mer coverage (C_k) following the formula:

$$G = \frac{T_b}{C_k} \times \frac{L - k + 1}{L}$$

where L is the read length and k the k-mer size. This value is consistent with previous experimental measures indicating a genome size of 550 Mbp [11].

To assemble a reference *B. lanceolatum* genome, reads were trimmed based on quality using *fastq-trimmer* from the *fastx-toolkit* (http://hannonlab.cshl.edu/fastx_toolkit/) bundled in the Genoscope in-house sequence production pipeline. Paired-end reads were corrected for errors using Musket (v1.0.6) with a k-mer of 21 [51]. Overlapping reads of the 180-bp short insert library were then merged using Flash (v1.2.1) [52]. De Brujin graph assembly was carried out using SOAPdenovo2 (v2.04) with a k-mer of 71 to build contigs [53]. We obtained a very fragmented collection of contigs (N50: 410 bp), totaling 1.21 Gbp, which represents about twice the estimated genome size. Next, to build scaffolds, we employed SOAPdenovo2 with a k-mer of 35 for read mapping. Mate-pair libraries were evaluated based on insert-size distribution and read duplication rates. Because of the limited complexity of the first mate-pair set (Truseq, Illumina), a second set of different insert size libraries was generated using the Nextera kit (Illumina), which uses a transposase instead of mechanical shearing and yields higher output from moderate amount of input DNA (Supplementary Table 2), which greatly improved scaffolding (N50: 718 kbp, total length: 1,019 Mbp). To fill the residual 29% of Gaps (Bl71nemr in Supplementary Table 13), we used Gapcloser (v1.12) from SOAPdenovo2 [53], employing both pairs-end libraries as input and an overlap length of 31, reducing gap fraction to 3% (Bl71nec).

Finally, a haploid version of the assembly was reconstructed using the Haplomerger pipeline (release 20111230), which generates a reciprocal alignment for all the scaffolds of the diploid assembly using Lastz and generates a graph representing allelic relationships for these alignments from which the best path is selected [54]. In a first step, Lastz alignments were used to detect and filter out tandem mis-assemblies. Then, we applied the main haplotype reconciliation tool and removed residual haplotypes that had escaped the primary detection cut-offs (only alignments >5,000 bp are processed) by realigning small residual scaffolds to the haplomerged assembly, as well as removing those with significant similarity to another existing locus. In the

output, scaffolds are renamed depending on whether they derived from merged scaffolds from the original assembly (starting with 'Sc') or whether they correspond to scaffolds from the original assembly, either unchanged ('xfSc') or trimmed ('spSc'). The reconciled assembly (Bl71nemr) closely matched the estimated genome size with a size of 495.4 Mbp, a N50 of 1,296 kbp and 4% of gaps. This assembly included 91.9% of a set of 458 core eukaryotic genes as estimated by CEGMA [55].

3.2) Gene annotation

To take advantage of the extensive RNA-seq dataset we have generated, we followed two distinct approaches to build a collection of transcripts and genes: (i) *ab initio* gene predictions with transcriptomics and similarity (EVidence Modeller, EVM) and (ii) Cufflinks-based transcriptomic mapping and transcript assembly, combined with ORF detection in assembled transcripts using TransDecoder.

3.2.1) EVM-based annotation

First, AUGUSTUS [56] was trained with the gene boundaries derived from the conserved CEGMA set, and 42,226 *ab initio* genes were subsequently predicted using this model across the Bl71nemr assembly. Conserved protein domains were detected by aligning human (Ensembl release 71, 21,432 aligned proteins) and *B. floridae* proteins (JGI models, 20,582 aligned proteins) using Exonerate (v2.2.0) [57]. *De novo* assembly of the bulk of RNA-seq data was performed using Trinity [58], enabling built-in *in silico* normalization to reduce computational burden. The assembled transcripts were aligned and assembled using the PASA pipeline, which grouped the 3,070,821 Trinity transcripts in 488,423 aligned loci [59]. We used EVM [60] to combine *ab initio* gene predictions (weight = 1), protein similarity (weight = 1) and PASA transcript alignments (weight = 5), yielding 33,120 protein-coding genes. To add UTRs and alternative spliced transcripts, we refined the EVM models by incorporating PASA transcript assembly running two consecutive rounds of annotation update, which finally resulted in 84,124 transcripts distributed among 32,360 genes.

3.2.2) Cufflinks-based annotation

Each strand-specific RNA-seq sample (Supplementary Dataset 3) was mapped against the Bl71nemr assembly using Tophat2 [61], and gene models were built using Cufflinks [62]. Each transcriptome-based annotation was then merged using Cuffmerge [63] to produce a single collection of transcripts. Next, this annotation was scanned for protein-coding regions using TransDecoder (v2.0.1) [64], employing blastp similarity against Uniprot sequences (with -max_target_seqs 1 -outfmt 6 -evalue 1e-5 parameters) and pfam-A profiles with hmmscan, as integrated in the TransDecoder pipeline. For each transcript, only the longest predicted ORF was maintained, and start and stop codon lines were inserted in the final GTF, when possible. The final Cufflinks-based annotation included 87,744 transcribed loci and 161,979 transcripts. 28,480 loci were considered protein-coding, and 59,264 were classified as potential non-coding RNAs (lncRNA, eRNA, etc).

3.2.3) Reconciliation of both annotations and additional filtering of gene models

Since Cufflinks affords a better representation of transcript diversity, transcript limits and transcription start site (TSS) positions, and EVM provides more robustly predicted protein-coding sequences, we unified both annotations into a single reconciled final annotation. For this purpose, we intersected exons from both sets of gene models in a pairwise manner, and assigned both transcripts to a single gene model with a unified identifier (e.g. BL00001) when an overlap greater than 25% existed for any of the two transcripts. To keep track of the source of origin, a suffix indicative of the dataset (e.g. BL00001_evm0 or BL00001_cuff0) was added to generate the final transcript identifier. When two transcripts were assigned to a given new identifier, all transcripts from the original gene model were assigned to the new gene model.

Additional filters were then applied to the set of clustered transcripts. First, only a representative transcript was selected when more than one transcript had fully redundant inferred mRNA sequences; priority was given to the EVM over the Cufflinks transcripts. Second, we assessed more closely the cases in which the final merged gene model was composed of transcripts from one gene model from one source (i.e. EVM or Cufflinks; thereafter the "long" model) and from at least two different gene models from the other source (thereafter the "short" models). These cases may be due to, for example, the long model being a chimera of two different genes artificially connected during transcriptome assembly. The short models, on the other hand, could

be incomplete gene models that only the pipeline providing the long gene model was able to assemble properly. Therefore, to identify such instances, for each merged gene model, we intersected the coordinates of the original gene models and identified those cases in which a gene model with three or more introns from one source (long model) overlapped two or more gene models with three or more introns from the other source (short models), all in the same orientation. To better evaluate the different scenarios mentioned above, all predicted proteins of the EVM and Cufflinks models were then independently blasted against the human reference proteome (Ensembl release 71), and the regions with detected similarity against each human protein were scored. In addition, predicted proteins from all EVM and all Cufflinks models were blasted against each other. The following situations were considered:

- The short models were discarded if they had blast similarity with the same human protein with an overlap of less than 20 amino acids in the correct relative positions (i.e. the upstream model showed similarity with a more N-terminal part of the human protein than the downstream short model). Manual inspection of several of these cases confirmed they corresponded to incomplete gene models.
- The long gene model was discarded if the two (or more) short models had similarity only to proteins from different human genes. Manual inspection confirmed the long models likely corresponded to technical chimeras. In these cases, the short gene models were reassigned independent gene IDs and considered independent gene models in the final annotation.
- If only one or none of the short gene models had similarity with human proteins, then the long gene was discarded if the two (or more) short gene models had reciprocal blast hits. Manual inspection of these cases showed that they usually corresponded to tandem duplicates that were incorrectly assembled (generally by Cufflinks) into a single gene long model due to their sequence similarity. As above, the two gene models were then reassigned two independent gene IDs. If the short models showed no similarity with each other, all models were kept as transcripts of a single model.

In total, 1,012 long models and 494 groups of short models were discarded. Our final reconciled annotation includes 218,070 transcripts (78,166 derived from EVM and 139,904 from Cufflinks) belonging to 90,927 unified loci. 63,638 of these loci have at least one isoform transcribed above 2 transcripts per million (TPM) in one of our RNA-seq dataset, and 33,800 have at least one

annotated intron. 20,569 loci are protein-coding and have similarity with at least another species used for family reconstruction (see Section 4.1), whereas 18,170 loci are predicted as protein-coding but have not being assigned orthology to any gene from any of the other studied species using OMA. High numbers of predicted protein-coding genes without orthology have been described in other amphioxus genomes. Using ESTs, Huang *et al* [13] identified 30,392 putative protein-coding genes in the *B. belcheri* genome, of which only 18,167 had an ortholog in *B. floridae*. Similarly high fractions have also been reported for specific gene families (e.g. tyrosine kinases [22]). Such genes often showed low expression levels (44% had a maximum expression lower than 5 TPMs in our RNA-seq panel, compared to 19% of orthology-supported genes) and their predicted coding sequence is significantly shorter than orthology-supported protein-coding genes (median 175 vs. 311; $p < 2.2 \times 10^{-16}$, Wilcoxon Sum-Rank test). Unless explicitly clarified, all subsequent analyses were limited to the set of 20,569 protein-coding gene models ('orthology-supported' gene set) that have orthologs in at least one of the species used for gene family reconstruction.

3.3) Identification and classification of long non-coding RNAs

To define the long non-coding transcriptome of the amphioxus genome, the full transcriptome annotation was feed into a filtering pipeline (Extended Data Fig. 1g) following [65] with some modifications. First, all transcripts shorter than 300 nt were removed. Second, CPAT software v1.2.1 [66] was used to identify likely protein-coding transcripts among all annotated isoforms. This software was trained with *bona fide* sets of coding and non-coding transcripts from *D. rerio*, using a cutoff of 0.38. All gene models with any transcript with a score above this value were marked as potentially protein-coding and subtracted from the main set. Third, blastx and HMMER searches in the six reading frames were performed versus non-redundant protein and Pfam-A databases, respectively. For the HMMER analysis, transcripts were previously translated using the TransDecoder suite v3.0.1 [64] and a maximum ORF of 150 aa was imposed. All genes with any transcript with a hit with an E-value lower than 10^{-4} were removed to produce the final set of lncRNAs. These lncRNAs were then classified according to their relative position with respect to neighboring protein-coding genes [67] into four categories: (i) intergenic: lncRNA transcripts located in the genomic interval between genes (26,735 loci; 1,284 multiexonic); (ii) intragenic: lncRNA transcripts that are present within a protein-coding locus, in the same strand

(2,530 loci; 66 multiexonic); (iii) antisense: lncRNA transcripts that share more than 50 nucleotides with a protein-coding gene and are placed in opposite strands (1,855 loci; 129 multiexonic); (iv) bidirectional: lncRNA transcripts located in the proximity (<1 kbp) or slightly overlapping (<50 nt) a protein-coding gene, in the antisense strand (365 loci; 33 multiexonic). All lncRNA loci coordinates and information are provided in Supplementary Dataset 6.

In order to assess the sequence conservation of the catalogue of lncRNAs, a series of nucleotide-nucleotide BLAST (blastn v2.2.29+) were performed with default parameters against five different genomes: *H. sapiens* (hg38), *M. musculus* (mm10), *O. latipes* (oryLat2), *D. rerio* (danRer11) and *S. purpuratus* (strPur2). Sequence-based comparisons against these species showed no significant conservation for any family, with the exception of one that appears to be a LINE-derived family with sequence similarity to the RTE-1_DR non-LTR retrotransposon from the RTE/BovB family. This is likely a horizontal gene transfer event, according to previous analysis of this LINE family [68].

3.4) Gene ontology term assignment and enrichment analyses

We scanned protein-coding gene models for PFAM domains using the Pfamscan.pl tool relying on hmmer HMM profile similarity search and a set of precompiled profiles [69]. We identified a total of 5,118 distinct PFAM domains assigned to 23,906 gene models. In parallel, InterPro domains were identified and assigned to gene models using the InterProScan tool [70]. Finally, the Gene Ontology (GO) annotation of human was mapped by blasting amphioxus gene models against the human proteins (e-value < $1e^{-6}$) and transferring the corresponding GO terms accordingly. A total of 12,510 distinct GO terms were assigned to 21,798 protein-coding gene models. GO enrichments for different gene sets throughout the study were calculated using topGO (R package version 2.29.0) [71], and plotted as histograms using custom scripts. For zebrafish GO analyses, topGO was also used with gene-to-GO assignments downloaded from Ensembl Biomart (release 80).

3.5) Repeat masking and TEs

A custom library of repeats was built using RepeatScout and RepeatModeler, and repetitive regions were masked using RepeatMasker (http://www.repeatmasker.org). The masked regions

represent 32% of the Bl71nemr assembly, and revealed a very diverse repertoire of DNA repeated elements (Supplementary Table 14). A landscape of repeats according to their age of origin (estimated as the K2P distance to the sequence consensus in the repeat library) revealed no recent invasions (Extended Data Fig. 1d). DNA transposable elements are the most common class among *B. lanceolatum* repeats (10.86% of genome), followed by LINE retro-elements (4.89%, including notably CR1 and RTE elements), and SINEs (1.86%). LTR retrotransposons, which are usually considered the longest and include several ORFs, are quite rare in the *B. lanceolatum* genome (0.43%), although a few recent Gypsy elements were detected in the Bl71nemr assembly. However, it should be noted that these low numbers are likely due to the gap filling step using paired-end reads during genome assembly. Overall, the abundance, diversity and apparent lack of any recent expansion of TE families in *B. lanceolatum* are consistent with the scenarios described for other amphioxus species [72], and corroborate the fact that amphioxus genomes do not seem to be particularly prone to major evolutionary changes mediated by TEs.

3.6) Genome-wide alignment of *Branchiostoma* genomes and PhastCons score calculation

Genome sequences from *B. floridae* and *B. belcheri* were aligned to the *B. lanceolatum* assembly after repeat masking using lastz (--inner=2000 --ydrop=3400 --gappedthresh=6000 --hspthresh=2200). A set of tools from the Kent utilities were used to subsequently process the alignments following UCSC guidelines [73]. Pairwise Axt alignments were converted to chain alignments using axtChain, and then sorted and filtered with chainMergeSort and chainPreNet. Alignment chains were converted to alignment 'nets' using chainNet. We combined the pairwise alignments in a single multiple alignment in *B. lanceolatum* coordinates using multiz-tba. Conservation scores and highly conserved regions were computed starting from the multi-species alignments using PhastCons [74], after calculating the background model from the longest scaffold using phyloFit [75].

4) RESOURCES FOR COMPARATIVE GENOMICS

4.1) Gene family reconstruction and orthology assignment

To infer orthology and paralogy (including ohnology) relationships between amphioxus and different vertebrate and invertebrate species, we reconstructed gene families using OMA (v1.0.5)

[76], which relies on Smith-Waterman alignment, processing of evolutionary distance and reconstruction of Hierarchical Orthologous Groups (HOGs) according to pre-defined phylogenetic relationships between species [77]. We applied this algorithm with default parameters to 11 proteomes derived from genome annotations (Supplementary Table 4), yielding 23,799 gene families. In cases where multiple transcript isoforms per locus were present in the annotation, we selected the longest protein isoform for gene family reconstruction. Finally, we used Ensembl paralogy information for human and mouse (release 80) to further merge gene families that contained members that were considered paralogs at the level of "Vertebrata" or "Euteleostomi". In such cases, the family with the highest numerical identifier was integrated into the family with the lowest identifier (original gene family identifiers are displayed in column "Fam_ID_Orig" in Supplementary Dataset 7). In total, our final orthology set consisted of 21,576 gene families.

4.2) Identification of ohnologs

To identify putative vertebrate ohnology groups derived from the two rounds of WGD, we first determined how many families have a consistent pattern of duplicates with two to four copies in at least three out of five studied vertebrate species (excluding zebrafish and medaka as they experienced an extra round of WGD). This yielded 1,781 candidate gene families. For each of these families, we performed a phylogenetic analysis with RAxML (v8.2.1) assuming a LG+Gamma model [78], and examined the topology of the corresponding trees using the ETE 3 python library [79]. In particular, for all duplicates, we required that their common ancestor matched the most recent ancestor of all vertebrate species in the tree, and not a more recent one as would be expected in the case of lineage specific duplication. We also required that all amphioxus gene copies (if more than one) were monophyletic in the tree. After these steps, we obtained 1,509 families, which were used for subsequent analyses (Supplementary Dataset 9). For comparison, we also generated a list of high confidence single-copy orthologs, from 2,511 gene families that have a single member in amphioxus, zebrafish, mouse, frog and medaka (Supplementary Dataset 9).

4.3) Definition of *trans-dev* and housekeeping genes

Orthologous clusters of *trans-dev* genes (i.e. genes implicated in transcriptional regulation or development [80]) were defined based on the GO annotations for the mouse orthologs. We downloaded GO annotations for mouse from Ensembl Biomart (release 80) and defined *trans-dev* genes as those with: (i) GO:0009790 (embryo development) and/or GO:0030154 (cell differentiation) Biological Process annotations; and (ii) GO:0043565 (sequence-specific DNA binding), GO:0007267 (cell-cell signaling) and/or GO:0008380 (RNA splicing) Molecular Function annotations. In contrast, mouse housekeeping genes were defined as those with a 1-to-1 ortholog with yeast (*Saccharomyces cerevisiae*) based on Ensembl Biomart, and that did not have GO:0009790 (embryo development) or GO:0030154 (cell differentiation) Biological Process annotations. After assigning these categories to the mouse genes, the annotations were transferred to all genes from the same orthologous group, including homologs from other species and their paralogs. This resulted in a total of 654/817, 809/916, 680/805 and 362/865 *trans-dev*/housekeeping genes in mouse, zebrafish, medaka and amphioxus, respectively (Supplementary Dataset 9).

4.4) Orthologous intron positions

Orthologous intron positions were determined for the orthologous gene clusters described in 4.1 for *B. lanceolatum*, zebrafish and mouse. Gene clusters with more than 5 genes from at least two species and/or with more than 10 genes from at least one species were discarded before further analysis. For all the members in each orthologous gene cluster, pairwise protein alignments between species were performed using MAFFT with default parameters [81]. Intron positions from the gene annotation of each species were used to define intron borders in their corresponding protein sequence and mapped to the resulting pairwise protein alignment. An intron position was considered to be orthologous between a pair of species if the introns had the same phase and if they were within three amino acids from each other in the protein alignment.

4.5) Syntenic analysis and Genomicus browser

Gene families containing annotated genes from nine reference species were downloaded from Ensembl release 83: *Monodelphis domestica, Homo sapiens, Mus musculus, Gallus gallus, Lepisosteus oculatus, Danio rerio, Ciona intestinalis, Caenorhabditis elegans, and Drosophila melanogaster*. Protein sequences from *B. lanceolatum* and *B. floridae* were integrated into these

families using a method previously developed for vertebrate and plant species [82,83]. Briefly, we (i) performed blastp comparisons between predicted protein sequences from each amphioxus genome against those from the nine species from Ensembl, and filtered the results with a cut-off value of 10^{-4}; (ii) calculated an average family bitscore between a given family of protein sequences and each amphioxus protein sequence to identify the most likely family it belongs to; (iii) performed a multiple alignment using M-Coffee to recompute a new reconciled gene tree with the species tree using the Treebest pipeline. We thus obtained families containing orthologous and paralogous genes containing two amphioxus, six vertebrates and three other non-vertebrate outgroups. The 7,255 trees contain 27,557 *B. lanceolatum* genes. The homology data and genome coordinates for all studied genomes were uploaded in a Genomicus database and online server [84] available at http://genomicus.biologie.ens.fr/genomicus-amphioxus.

To illustrate how amphioxus can be used to analyze the consequences of the two rounds of WGD on genome organization, we built a Quadruple Conserved Synteny (QCS) map of the *B. lanceolatum* scaffold Sc0000001, which contains 769 genes (Extended Data Fig. 1h). Ancestral chordate nodes in the 7,255 trees were identified and, when the amphioxus descendant mapped to this scaffold, the human descendants were recorded together with their chromosome location. The majority (73.3%) of the amphioxus genes possessed human orthologs on at least one of four chromosomes (human chromosomes 1, 5, 9 and 19). Reciprocally, 61.3% of all human orthologs of amphioxus genes mapping to this scaffold were located on any of these four chromosomes. We used an *ad hoc* script to draw the map (top panel of Extended Data Fig. 1h) and examined one locus in detail using the PhyloView display from Genomicus.

5) GENOME-WIDE PATTERNS OF ChIP-seq AND ATAC-seq

5.1) ATAC-seq mapping, peak calling and classification of regulatory elements

ATAC-seq reads were mapped using Bowtie2 [85] with "--very-sensitive -X 2000 -I 0" as additional options. Read pairs that had a fragment length smaller than 120 bp were considered as "nucleosome free" and used for peak calling, which was done in two steps. First, MACS2 [48] was used for low-threshold peak calling using the following parameters: callpeak --nomodel --keep-dup 1 --llocal 10000 --extsize 74 --shift -37 -p 0.07. Next, the IDR framework (idr 2.0.3;

https://github.com/nboley/idr) was used to obtain high confidence peaks based on replicate information.

5.2) Overlap between ChIP-seq and ATAC-seq-derived elements

In order to compare the general profiles of various epigenomic marks around different types of ATAC-seq peaks, the latter were classified into four groups, depending on their relative genomic position: (i) "Promoters" were defined as those peaks overlapping with the region spanning from -1 kbp to +0.5 kbp of any annotated TSS of the 20,569 orthology-supported protein-coding genes; (ii) “Other APREs” corresponded to peaks that did not overlap with the TSS region of any protein-coding gene, conservatively considering genes both with or without orthologs in vertebrates (38,719 gene models); from this group, (ii.a) “proximal" APREs were defined as those peaks at 5 kbp or less from any TSS of genes with orthologs in other species, and (ii.b) “distal" APREs corresponded to peaks located further away than 5 kbp of any TSS of any protein-coding gene. ATAC-seq peaks that were not assigned to any of these conservative categories were not used for analyses in Fig. 1c and Extended Data Fig. 2d. A fifth test group was added, comprising all the previously validated amphioxus enhancers (Supplementary Table 9). For this, we collected information from all 36 previously published experimentally validated enhancers (from 20 articles [24-27,44,86-101]) from three amphioxus species that could be uniquely mapped to our *B. lanceolatum* reference genome. 32 of them (88.8 %) overlapped with ATAC-seq peaks from our dataset (including all seven APREs tested in amphioxus). Finally, two control sets with a similar size distribution to that of our ATAC-seq peaks were defined: a set of random regions from anywhere in the genome and a set of random regions excluding sequences overlapping with ATAC-seq peaks. The total number of mapped reads of H3K4me3, H3K27ac, ATAC-seq and CAGE-seq overlapping with peaks of each group was calculated using Bedtools [102].

To further investigate the nature of our APRE catalog, we analyzed their H3K27ac dynamics during development using ChIP-seq data. Between 36.3 and 43.9% of APREs were active at a given developmental stage based on H3K27ac levels (Extended Data Fig. 2e), and a substantial fraction of these became active/inactive during the time course (Extended Data Fig. 2f). Interestingly, dynamic APREs were enriched for distinct TF binding motifs as determined by

MEME, including pluripotency- and differentiation-associated TFs in early and late active APREs, respectively (Extended Data Fig. 2g). In contrast, APREs that were inactive (based on H3K27ac signal) across the time course were enriched for CTCF motifs, among others, suggesting APREs also encompass other types of regulatory elements.

6) COMPARATIVE TRANSCRIPTOMICS

6.1) Comparison of developmental stages across chordates and phylogenetic period analysis

For comparisons with our amphioxus stage-specific RNA-seq, we generated RNA-seq data from time-courses of developing zebrafish and medaka, and gathered available data for developmental stages of chicken and frog from different sources (Supplementary Dataset 3 and Supplementary Table 3). Gene expression for each species was estimated using Kallisto (v0.42.4) [103] for the full set of available Ensembl transcripts for each species (Supplementary Table 4). Gene-level expression was obtained by summing up TPMs from all transcript isoforms per gene.

To perform gene expression comparisons of embryonic stages between each species pair, we used single-copy orthologs for the specific pair, as inferred by OMA (Supplementary Table 15). We then applied quantile normalization to the gene expression metrics with the *preprocessCore* package from Bioconductor, and examined several distances and correlation metrics to estimate gene expression divergence between species: Euclidean, Manhattan and Jensen-Shannon distances as well as Spearman correlation. We compared the temporal conservation profiles obtained with these different metrics, and verified that all gave qualitatively similar results (Extended Data Fig. 7b), and were resilient to the impact of highly expressed genes. We decided to use Jensen-Shannon metrics (square root of Jensen-Shannon distance [JSD_s] to respect the triangle inequality) for further analyses, due to its capacity to sum up the effect of genes with distinct expression levels. JSD_s was computed as the sum over orthologous genes (*g*) of species 1 (*p*) and species 2 (*q*) with normalized TPMs:

$$JSD_s = \sqrt{\frac{1}{2}\sum_{g=0}^{n_{\mathrm{og}}} p_g \cdot log(\frac{p_g}{\frac{1}{2}(p_g+q_g)}) + \frac{1}{2}\sum_{g=0}^{n_{\mathrm{og}}} q_g \cdot log(\frac{q_g}{\frac{1}{2}(p_g+q_g)})}$$

To obtain an estimate of the robustness of this metric towards gene sampling, we performed a bootstrap resampling of the orthologous gene set (100 iterations of 5,000 orthologs with replacement), we computed the JSD metrics on each replicate, and then estimated the standard deviation of the JSD metrics for the pair of stages considered.

6.2) Transcriptome clustering during embryo development

For this and subsequent gene expression analyses, we used the cRPKM metric (corrected-for-mappability Reads Per Kilobasepair of uniquely mappable positions per Million mapped reads [104]). For this purpose, a reference transcript per protein-coding gene was selected for amphioxus as the transcript with the highest expression support across all samples (Supplementary Dataset 14). For vertebrate species, the reference transcript was obtained from Ensembl BioMart (release 80). Then, the number of uniquely mappable positions for each reference transcript was determined, and cRPKMs for each gene were calculated as previously described [104]. To increase read depth and the robustness of the quantifications, RNAs from several independent experiments for the same stage and/or adult tissue were pooled to estimate gene expression, when possible (Supplementary Dataset 3).

To cluster genes from amphioxus and zebrafish according to their developmental expression dynamics in each species, we used the soft clustering software Mfuzz [105] independently for both amphioxus and zebrafish datasets. To be able to compare the profiles, we selected eight developmental stages from amphioxus and zebrafish that could be matched one-to-one from unfertilized eggs to fully autonomous larvae based on developmental reference points such as fertilization, gastrulation and organogenesis (Supplementary Table 5). Starting with genes in orthologous groups shared by both species as assigned by OMA (10,777 in amphioxus and 13,719 in zebrafish), we first filtered out genes with low variability (coefficient of variation < 1) to 8,244 amphioxus and 10,783 zebrafish genes, before running Mfuzz clustering with default parameters. To choose numbers of clusters, minimum centroid distance was calculated for hypothetical numbers of clusters using the “Dmin” function in Mfuzz, with 28 clusters being the optimum number in both species (Supplementary Fig. 3 and 4). GO enrichment analyses were used to assign categories to each cluster. For this, we used two-sided Fisher's exact tests as implemented by topGO, with the set of all genes present in clusters were used as background.

We calculated the statistical significance of the overlap of orthologous genes between all pairs of clusters from each species, using an upper-tail hypergeometric test. This revealed pairs of clusters with highly significant overlap of orthologous genes (Extended Data Fig. 8a). In most cases, these profiles had very similar temporal dynamics with respect to the equivalent developmental landmarks, despite markedly distinct cell type compositions and differentiation dynamics of the embryos. Next, for those pairs of clusters with significant ortholog overlap ($p < 10^{-10}$, upper-tail hypergeometric test), we classified them as: (i) homochronic, if the two profiles have the exact temporal dynamics or delayed by one stage (e.g. upregulated at the last vs. second to last stage in amphioxus and zebrafish, respectively)($n = 48$); (ii) intermediate, if they had the same temporal dynamics but had two or three stages of delay; (iii) heterochronic, with a different temporal profile or a delay of four or more stages ($n = 35$). Then, for each group, the number of zebrafish clusters with a given enriched GO term (FDR<0.05) was scored as a measure of global GO enrichment of the group. The top enriched terms for homochronic and heterochronic groups were plotted together using Z-score normalization. Interestingly, clusters with significant ortholog overlap that showed similar temporal dynamics in both species were highly enriched for "intracellular" gene functions and components, such as nucleic acid binding and nucleus, whereas the heterochronic clusters were enriched for membrane-related and extracellular functions and components (Extended Data Fig. 8c). To specifically investigate the conservation of temporal regulation of components of key developmental pathways (Extended Data Fig. 8d), we first obtained a list of genes associated with each of the pathways from zebrafish GO annotations (from Ensembl BioMart) (Supplementary Dataset 15). Characteristic genes from a given pathway (e.g. Fgfr or Fgf genes for FGF, Wnt genes for WNT, etc.) were removed from the other pathways when annotated in GO due to pathway crosstalk ("EXCLUDE" in column "Crosstalk"). Then, for pairs of orthologs of pathway components that were present in pairs of Mfuzz clusters with significant ortholog overlap, we scored the fraction of such pairs that are homochronic, intermediate or heterochronic. To statistically compare between pathways, we performed two-sided 3-way Fisher's exact tests and corrected for multiple testing using Bonferroni correction. Overall, genes from the Hedgehog and Hippo pathway more often fell into homochronic cluster pairs with significant ortholog overlap (Extended Data Fig. 8d).

6.3) Weighted Gene Correlation Network Analysis (WGCNA)

To obtain modules of coexpressed genes across developmental stages and adult tissues, we used WGCNA [106]. We selected 17 amphioxus and 27 zebrafish samples, including replicates of adult tissues when possible (Supplementary Table 6). Of 20,569 amphioxus and 20,082 zebrafish genes that had an ortholog in at least one other species used for gene family construction, 16,421 and 18,285 genes, respectively, had enough variance (CV ≥ 1), to be considered for further analysis. WGCNA was run with default parameters (softPower settings 9 in amphioxus and 7 in zebrafish; with unsigned networks), resulting in 25 and 23 modules in amphioxus and zebrafish, respectively. Genes in each cluster were assigned positive or negative correlation status. Each cluster was assigned a tissue affinity and/or functional category based on its overall gene expression and GO enrichment profiles (Supplementary File 1). Next, to assess the statistical significance of homolog overlap between each pair of clusters from each species, we counted the number of overlapping homologous groups (i.e. paralogs within a gene family were not counted multiple times), and performed a hypergeometric test taking as background only the number of homologous groups with members in both amphioxus and zebrafish.

6.4) Neighborhood Analysis of Conserved Co-expression (NACC) analysis

To have a first general assessment of the extent of conservation or divergence in gene expression among chordates at adult stages, we used Neighborhood Analysis of Conserved Co-expression (NACC)[107], a method developed during the ENCODE project to compare heterogeneous, non-matched sample sets of RNA-seq between human and mouse. The NACC value for any given gene, e.g. gene 1 in species A (gene1A) and its ortholog in species B (gene1B) was calculated as follows. First, we obtained the 20 genes with the smallest Euclidean transcriptomic distance to gene1A in species A (neighbor1A$_{1\text{-}20}$), excluding homologs belonging to the same orthology group as gene 1. The average of the distances from gene1A to each neighbor1A$_{1\text{-}20}$ was termed "avA". Next, we retrieved the orthologs of these neighbors in species B (neighbor1AB$_{1\text{-}20}$) and calculated their distances to gene1B, producing an average value "avAB". In the same way, we obtained the 20 closest neighbors for gene1B within species B (neighbor1B$_{1\text{-}20}$) and their orthologs in species A (neighbor1BA$_{1\text{-}20}$) and calculated the distances of the latter set to gene1A, providing the average distances "avB" and "avBA". The NACC value for gene 1 between the two compared species corresponded to NACC = [(avAB-avA)+(avBA-avB)]/2. Finally, random

sets of NACC values were obtained using a similar procedure, but randomizing the orthology connections between the two species.

NACC values were plotted for each species against human for all gene families with a single amphioxus member, one to four human members and one to eight members for the compared vertebrate species. In the case of multi-gene families, when searching for neighbor orthologs, only the family member with the shortest distance to the reference gene was selected in each comparison. Using human as reference, we found significant conservation of vertebrate and amphioxus orthologous gene expression patterns compared to gene sets with randomized orthology relationships (Extended Data Fig. 8b). Use of gene families with only one member across all species gave very similar results. RNA-seq samples used for these analyses are shown in Supplementary Table 16; for human and mouse, we employed a large collection of samples previously compiled by [108].

7) CAGE-SEQ AND PROMOTER ANALYSES

7.1) CAGE-seq mapping and generation of tag clusters

CAGE-seq tags were aligned to the amphioxus genome using Bowtie v1.1.2 [47], with a seed length of 25, allowing up to two mismatches in the seed region and discarding multi-mapping reads (Supplementary Dataset 1). The resulting alignments were processed to remove leading G nucleotides where these did not map to the genome, and tags mapping to each nucleotide were counted and normalized to follow a power-law distribution [109].

CAGE transcription start sites (CTSSs) map initiation at single base-pair resolution and commonly occur in clusters that give rise to functionally equivalent transcripts (Supplementary Fig. 5a). Nearby individual CTSSs were thus combined using the distance-based clustering method in CAGEr [37] to produce tag clusters (TCs), which summarize expression at individual promoters. To reduce sensitivity to outlying CTSSs and improve robustness, the width of each TC was calculated by discarding the first and the last 10% of the CAGE signal. This is referred to as the Inter-quantile (IQ) range [37]. TCs were also assigned a dominant CTSS, which is the individual CTSS with the highest expression level. TCs with combined expression of less than 1 tag per million (TPM) were filtered out. The final set of CAGE-seq TCs is provided as

Supplementary Dataset 16, along with their associated features. All samples except muscle passed the quality control, showing the expected power-law distribution of tag counts and bimodal distribution of cluster widths (Supplementary Fig. 5b-h). The muscle sample was dominated by TCs of width = 1, which are not indicative of biologically meaningful promoters and so was excluded from further analysis.

To assess the accuracy of TSS annotations respect to CAGE-seq data, we performed the following analysis. For genes with a cRPKM ≥ 5 in any of the RNA-seq samples covered also by CAGE-seq data, we plotted the cumulative distribution of the distance between CAGE-seq TCs and the closest annotated TSS (Extended Data Fig. 1b). Only TCs within 1kbp of an annotated TSS were considered for this analysis.

7.2) Expression clustering

TCs were further clustered across cell types to produce comparable promoter regions, referred to as "consensus clusters". The consensus clusters were then grouped by expression patterns using a self-organizing map (SOM) [110], using a 5x5 arrangement. The SOM produced both defined groups and topographic relationships between groups (Supplementary Fig. 6). The topographic relationships of groups in the SOM allowed us to combine similar groups together to produce sets of consensus TCs (conceptually, sets of promoters), which are expressed in similar tissues during the course of development. Based on these relationships, we defined five promoter sets for further analyses (Supplementary Fig. 6): (i) ubiquitous (2_1, 3_1, 2_2, 3_2), (ii) embryo-specific (3_4, 4_3, 4_4), (iii) neural-specific (0_4), (iv) hepatic-specific (0_0) and (v) female gonad-specific (4_0).

7.3) ATAC-seq data and nucleosome positioning

Nucleosome positioning was calculated from aligned ATAC-seq data using NucleoATAC v0.3.2 [111] with default parameters. ATAC-seq data for mouse and zebrafish for, respectively, the limb and whole body embryo at 24hpf were taken from [42] and aligned using Bowtie, with a 25-bp seed region and allowing up to two mismatches. The 15hpf stage, from the present study, was used for amphioxus.

7.4) Feature enrichment and visualization

We investigated the relative presence and enrichment of the following features: TATA box, YY1 motif, GC and AT content, SS and WW dinucleotides, first exons and nucleosome positioning signal. Heatmaps were plotted for visualization by scanning either for exact dinucleotide matches or PWM matches at 80% of the maximum score. PWMs for TATA and YY1 were taken from the JASPAR vertebrate collection. The binary matrix resulting from sequence matches (either PWM-based or exact) was then smoothed and downsized using a binned Gaussian kernel density approach. Nucleosome positioning signal was winsorized to the 99th percentile and smoothed using a Gaussian blur. Promoters were sorted by IQ width, unless otherwise specified. All plots were made using R/Bioconductor with the *heatmaps* package, which is included in Bioconductor 3.5 [112].

At the sequence level, ubiquitous promoters had a unique architecture, not described in any other organism, characterized by a WW (where W is A or T) dinucleotide enrichment preceding the TSS followed by two stretches of SS (C or G) dinucleotide-enriched regions, and with a nucleosome positioned in between (around position +90 from the TSS) (Extended Data Fig. 3a,d). Moreover, the dominant TSS was usually asymmetrically located in the 5'-most region of the promoter (Extended Data Fig. 3a). On the other hand, as in other metazoans, ubiquitous promoters were often broad (i.e. with multiple TSS positions within a stretch of sequence) (Extended Data Fig. 3a), and, as in vertebrates, were enriched in YY1 motifs (particularly the narrowest ones) (Extended Data Fig. 3e,f). In contrast, embryo- and tissue-specific promoters did not share these features (Extended Data Fig. 3b,c), and had similar architectures at the sequence level to those described for other metazoans [36,38,113].

7.5) Bidirectional promoter analysis

Bidirectional promoters were defined as promoters in opposite orientation with a distance of less than 1 kbp between them. The dominant CTSS was used for the exact localization of promoters. Only pairs of promoters associated with two annotated protein-coding genes were used for this analysis, since CAGE-seq can detect antisense transcription at promoters, which are unlikely to be functional. CAGE-seq derived peaks were associated to specific genes if they were within 1 kbp of an annotated model. Pairs of bidirectional promoters displayed a marked inter-promoter

distance periodicity (Extended Data Fig. 4b), with a period consistent with the spacing of zero, one or two nucleosomes, based on NucleoATAC signal [111] (Extended Data Fig. 4c). GO enrichment analysis was performed as described above. To evaluate the relative number of CAGE-supported bidirectional promoters involving two protein-coding genes or one protein-coding gene and non-coding transcript in each species (Extended data Fig. 4f), we defined the latter as those bidirectional promoters associated with an annotated protein-coding gene and either an annotated non-coding gene or no annotated gene at all, which were inferred to produced a non-coding transcript.

7.6) Evolution of ancestral bidirectional promoters

To investigate the putative WGD-assisted disentanglement of bidirectional promoters in vertebrates, we first identified putatively ancestral chordate CAGE-supported bidirectional promoters using three complementary approaches. First, for each pair of protein-coding genes involved in amphioxus or mouse CAGE-supported bidirectional promoters, we matched them to the ancestral microsyntenic pairs reported in [26]. Since this study used *B. floridae* and human, best reciprocal blastP hits between the two amphioxus and the two mammals were used to assign 1-to-1 orthology. Second, for each pair of protein-coding genes involved in amphioxus, zebrafish or mouse CAGE-supported bidirectional promoters, we searched for orthologs in the hemichordate *Saccoglossus kowalevskii* and the sea urchin *Strongylocentrotus purpuratus* in our orthology gene set (Supplementary Dataset 7). If the orthologs of both genes in any of the two species were found in a 5'-to-5' orientation with no intervening genes, it was assumed to be an ancestral bidirectional promoter. Third, we compared the zebrafish and mouse CAGE-supported bidirectional promoters with those of amphioxus. Those involving the same pair of orthologs were conserved ancestral to chordates. Then, for this combined set of bidirectional promoters (i.e. protein-coding gene pairs), we required that all amphioxus, zebrafish and mouse had at least one ortholog for each gene in the pair. Moreover, cases in which amphioxus had more than three orthologs were discarded. This resulted in a list of 372 pairs of putatively ancestral CAGE-supported bidirectional promoters. To infer their evolutionary dynamics (Extended Data Fig. 4e), two approaches were taken: (i) an ancestral bidirectional promoter was considered to be conserved in a given species if it was detected using CAGE-seq data; or (ii) in addition to these, if the two genes were present in a 5'-to-5' orientation with no intervening genes, they were also

assumed to be conserved (higher bound for conservation). Losses in the phylogenetic tree were inferred using parsimony. Therefore, the number of disentanglements in stem vertebrates is likely an overestimate, since losses occurring independently in the lineages leading to mouse and zebrafish since their split were considered a single loss in stem vertebrates by our approach.

8) METHYLOME ANALYSES

8.1) MethylC-seq data analyses

The reads in FASTQ format were mapped to the *in silico* bisulfite-converted *B. lanceolatum* reference genome [114,115] using the Bowtie alignment algorithm (-e 120 -l 20 -n 0) [47]. To estimate the bisulfite non-conversion frequency, the frequency of all cytosine base-calls at CH positions (where H = A, C, T) was divided by the total number of base-calls at CH positions. Sequencing and mapping metrics, including bisulfite conversion rates, are summarized in Supplementary Dataset 5.

8.2) Relationship between DNA methylation and other genomic features

To assess the relationships between DNA methylation, histone modifications (H3K4me3, H3K27me3 and H3K27ac) and CpG density, the genome was partitioned into non-overlapping 1 kbp bins. The genomic coverage (number of reads mapping to each bin) was calculated using Bedtools (*coverageBed*) [102]. Only bins corresponding to "Sc" scaffolds (xp and xf scaffolds removed) and containing a minimum of 10 reads and 20 CpG sites were used for further analyses. Plots were generated in R using the *smoothScatter* function. The lines were fitted using the smoothing spline method (*smooth.spline* in R).

To assess whether transcript abundance and gene body DNA 5mC levels are correlated, we obtained a set of reference transcripts by retaining only the most highly expressed isoform of each locus from all gene models in the consensus annotation (n = 90,927). Out of those, 35,847 were not expressed at 15hpf (0th decile, Extended Data Fig. 5b). The rest were divided into ten equally sized bins (1st-10th deciles), and average DNA methylation levels (mCG/CG) were plotted over their gene bodies using *computeMatrix* and *plotProfile* algorithms from the deepTools suite [116] with the following parameters: computeMatrix scale-regions --beforeRegionStartLength 3000 --regionBodyLength 5000 --afterRegionStartLength 3000.

Average methylation of repetitive elements was calculated using *overlapRatios* function (https://github.com/astatham/aaRon/blob/master/R/overlaps.R). CpG dinucleotides with sequencing coverage less than 5x were filtered out, and repetitive elements with less than 50% of CpGs covered were not taken into consideration. For Extended Data Fig. 1f, average methylation was split into 10 bins (from 0-10% to 90-100%) and percentage of repeats falling into each bin was calculated.

8.3) Identification and characterization of Differentially Methylated Regions (DMRs)

DMRs in CpG context were identified as previously described [115]. Firstly, a root mean square test on each CpG site across all samples was performed. The P-values were simulated using 10,000 permutations. The largest P-value cut-off that still fulfilled the 0.01 FDR prerequisite was chosen. Differentially methylated sites were then combined into blocks if they were within 500 bp of one another and displayed methylation changes in the same direction. Furthermore, blocks containing less than 10 differentially methylated sites were discarded.

We identified a total of 30,047 DMRs (Supplementary Dataset 7), with a median size of 519 bp (Extended Data Fig. 5f). The majority of the DMRs (88%) corresponded to the 36hpf – liver transition (Extended Data Fig. 5e). Of those, 84% (n = 22,333) were hypomethylated in the liver when compared to 36hpf. The DMRs displayed median changes in the fraction of methylated CpG sites (ΔmCG) ranging between 0.2 and 0.42 (Extended Data Fig. 5e) and were widely distributed across the genome (Extended Data Fig. 5g).

8.4) Dynamics of methylation in differentially regulated ATAC-seq peaks

To identify peaks that were differentially enriched in ATAC-seq signal between the three embryonic stages (8hpf, 15hpf and 36hpf) and the adult hepatic diverticulum sample, ATAC-seq peaks were called using MACS2 [48] with default settings, on ATAC-seq fragments extended to 50 bp on each side from the predicted cut site. Differential enrichment was obtained with *DiffBind* from Bioconductor (http://bioconductor.org/packages/release/bioc/html/DiffBind.html). The analysis was performed with default settings except for: *dba.contrast* (minMembers=2) and *dba.report* (th=0.01, which sets the FDR threshold to 0.01). Only peaks displaying at least a 4-

fold change in read abundance were included in further analyses. The k-means clustering and visualization were performed using the deepTools suite [116] algorithms: *computeMatrix* (reference-point --referencePoint center -b 3000 -a 3000 -bs 50) and *plotHeatmap* (--kmeans 2). For the computation of ATAC-seq matrices, missing data values were set to zero (--missingDataAsZero), whereas, for the DNA methylation maps, missing values were replaced with the average genomic *B. lanceolatum* DNA methylation level (mCG/CG = 0.235).

To assess whether the liver-specific ATAC-peaks associated with DNA demethylation contained enriched transcription factor (TF) binding motifs associated with liver function, such as *Hnf4a* [117], or with general and broadly expressed pioneer TFs such as *Foxa* [118-120], we performed a motif search using the Homer suite (homer.salk.edu/), and the library of motifs described below in section 9.1. Specifically, we used the findMotifsGenome.pl script with default settings except for: -size 100 -mask. GO enrichment analyses for the closest genes were performed as described above, using the full gene set with GO annotations as background.

8.5) RRBS data analyses

Raw reads in FASTQ format were mapped to the *in silico* bisulfite-converted *B. lanceolatum* reference genome using WALT [121], allowing for up to 5 mismatches per read. Cytosine base calls of uniquely mapped reads were determined using MethylDackel (https://github.com/dpryan79/MethylDackel). To estimate the bisulfite non-conversion frequency, the frequency of all cytosine base-calls at CH positions (where H = A, C, T) was divided by the total number of base-calls at CH positions. In all cases, the non-conversion rate was < 0.5%. The total number of methylated cytosine calls was divided by the total number of reads spanning that position for each CpG detected within differentially accessible APREs as determined by ATAC-seq. The methylation levels of CpGs were stratified into highly methylated (≥ 0.8), unmethylated (0), intermediately methylated (>0.2 <0.8) and poorly methylated (>0 ≤ 0.2) CpGs and represented as barcharts.

9) ATAC-seq PEAK ANALYSES

9.1) Distribution of phastCons scores among different developmental peaks

We first created a consensus set of ATAC-seq peaks in amphioxus by merging all peaks called in each of the samples (four developmental stages and hepatic diverticulum). Peaks from two different samples were considered the same if they overlapped by at least one nucleotide. Next, we evaluated the "activity profile" of each consensus peak across the time course by considering "active" any peak that was independently called at any of the specific stages and "negative" those that were not. Based on this binary profile, we defined as dynamic peaks with no temporal discontinuity of activity (or "dynamic peaks") those consensus peaks that had any of the following profiles: '00011', '00110', '01100', '11000', '00111', '01110', '11100', '01111', '11110'. A similar logic was applied for zebrafish (but with a total of six stages).

For each stage, we examined the distribution of phastCons conservation scores (see 3.5) averaged over the range of ATAC-seq peaks for both stage-specific and dynamic peaks active in that particular stage. All peaks are larger than 73 bp and shorter than 3,243 bp. As a control, we assessed conservation scores after shuffling the location of peaks in the genome using Bedtools [102] (*shuffle* command).

9.2) Number of ATAC-seq peaks in regulatory landscapes

We first defined two regulatory regions for each protein-coding gene, as previously described [122]. First, the BASAL (or proximal) regulatory region of a given gene spans 5 kbp upstream and 1 kbp downstream of the TSS with the highest number of associated annotated transcripts or until a TSS from another gene model is found. BASAL regions were used for all motif analyses. Next, to define the GREAT regulatory region, the BASAL domain is extended up to 1 Mbp in both directions, until another BASAL region is found. In all related analyses, we counted ATAC-seq peaks (i.e. APREs) in the GREAT regions. For this, peaks were first filtered for overlap with repeats, discarding all peaks that overlapped by at least 30% of their length with any repeat. Filtered peaks were then counted in the GREAT region for each stage as well as for the consensus peak set and used for different downstream analyses. For the comparisons shown in Fig. 5a-c and Extended Data Fig. 9a, only genes from homology families with one gene in amphioxus and between one and four in mouse (used as a reference for vertebrates) were compared, for consistency. Comparing all genes in Fig. 5a produced the same results (highly significantly more APREs per gene in vertebrates). In addition, using only APREs with

H3K27ac ChIP-seq support and/or within 1 kbp of an annotated TSS with H3K4me3 ChIP-seq support produced similar results (Supplementary Fig. 7a,b).

Stratification of gene sets by GREAT or intergenic region size between amphioxus and zebrafish (Fig. 5d and Extended Data Fig. 9g,h) was done using the function *stratify* from the *matt* suite [123] (http://matt.crg.eu), with a range of +/- 500 bp. For the downsampling analyses (Extended Data Fig. 9b,c), the "effective genome" size of each species was calculated as the species' genome size minus its repeat masked and undetermined (i.e. Ns) regions (amphioxus: 322 Mbp; zebrafish: 612 Mbp; medaka: 677 Mbp). Next, we calculated the number of nucleosome-free reads per kbp of effective genome in our experiments to identify the lowest coverage (~28 reads/kbp for the 15hpf sample in amphioxus), which we refer to as "minimum read coverage". Then, for each sample to be compared (15hpf in amphioxus and 8 somites in zebrafish and medaka), we randomly selected nucleosome-free reads to have subsets with decreasing percentages of the minimum read coverage with 5% intervals (from 100% up to 5%), and called and counted peaks in the GREAT regions for those subsets of reads as described above. These ATAC-seq peak counts in the vertebrate subsets were compared to the amphioxus subset with 100% of the minimum coverage employing one-sided Mann-Whitney U tests.

10) CIS-REGULATORY CONTENT COMPARISONS

10.1) TF annotation and TF binding specificity prediction

We identified putative TFs by scanning the amino acid sequences of predicted protein-coding genes for putative DNA binding domains (DBDs) and, when possible, we predicted the DNA binding specificity of each TF using the procedures described in [124]. Briefly, we scanned all protein sequences for putative DBDs using the 81 Pfam [125] models listed in [126] and the HMMER tool [127], with the recommended detection thresholds of per-sequence E-value < 0.01 and per-domain conditional E-value < 0.01. Each protein was classified into a family based on its DBD and its order in the protein sequence (e.g., bZIPx1, AP2x2, Homeodomain+Pou). We then aligned the resulting DBD sequences within each family using clustalOmega [128], with default settings. For protein pairs with multiple DBDs, each DBD was aligned separately. From these alignments, we calculated the sequence identity of all DBD sequence pairs (i.e. the percentage of amino acid residues that are exactly the same across all positions in the alignment). Using

previously established sequence identify thresholds for each family [124], we mapped the predicted DNA binding specificities by simple transfer. For example, the DBD of BL07183_evm0 (*UNCX*) is 88% identical to the zebrafish BX908797.1 protein. Since the DNA binding specificity of BX908797.1 has already been experimentally determined, and the cut-off for the Homeodomain family of TFs is 70%, we can infer that BL07183_evm0 will have the same binding specificity as BX908797.1. This approach produced a table with putative TFs and associated binding motifs (when possible) for amphioxus (Supplementary Dataset 10).

Next, we created a set of related motifs using the following procedure. We first downloaded all motif and TF information from CIS-BP version 1.02 [124]. For all motifs in Supplementary Dataset 10, we selected similar motifs from CIS-BP based on the Jaccard Index (intersection divided by union) of associated TFs; Jaccard Index ≥ 0.95 was used as the threshold. In addition, based on NCBI taxonomy, we selected all motifs from the CIS-BP database that were associated with vertebrate proteins. From this set of motifs, we selected all motifs where the sum of the Information Content (IC) of all individual positions was at least 5. We clustered the motifs using GimmeMotifs [129] with a threshold of 0.9999 ($p \leq 0.0001$). All clustered motifs with a total IC ≤ 5 were discarded. The motif clusters were then associated with TFs based on the annotation of the individual motifs in CIS-BP and Supplementary Dataset 10. Next, we performed an additional step to assign motif clusters to orthologous groups. For this purpose, we took the motif clusters that were assigned to any zebrafish TF, and this assignment was transferred to all homologs in the orthologous group. This procedure resulted in a list of 462 motif clusters assigned to one or more orthologous groups. Finally, if the set of assigned orthologous groups was identical for any given pair of motif clusters, these clusters were considered as a single motif cluster. This approach yielded a list of 242 clusters of motifs assigned to one or more orthologous groups in both amphioxus and zebrafish (Supplementary Dataset 10), which were used for further analyses.

10.2) TF motif mapping onto ATAC-seq peaks

To identify TFs that potentially bind to ATAC-seq peaks, we first used *gimme threshold,* from GimmeMotifs [129], to determine the detection threshold (1% false positive rate; FPR) for each TF motif, based on randomly selected genomic sequences with a similar GC content distribution as

the ATAC-seq peaks. With these thresholds, we scanned all ATAC-seq peaks for our consensus motif clusters using *gimme scan* from GimmeMotifs version 0.11.0 [129].

10.3) Comparison of motif similarity at embryonic stages

For each dynamic peak (sees Section 9.1) in amphioxus and zebrafish, we obtained the number of hits for each motif cluster. Then, for each stage and motif cluster, the number of hits in peaks that are active at that stage was divided by the total number of hits of that motif cluster across all valid peaks and standardized between the different developmental stages by calculating relative motif count z-scores for each motif cluster. This gave an array of values of standardized relative motif counts for each stage and species, which was used to compute pairwise correlations between all amphioxus and all zebrafish developmental stages.

10.4) Comparison of motif enrichment among WGCNA modules

We first obtained the number of hits for each motif cluster within the BASAL region of each gene. Then, the motif counts for all the genes in a given WGCNA module were divided by the total number of genes in each module and standardized between modules by calculating relative motif count z-scores for each motif cluster. As above, for each WGCNA module and species, we obtained an array with normalized scores for each motif cluster (Supplementary Dataset 17), which was then used to compute pairwise correlations between all modules from both species. The z-scores of motif clusters with a z-score higher than 1.5 for significantly correlated modules were plotted for each selected module in both species. Using only APREs with H3K27ac ChIP-seq support and/or within 1 kbp of an annotated TSS with H3K4me3 ChIP-seq support produced very similar results in terms of ortholog overlap and TF motif enrichment (Supplementary Fig. 7c-e).

11) WGD IMPACT ON GENE REGULATION AND EXPRESSION

11.1) Comparison of expression domains in ohnologs versus singletons

To be able to compare expression between amphioxus and vertebrates, we first selected nine samples that, despite morphological differences, could be unambiguously matched as homologs among the four studied species (amphioxus, zebrafish, frog and mouse): eggs/oocyte, mid-stage embryo, male gonads, female gonads, digestive tract, hepatic diverticulum/liver, epidermis/skin,

skeletal muscle and neural tube (Supplementary Table 7). cRPKM values were obtained for each sample and species, and normalized using quantile normalization as implemented in *normalizebetween* arrays from the *limma* package. Then, for each vertebrate species, we compared the expression of single-copy orthologs and ohnologs (see Section 4.2) with that of amphioxus. For ohnologs, we restricted our analysis to those families with a single amphioxus member and two or more in the studied vertebrate species.

To perform this comparison (see scheme in Fig. 6a), for each species and sample, the expression of each gene was binarized, and a gene was considered expressed, if it had a normalized cRPKM value higher than 5, and not expressed if ≤ 5 (use of different cut-offs produced very similar qualitative patterns). This produced a binary expression array of nine elements (0 or 1) for each gene and species. Next, for each ohnolog group in the vertebrate species, we built two additional binary expression arrays. One corresponded to the union of positive expression domains across all members of the family ("OHNO_fam"). The other one was built after summing the normalized cRPKM values of all the members for each expression domain and applying the same definition for positive expression (cRPKM > 5; "OHNO_fam_S"). Then, for each pair of orthologs in which at least one of the genes is expressed in at least one of the nine samples, we calculated the difference between the number of samples in which the amphioxus ortholog is expressed and the number of samples in which the vertebrate ortholog is expressed. A similar comparison was done for the amphioxus gene versus the OHNO_fam and OHNO_fam_S arrays. These values were plotted as histograms in Fig. 6b and Extended Data Figure 10a,b. In total, we compared 2,478 single pairs and 3,427 ohnologs in 1,135 families for zebrafish, 2,450 single pairs and 3,011 ohnologs in 1,212 families for mouse, and 2,482 single pairs and 2,635 ohnologs in 1,094 families for frog.

11.2) Investigation of the fates of ohnologs with regards to ancestral expression

Using the same reasoning as above, for each vertebrate species, we selected families of ohnologs with expression across all nine samples for both the union array (OHNO_fam) and the amphioxus counterpart (370 families for mouse, 360 for frog and 366 for zebrafish), and thus presumably also in the pre-duplicative ancestral gene. We then evaluated the possible fates for each of the vertebrate families and defined the following scenarios (Fig. 6c): (i) Redundancy: if

all the family members have expression across the nine domains in the vertebrate species; (ii) Subfunctionalization: if none of the members have expression across all nine domains; and (iii) Specialization: if at least one of the members has expression across all nine domains, but at least one member does not. In the last scenario, for the members that have underwent restriction of expression (thereafter 'specialized ohnologs'), we also distinguished between strong and mild specialization, depending on whether the ohnolog is expressed in ≤ 2 or ≥ 3 expression domains, respectively. Based on these data, we also scored the number of domains lost from the ancestral state for each specialized ohnolog (from 1 to 9). Finally, to assess whether the different types of ohnologs where expressed in tissues that do not have an homologous counterpart in amphioxus or that have been greatly elaborated and modified in vertebrates (e.g. eye and lens, pancreas, lung, heart, neural crest cell, kidney; see Supplementary Table 17) we have collected RNA-seq data from multiple such sources for mouse, frog and zebrafish (Supplementary Dataset 3) and scored binary expression as described above. Overall, strongly specialized ohnologs were not generally expressed in any of these tissues, with the exception of neural-associated tissues (such as retina/eye, and endocrine pancreatic cells which share regulatory programs with neurons) (Supplementary Fig. 8). Thus, we conclude that the increase in APREs observed in strongly specialized ohnologs is not likely to be a result of adding new expression domains, but rather to the need for more complex regulation as a gene evolves from having broad expression pattern to having tissue-specific expression.

11.3) Sequence conservation and dN/dS for different fates

To compare the evolutionary rates of different sets of ohnologs based on their fate upon WGD, we downloaded the dN, dS and percent identity for mouse genes against human homologs from Ensembl Biomart (release 80). The percent identity and the dN/dS ratio were plotted for each mouse gene with 1-to-1 orthologs according to Ensembl.

11.4) *Tau* analyses of ohnologs

We computed the *Tau* tissue-specificity index as previously described [130], using the following expression:

$$\tau = \frac{\sum_{i=1}^{n} 1 - \hat{x}_i}{n-1} \quad \text{with} \quad \hat{x}_i = \frac{x_i}{\max\limits_{1 \le i \le n} (x_i)}$$

where x_i is the expression component and n the number of components (here, samples).

To calculate *Tau*, we used a comparable subset of stages and tissues in each species (Supplementary Table 18): six embryonic stages covering the full developmental time course, heart (when present), muscle, gut, pancreas, liver or hepatic diverticulum (amphioxus), kidney, gill, skin, sensory organs, eye, brain, testis, ovary, sperm. For mouse, spleen, thymus and placenta were also used. Following [131], we attempted to evaluate tissue specificity changes across reconstructed paralogous families by plotting the *Tau* value for each amphioxus gene versus the ohnolog with the highest *Tau* score in the vertebrate species.

11.5) *In situ* hybridization assays for specialized families in zebrafish and amphioxus

To identify zebrafish families that have undergone specialization and have available *in situ* hybridization data, we manually searched the Zfin database (http://zfin.org) for such gene families in which *in situ* hybridization data at 24hpf-48hpf had been previously reported for at least one of the specialized ohnologs and one of the "equal" ohnologs. This rendered 23 families. Since we have defined specialization based on RNA-seq data from adult tissues, from the original 23 families we prioritized for further analyses 13 cases in which the specialized expression pattern could already be detected during zebrafish embryonic stages (24h and 48h). Given that the expression patterns of "equal" ohnologs was always annotated as not spatially restricted in Zfin, the only available images for most of these genes were general overviews of mixed developmental stages.

In the case of amphioxus, we purchased *in situ* hybridization probes for the orthologs of these 13 genes, but only 11 probes could be successfully synthesized (Supplementary Table 19). Synthesized cDNA sequences were then cloned into pBluescript II SK at the EcoR1 restriction site. Linearization was done using Xho1 restriction enzyme and riboprobe synthesis was performed employing a DIG labeling kit with the T3 RNA polymerase. Embryos were fixed in PFA 4% dissolved in MOPS-EGTA buffer at different developmental stages and processed for in

situ hybridization as previously described in [132], with slight modifications: the chromogenic reaction was developed using BCIP/NBT and proteinase K digestion was omitted. *MLC* (sequence EU685290.1) was used as a positive control for a gene with tissue-restricted expression. The corresponding amphioxus *in situ* hybridization expression patterns are shown in Extended Data Fig. 10h,i and Supplementary Fig. 9.

Statistics and Reproducibility

All boxplots in Main and Extended Data and Supplementary Figures represent the R and python default for the boxplot function: the boxes show the interquartile range (IQR) around the median, and the whiskers extend from the minimum value to the maximum value unless the distance to the first or third quartile was more than 1.5 times the IQR. In all cases, outliers were not plotted for simplicity. P-values and sample sizes for relevant comparisons are provided in Supplementary Dataset 8.

Detailed Author Contributions

Study design and project coordination. F.M., P.F., I.M., J.J.T, O.B., M.P., B.L., P.W.H.H., H.E., J.L.G.-S., M.I. concept and study design. I.M., H.E., J.L.G.-S. and M.I. coordinated the project. H.E. wrote the initial genome project submitted to Genoscope and extracted genomic DNA for genome sequencing. M.I., I.M., P.W.H. and F.M. wrote the main text, with input from all authors.

B. lanceolatum genome sequencing and annotation. J.M.A., S.M., P.W. (Genoscope) sequenced the genome. F.M. made the genome assembly and repeat annotation. F.M., D.B., M.I. made the gene annotation. F.M., M.I. made the orthology assignment and built gene families. F.M. set up the amphioxus UCSC hub. A.L., H.R.C. set up the amphioxus Genomicus browser. R.A., E.B.-G., C.C., F.C., S.D.A., D.E.K.F., S.H., V.L., G.A.B.M., P.P., M.S., H.S., I.S., T.T., O.M., A.X., J-K.Y. contributed to genome sequencing and gene family curation.

Material collection and data generation. D.A., Y.L.P., A.L.F, L.S., S.B. and H.E. worked on amphioxus collection, animal care, spawning induction and embryos collection. D.B., J.P., S.B., and L.S. collected, dissected and extracted RNA from amphioxus samples. M.I., H.E., Genoscope, J.G.F, generated amphioxus RNA-seq data. J.P. collected, dissected and extracted RNA from zebrafish samples. M.I. generated zebrafish RNA-seq data. L.B. collected and

extracted RNA from medaka samples. J.R.M.-M. generated medaka RNA-seq data. D.B., J.P., S.B., and L.S. collected, dissected and extracted RNA for amphioxus CAGE-seq. B.L., P.C. generated amphioxus CAGE-seq data. D.B., J.P., I.M., E.C.-M., S.B., and H.E. collected, dissected and extracted DNA for amphioxus MethylC/RRBS. O.B., R.L. generated amphioxus MethylC/RRBS data. S.B., D.A., B.A.-C., I.M. collected and fixed samples for amphioxus ChIP-seq. B.A.-C., I.M. set up the fixation protocol for amphioxus ChIP-seq. E.C.-M. set up the amphioxus ChIP-seq protocol. E.C.-M., I.M. did the ChIP-seq experiments. I.M., S.J.-G., S.B. did the amphioxus ATAC-seq experiments. J.L.G.-S. generated ChIP-seq, ATAC-seq and 4C-seq data. E.C.-M. and E.F. did the zebrafish ATAC-seq experiments. E.F. did the medaka ATAC-seq experiments. S.B. collected and fixed samples for amphioxus 4C-seq. R.D.A., S.N. did the amphioxus, zebrafish and mouse 4C-seq experiments. R.D.A., I.M. cloned APRES for enhancer reporter assays (EDF2e). E.C.-M., J.J.T., R.D.A., I.M. performed zebrafish enhancer reporter assays (EDF2e). I.K., Z.K. performed amphioxus enhancer reporter assays (EDF2f).

Analyses of functional genomics data. J.J.T. analyzed ChIP-seq data (EDF2d). R.D.A. and I.M. analyzed 4C-seq data (EDF9e). C.H.-U. and J.G.F. did the lncRNAs analyses (EDF1g). J.J.T. analyzed APRES genomic distributions, developmental dynamics, histone modifications and TF motifs content (EDF2g-i). M.P., P.B. and B.L. analyzed CAGE-seq data (EDF3). M.P., B.L. and M.I. investigated bidirectional promoters (EDF4). O.B. analyzed MethylC/RRBS data and methylation of TEs, with contributions from K.S., P.E.D., M.I. and I.M. (Fig. 2, EDF1, EDF5, EDF6). F.M. did the phylotypic stage analyses of transcriptomics (Fig. 3a,b and EDF7). P.F. performed the phylotypic stage analyses of APREs (Fig. 3c). F.M. did the phastCons sequence conservation analyses (Fig. 3d). S.J.vH., M.W., P.F., I.M., M.I. did the DBP and TF motifs annotation and clustering. Y.M. identified orthologous introns between amphioxus and vertebrates. P.F., I.M., C.W. and M.I. did the analysis of developmental and tissue expression and regulatory conservation (Fig. 4 and EDF8). P.F. and M.I. did the comparative analyses on the amount and distribution of APREs (Fig. 5, EDF9). M.I. did the DDC, subfunctionalization and specialization analyses (Fig. 6, EDF10, SF8). F.M. did the Tau analyses (EDF10c-e). S.B. performed the amphioxus ISH for specialization analyses (EDF10h,i, SF9). I.M., J.L.G.-S. visualized the profiles of the functional genomics data in the genome browser and generated the different genome browser captures (Fig1b, EDF6f, SF1, SF2). J.L.G.-S., I.M. screened publicly available zebrafish ISH data for specialization analyses (EDF10h,i, SF9).

Additional funding sources

In addition those sources listed in the main text, this work has been supported by the Andalusian Government (BIO-396 to J.L.G.-S.), Royal Society International Exchanges grant to P.W.H.H., the Spanish Ministerio de Economía y Competitividad (BFU2014-58449-JIN to J.J.T., BFU2014-58908P and BFU2017-86152 to J.G.F., BFU2016-80601-P to C.C., BIO2015-67358-C2-1-P to R.A.), ICREA - Generalitat de Catalunya (Academia Prize to J.G.F.), the Institut Universitaire de France to S.B., the Taiwan Ministry of Science and Technology (MOST105-2628-B-001-003-MY3 to J.-K.Y.), the Academia Sinica (AS-98-CDA-L06 to J.-K.Y.), the Czech Science Foundation (15-21285J and 17-15374S to Z.K.), a Leverhulme Trust Research Project Grant (RPG-2014-370 to T.T.), an European Molecular Biology Organization (EMBO) short fellowship (ASTF377-2014 to I.M.). C.D.R.W. a La Caixa PhD fellowship, D.B. an APIF fellowship from University of Barcelona, Y.M. an EMBO Long Term postdoctoral fellowship (ALTF 1505-2015), C.H.-U. a predoctoral FPI Fellowship (Spanish Ministerio de Economía y Competitividad). We thank the CRG Genomics Unit for their help with high-throughput sequencing.

References

1 Pallas, P. S. *Naturgeschichte merkwurdiger tiere in welcher vornehmlich neue unbekannte tierarten durch kupferstiche, beschreibungen und erklärungen erläutert werden*. (Lange, Gottlied A., 1774).

2 Bertrand, S. & Escriva, H. Evolutionary crossroads in developmental biology: amphioxus. *Development* **138**, 4819-4830 (2011).

3 Delsuc, F., Brinkmann, H., Chourrout, D. & Philippe, H. Tunicates and not cephalochordates are the closest living relatives of vertebrates. *Nature* **439**, 965-968 (2006).

4 Poss, S. G. & Boschung, H. T. Lancelets (Cephalochordata: Branchiostomidae): How many species are valid? *Isr J Zool* **42**, S13-66 (1996).

5 Kon, T. *et al.* Phylogenetic position of a whale-fall lancelet (Cephalochordata) inferred from whole mitochondrial genome sequences. *BMC Evol Biol* **7**, 127 (2007).

6 Igawa, T. *et al.* Evolutionary history of the extant amphioxus lineage with shallow-branching diversification. *Sci Rep* **7**, 1157 (2017).

7 Kowalevsky, A. O. Entwicklungsgeschichte des *Amphioxus lanceolatus*. *Mém. Acad. Sci. St. Petersb.* **11**, 1-17 (1867).

8 Monniot, F. Recherches sur les graviers a amphioxus de la region de Banyuls sur Mer. *Vie et Milieu* **XIII**, 232-322 (1962).

9 Nelson, G. Amphioxus in old Tampa Bay, Florida. *Q J Fl Acad Sci* **31**, 93-100 (1969).

10 Desdevises, Y., Maillet, V., Fuentes, M. & Escriva, H. A snapshot of the population structure of Branchiostoma lanceolatum in the Racou beach, France, during its spawning season. *PLoS One* **6**, e18520 (2011).
11 Atkin, N. B. & Ohno, S. DNA values of four primitive chordates. *Chromosoma* **23**, 10-13 (1967).
12 Putnam, N. *et al.* The amphioxus genome and the evolution of the chordate karyotype. *Nature* **453**, 1064-1071 (2008).
13 Huang, S. *et al.* Decelerated genome evolution in modern vertebrates revealed by analysis of multiple lancelet genomes. *Nat Commun* **5**, 5896 (2014).
14 Nogusa, S. The chromosomes of the Japanese lancelet, Branchiostoma belcheri (Gray), with special reference to the sex-chromosomes. *Annotationes Zoologicae Japonenses* **30**, 42-46 (1957).
15 Saotome, K. & Ojima, Y. Chromosomes of the lancelet Branchiostoma belcheri Gray. *Zoological Sci* **18**, 683-686 (2001).
16 Wang, C., Zhang, S. & Chu, J. G-banding patterns of the chromosomes of amphioxus Branchiostoma belcheri tsingtauense. *Hereditas* **141**, 2-7 (2004).
17 Howell, W. M. & Boschung, H. T. J. Chromosomes of the lancelet, Branchiostoma floridae (order Amphioxi). *Experientia* **27**, 1495-1496 (1971).
18 Colombera, D. Male chromosomes in two populations of Branchiostoma lanceolatum. *Experientia* **30**, 353-355 (1974).
19 Dehal, P. & Boore, J. L. Two rounds of whole genome duplication in the ancestral vertebrate. *PLoS Biol* **3**, e314 (2005).
20 Paps, J., Holland, P. W. & Shimeld, S. M. A genome-wide view of transcription factor gene diversity in chordate evolution: less gene loss in amphioxus? *Brief Funct Genomics* **11**, 177-186 (2012).
21 Takatori, N. *et al.* Comprehensive survey and classification of homeobox genes in the genome of amphioxus, Branchiostoma floridae. *Dev Genes Evol* **218**, 579-590 (2008).
22 D'Aniello, S. *et al.* Gene expansion and retention leads to a diverse tyrosine kinase superfamily in amphioxus. *Mol Biol Evol* **25**, 1841-1854 (2008).
23 Holland, L. Z. & Short, S. Gene duplication, co-option and recruitment during the origin of the vertebrate brain from the invertebrate chordate brain. *Brain Behav Evol* **72**, 91-105 (2008).
24 Royo, J. L. *et al.* Transphyletic conservation of developmental regulatory state in animal evolution. *Proc Natl Acad Sci USA* **108**, 14186-14191 (2011).
25 Clarke, S. L. *et al.* Human developmental enhancers conserved between deuterostomes and protostomes. *PLoS Genet* **8**, e1002852 (2012).
26 Irimia, M. *et al.* Extensive conservation of ancient microsynteny across metazoans due to cis-regulatory constraints. *Genome Res* **22**, 2356-2367 (2012).
27 Acemel, R. D. *et al.* A single three-dimensional chromatin compartment in amphioxus indicates a stepwise evolution of vertebrate Hox bimodal regulation. *Nat Genet* **48**, 336-341 (2016).
28 Fuentes, M. *et al.* Preliminary observations on the spawning conditions of the European amphioxus (Branchiostoma lanceolatum) in captivity. *J Exp Zool B Mol Dev Evol* **302**, 384-391 (2004).

29 Fuentes, M. *et al.* Insights into spawning behavior and development of the European amphioxus (Branchiostoma lanceolatum). *J Exp Zool B Mol Dev Evol* **308**, 484-493 (2007).

30 Hirakow, R. & Kajita, N. Electron microscopic study of the development of amphioxus, Branchiostoma belcheri tsingtauense: the gastrula. *J Morph* **207**, 37–52 (1991).

31 Hirakow, R. & Kajita, N. Electron microscopic study of the development of amphioxus, Branchiostoma belcheri tsingtauense: the neurula and larva. *Kaibogaku Zasshi.* **69**, 1-13 (1994).

32 Bogdanović, O. & Veenstra, G. J. Affinity-based enrichment strategies to assay methyl-CpG binding activity and DNA methylation in early Xenopus embryos. *BMC Res Notes* **4**, 300 (2011).

33 Li, R. *et al.* SOAP2: an improved ultrafast tool for short read alignment. *Bioinformatics* **25**, 1966-1967 (2009).

34 Iwamatsu, T. Stages of normal development in the medaka Oryzias latipes. *Mech Dev* **121**, 605-618 (2004).

35 Murata, M. *et al.* Detecting expressed genes using CAGE. *Methods Mol Biol* **1164**, 67-85 (2014).

36 Forrest, A. R. *et al.* A promoter-level mammalian expression atlas. *Nature* **507**, 462-470 (2014).

37 Haberle, V., Forrest, A. R., Hayashizaki, Y., Carninci, P. & Lenhard, B. CAGEr: precise TSS data retrieval and high-resolution promoterome mining for integrative analyses. *Nucleic Acids Res* **43**, e51 (2015).

38 Nepal, C. *et al.* Dynamic regulation of the transcription initiation landscape at single nucleotide resolution during vertebrate embryogenesis. *Genome Res* **23**, 1938-1950 (2013).

39 Buenrostro, J. D., Giresi, P. G., Zaba, L. C., Chang, H. Y. & Greenleaf, W. J. Transposition of native chromatin for fast and sensitive epigenomic profiling of open chromatin, DNA-binding proteins and nucleosome position. *Nat Methods* **10**, 1213-1218 (2013).

40 Fernandez-Minan, A., Bessa, J., Tena, J. J. & Gomez-Skarmeta, J. L. Assay for transposase-accessible chromatin and circularized chromosome conformation capture, two methods to explore the regulatory landscapes of genes in zebrafish. *Methods Cell Biol* **135**, 413-430 (2016).

41 Bessa, J. *et al.* Zebrafish enhancer detection (ZED) vector: a new tool to facilitate transgenesis and the functional analysis of cis-regulatory regions in zebrafish. *Dev Dyn* **238**, 2409-2417 (2009).

42 Gehrke, A. R. *et al.* Deep conservation of wrist and digit enhancers in fish. *Proc Natl Acad Sci USA* **112**, 803-808 (2015).

43 Kawakami, K. Transgenesis and gene trap methods in zebrafish by using the Tol2 transposable element. *Methods Cell Biol* **77** (2004).

44 Feng, J., Li, G., Liu, X., Wang, J. & Wang, Y. Q. Functional analysis of the promoter region of amphioxus β-actin gene: a useful tool for driving gene expression in vivo. *Mol Biol Rep* **41**, 6817-6826 (2014).

45 Ding, S. *et al.* Efficient transposition of the piggyBac (PB) transposon in mammalian cells and mice. *Cell* **122**, 473-483 (2005).

46 Bogdanovic, O., Fernández-Miñán, A., Tena, J. J., de la Calle-Mustienes, E. & Gómez-Skarmeta, J. L. The developmental epigenomics toolbox: ChIP-seq and MethylCap-seq profiling of early zebrafish embryos. *Methods* **62**, 207-215 (2013).
47 Langmead, B., Trapnell, C., Pop, M. & Salzberg, S. L. Ultrafast and memory-efficient alignment of short DNA sequences to the human genome. *Genome Biol* **10**, R25 (2009).
48 Zhang, Y. *et al.* Model-based analysis of ChIP-Seq (MACS). *Genome Biol* **9**, R137 (2008).
49 Geeven, G., Teunissen, H., de Laat, W. & de Wit, E. peakC: a flexible, non-parametric peak calling package for 4C and Capture-C data *Nucleic Acids Res*, doi: 10.1093/nar/gky1443. (2018).
50 Marçais, G. & Kingsford, C. A fast, lock-free approach for efficient parallel counting of occurrences of k-mers. *Bioinformatics* **27**, 764-770 (2011).
51 Liu, Y., Schröder, J. & Schmidt, B. Musket: a multistage k-mer spectrum-based error corrector for Illumina sequence data. *Bioinformatics* **29**, 308-315 (2013).
52 Magoč, T. & Salzberg, S. L. FLASH: fast length adjustment of short reads to improve genome assemblies. *Bioinformatics* **27**, 2957-2963 (2011).
53 Luo, R. *et al.* SOAPdenovo2: an empirically improved memory-efficient short-read de novo assembler. *Gigascience* **1**, 18 (2012).
54 Huang, S. *et al.* HaploMerger: reconstructing allelic relationships for polymorphic diploid genome assemblies. *Genome Res* **22**, 1581-1588 (2012).
55 Parra, G., Bradnam, K. & Korf, I. CEGMA: a pipeline to accurately annotate core genes in eukaryotic genomes. *Bioinformatics* **23**, 1061-1067 (2007).
56 Keller, O., Kollmar, M., Stanke, M. & Waack, S. A novel hybrid gene prediction method employing protein multiple sequence alignments. *Bioinformatics* **27**, 757-763 (2011).
57 Slater, G. S. & Birney, E. Automated generation of heuristics for biological sequence comparison. *BMC Bioinformatics* **6**, 31 (2005).
58 Grabherr, M. G. *et al.* Full-length transcriptome assembly from RNA-Seq data without a reference genome. *Nat Biotechnol* **29**, 644-652 (2011).
59 Haas, B. J. *et al.* Improving the Arabidopsis genome annotation using maximal transcript alignment assemblies. *Nucl. Acids Res.* **31**, 5654-5666 (2003).
60 Haas, B. J. *et al.* Automated eukaryotic gene structure annotation using EVidenceModeler and the Program to Assemble Spliced Alignments. *Genome Biol* **9**, R7 (2008).
61 Kim, D. *et al.* TopHat2: accurate alignment of transcriptomes in the presence of insertions, deletions and gene fusions. *Genome Biol* **14**, R36 (2013).
62 Trapnell, C. *et al.* Transcript assembly and quantification by RNA-Seq reveals unannotated transcripts and isoform switching during cell differentiation. *Nat Biotechnol* **28**, 511-515 (2010).
63 Trapnell, C. *et al.* Differential gene and transcript expression analysis of RNA-seq experiments with TopHat and Cufflinks. *Nat Protoc* **7**, 562-578 (2012).
64 Haas, B. J. *et al.* De novo transcript sequence reconstruction from RNA-seq using the Trinity platform for reference generation and analysis. *Nat Protoc* **8**, 1494-1512 (2013).
65 Pauli, A. *et al.* Systematic identification of long noncoding RNAs expressed during zebrafish embryogenesis. *Genome Res* **22**, 577-591 (2012).
66 Wang, L. *et al.* CPAT: Coding-Potential Assessment Tool using an alignment-free logistic regression model. *Nucleic Acids Res* **41**, e74 (2013).

67 Ponting, C. P., Oliver, P. L. & Reik, W. Evolution and functions of long noncoding RNAs. *Cell* **136**, 629-641 (2009).
68 Arriagada, G. *et al.* Activation of transcription and retrotransposition of a novel retroelement, Steamer, in neoplastic hemocytes of the mollusk Mya arenaria. *Proc Natl Acad Sci USA* **111**, 14175-14180 (2014).
69 Finn, R. D. *et al.* Pfam: the protein families database. *Nucleic Acids Res* **42**, D222-230 (2014).
70 Jones, P. *et al.* InterProScan 5: genome-scale protein function classification. *Bioinformatics* **30**, 1236-1240 (2014).
71 Alexa, A., Rahnenführer, J. & Lengauer, T. Improved scoring of functional groups from gene expression data by decorrelating GO graph structure. *Bioinformatics* **22**, 1600-1607 (2006).
72 Cañestro, C. & Albalat, R. Transposon diversity is higher in amphioxus than in vertebrates: functional and evolutionary inferences. *Brief Funct Genomics* **11**, 131-141 (2012).
73 Blanchette, M. *et al.* Aligning multiple genomic sequences with the threaded blockset aligner. *Genome Res* **14**, 708-715 (2004).
74 Siepel, A. *et al.* Evolutionarily conserved elements in vertebrate, insect, worm, and yeast genomes. *Genome Res* **15**, 1034-1050 (2005).
75 Margulies, E. H., M;, B., Program, N. C. S., Haussler, D. & Green, E. D. Identification and characterization of multi-species conserved sequences. *Genome Res* **13**, 2507-2518 (2003).
76 Roth, A. C., Gonnet, G. H. & Dessimoz, C. Algorithm of OMA for large-scale orthology inference. *BMC Bioinformatics* **9**, 518 (2008).
77 Altenhoff, A. M., Gil, M., Gonnet, G. H. & Dessimoz, C. Inferring hierarchical orthologous groups from orthologous gene pairs. *PLoS One* **8**, e53786 (2013).
78 Stamatakis, A. RAxML version 8: a tool for phylogenetic analysis and post-analysis of large phylogenies. *Bioinformatics* **30**, 1312-1313 (2014).
79 Huerta-Cepas, J., Serra, F. & Bork, P. ETE 3: Reconstruction, Analysis, and Visualization of Phylogenomic Data. *Mol Biol Evol* **33**, 1635-1638 (2016).
80 Woolfe, A. *et al.* Highly Conserved Non-Coding Sequences Are Associated with Vertebrate Development. *PLoS Biology* **3**, e7 (2005).
81 Katoh, K., Kuma, K., Toh, H. & Miyata, T. MAFFT version 5: improvement in accuracy of multiple sequence alignment. *Nucleic Acids Res* **33**, 511-518 (2005).
82 Berthelot, C. *et al.* The rainbow trout genome provides novel insights into evolution after whole-genome duplication in vertebrates. *Nat Commun* **5**, 3657 (2014).
83 Murat, F. *et al.* Understanding Brassicaceae evolution through ancestral genome reconstruction. *Genome Biol* **16**, 262 (2015).
84 Nguyen, N. T. T., Vincens, P., Roest Crollius, H. & Louis, A. Genomicus 2018: karyotype evolutionary trees and on-the-fly synteny computing. *Nucleic Acids Res*, 10.1093/nar/gkx1003 (2017).
85 Langmead, B. & Salzberg, S. L. Fast gapped-read alignment with Bowtie 2. *Nat Methods* **9**, 357-359 (2012).
86 Beaster-Jones, L., Schubert, M. & Holland, L. Z. Cis-regulation of the amphioxus engrailed gene: Insights into evolution of a muscle-specific enhancer. *Mech Dev* **124**, 532-542 (2007).

87 Holland, L. Z. *et al.* The amphioxus genome illuminates vertebrate origins and cephalochordate biology. *Genome Res* **18**, 1100-1111 (2008).
88 Hufton, A. L. *et al.* Deeply conserved chordate noncoding sequences preserve genome synteny but do not drive gene duplicate retention. *Genome Res* **19**, 2036-2051 (2009).
89 Irimia, M. *et al.* Comparative genomics of the Hedgehog loci in chordates and the origins of Shh regulatory novelties. *Sci Rep* **2**, 433 (2012).
90 Kozmikova, I. & Kozmik, Z. Gene regulation in amphioxus: An insight from transgenic studies in amphioxus and vertebrates. *Mar Genomics* **24**, 159-166 (2015).
91 Kozmikova, I., Smolikova, J., Vlcek, C. & Kozmik, Z. Conservation and diversification of an ancestral chordate gene regulatory network for dorsoventral patterning. *PLoS One* **6**, e14650 (2011).
92 Kozmikova, I., Candiani, S., Fabian, P., Gurska, D. & Kozmik, Z. Essential role of Bmp signaling and its positive feedback loop in the early cell fate evolution of chordates. *Dev Biol* **382**, 538-554 (2013).
93 Li, D. *et al.* Isolation and functional analysis of the promoter of the amphioxus Hsp70a gene. *Gene* **510**, 39-46 (2012).
94 Maeso, I. *et al.* An ancient genomic regulatory block conserved across bilaterians and its dismantling in tetrapods by retrogene replacement. *Genome Res* **22**, 642-655 (2012).
95 Manzanares, M. *et al.* Conservation and elaboration of Hox gene regulation during evolution of the vertebrate head. *Nature* **408**, 854-857 (2000).
96 Ochi, H. *et al.* Evolution of a tissue-specific silencer underlies divergence in the expression of pax2 and pax8 paralogues. *Nat Commun* **3**, 848 (2012).
97 Punnamoottil, B. *et al.* Cis-regulatory characterization of sequence conservation surrounding the Hox4 genes. *Dev Biol* **340**, 269-282 (2010).
98 Van Otterloo, E. *et al.* Novel Tfap2-mediated control of soxE expression facilitated the evolutionary emergence of the neural crest. *Development* **139**, 720-730 (2012).
99 Wada, H., Escriva, H., Zhang, S. & Laudet, V. Conserved RARE localization in amphioxus Hox clusters and implications for Hox code evolution in the vertebrate neural crest. *Dev Dyn* **235**, 1522-1531 (2006).
100 Yu, J.-K., Holland, N. D. & Holland, L. Z. Tissue-specific expression of FoxD reporter constructs in amphioxus embryos. *Dev Biol* **274**, 452-461 (2004).
101 Yue, J. X. *et al.* Conserved Noncoding Elements in the Most Distant Genera of Cephalochordates: The Goldilocks Principle. *Genome Biol Evol* **8**, 2387-2405 (2016).
102 Quinlan, A. R. & Hall, I. M. BEDTools: a flexible suite of utilities for comparing genomic features. *Bioinformatics* **26**, 841-842 (2010).
103 Bray, N. L., Pimentel, H., Melsted, P. & Pachter, L. Near-optimal probabilistic RNA-seq quantification. *Nat Biotechnol* **34**, 525-527 (2016).
104 Labbe, R. M. *et al.* A comparative transcriptomic analysis reveals conserved features of stem cell pluripotency in planarians and mammals. *Stem Cells* **30**, 1734-1745 (2012).
105 Kumar, L. & Futschik, M. E. Mfuzz: A software package for soft clustering of microarray data. *Bioinformatics* **2**, 5-7 (2007).
106 Langfelder, P. & Horvath, S. WGCNA: an R package for weighted correlation network analysis. *BMC Bioinformatics* **9**, 559 (2008).
107 Yue, F. *et al.* A comparative encyclopedia of DNA elements in the mouse genome. *Nature* **515**, 355-364 (2014).

108 Irimia, M. *et al.* A highly conserved program of neuronal microexons is misregulated in autistic brains. *Cell* **159**, 1511-1523 (2014).
109 Balwierz, P. J. *et al.* Methods for analyzing deep sequencing expression data: constructing the human and mouse promoterome with deepCAGE data. *Genome Biol* **10**, R79 (2009).
110 Wehrens, R. & Buydens, L. M. C. Self- and Super-organising Maps in R: the kohonen package. *J Stat Softw* **21** (2007).
111 Schep, A. N. *et al.* Structured nucleosome fingerprints enable high-resolution mapping of chromatin architecture within regulatory regions. *Genome Res* **25**, 1757-1770 (2015).
112 Huber, W. *et al.* Orchestrating high-throughput genomic analysis with Bioconductor. *Nat Methods* **12**, 115-121 (2005).
113 Celniker, S. E. *et al.* Unlocking the secrets of the genome. *Nature* **459**, 927-930 (2009).
114 Lister, R. *et al.* Hotspots of aberrant epigenomic reprogramming in human induced pluripotent stem cells. *Nature* **471**, 68-73 (2011).
115 Bogdanović, O. *et al.* Active DNA demethylation at enhancers during the vertebrate phylotypic period. *Nat Genet* **48**, 417-426 (2016).
116 Ramírez, F. *et al.* deepTools2: a next generation web server for deep-sequencing data analysis. *Nucleic Acids Res* **44**, W160-165 (2016).
117 Odom, D. T. *et al.* Control of pancreas and liver gene expression by HNF transcription factors. *Science* **303**, 1378-1381 (2004).
118 Aldea, D., Leon, A., Bertrand, E. & Escriva, H. Expression of Fox genes in the cephalochordate Branchiostoma lanceolatum. *Front Ecol Evol* **3**, 80 (2015).
119 Zhang, Y. *et al.* Nucleation of DNA repair factors by FOXA1 links DNA demethylation to transcriptional pioneering. *Nat Genet* **48**, 1003-1013 (2016).
120 Yang, Y. A. *et al.* FOXA1 potentiates lineage-specific enhancer activation through modulating TET1 expression and function. *Nucleic Acids Res* **44**, 8153-8164 (2016).
121 Chen, H., Smith, A. D. & Chen, T. WALT: fast and accurate read mapping for bisulfite sequencing. *Bioinformatics* **32**, 3507-3509 (2016).
122 McLean, C. Y. *et al.* GREAT improves functional interpretation of cis-regulatory regions. *Nat Biotechnol* **28**, 495-501 (2010).
123 Gohr, A. & Irimia, M. Matt: Unix tools for alternative splicing analysis. *Bioinformatics*, doi: 10.1093/bioinformatics/bty1606 (2018).
124 Weirauch, M. T. *et al.* Determination and inference of eukaryotic transcription factor sequence specificity. *Cell* **158**, 1431-1443 (2014).
125 Finn, R. D. *et al.* The Pfam protein families database. *Nucleic Acids Res* **38**, D211-222 (2010).
126 Weirauch, M. T. & Hughes, T. R. A catalogue of eukaryotic transcription factor types, their evolutionary origin, and species distribution. *Subcell Biochem* **52**, 25-73 (2011).
127 Eddy, S. R. A new generation of homology search tools based on probabilistic inference. *Genome Inform* **23**, 205-211 (2009).
128 Sievers, F. *et al.* Fast, scalable generation of high-quality protein multiple sequence alignments using Clustal Omega. *Mol Syst Biol* **7**, 539 (2011).
129 van Heeringen, S. J. & Veenstra, G. J. GimmeMotifs: a de novo motif prediction pipeline for ChIP-sequencing experiments. *Bioinformatics* **27**, 270-271 (2011).
130 Yanai, I. *et al.* Genome-wide midrange transcription profiles reveal expression level relationships in human tissue specification. *Bioinformatics* **21**, 650-659 (2005).

131 Kryuchkova-Mostacci, N. & Robinson-Rechavi, M. Tissue-Specificity of Gene Expression Diverges Slowly between Orthologs, and Rapidly between Paralogs. *PLoS Comput Biol* **12**, e1005274 (2016).
132 Somorjai, I., Bertrand, S., Camasses, A., Haguenauer, A. & Escriva, H. Evidence for stasis and not genetic piracy in developmental expression patterns of Branchiostoma lanceolatum and Branchiostoma floridae, two amphioxus species that have evolved independently over the course of 200 Myr. *Dev Genes Evol* **218**, 703-713 (2008).
133 Campbell, P. D. & Marlow, F. L. Temporal and tissue specific gene expression patterns of the zebrafish kinesin-1 heavy chain family, kif5s, during development. *Gene Expr Patterns* **13**, 271-279 (2013).
134 Fassier, C. *et al.* Zebrafish atlastin controls motility and spinal motor axon architecture via inhibition of the BMP pathway. *Nat Neurosci* **13**, 1380-1387 (2010).

SUPPLEMENTARY DATASETS

Supplementary Dataset 1 - Information about the CAGE-seq data generated in the current study for amphioxus and associated mapping statistics.

Supplementary Dataset 2 - Information about the ATAC-seq data generated in the current study for amphioxus, zebrafish and medaka and associated mapping statistics. Previously published data for vertebrates that were used in this study are also included.

Supplementary Dataset 3 - Information about the RNA-seq data generated in the current study for amphioxus, zebrafish and medaka and associated mapping statistics. Previously published data for vertebrates that were used in this study are also included.

Supplementary Dataset 4 - Information about the ChIP-seq data generated in the current study for amphioxus and associated mapping statistics.

Supplementary Dataset 5 - Information about the MethylC-seq and RRBS data generated in the current study for amphioxus and associated conversion statistics.

Supplementary Dataset 6 - Catalog of lncRNAs identified in the *B. lanceolatum* genome. Types: antisense, it overlaps with a protein-coding gene in the reverse strand; intragenic, it overlaps with a protein-coding gene in the same strand; bidirectional, within 1 kbp of a TSS of a protein-coding gene in the antisense strand, likely a product of a bidirectional promoter; intergenic, it does not overlap with any protein-coding gene.

Supplementary Dataset 7 - Complete list of gene families used in this study. Fam_ID_Final, final family identifier after re-clustering using Ensembl information; Fam_ID_Orig, original family identifier as obtained from the OMA analysis.

Supplementary Dataset 8 – Complementary information on sample size and P-values for various comparisons in the Main and Extended Data Figures.

Supplementary Dataset 9 - Lists of gene families categorized as having multiple ohnologs in vertebrates ("ohnologs"), having returned to single copy in vertebrates ("1-to-1"), *trans-dev* and housekeeping genes.

Supplementary Dataset 10 - The first table shows the predicted TFs and their predicted DNA binding motifs. The second table reports those predicted TFs for which no binding motif could be associated. The third table ("Final_motif_clusters") contains the full list of motif clusters used for all analyses, and the gene and gene families to which they were associated.

Supplementary Dataset 11 - List of DMRs at each transition (8hpf to 15hpf, 15hpf to 36hpf, 36hpf to hepatic diverticulum) in BED format.

Supplementary Dataset 12 - Enriched GO categories for each Mfuzz cluster for amphioxus and zebrafish and associated statistics, as provided by topGO. P-values correspond to uncorrected two-sided Fisher's exact tests. All cluster profiles are provided in Supplementary Fig. 3 and 4.

Supplementary Dataset 13 - Information about the 4C-seq data generated in the current study for amphioxus, zebrafish and mouse and associated mapping statistics.

Supplementary Dataset 14 - List with the selected reference transcript per gene for amphioxus used for the calculation of cRPKMs.

Supplementary Dataset 15 - Developmental pathway gene catalogs for zebrafish retrieved based on GO annotations retrieved from Ensembl Biomart. Characteristic genes from certain pathways that are annotated as members of other pathways due to pathway crosstalk were excluded from the analyses (column "Crosstalk").

Supplementary Dataset 16 - Clusters of CAGE-seq-derived promoters and associated information for amphioxus. "cluster" corresponds to the group in the self-organizing map from

Supplementary Fig. 6. "tata", "yy1", "ry" indicates whether or not the promoter contains these sequence motifs. Last columns correspond to the expression values of the promoters in the different samples, using the TPM metric.

Supplementary Dataset 17 - Z-score for each TF binding site motif ("Motif uID") across amphioxus and zebrafish WGCNA modules. Detailed information about each WGCNA module is provided in Supplementary File 1.

SUPPLEMENTARY TABLES

Supplementary Table 1 - Information about the staging of amphioxus embryos and the corresponding developmental time after fertilization at 19°C.

Supplementary Table 2 - Illumina sequencing libraries generated for this study, and coverage based on a estimated genome size of 550Mbp. All values are provided in bp.

Supplementary Table 3 - Selection of RNA-seq samples used for the identification of a chordate phylotypic period. Sample IDs correspond to the names provided in Supplementary Dataset 3. For those stages with several associated sample IDs, average expression values were calculated from those samples.

Supplementary Table 4 - Proteomes used to build the orthology relationships and gene families, with associated statistics.

Supplementary Table 5 - Selection of RNA-seq samples used for the Mfuzz clustering of amphioxus and zebrafish developmental stages. Sample IDs correspond to the names provided in Supplementary Dataset 3. For those stages with several associated sample IDs, expression values were calculated by pooling those samples.

Supplementary Table 6 - Selection of RNA-seq samples used for the WGCNA in amphioxus and zebrafish. Sample IDs correspond to the names provided in Supplementary Dataset 3. For those stages with several associated sample IDs, expression values were calculated by pooling those samples.

Supplementary Table 7 - Selection of RNA-seq samples used for the analyses on the fates of ohnologs in amphioxus, zebrafish, frog and mouse. Sample IDs correspond to the names provided in Supplementary Dataset 3. For those stages with several associated sample IDs, expression values were calculated by pooling those samples.

Supplementary Table 8 - Primer sequences used to clone the amphioxus APREs tested by transgenic assays in zebrafish, and information about each transgenic assay (number of F1 founders and categories of expression during development).

Supplementary Table 9 - Previously validated enhancers from amphioxus species obtained from the literature mapped to the *B. lanceolatum* genome. *Hox1A* and *Hox 1801* are overlapping enhancers and thus counted only once. References: Feng et al (2014) [44], Acemel et al. (2016) [27], Beaster-Jones et al. (2007) [86], Clarke et al. (2012) [25], Holland et al. (2008)[87], Hufton et al. (2009) [88], Irimia et al. (2012)[89], Kozmikova and Kozmik (2015) [90], Kozmikova et al. (2011) [91], Kozmikova et al. (2013) [92], Li et al. (2012) [93], Maeso et al. (2012) [94], Manzanares et al. (2000)[95], Ochi et al. (2012)[96], Punnamoottil et al. (2010) [97], Royo et al. (2011)[24], Van Otterloo et al. (2012) [98], Wada et al. (2006) [99], Yu et al. (2004) [100], Yue et al. (2016) [101].

Supplementary Table 10 - Coordinates of hepatic-specific APREs (i.e. ATAC-seq peaks), divided based on their 5mC content across embryonic stages and hepatic diverticulum. Cluster 1 APREs are those with differential 5mC levels whereas Cluster 2 APREs showed constitutive lack of 5mC.

Supplementary Table 11 - List of amphioxus APREs whose activation is associated with demethylation that are within introns that are conserved with zebrafish introns that contain DMRs. Components of the Hippo pathway are highlighted in green and genes likely part of multi-gene Genomic Regulatory Blocks (GRBs) are highlighted in blue.

Supplementary Table 12 - Experimentally determined regulatory landscapes using 4C-seq for 11 gene families in amphioxus (11 members), zebrafish (27 members) and mouse (20 members). The number of APREs identified by ATAC-seq at each stage within the regulatory landscape is indicated for each species. Stages used for Extended Data Fig. 9e are indicated by an asterisk.

Supplementary Table 13 - Assembly metrics at successive steps of the genome assembly of *B. lanceolatum.*

Supplementary Table 14 - Census of repetitive elements in the *B. lanceolatum* genome by family as identified by RepeatMasker.

Supplementary Table 15 - Number of 1-to-1 orthologs used for each pairwise comparison of developing transcriptomes.

Supplementary Table 16 - Selection of RNA-seq samples used for the NACC analysis comparing human with amphioxus, zebrafish and mouse. Sample IDs correspond to the names provided in Supplementary Dataset 3. For those stages with several associated sample IDs, expression values were calculated by pooling those samples.

Supplementary Table 17 - Selection of RNA-seq samples from vertebrate tissues and organs that have no clear homolog in amphioxus or have been highly elaborated in vertebrates. Sample IDs correspond to the names provided in Supplementary Dataset 3.

Supplementary Table 18 - Selection of RNA-seq samples used for the TAU analysis comparing amphioxus with zebrafish, frog and mouse. Sample IDs correspond to the names provided in Supplementary Dataset 3. For those stages with several associated sample IDs, expression values were calculated by pooling those samples.

Supplementary Table 19 - Sequence of probes used for *in situ* hybridization assays in amphioxus.

Supplementary File 1

This file contains the detailed annotation and expression patterns for each module of co-regulated genes identified by WGCNA in amphioxus and zebrafish. Section description:

1 - Amphioxus and zebrafish module annotation & comparisons. **a**, Name assigned to each module (color) based on gene expression and/or GO enrichment. **b**, Same heatmaps as in Fig. 4a,b including the names of each module.

2. Clustered heatmap of TF-motifs vs amphioxus & zebrafish modules. **a**, WGCNA modules from the two species are plotted against all motif clusters. The values visualized are the z-scores of each motif in each module. Modules and motifs are clustered based on the correlation of the visualized z-scores. **b**, Key for TF motif super-families in a, ordered by size of the motif name.

3. Amphioxus module RNA-seq expression and GO terms. For each amphioxus module, boxplots without whiskers showing the median and interquartile range of gene expression levels (using the cRPKM metrics) across RNA-seq samples (top), and significantly enriched GO categories (bottom). P-values correspond to uncorrected p-values from two-sided Fisher's exact tests as calculated by topGO. Number of genes per module is provided in Supplementary Dataset 8.

4. Zebrafish module RNA-seq expression and GO terms. For each zebrafish module, boxplots without whiskers showing the median and interquartile range of gene expression levels (using the cRPKM metrics) across RNA-seq samples (top), and significantly enriched GO categories (bottom). P-values correspond to uncorrected p-values from two-sided Fisher's exact tests as calculated by topGO. Number of genes per module is provided in Supplementary Dataset 8.

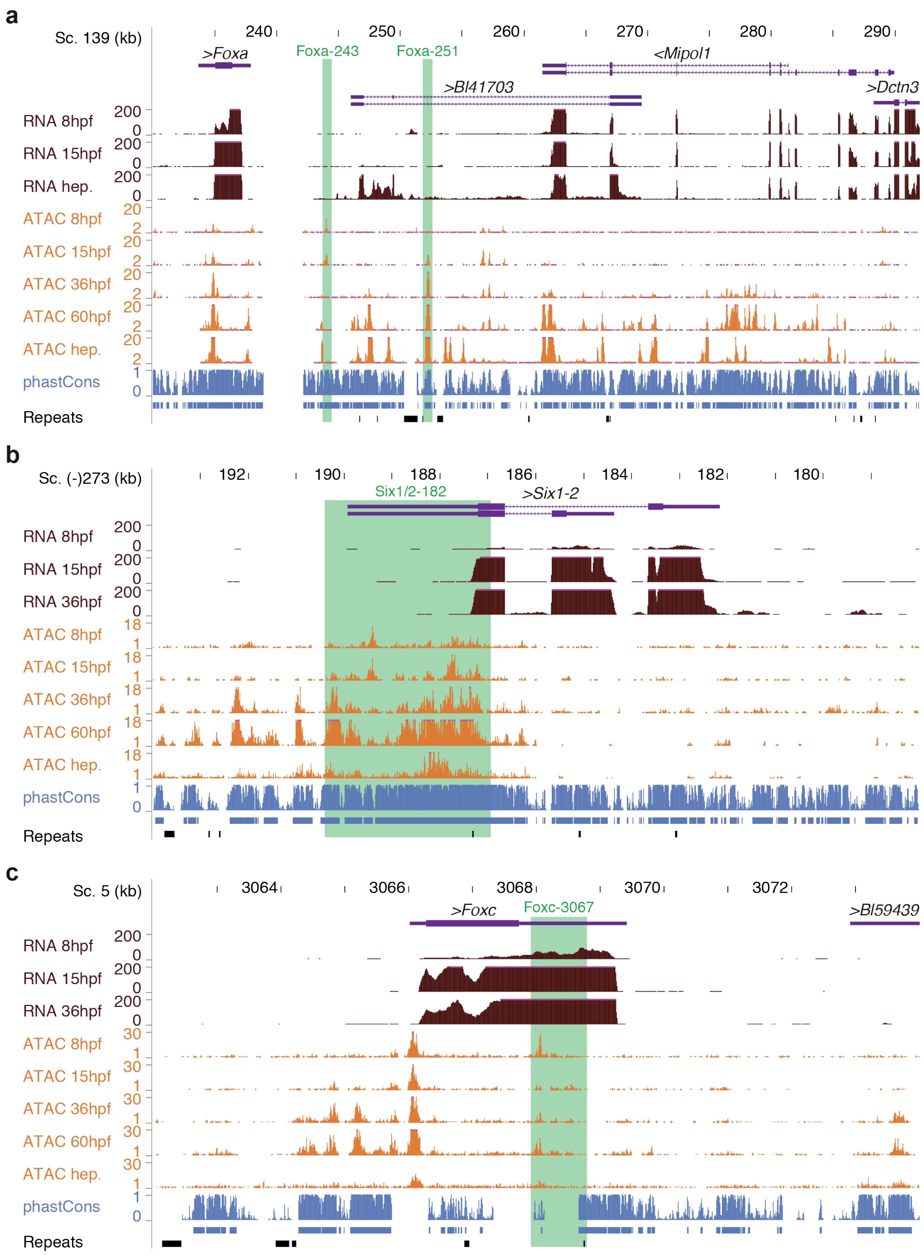

Supplementary Fig 1 - Browser captures for tested amphioxus enhancers

Browser captures showing the genomic contexts and putative target genes of the ATAC-seq peaks driving tissue-specific GFP expression shown in Extended Data Fig. 2h (Foxa-243, Foxa-251, Foxc-3067, Six1/2-182) and Extended Data Fig. 2i (Foxa-251, Foxc-3067). The genomic regions cloned in the reporter vectors are shaded with green rectangles. The genomic coordinates of these and the remaining tested ATAC-seq elements (Pax1/9-126, Pax1/9-157 and non-tissue specific elements) are listed in Supplementary Table 8. The number of biological replicates for each experiment displayed in the track is provided in Fig. 1a.

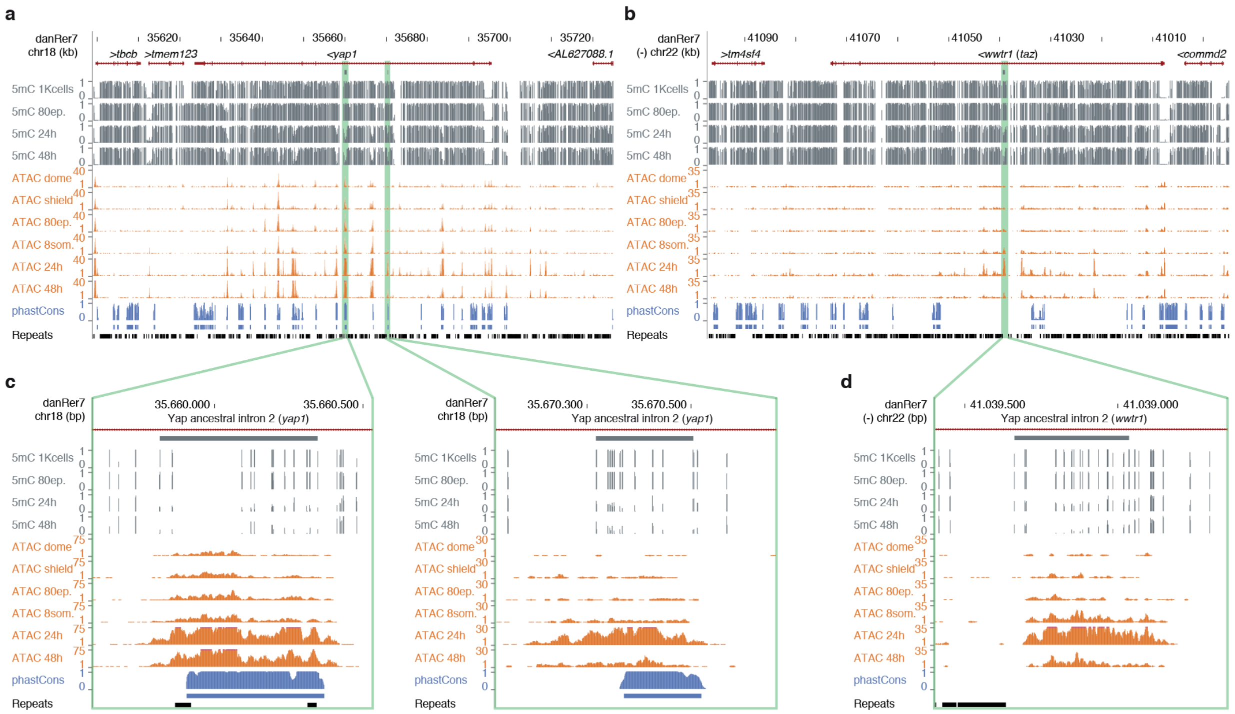

Supplementary Fig 2 - Browser captures for zebrafish DMRs

Genomic regions of two zebrafish orthologs (*yap1* [a] and *wwtr1* [b]) of *Yap*, one of the 14 gene families containing conserved introns with differentially methylated APREs in both zebrafish and amphioxus. Higher magnification views of each corresponding adult-specific demethylated enhancer are shown in c and d. The amphioxus ortholog is shown in Extended Data Figure 6f. The number of biological replicates for each experiment displayed in the track is provided in Fig. 1a.

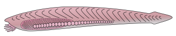

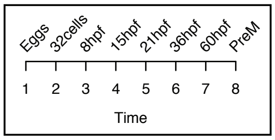

B. lanceolatum Mfuzz clusters

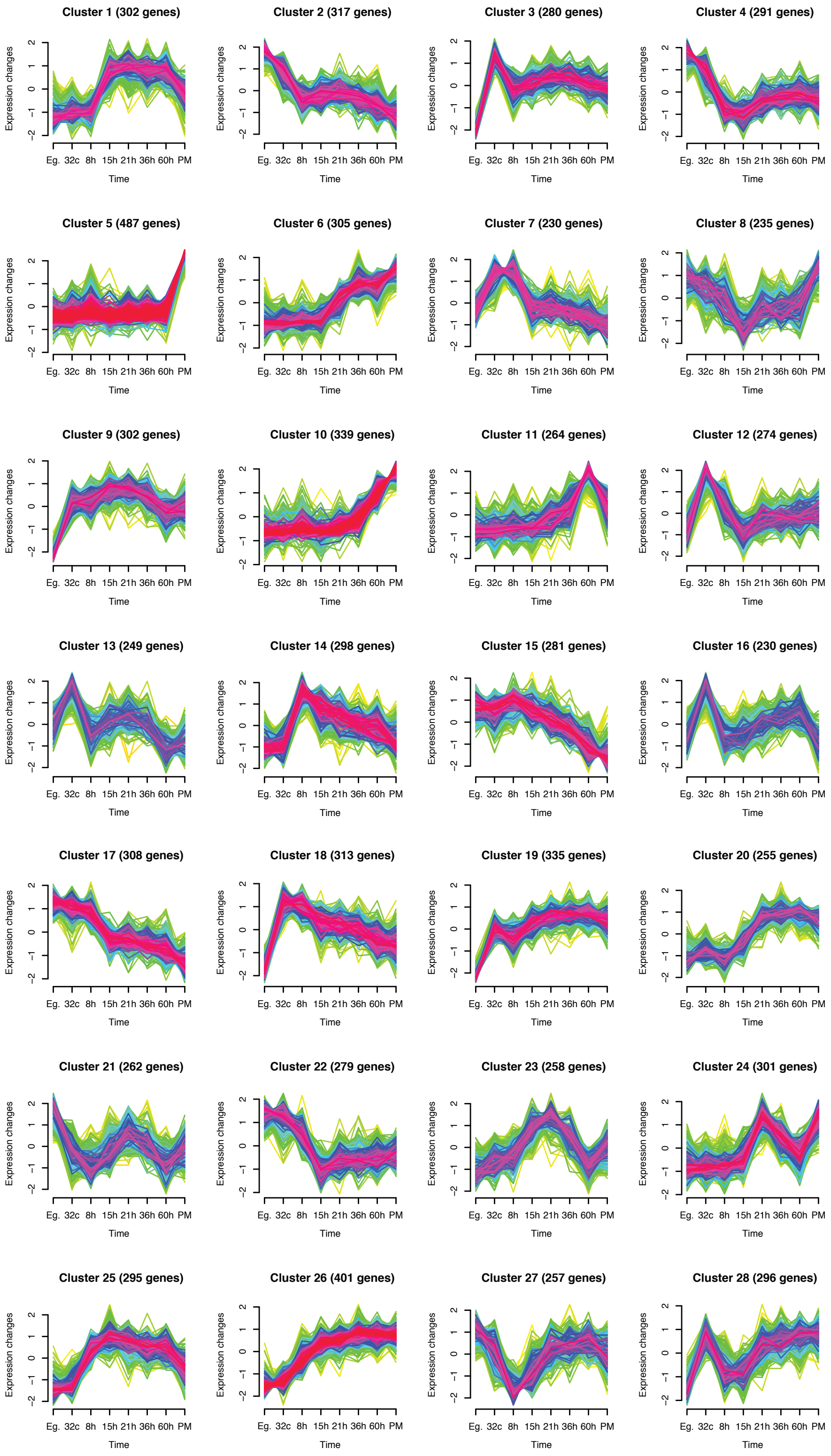

Supplementary Fig 3 – Mfuzz cluster profiles for amphioxus

Amphioxus gene clusters based on developmental expression dynamics as defined by Mfuzz. Eight matched developmental stages were chosen in amphioxus and zebrafish based on equivalent landmarks (fertilization, gastrulation, phylotypic stage, neurulation). The number of genes in each cluster is indicated in parenthesis. PM/PreM: pre-mouth larva.

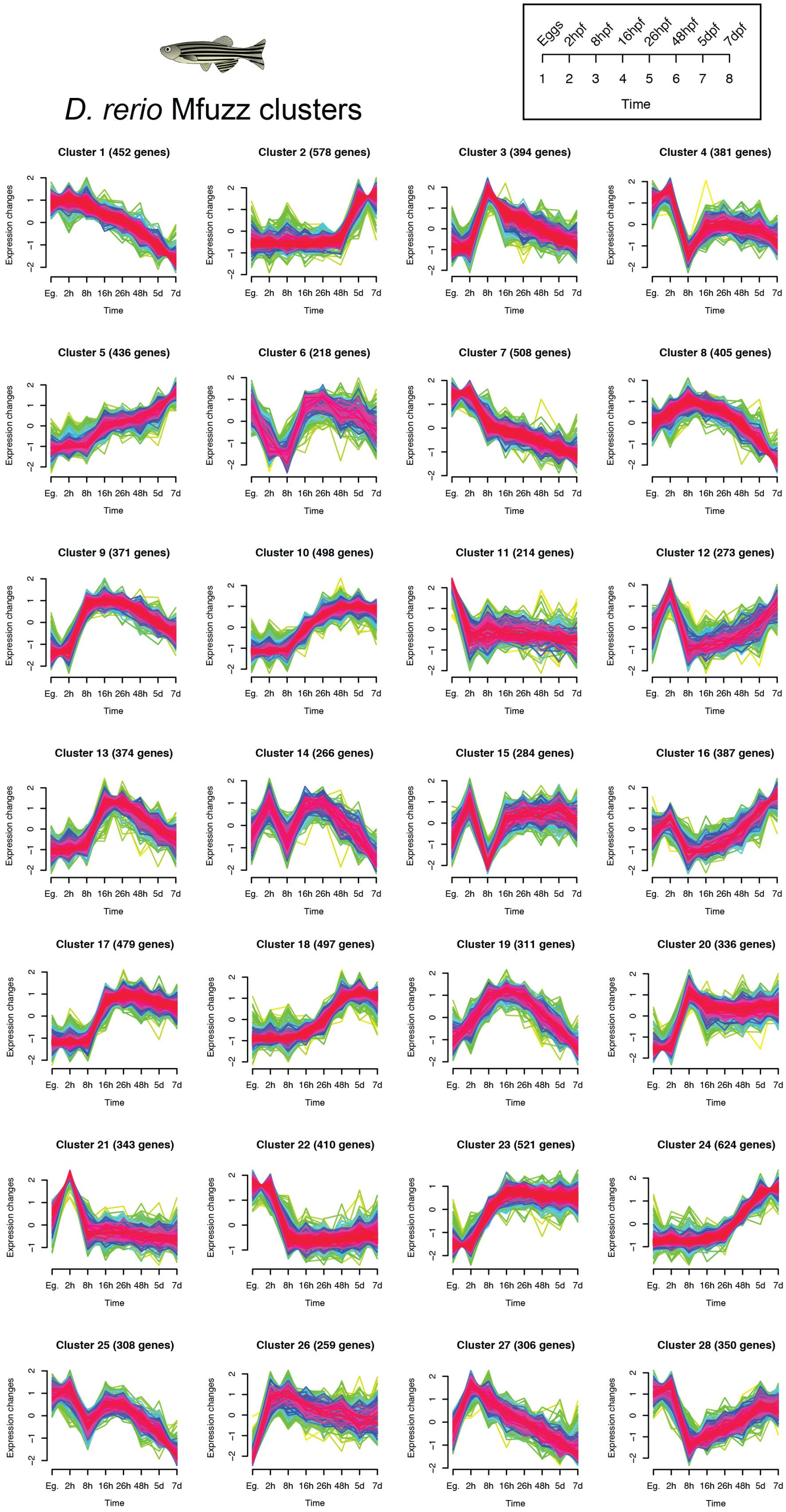
D. rerio Mfuzz clusters
Eggs 2hpf 8hpf 16hpf 26hpf 48hpf 5dpf 7dpf
1 2 3 4 5 6 7 8
Time
Cluster 1 (452 genes)
Cluster 2 (578 genes)
Cluster 3 (394 genes)
Cluster 4 (381 genes)
Cluster 5 (436 genes)
Cluster 6 (218 genes)
Cluster 7 (508 genes)
Cluster 8 (405 genes)
Cluster 9 (371 genes)
Cluster 10 (498 genes)
Cluster 11 (214 genes)
Cluster 12 (273 genes)
Cluster 13 (374 genes)
Cluster 14 (266 genes)
Cluster 15 (284 genes)
Cluster 16 (387 genes)
Cluster 17 (479 genes)
Cluster 18 (497 genes)
Cluster 19 (311 genes)
Cluster 20 (336 genes)
Cluster 21 (343 genes)
Cluster 22 (410 genes)
Cluster 23 (521 genes)
Cluster 24 (624 genes)
Cluster 25 (308 genes)
Cluster 26 (259 genes)
Cluster 27 (306 genes)
Cluster 28 (350 genes)
Expression changes
Eg. 2h 8h 16h 26h 48h 5d 7d
Time

Supplementary Fig 4 – Mfuzz cluster profiles for zebrafish

Zebrafish gene clusters based on developmental expression dynamics as defined by Mfuzz. Eight matched developmental stages were chosen in amphioxus and zebrafish based on equivalent landmarks (fertilization, gastrulation, phylotypic stage, neurulation). The number of genes in each cluster is indicated in parenthesis.

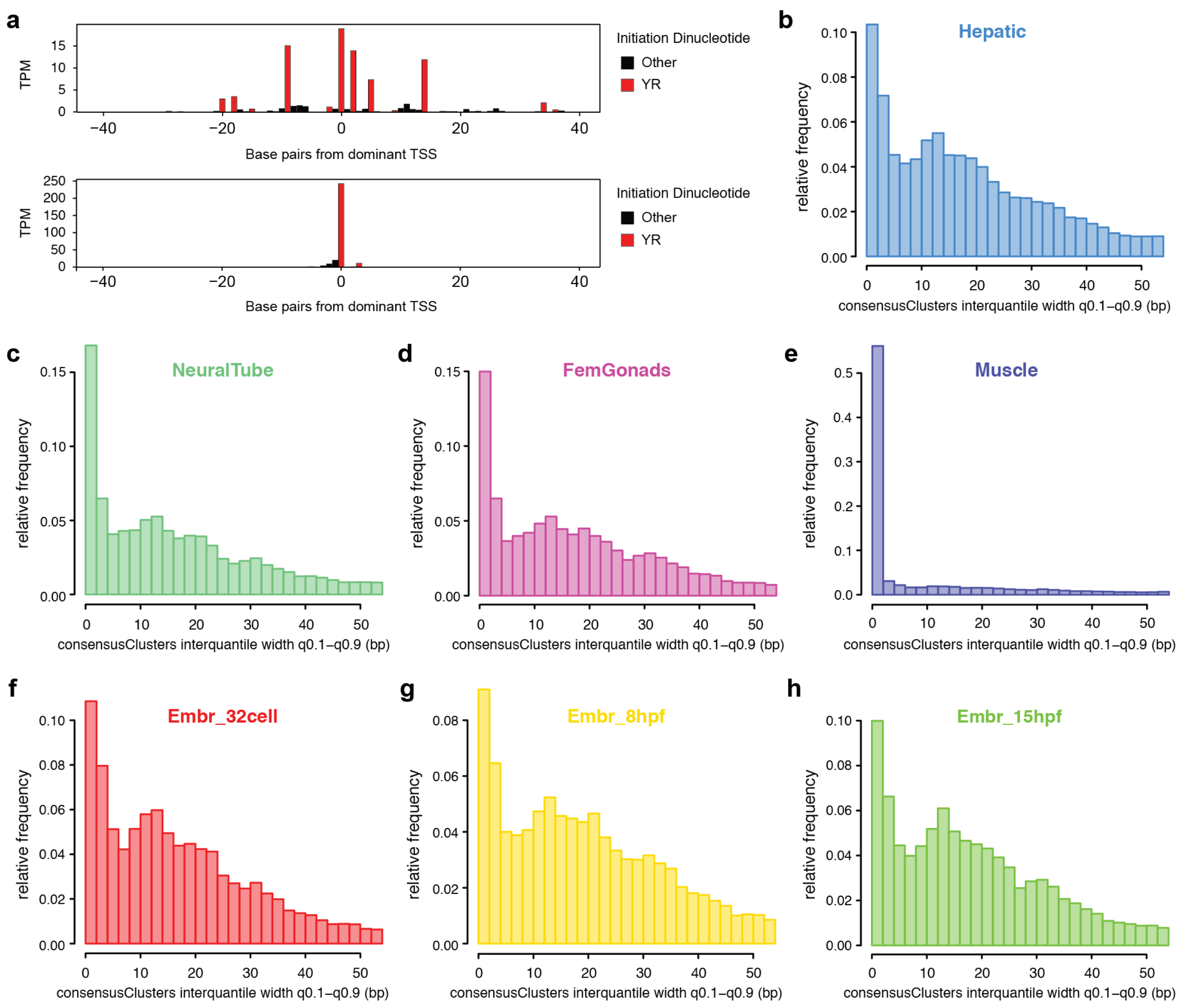

Supplementary Fig 5 – Distribution of tags per CAGE-seq derived promoter

a, Examples of a broad and sharp promoter. Top: CAGE-seq tags in embryo 15hpf from the region Sc0000001:7714823-7714902. Broad promoters initiate from multitude of sites over dozens of base pairs. Bottom: CAGE tags in embryo 15hpf from the region Sc0000240:29994-30073. Sharp promoters usually initiate predominantly from a single base pair. In both cases, the strongest initiation nucleotides are purines (R) that are immediately preceded by a pyrimidine (Y). **b-h**, CAGE Clusters show a bimodal distribution in width between “broad” and “sharp” promoters, which have characteristic patterns of initiation. Muscle sample (e) shows an unusually high number of single-tag promoters, consistent with the very low sequencing depth obtained from the corresponding library and was discarded from further analyses.

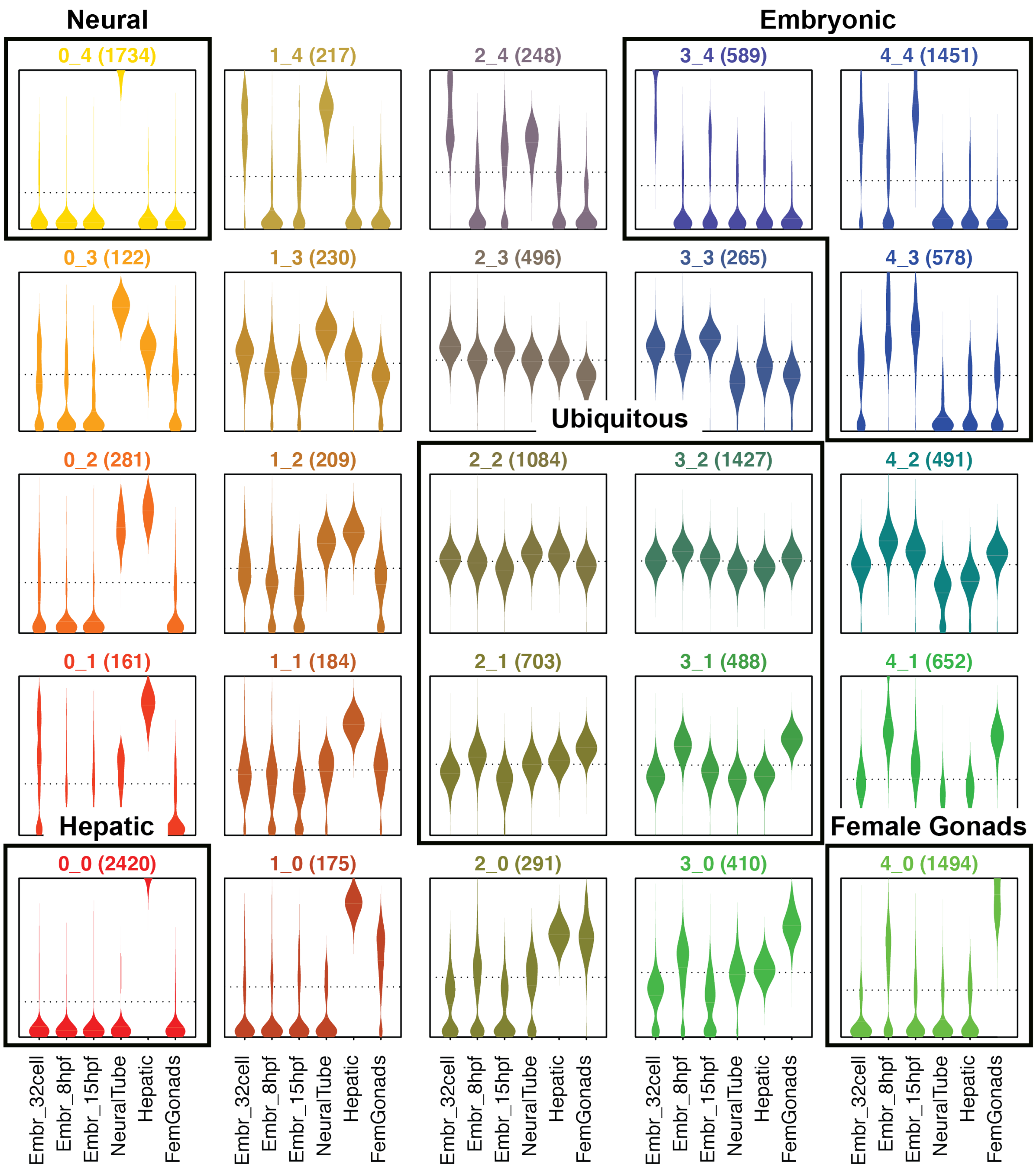

Supplementary Fig 6 – Self-organizing maps of consensus CTSS clusters

Consensus CAGE-seq derived clusters arranged in a self-organizing map (SOM). Each box represents one SOM cluster, with a series of bean-plots showing distribution of scaled expression (logarithm of normalized number of CAGE tags per million) in different samples for all promoters belonging to that SOM cluster (number of promoters denoted above the box, total

N=16,400). White lines within each bean-plot show the mean expression for each sample and SOM cluster. The dotted line represents the average expression level across samples for a given SOM cluster. The highlighted SOM clusters were used in downstream analyses.

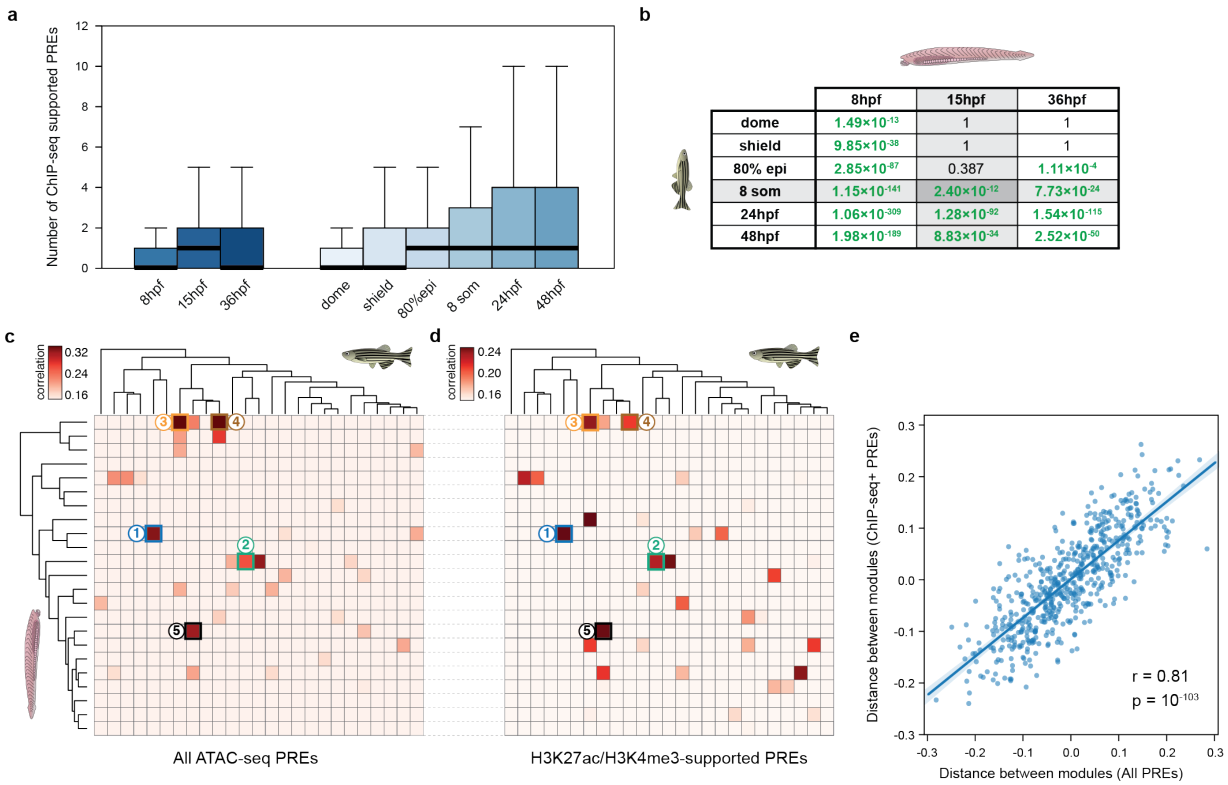

	8hpf	15hpf	36hpf
dome	1.49×10^{-13}	1	1
shield	9.85×10^{-38}	1	1
80% epi	2.85×10^{-87}	0.387	1.11×10^{-4}
8 som	1.15×10^{-141}	2.40×10^{-12}	7.73×10^{-24}
24hpf	1.06×10^{-309}	1.28×10^{-92}	1.54×10^{-115}
48hpf	1.98×10^{-189}	8.83×10^{-34}	2.52×10^{-50}

Supplementary Fig 7 – Analyses with APREs supported by H3K27ac or H3K4me3 data.

a, Distribution of the number of APREs with H3K27ac or H3K4me3 support within each gene's GREAT region at different developmental stages of zebrafish (n=9,239 genes) and amphioxus (n=6,047 genes). **b,** Comparisons between pairs of distributions of number of APREs with H3K27ac or H3K4me3 support from zebrafish and amphioxus stages (from a) using one-sided Mann-Whitney U tests. **c,d**, Heatmap of all pairwise Pearson correlations between the modules of the two species, based on the relative TF motif z-scores (across 242 motif super-families) for each module either in all (c, as in Fig. 4b) or H3K27ac/H3K4me3-supported APREs (d). Modules are ordered according to the clustering in Fig. 4a. **e,** Scattered plot of the distance between pairs of modules based on all (x-axis) and H3K27ac/H3K4me3-supported (y-axis) APREs.

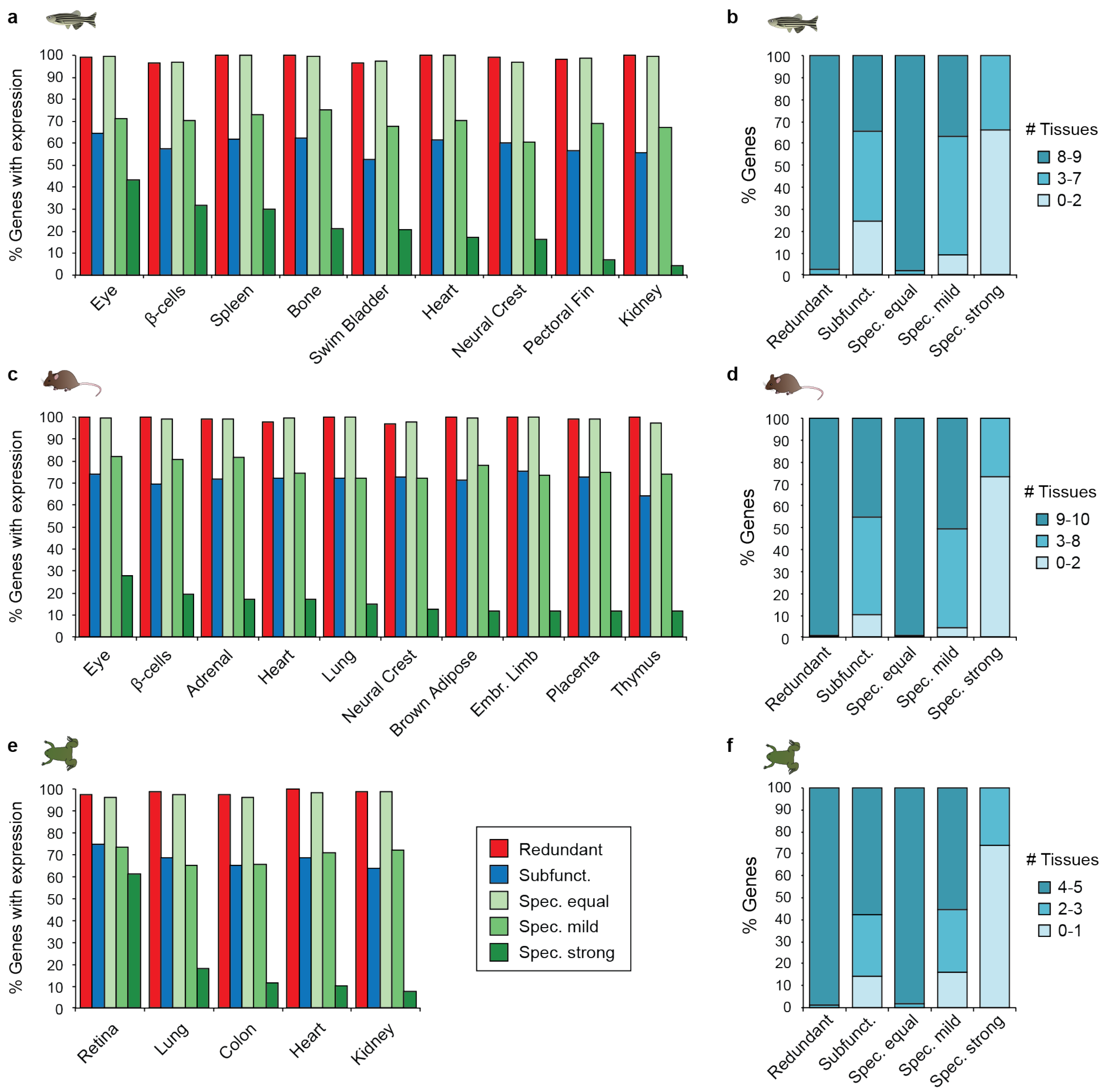

Supplementary Fig. 8 - Expression of different types of gene families

a,c,e, Percent of zebrafish (a), mouse (c) and frog (e) genes that have positive expression in the different studied tissues by type of gene family fate. **b,d,f**, Percent of zebrafish (b), mouse (d) and frog (f) genes that have positive expression in a given number of studied tissues by type of gene family fate.

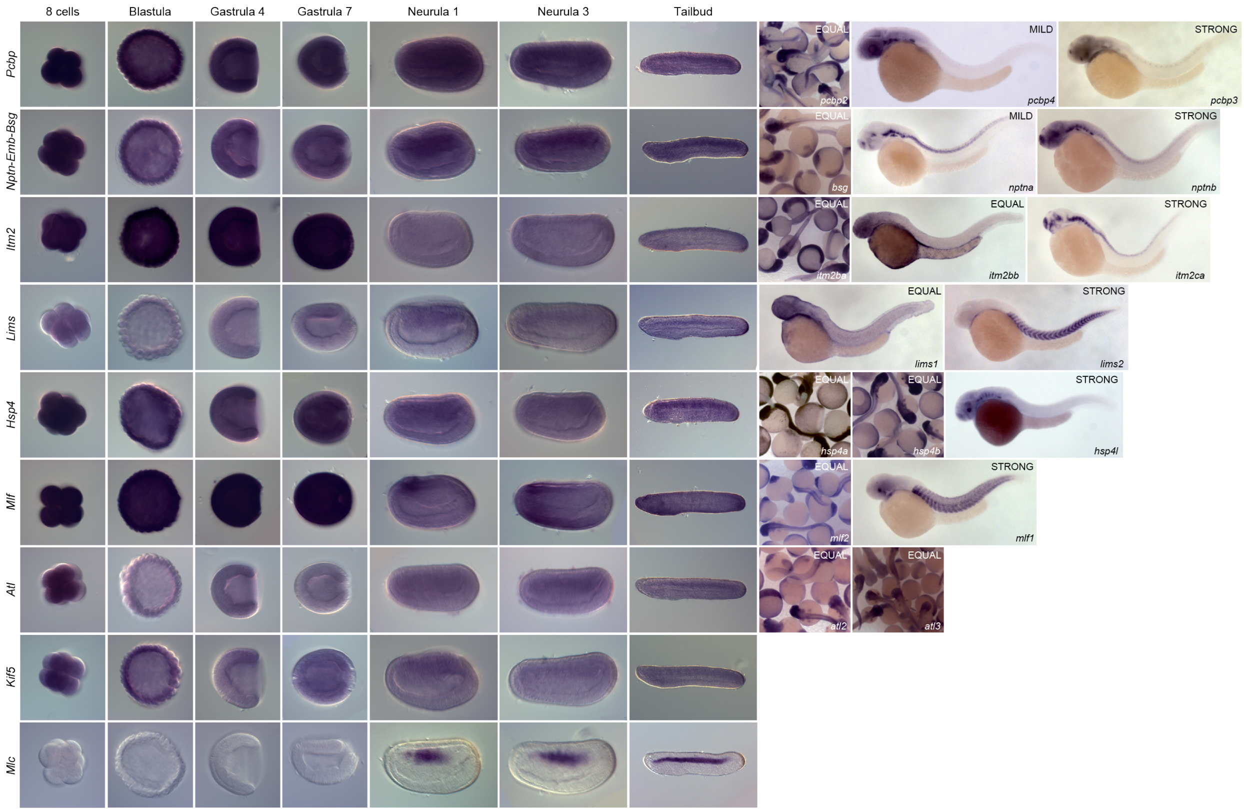

Supplementary Fig. 9 - *In situ* hybridization assays for specialized zebrafish families

In situ hybridization for multiple amphioxus stages for 11 genes whose zebrafish orthologous family has undergone specialization of at least one member. Only the positive control (*Mlc*, bottom) showed clearly restricted expression in amphioxus. The experiment was performed once, using 10 embryos per probe, all of which showed the same pattern for each distinct probe. *In situ* hybridization data for zebrafish was obtained from Zfin, with the exception of *Kif5* genes [133] and the *Atl* strongly specialized ohnolog *atl1* [134], which have been reported before and are not shown here.
