# Supplementary material for: Amphioxus functional genomics and the origins of vertebrate gene regulation
Source: Nature. 2018 Nov 21;564(7734):64–70. doi: 10.1038/s41586-018-0734-6 (PMC6292497; doi:10.1038/s41586-018-0734-6)
Supplement: Supplementary file 2 — Reporting Summary [file 41586_2018_734_MOESM2_ESM.pdf]

nature research

Corresponding author(s): Manuel Irimia, Jose Luis Gomez-Skarmeta, Hector Escriva, Ignacio Maeso

Reporting Summary

Nature Research wishes to improve the reproducibility of the work that we publish. This form provides structure for consistency and transparency in reporting. For further information on Nature Research policies, see Authors & Referees and the Editorial Policy Checklist.

Statistical parameters

When statistical analyses are reported, confirm that the following items are present in the relevant location (e.g. figure legend, table legend, main text, or Methods section).

n/a	Confirmed	
☐	☒	The exact sample size (n) for each experimental group/condition, given as a discrete number and unit of measurement
☒	☐	An indication of whether measurements were taken from distinct samples or whether the same sample was measured repeatedly
☐	☒	The statistical test(s) used AND whether they are one- or two-sided<br>*Only common tests should be described solely by name; describe more complex techniques in the Methods section.*
☒	☐	A description of all covariates tested
☐	☒	A description of any assumptions or corrections, such as tests of normality and adjustment for multiple comparisons
☐	☒	A full description of the statistics including central tendency (e.g. means) or other basic estimates (e.g. regression coefficient) AND variation (e.g. standard deviation) or associated estimates of uncertainty (e.g. confidence intervals)
☐	☒	For null hypothesis testing, the test statistic (e.g. F, t, r) with confidence intervals, effect sizes, degrees of freedom and P value noted<br>*Give P values as exact values whenever suitable.*
☒	☐	For Bayesian analysis, information on the choice of priors and Markov chain Monte Carlo settings
☒	☐	For hierarchical and complex designs, identification of the appropriate level for tests and full reporting of outcomes
☒	☐	Estimates of effect sizes (e.g. Cohen's d, Pearson's r), indicating how they were calculated
☐	☒	Clearly defined error bars<br>*State explicitly what error bars represent (e.g. SD, SE, CI)*

Our web collection on statistics for biologists may be useful.

Software and code

Policy information about availability of computer code

Data collection

Images of reporter assays in zebrafish embryos were collected using cell Sens Entry 1.6 from Olympus Corporation, amphioxus ISH images with QED Capture Version 2.0.33 and amphioxus reporter assays with LAS AF (Leica Application Suite Advanced Fluorescence, version 2.7.3.). CAGE data from the FANTOM repository were obtained with CAGEr. Zebrafish CAGE-seq data were obtained through the ZebrafishDevelopmentalCAGE R package available at http://promshift.genereg.net/CAGEr/.

Data analysis

Custom code is available at: https://gitlab.com/FunctionalAmphioxus

The following packages and software were used:

1. FastXend, based on fastx v0.0.13.1 (https://github.com/institut-de-genomique/fastxtend)
2. SOAP de novo (v2.04, release 13 Jul 2012)
3. Bowtie2 (v 2.2.6)
4. Bowtie (v1.1.2)
5. MACS2 (v2.1.1.20160309)
6. peakC (v0.2)
7. Jellyfish (v2.2.0)
8. Musket (v1.0.6)
9. Flash (v1.2.1)
10. Gapcloser (v1.12)

11. Haplomerger pipeline (release 20111230)
12. Lastz (v1.02.00 release 20100112)
13. CEGMA (v2.4, rel Nov 2006)
14. EVidence Modeller, EVM (r2012-06-25)
15. TransDecoder (v2.0.1)
16. AUGUSTUS (v2.5.5)
17. Exonerate (v2.2.0)
18. Trinity (v2.2.0)
19. PASA (v2.0.2)
20. Tophat2 (v2.1.1)
21. Cufflinks (v2.2.1)
22. Cuffmerge (v1.0.0)
23. CPAT software (v1.2.1)
24. InterProScan (v5.10-50)
25. TopGO (R package version 2.29.0, R version: 3.2.4)
26. RepeatScout (v1.0.5)
27. RepeatModeler (v1-0-8)
28. RepeatMasker (open-4.0.5) (http://www.repeatmasker.org)
29. UCSC Kent utilities (downloaded Oct-22-2013): axtChain, chainMergeSort, chainPreNet, chainNet, multiz-tba (version 2009-Jan-21)
30. PhastCons (v1.3)
31. OMA (v1.0.5)
32. RAxML (v8.2.1)
33. ETE 3 python library (v3.0.0b36)
34. MAFFT (v7.2.2.2)
35. idr (v2.0.3)
36. Kallisto (v0.42.4)
37. preprocessCore package from Bioconductor (v1.40.0)
38. Mfuzz (Program version: v2.40.0; R version: 3.2.4)
39. Weighted Gene Correlation network analysis (WGCNA) (Program version: v1.62; R version 3.3.2)
40. CAGEr (Bioconductor v1.18.0)
41. heatmaps package, Bioconductor (v.1.4.0)
42. NucleoATAC (v0.3.2)
43. Bedtools (v2.26.0)
44. deepTools (v2.4)
45. DiffBind (v2.2.3; R version: 3.3.1)
46. WALT (v1.0) (https://github.com/smithlabcode/walt)
47. MethylDackel (v0.3) (https://github.com/dpryan79/MethylDackel)
48. HMMER (v3.1b)
49. GimmeMotifs (v0.11.1)
50. Matt v.1.2.0 (http://matt.crg.eu/)
51. ImageJ Fiji 64
52. jupyter-notebooks (v5.1.0)
53. Photoshop CS5.1
54. Inkscape (v0.92.1 r15371)
55. Illustrator CS5.1
56. Python (v2.7)
57. Python (v3.5)
58. Python libraries: numpy (1.13.3), scipy (1.0.0), pandas (0.19.2), sklearn (0.18.1), pybedtools(0.7.10), pysam (0.12.0.1), matplotlib (2.1.0), seaborn (0.8.1)
59. GNU/Linux Command-Line Tools: zcat (1.5) , awk (4.0.2), GNU coreutils 8.22 (shuf,cat,sort,uniq,wc,head,tail)
60. Blast (v2.2.29)
61. perl (v5.14.12)
62. Pfam database (v30.0)
63. vioplot (v0.2; R version: 3.2.4)
64. alluvial (v0.1-2; R version: 3.4.2)
65. methylpy (https://bitbucket.org/schultzmattd/methylpy.git)
66. fastp (v0.12.5)
67. samtools (v1.6)
68. kentUtils (v302.1)
69. ggplot2 (v3.0.0; R version: 3.5.0)

For manuscripts utilizing custom algorithms or software that are central to the research but not yet described in published literature, software must be made available to editors/reviewers upon request. We strongly encourage code deposition in a community repository (e.g. GitHub). See the Nature Research guidelines for submitting code & software for further information.

Data

Policy information about availability of data

All manuscripts must include a data availability statement. This statement should provide the following information, where applicable:

- Accession codes, unique identifiers, or web links for publicly available datasets
- A list of figures that have associated raw data
- A description of any restrictions on data availability

Next generation sequencing data have been deposited in Gene Expression Omnibus (GEO) under the following accession numbers: GSE106372 (ChIP-seq),

GSE106428 (ATAC-seq), GSE106429 (CAGE-seq), GSE106430 (RNA-seq), GSE102144 (MethylC-seq and RRBS), and GSE115945 (4C-seq). Raw genome sequencing data and genome assembly have been submitted to European Nucleotide Archive (ENA) under the accession number PRJEB13665.

Field-specific reporting

Please select the best fit for your research. If you are not sure, read the appropriate sections before making your selection.

☒ Life sciences ☐ Behavioural & social sciences ☐ Ecological, evolutionary & environmental sciences

For a reference copy of the document with all sections, see nature.com/authors/policies/ReportingSummary-flat.pdf

Life sciences study design

All studies must disclose on these points even when the disclosure is negative.

Sample size	No statistical method was used to predetermine sample size. For each cross-species transcriptomic and epigenomic comparisons as many orthologous genes as possible were used. Samples sizes for each analysis in the figures are indicated in the legends, Supplementary Dataset 8 and/or Supplementary Information. For each next generation sequencing experiment, for each of the biological replicate we used as many embryos or amount of adult tissues as necessary to obtain the enough amount of RNA/DNA for library preparation and sequencing, according to previous experience in the lab and previous publications (PMIDs: 26829752, 26928226, 23624103). These numbers are indicated in the corresponding sections of the Supplementary Information.
Data exclusions	For the CAGE-seq analysis, the muscle sample was excluded, as it did not fulfill the standard quality checks. Exclusion criteria for CAGE data are not predetermined, however it is long established that CAGE data has a characteristic variation in widths (e.g. Carninci et al. 2006, Nature Genetics 38 (6): 626–35), and this, along with the very low number of reads recovered (Supplementary Dataset 1) was the rationale for exclusion, as explained in Section 7.1 of Supplementary Information.
Replication	Nearly all the findings reported in this study correspond to computational analyses of next generation sequencing data. We provide the code and guidelines to reproduce all the analyses (https://gitlab.com/FunctionalAmphioxus). We also perform two main types of experiments, largely for validation purposes: (i) generation of transgenic assays and (ii) in situ hybridization of specialized families. For (i), we provide the number of independent founders identified for each tested element and a description of the patterns obtained for each founder in Supplementary Table 8. For (ii), we have performed the in situ hybridization only once, using ten embryos for probe, and all of them showed the same pattern.
Randomization	We did not have experimental groups that apply here. In our study we compared either (i) different tissues and developmental stages within a species, or (ii) matched samples for different species.
Blinding	Blinding was not relevant to our study since we did not have experimental groups to compare.

Reporting for specific materials, systems and methods

Materials & experimental systems

n/a	Involved in the study
☒	☐ Unique biological materials
☐	☒ Antibodies
☒	☐ Eukaryotic cell lines
☒	☐ Palaeontology
☐	☒ Animals and other organisms
☒	☐ Human research participants

Methods

n/a	Involved in the study
☐	☒ ChIP-seq
☒	☐ Flow cytometry
☒	☐ MRI-based neuroimaging

Antibodies

Antibodies used	-Rabbit polyclonal to Histone H3 (tri methyl K4) - ChIP Grade (#ab8580, Abcam), 1:200<br>-Rabbit polyclonal to Histone H3 (acetyl K27) - ChIP Grade (#ab4729, Abcam), 1:200<br>-Mouse monoclonal to Histone H3 (tri methyl K27) - ChIP Grade (#ab6002, Abcam), 1:200
Validation	The three primary antibodies used are all high-quality commercial antibodies against Histone H3 modifications, validated as ChIP grade by the manufacturer (Abcam):<br>https://www.abcam.com/histone-h3-tri-methyl-k4-antibody-chip-grade-ab8580.html<br>https://www.abcam.com/histone-h3-acetyl-k27-antibody-chip-grade-ab4729.html<br>https://www.abcam.com/histone-h3-tri-methyl-k27-antibody-mabcam-6002-chip-grade-ab6002.html

the Histone Antibody Specificity Database (http://www.histoneantibodies.com, PMID : 26212453) and the Antibody Validation Database (http://compbio.med.harvard.edu/antibodies/). Furthermore, these antibodies have been validated by ChIP-seq in previous publications in a wide range of animal species, all of which have 100% amino acid identity in their Histone 3 sequences (PMIDs: 24642862, 22593555, 22196729).

Animals and other organisms

Policy information about studies involving animals; ARRIVE guidelines recommended for reporting animal research

Laboratory animals

For zebrafish and medaka data, embryos were obtained from crosses of adult animals of the AB and Cab strains, respectively. Gender was not determined, since we have used pools of embryos.

Wild animals

The study did not involve animals in the wild.

Field-collected samples

Most of the next-generation sequencing data generated in this study is from the amphioxus Branchiostoma lanceolatum, a marine, non-cephalopod invertebrate. As indicated in the Methods section, adult specimens were collected from the wild at the Racou beach near Argelès-sur-Mer, France, (latitude 42° 32’ 53” N and longitude 3° 03’ 27” E) with a specific permission delivered by the Prefect of Region Provence Alpes Côte d’Azur. Branchiostoma lanceolatum is not a protected species. Gametes are collected by heat stimulation. Embryos are collected from in vitro fertilization after induced spawning, and often batches of several dozens or hundreds of mixed embryos are used for the experiments (detailed in Methods). Adult tissues are collected from several individuals of both genders, except for male and female gonads.

ChIP-seq

Data deposition

☒ Confirm that both raw and final processed data have been deposited in a public database such as GEO.

☒ Confirm that you have deposited or provided access to graph files (e.g. BED files) for the called peaks.

Data access links
May remain private before publication.

https://www.ncbi.nlm.nih.gov/geo/query/acc.cgi?acc=GSE106372

Files in database submission

GSM2836695 ChIPseq amphioxus H3K27ac 15h replicate 1 Oct 31, 2020 approved BED
GSM2836696 ChIPseq amphioxus H3K27ac 15h replicate 2 Oct 31, 2020 approved BED
GSM2836697 ChIPseq amphioxus H3K27ac 36h replicate 1 Oct 31, 2020 approved BED
GSM2836698 ChIPseq amphioxus H3K27ac 36h replicate 2 Oct 31, 2020 approved BED
GSM2836699 ChIPseq amphioxus H3K27ac 8h replicate 1 Oct 31, 2020 approved BED
GSM2836700 ChIPseq amphioxus H3K27ac 8h replicate 2 Oct 31, 2020 approved BED
GSM2836701 ChIPseq amphioxus H3K27me3 15h replicate 1 Oct 31, 2020 approved BED
GSM2836702 ChIPseq amphioxus H3K27me3 15h replicate 2 Oct 31, 2020 approved BED
GSM2836703 ChIPseq amphioxus H3K27me3 36h replicate 1 Oct 31, 2020 approved BED
GSM2836704 ChIPseq amphioxus H3K27me3 36h replicate 2 Oct 31, 2020 approved BED
GSM2836705 ChIPseq amphioxus H3K27me3 8h replicate 1 Oct 31, 2020 approved BED
GSM2836706 ChIPseq amphioxus H3K4me3 15h replicate 1 Oct 31, 2020 approved BED
GSM2836707 ChIPseq amphioxus H3K4me3 15h replicate 2 Oct 31, 2020 approved BED
GSM2836708 ChIPseq amphioxus H3K4me3 36h replicate 1 Oct 31, 2020 approved BED
GSM2836709 ChIPseq amphioxus H3K4me3 36h replicate 2 Oct 31, 2020 approved BED
GSM2836710 ChIPseq amphioxus H3K4me3 8h replicate 1 Oct 31, 2020 approved BED
GSM2836711 ChIPseq amphioxus H3K4me3 8h replicate 2 Oct 31, 2020 approved BED

Genome browser session
(e.g. UCSC)

https://genome-asia.ucsc.edu/cgi-bin/hgTracks?db=hub_78274_BraLan2

Methodology

Replicates

As described in Fig 1A, all samples were done in duplicates, with the exception of H3K27me3 for 8hpf, for which only one library could be constructed.

Sequencing depth

H3K27ac_8hpf_a 8 hpf 49 SE 18,380,227
H3K27ac_8hpf_b 8 hpf 49 SE 47,674,217
H3K27ac_15hpf_a 15 hpf 49 SE 11,788,559
H3K27ac_15hpf_b 15 hpf 49 SE 15,294,563
H3K27ac_36hpf_a 36 hpf 49 SE 42,320,156
H3K27ac_36hpf_b 36 hpf 49 SE 35,867,629
H3K27me3_8hpf_a 8 hpf 49 SE 11,896,658
H3K27me3_15hpf_a 15 hpf 49 SE 40,188,161
H3K27me3_15hpf_b 15 hpf 49 SE 39,334,807
H3K27me3_36hpf_a 36 hpf 49 SE 11,969,177
H3K27me3_36hpf_b 36 hpf 49 SE 12,496,630
H3K4me3_8hpf_a 8 hpf 49 SE 53,565,742
H3K4me3_8hpf_b 8 hpf 49 SE 23,484,131
H3K4me3_15hpf_a 15 hpf 49 SE 11,831,374

H3K4me3_15hpf_b 15 hpf 49 SE 58,501,168
H3K4me3_36hpf_a 36 hpf 49 SE 33,948,863
H3K4me3_36hpf_b 36 hpf 49 SE 32,457,194

Antibodies

-Rabbit polyclonal to Histone H3 (tri methyl K4) - ChIP Grade (#ab8580, Abcam)
-Rabbit polyclonal to Histone H3 (acetyl K27) - ChIP Grade (#ab4729, Abcam)
-Mouse monoclonal to Histone H3 (tri methyl K27) - ChIP Grade (#ab6002, Abcam)

Peak calling parameters

Reads were mapped against the amphioxus reference genome using Bowtie, and peaks were called using the MACS2 software with default parameters.

Data quality

Chip-seq peaks were only used to overlap with the ATAC-seq peaks in multiple cross-validation analyses. Since we used MACS2 by default, all peaks were below FDR 5%.

Software

Reads were mapped against the amphioxus reference genome using Bowtie, and peaks were called using the MACS2 software with default parameters. The overlap between ATAC-seq and ChIP-seq peak was calculated using Bedtools.
