# Supplementary material for: Amphioxus functional genomics and the origins of vertebrate gene regulation
Source: Nature. 2018 Nov 21;564(7734):64–70. doi: 10.1038/s41586-018-0734-6 (PMC6292497; doi:10.1038/s41586-018-0734-6)

WGCNA module annotation

1. Amphioxus and zebrafish module annotation & comparisons...................page 2
    a. Module color & annotated names
    b. Amphioxus & zebrafish module comparisons (genes and TF-motifs, as in Fig.4)

2. Clustered heatmap of TF-motifs vs amphioxus & zebrafish modules........page 3
    a. WGCNA modules from the two species are plotted against all motif clusters. The values visualized are the z-scores of each motif in each module. Modules and motifs are clustered based on the correlation of the visualized z-scores
    b. TF motif clusters and their corresponding associated gene symbols. Clusters are sorted first by number of associated genes and then by their ID number

3. Amphioxus module RNA-seq expression and GO terms...........................page 4

4. Zebrafish module RNA-seq expression and GO terms.............................page 29

a

B. lanceolatum **WGCNA modules annotation**

Module Color	Module annotated name
black	32 cells
blue	Neural tube - synapsis
brown	Gills
cyan	Neural tube - neurogenesis
darkmagenta	Ovary / Testis - translation
darkorange	Cilium
darkred	Muscle
darkseagreen4	Embryo 8-36h / Skin / Cirri - lipid membranes
darkslateblue	Embryo 8h - transcription, splicing
darkturquoise	Embryo 36h
green	Eggs / 32 cells -cell cycle
greenyellow	Hepatic - lipid metabolism
lavanderblush3	Cirri / PreMet. / Muscle - actomyosin
lightpink4	Embryo 15h
magenta	Skin
navajowhite2	Immune
palevioletred3	Ovary / Eggs
pink	Gut
plum1	Gut / Hepatic
plum2	Proteasome
red	PreMet. larvae
salmon	Cirri - lipid membranes
sienna3	Hepatic
thistle2	Gills / PreMet. larvae
turquoise	Mitochondrion

D. rerio **WGCNA modules annotation**

Module Color	Module annotated name
bisque4	Larvae 7d - melanin
black	Ovary / Sperm
blue	Brain
brown	Cilium
brown4	Liver B
coral2	Mitochondrion
darkgreen	Intestine
darkgrey	RNA, ribosome, proteasome
darkmagenta	Translation
darkorange	Muscle
darkred	Liver A
darkseagreen4	Embryo 12-26h / Skin
darkslateblue	Immune
green	Splicing
honeydew1	Embryo 12-26h
ivory	Eye
lightcyan	Heart
lightgreen	Pancreas / Testis
magenta	Gills
pink	Skin
salmon	Embryo 20-26h
yellow4	Kidney
yellowgreen	Gills early

b

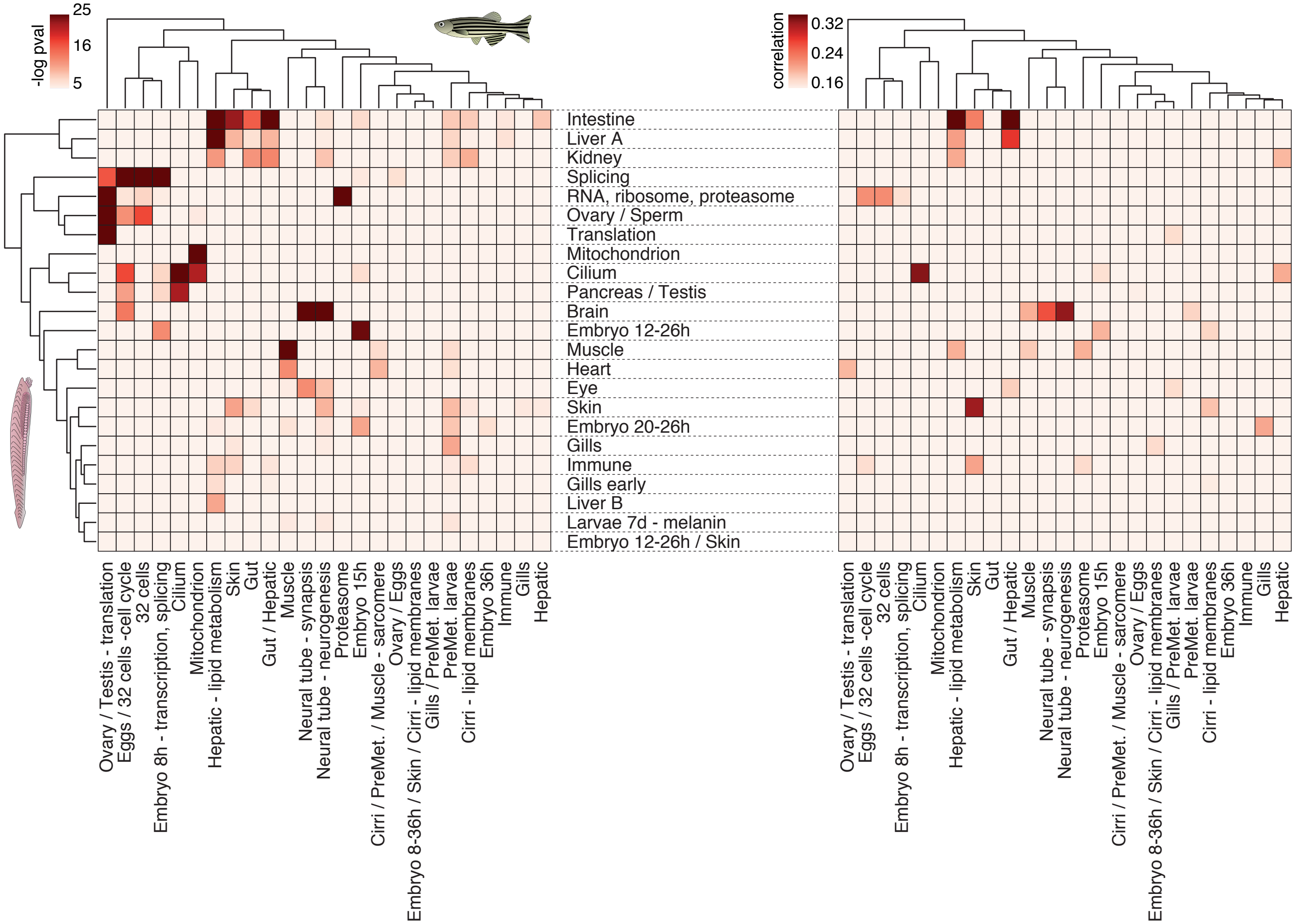

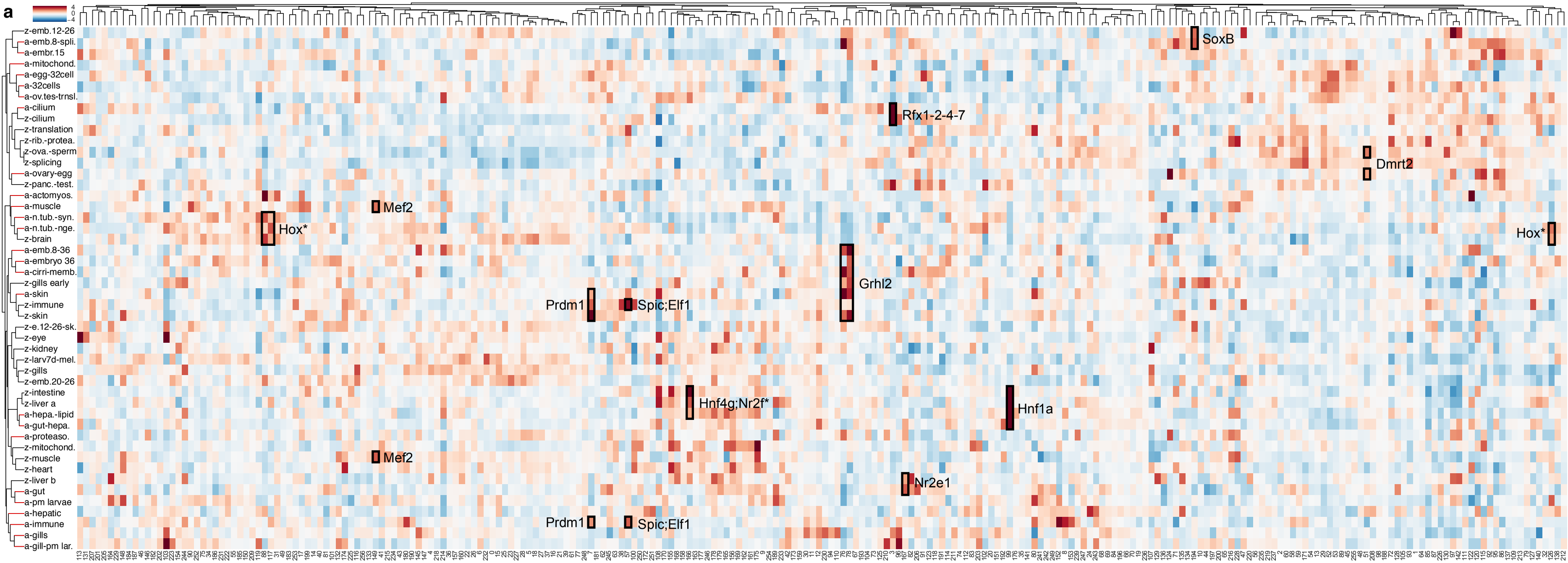

b

0 Tfap2e
1 Arid3b
2 Arid5b
4 Zbtb8a
5 Zfp740
7 Prdm1
8 Osr2
9 Fezf1
10 Ikzf4
12 Scrt2
13 Zfp523
16 Egr1
17 Gfi1b
18 Bcl6b
19 Ctcf
20 Klf11
21 Zbtb21
22 Rreb1
24 Hlf
28 Plag1
29 Yy1
30 Zeb2
31 Zbtb1
32 snai1a
33 Sp2
36 Zic1
38 Bcl11a
40 Gli3
41 Prdm4
43 Hivep1
44 Zfp362
45 Nfya
46 Ybx3
47 Rbpj
48 Onecut2
49 Cux2
51 Dmrt2
52 Tfdp2
54 E2f4
55 E2f3
56 Spdef
62 Elf5
64 Foxp1
65 Foxo1
70 Foxm1
71 Foxh1
75 Gcm2
77 Tfcp2
78 Grhl2
80 Hsf1
81 Lhx9
82 Emx2
84 Irx3
85 Pou6f1
89 Pbx1
91 Barhl2
99 Hnf1a
105 Cdx1
108 Lhx8
112 En1
121 Hlx
123 Lbx1
124 Meox2
125 Hoxb13
129 Pknox1
130 Prop1
132 Tgif1
133 Tlx1
138 Pou4f1
140 Pou1f1
141 Pax3
146 Pax2
148 Srf
149 Mef2d
150 Mecp2
151 Mybl1
152 Cdc5l
153 Smarcc2
154 Myrfl
155 Nr6a1
158 Thrb
167 Nr2e1
170 Nr1i2
171 Nr2c1
172 Rxrb
173 Nr2e3
175 Esr1
176 Hnf4g
177 Nr4a3
178 Nr1h4
179 Rarb
180 Nfkb2
181 Nfatc3
182 Runx1
186 Smad4
187 Smad2
188 Stat5b
190 Stat4
193 Tcf7l2
198 Hbp1
199 Cic
200 Sox11
205 Tbx19
207 Tbx2
208 Tbpl2
209 Prkrir
210 Twist2
212 Srebf1
217 Myf5
230 Tcf3
231 Bhlhe41
232 Npas1
233 Hand1
235 Max
247 Creb3l1
248 Atf4
249 Jund
252 Crem
253 Bach1
254 Mafa
256 Trp73
6 Zxdc;Glis3
23 Zic1;zic6
25 Zfp740;znf740b
26 Zbtb21;Gli3
42 Zbtb8a;Zfp652
53 E2f4;E2f3
61 Spic;spic
76 Grhl2;Tfcp2
79 Hsf1;hsf4
86 Lhx9;Meox2
88 Hoxb2;Hoxa1
100 En1;Gbx1
111 Hoxa6;Hoxa7
114 Hoxa10;Hoxa11
116 Hmx3;hmx4
118 six5;six4b
119 Nkx2-6;Nkx2-5
120 Evx2;Isx
137 Pou3f2;Pou2f1
139 Pou3f2;pou5f3
147 Pax2;Pax9
159 Ar;pgr
160 Hnf4g;Rxrb
161 Nr5a1;nr5a5
162 Nr4a3;Thrb
183 Nfia;nfixa
185 Smad2;Smad5
189 Stat4;Stat5b
194 Sox3;Sox21
201 Tbx1;Tbx20
202 Eomes;tbx21
215 Tal2;tal2
218 Neurod6;Atoh7
220 Tcf3;Hand1
225 Tcf15;Tcf3
227 Arnt2;Arntl
228 Bhlhe23;Olig3
229 ahr1b;Ahr
234 Ptf1a;Nhlh1
237 Creb3l1;Bach1
238 Creb5;Crem
246 Mafa;Maff
250 Nfil3;nfil3-2
251 Cebpb;cebpb
255 Fos;fosaa
11 Snai1;snai1a;Scrt2
34 Sp6;Sp5;Sp2
35 Tbx2;tbx16;Tbx5
50 Dmrt3;Dmrta1;Dmrta2
57 Spic;Elf1;spic
74 Gata3;Gata5;gata1a
90 Pknox1;Tgif1;Meis2
92 Barx1;Msx2;msxc
101 Isl2;Hmx3;hmx4
102 Tlx1;Lhx5;Lhx3
122 Gsx2;Hoxd4;Hoxd3
128 Hoxa10;Hoxc8;Hoxa6
134 Pou3f2;Pou2f1;pou5f3
145 Pax6;Pax2;Pax9
164 Rorc;Nr1d1;nr1d4a
174 Nr2f6;Nr2f1;nr2f5
184 Smad2;Smad4;Smad5
206 Tbx1;Tbx20;Tbx15
226 Npas4;Arnt2;Arntl
236 Fos;Atf3;fosaa
245 Cebpb;Cebpg;cebpb
3 Rfx7;Rfx1;Rfx2;Rfx4
15 Zxdc;Zic1;Glis3;zic6
39 Sp6;Sp5;sp5l;Sp2
83 Nkx2-6;Nkx2-2;Nkx2-4;
Nkx2-5
94 Nkx2-6;Nkx3-1;Nkx2-5;
nkx3.3
135 Pou3f2;Pou2f1;Pou1f1;
pou5f3
143 Prrxl1;Alx3;Dmbx1;Arx
156 Rorc;Rarb;Esrra;esrrd
168 Nr1i2;Rarb;Esrra;
esrrd
169 Esr1;Pparg;Esrra;
esrrd
197 Vwa2;Sox3;Sox8;Sox10
203 Tbx1;Tbx20;Eomes;
tbx21
211 ahr1b;Arnt2;Arntl;Ahr
213 rx1;Alx3;Uncx;
Vsx1
214 Msc;Neurog1;Tcf15;
Neurod6
239 Atf6;xbp1;Creb3l1;
Creb3l3
243 Hlf;Creb5;Nfil3;
nfil3-2
244 Mafa;Nfe2l2;Maff;
Bach1
87 Hoxa10;Pknox1;Hoxc8;
Meis2;Hoxa6
96 Hoxa10;Hoxc8;Hoxa11;
Hoxa6;Hoxd12
103 Hox1b;Otp;Prrxl1;
Otx2;otx1a
113 Hox1b;Prrxl1;rx1;
Otx2;otx1a
131 Prrxl1;rx1;Prrx1;
Alx3;Arx
136 Pou4f1;Pou3f2;Pou2f1;
Pou1f1;pou5f3
165 Rorc;Rarb;Nr2f6;
Nr2f1;nr2f5
219 Sim2;Npas1;Arnt2;
Usf1;Arntl
223 Heyl;hes2.1;her1;
Hes2;Hes1
93 Hoxa10;Hoxb13;Hoxc8;
Hoxa11;Hoxa6;Hoxd12
98 six9;Six3;Six2;six5;
six4b;six7
110 Nkx2-6;Nkx3-1;Nkx2-2;
Nkx2-4;Nkx2-5;nkx3.3
166 Pparg;Hnf4g;Nr2f6;
Rxrb;Nr2f1;nr2f5
192 Vwa2;Sox7;Sox3;Sox21;
Sox8;Sox10
196 Sox3;Sox5;Sox21;Sox8;
Sox10;Sox13
240 Nfe2l2;Jund;Maff;Fos;
Atf3;fosaa
37 Klf4;Klf5;Klf12;Klf6;
klf17;klf3;klf1
104 Cdx1;Hoxa10;Hoxb13;
Hoxc8;Hoxa11;Hoxa6;
Hoxd12
127 Hoxa5;Hoxd4;Hoxc5;
Hoxd3;Hoxc8;Hoxa6;
Hoxa7
157 Rorc;Nr2c1;Rarb;
Nr2f6;Rxrb;Nr2f1;
nr2f5
191 Vwa2;Sox7;Sox3;Sox21;
Sox11;Sox8;Sox10
195 Vwa2;Sox3;Sox5;Sox21;
Sox11;Sox8;Sox10
222 ahr1b;Arnt2;hes2.1;
Hes2;Hes1;Arntl;Ahr
241 Nfe2l2;Jund;Fos;Atf3;
Bach1;Batf3;fosaa
63 Fli1;Gabpa;Elk3;Elk4;
Etv1;Fev;Elk1;etv2
73 Foxa1;Foxl2;Foxc1;
Foxe3;Foxd1;Foxf2;
Foxf1;Foxe1
126 Pdx1;Hoxa5;Hoxd4;
Hoxc5;Hoxd3;Hoxc8;
Hoxa6;Hoxa7
204 Tbx1;Tbx20;Tbx2;
Tbx15;tbx16;Tbx5;
Eomes;tbx21
242 Creb5;Crem;Jund;xbp1;
Atf3;Creb3l1;Creb3l3;
Batf3
95 Pdx1;Hoxa5;Hoxb2;
Hoxd4;Hoxc5;Lhx8;
Hoxd3;Hoxa6;Hoxa7
115 Otp;Prrxl1;rx1;Prrx1;
Alx3;Dmbx1;Uncx;Vsx1;
Arx
117 Pdx1;Hoxa5;Hoxb2;
Hoxd4;Hoxc5;Hoxd3;
Hoxc8;Hoxa6;Hoxa7
60 Fli1;Gabpa;Elk3;Elk4;
Etv1;Erf;Fev;Elk1;etv2;
si:ch211-265g22.4
221 Mnt;Npas2;Bhlhe41;
hes2.1;her1;Hes2;
Hes1;Mlx;Tfe3;Max
106 Hox1b;Gsc;Otp;Prrxl1;
rx1;Alx3;Dmbx1;Pitx2;
Otx2;Arx;otx1a
107 Pdx1;Hoxa5;Hoxb2;
Hoxd4;Hoxc5;Hoxa10;
Hoxd3;Pbx1;Hoxc8;
Hoxa6;Hoxa7
144 Prop1;Otp;Prrxl1;rx1;
Prrx1;Alx3;Shox2;
Dmbx1;Uncx;Vsx1;Arx
14 Sp6;Klf4;Klf5;Sp5;
sp5l;Klf11;Sp2;Klf12;
Klf6;klf17;klf3;klf1
58 Elf5;Fli1;Gabpa;Elk3;
Elk4;Etv1;Elf1;Erf;
Fev;Elk1;
etv2;si:ch211-265g22.4
142 Hox1b;Otp;Prrxl1;rx1;
Prrx1;Alx3;Shox2;Dmbx1;
Otx2;Uncx;Vsx1;Arx
216 Msc;Twist2;Neurog1;
Tcf15;Tcf3;Bhlha15;
Neurod6;Bhlhe23;Olig3;
Atoh7;Tal2;tal2
97 Hox1b;Prop1;Otp;Prrxl1;
rx1;Prrx1;Alx3;Shox2;
Dmbx1;Otx2;Uncx;Vsx1;Arx
59 Elf5;Fli1;Gabpa;Elk3;
Elk4;Etv1;Spic;Elf1;
Erf;Fev;Elk1;etv2;
si:ch211-265g22.4;spic
72 Foxa1;Foxj2;Foxl2;Foxc1;
Foxn3;Foxd1;Foxf2;Foxk1;
Foxf1;Foxi3;Foxg1;Foxj3;
foxg1b;foxl1
27 Sp6;Klf4;Klf5;Sp5;sp5l;
Klf11;Sp2;wt1b;Klf12;
Klf6;Egr1;Wt1;klf17;
klf3;klf1
66 Foxb2;Foxa1;Foxj2;Foxl2;
Foxc1;Foxe3;Foxd1;Foxf2;
Foxk1;Foxf1;Foxi3;Foxg1;
Foxe1;foxq2;Foxj3;
foxg1b;foxl1
67 Foxb2;Foxa1;Foxj2;Foxl2;
Foxc1;Foxn3;Foxe3;Foxd1;
Foxf2;Foxk1;Foxf1;Foxi3;
Foxg1;Foxe1;foxq2;Foxj3;
foxg1b;foxl1
163 Nr5a1;Esr1;Nr1h4;Nr1i2;
Rorc;Pparg;Nr2c1;Rarb;
Nr2e1;Nr2f6;Esrra;esrrd;
Rxrb;Nr4a3;Thrb;Nr2f1;
nr2f5;nr5a5
69 Foxb2;Foxa1;Foxj2;Foxl2;
Foxc1;Foxo1;Foxn3;Foxe3;
Foxd1;Foxf2;Foxk1;Foxm1;
Foxf1;Foxi3;Foxg1;Foxe1;
foxq2;Foxj3;foxg1b;foxl1
68 Foxb2;Foxa1;Foxj2;Foxl2;
Foxc1;Foxo1;Foxn3;Foxe3;
Foxd1;Foxp1;Foxf2;Foxk1;
Foxf1;Foxi3;Foxg1;Foxe1;
foxq2;Foxj3;Foxh1;
foxg1b;foxl1
224 Mnt;Arnt2;Npas2;Heyl;
Bhlhe41;Mlx;Msc;Twist2;
Neurog1;Ptf1a;Myf5;ascl1b;
Usf1;Tcf15;Ascl1;Mycn;
Srebf1;Tcf3;Tfe3;Arntl;
Neurod6;Id1;Nhlh1;Atoh7;
Tal2;Max;tal2
109 Hox1b;Barhl2;Pdx1;Prop1;
Vax2;Hoxa5;Evx2;Dlx3;Otp;
Gsx2;Hoxb2;Nkx2-6;Barx1;
Nkx6-2;Prrxl1;Hoxa1;rx1;
Prrx1;Nkx3-1;Mnx1;Cdx1;
Bsx;Dbx1;Hoxd4;Hlx;Tlx1;
En1;Dlx6;Hoxc5;Msx2;Alx3;
Shox2;Hoxa10;lmx1a;Lmx1a;
Lhx9;Lhx5;Lhx3;Lhx8;Meox2;
Hoxd3;Nkx2-2;Dmbx1;Hoxc8;
Isl2;Nkx2-4;Otx2;Emx2;
Nkx2-5;Uncx;Gbx1;Hoxa6;
Hoxa7;nkx3.3;Vsx1;Vsx2;
Hmx3;Arx;Lbx1;msxc;hmx4;
otx1a;noto;mixl1;Isx;lmx1al

B. lanceolatum modules

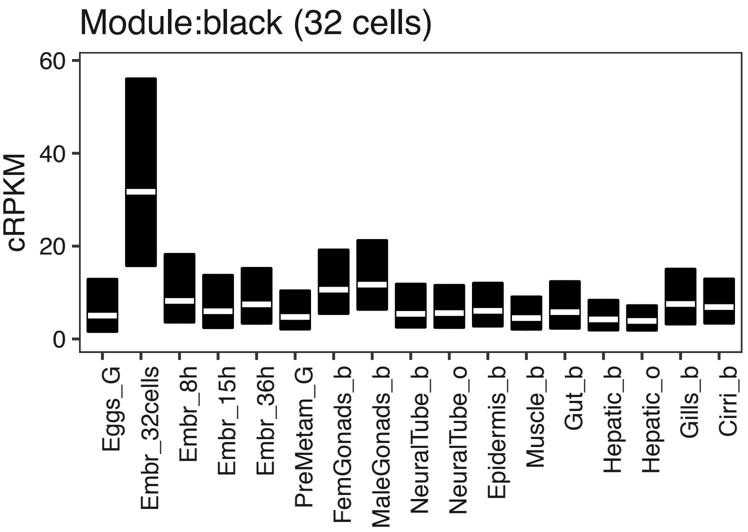

Module:black (32 cells)
cRPKM
0
20
40
60
Eggs_G
Embr_32cells
Embr_8h
Embr_15h
Embr_36h
PreMetam_G
FemGonads_b
MaleGonads_b
NeuralTube_b
NeuralTube_o
Epidermis_b
Muscle_b
Gut_b
Hepatic_b
Hepatic_o
Gills_b
Cirri_b

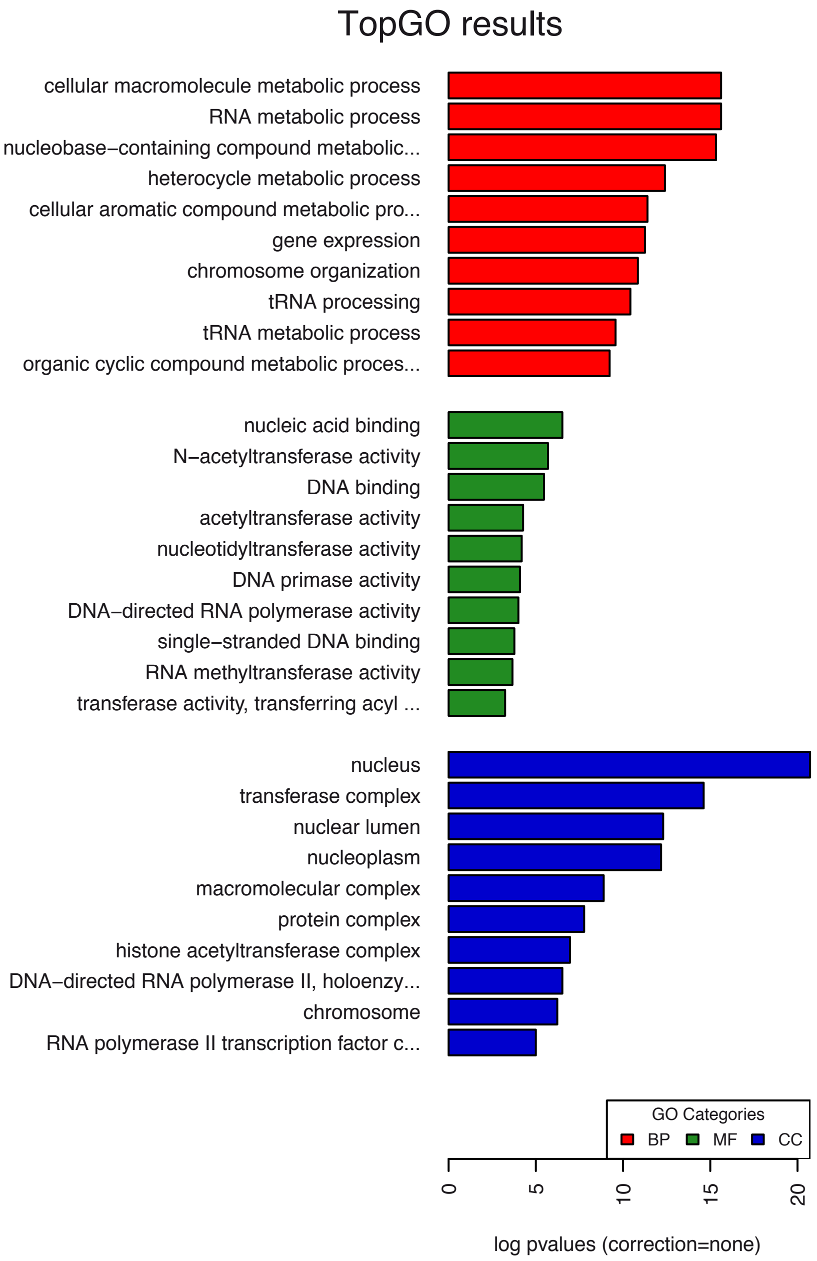

TopGO results
cellular macromolecule metabolic process
RNA metabolic process
nucleobase−containing compound metabolic...
heterocycle metabolic process
cellular aromatic compound metabolic pro...
gene expression
chromosome organization
tRNA processing
tRNA metabolic process
organic cyclic compound metabolic proces...
nucleic acid binding
N−acetyltransferase activity
DNA binding
acetyltransferase activity
nucleotidyltransferase activity
DNA primase activity
DNA−directed RNA polymerase activity
single−stranded DNA binding
RNA methyltransferase activity
transferase activity, transferring acyl ...
nucleus
transferase complex
nuclear lumen
nucleoplasm
macromolecular complex
protein complex
histone acetyltransferase complex
DNA−directed RNA polymerase II, holoenzy...
chromosome
RNA polymerase II transcription factor c...
GO Categories
BP
MF
CC
0
5
10
15
20
log pvalues (correction=none)

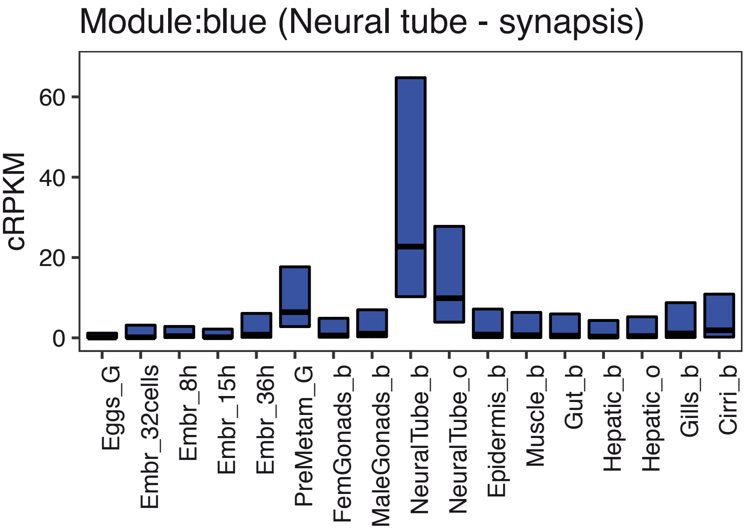

Module:blue (Neural tube - synapsis)
cRPKM
60
40
20
0
Eggs_G
Embr_32cells
Embr_8h
Embr_15h
Embr_36h
PreMetam_G
FemGonads_b
MaleGonads_b
NeuralTube_b
NeuralTube_o
Epidermis_b
Muscle_b
Gut_b
Hepatic_b
Hepatic_o
Gills_b
Cirri_b

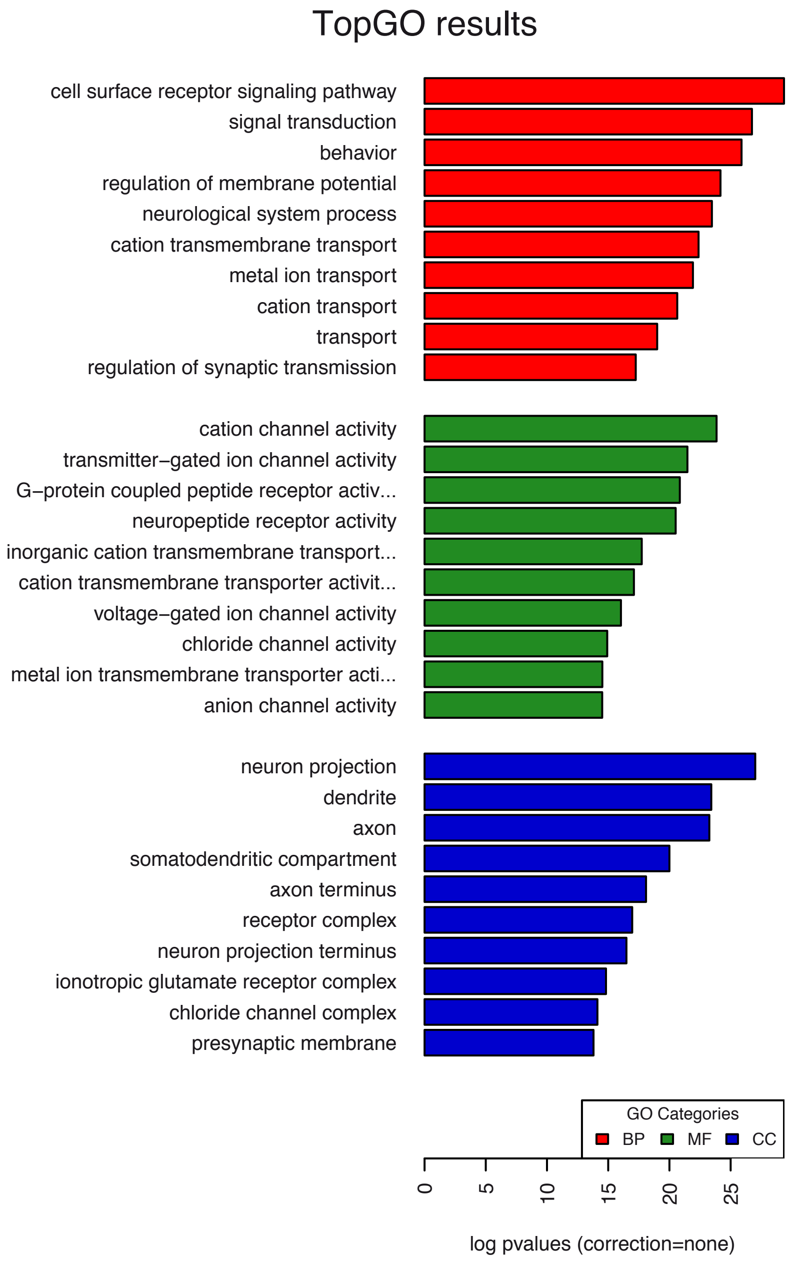

TopGO results
cell surface receptor signaling pathway
signal transduction
behavior
regulation of membrane potential
neurological system process
cation transmembrane transport
metal ion transport
cation transport
transport
regulation of synaptic transmission
cation channel activity
transmitter−gated ion channel activity
G−protein coupled peptide receptor activ...
neuropeptide receptor activity
inorganic cation transmembrane transport...
cation transmembrane transporter activit...
voltage−gated ion channel activity
chloride channel activity
metal ion transmembrane transporter acti...
anion channel activity
neuron projection
dendrite
axon
somatodendritic compartment
axon terminus
receptor complex
neuron projection terminus
ionotropic glutamate receptor complex
chloride channel complex
presynaptic membrane
GO Categories
BP
MF
CC
0
5
10
15
20
25
log pvalues (correction=none)

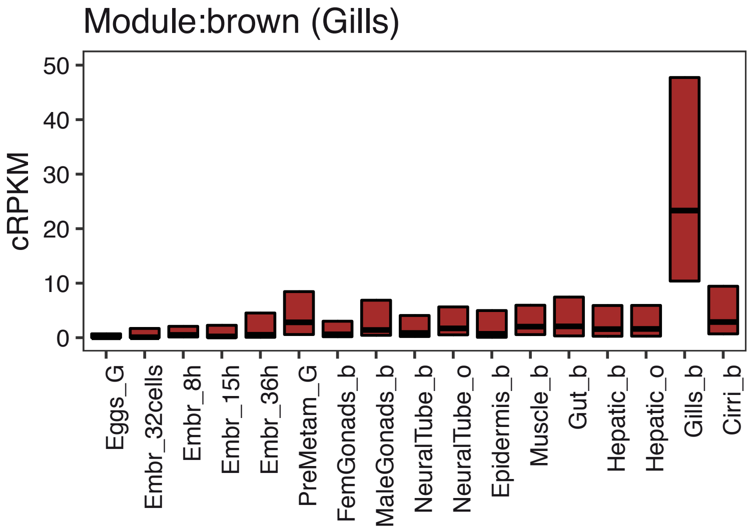
Module:brown (Gills)
cRPKM
0
10
20
30
40
50
Eggs_G
Embr_32cells
Embr_8h
Embr_15h
Embr_36h
PreMetam_G
FemGonads_b
MaleGonads_b
NeuralTube_b
NeuralTube_o
Epidermis_b
Muscle_b
Gut_b
Hepatic_b
Hepatic_o
Gills_b
Cirri_b

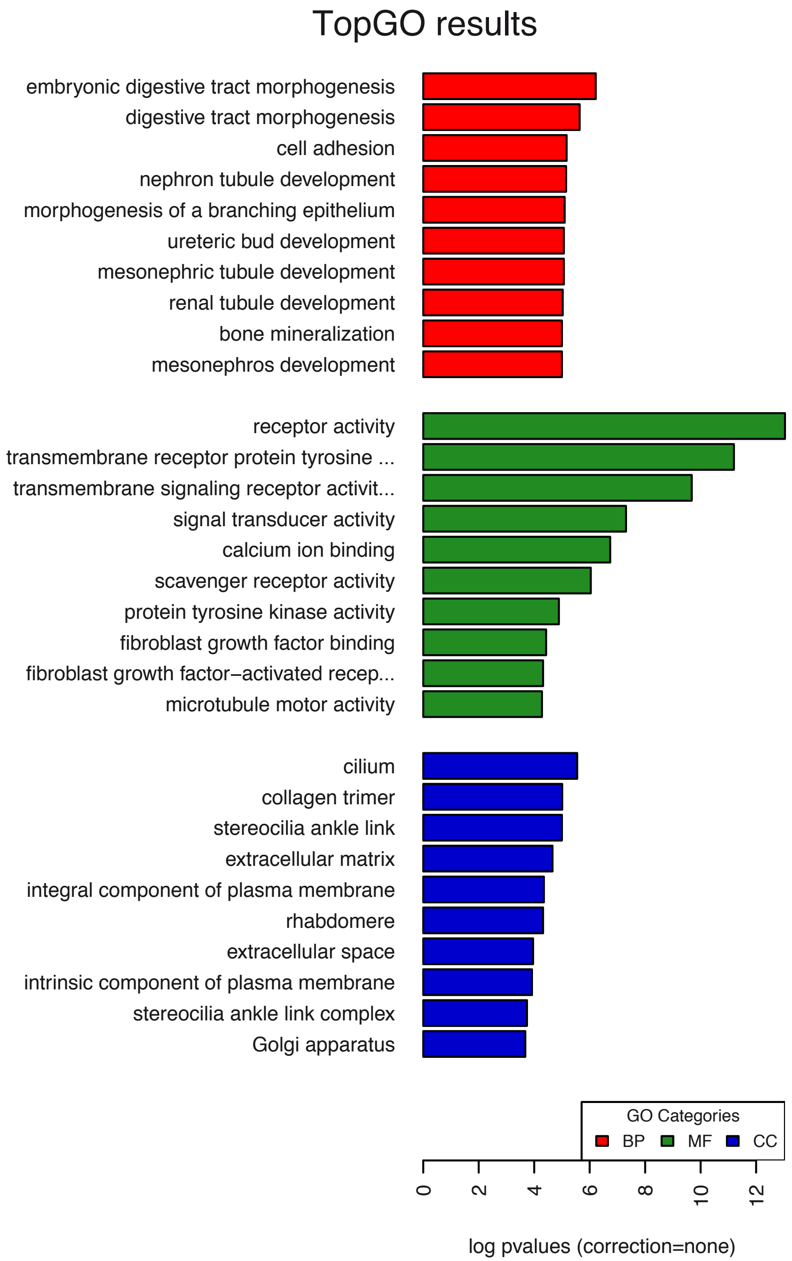
TopGO results
embryonic digestive tract morphogenesis
digestive tract morphogenesis
cell adhesion
nephron tubule development
morphogenesis of a branching epithelium
ureteric bud development
mesonephric tubule development
renal tubule development
bone mineralization
mesonephros development
receptor activity
transmembrane receptor protein tyrosine ...
transmembrane signaling receptor activit...
signal transducer activity
calcium ion binding
scavenger receptor activity
protein tyrosine kinase activity
fibroblast growth factor binding
fibroblast growth factor−activated recep...
microtubule motor activity
cilium
collagen trimer
stereocilia ankle link
extracellular matrix
integral component of plasma membrane
rhabdomere
extracellular space
intrinsic component of plasma membrane
stereocilia ankle link complex
Golgi apparatus
GO Categories
BP
MF
CC
0
2
4
6
8
10
12
log pvalues (correction=none)

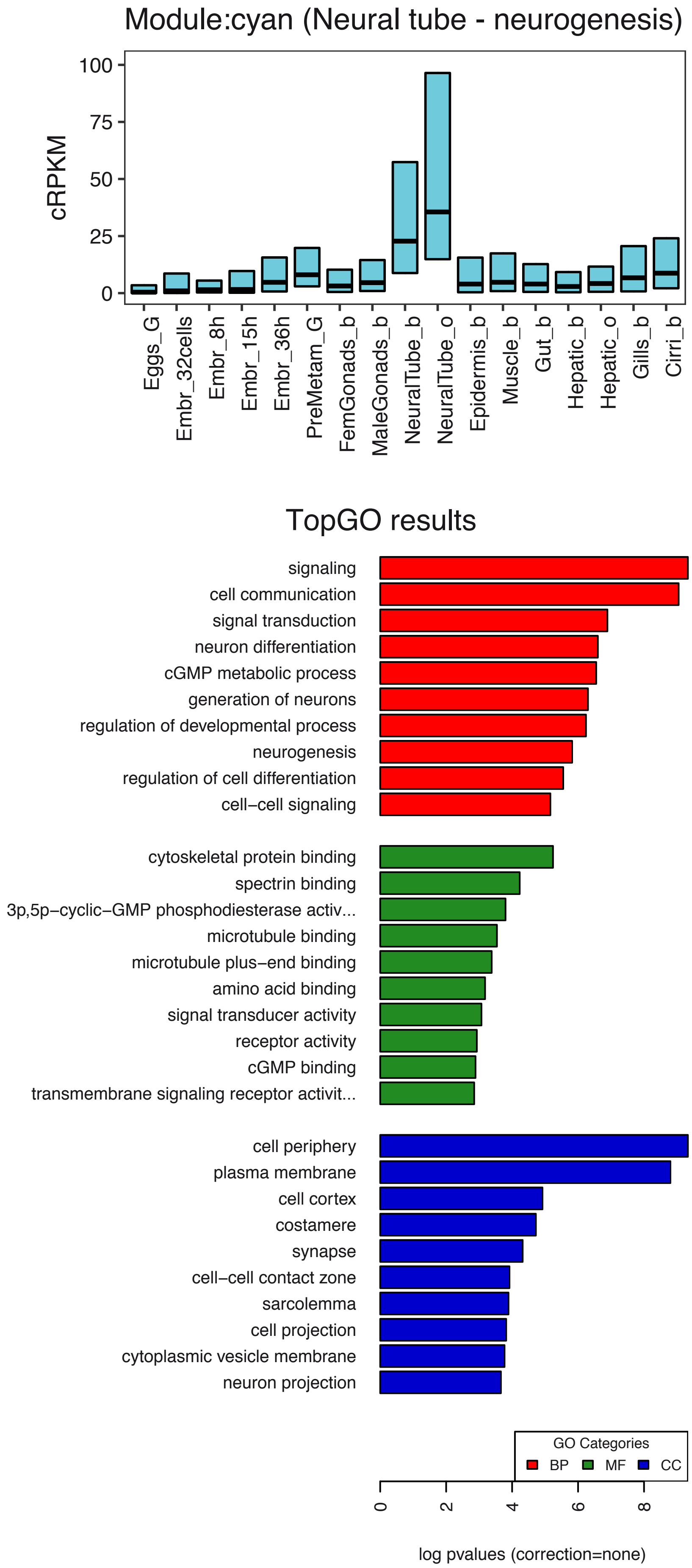

Module:cyan (Neural tube - neurogenesis)
cRPKM
100
75
50
25
0
Eggs_G
Embr_32cells
Embr_8h
Embr_15h
Embr_36h
PreMetam_G
FemGonads_b
MaleGonads_b
NeuralTube_b
NeuralTube_o
Epidermis_b
Muscle_b
Gut_b
Hepatic_b
Hepatic_o
Gills_b
Cirri_b
TopGO results
signaling
cell communication
signal transduction
neuron differentiation
cGMP metabolic process
generation of neurons
regulation of developmental process
neurogenesis
regulation of cell differentiation
cell−cell signaling
cytoskeletal protein binding
spectrin binding
3p,5p−cyclic−GMP phosphodiesterase activ...
microtubule binding
microtubule plus−end binding
amino acid binding
signal transducer activity
receptor activity
cGMP binding
transmembrane signaling receptor activit...
cell periphery
plasma membrane
cell cortex
costamere
synapse
cell−cell contact zone
sarcolemma
cell projection
cytoplasmic vesicle membrane
neuron projection
GO Categories
BP
MF
CC
0
2
4
6
8
log pvalues (correction=none)

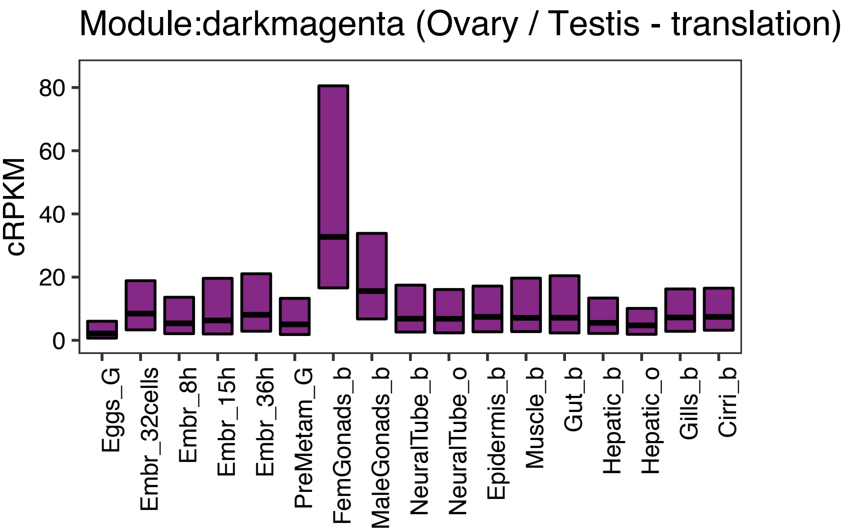
Module:darkmagenta (Ovary / Testis - translation)
cRPKM
0
20
40
60
80
Eggs_G
Embr_32cells
Embr_8h
Embr_15h
Embr_36h
PreMetam_G
FemGonads_b
MaleGonads_b
NeuralTube_b
NeuralTube_o
Epidermis_b
Muscle_b
Gut_b
Hepatic_b
Hepatic_o
Gills_b
Cirri_b

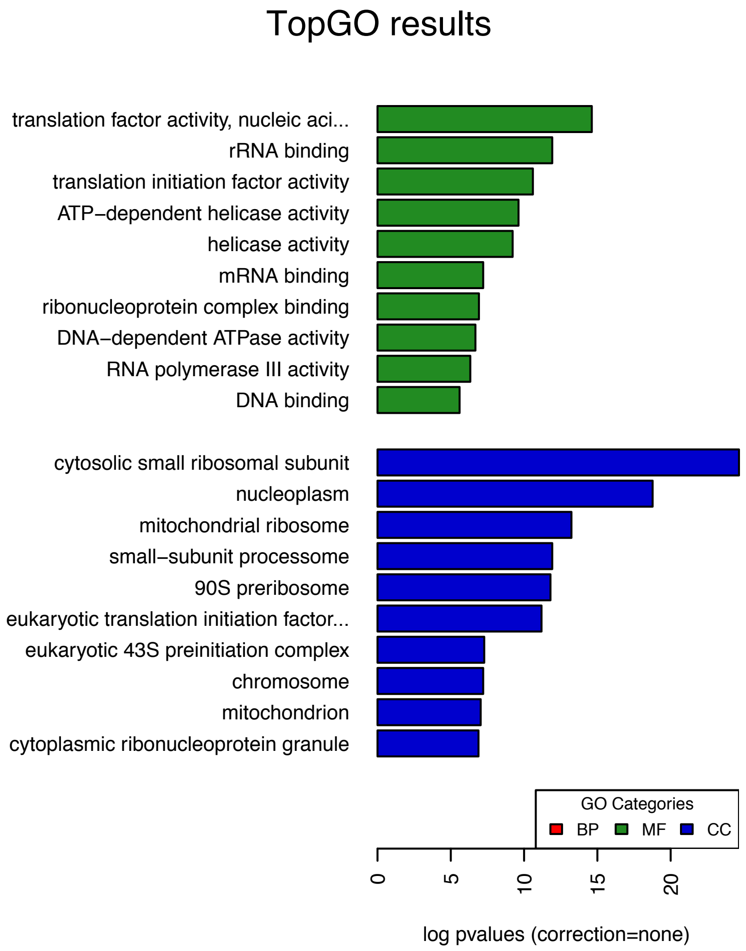
TopGO results
translation factor activity, nucleic aci...
rRNA binding
translation initiation factor activity
ATP−dependent helicase activity
helicase activity
mRNA binding
ribonucleoprotein complex binding
DNA−dependent ATPase activity
RNA polymerase III activity
DNA binding
cytosolic small ribosomal subunit
nucleoplasm
mitochondrial ribosome
small−subunit processome
90S preribosome
eukaryotic translation initiation factor...
eukaryotic 43S preinitiation complex
chromosome
mitochondrion
cytoplasmic ribonucleoprotein granule
GO Categories
BP
MF
CC
0
5
10
15
20
log pvalues (correction=none)

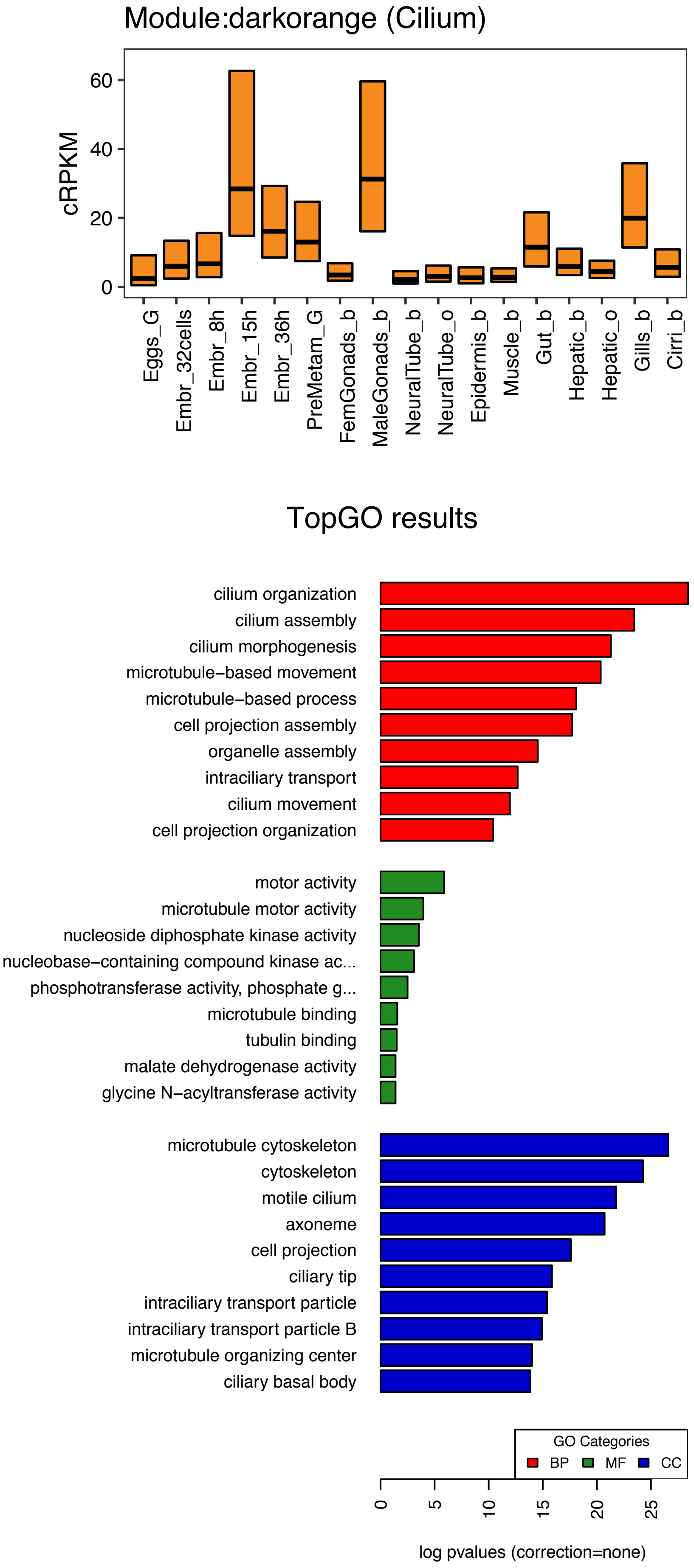

Module:darkorange (Cilium)
cRPKM
0
20
40
60
Eggs_G
Embr_32cells
Embr_8h
Embr_15h
Embr_36h
PreMetam_G
FemGonads_b
MaleGonads_b
NeuralTube_b
NeuralTube_o
Epidermis_b
Muscle_b
Gut_b
Hepatic_b
Hepatic_o
Gills_b
Cirri_b
TopGO results
cilium organization
cilium assembly
cilium morphogenesis
microtubule−based movement
microtubule−based process
cell projection assembly
organelle assembly
intraciliary transport
cilium movement
cell projection organization
motor activity
microtubule motor activity
nucleoside diphosphate kinase activity
nucleobase−containing compound kinase ac...
phosphotransferase activity, phosphate g...
microtubule binding
tubulin binding
malate dehydrogenase activity
glycine N−acyltransferase activity
microtubule cytoskeleton
cytoskeleton
motile cilium
axoneme
cell projection
ciliary tip
intraciliary transport particle
intraciliary transport particle B
microtubule organizing center
ciliary basal body
GO Categories
BP
MF
CC
0
5
10
15
20
25
log pvalues (correction=none)

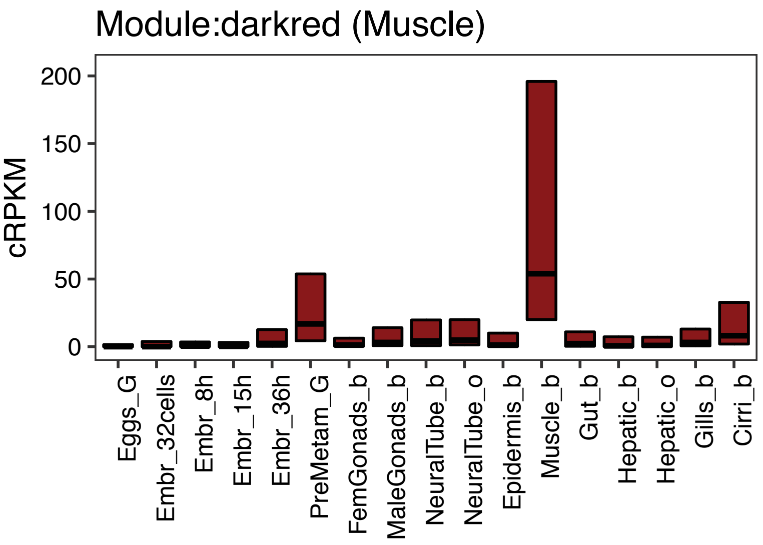

Module:darkred (Muscle)
cRPKM
0
50
100
150
200
Eggs_G
Embr_32cells
Embr_8h
Embr_15h
Embr_36h
PreMetam_G
FemGonads_b
MaleGonads_b
NeuralTube_b
NeuralTube_o
Epidermis_b
Muscle_b
Gut_b
Hepatic_b
Hepatic_o
Gills_b
Cirri_b

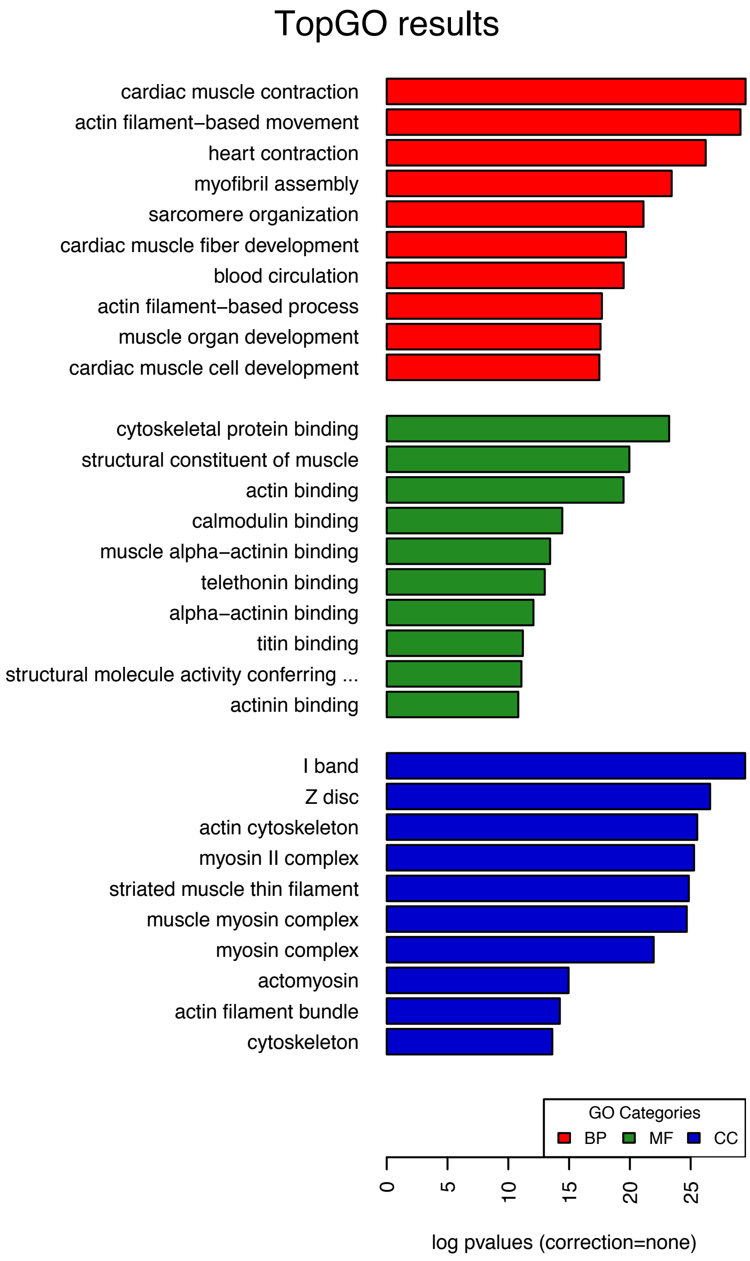

TopGO results
cardiac muscle contraction
actin filament–based movement
heart contraction
myofibril assembly
sarcomere organization
cardiac muscle fiber development
blood circulation
actin filament–based process
muscle organ development
cardiac muscle cell development
cytoskeletal protein binding
structural constituent of muscle
actin binding
calmodulin binding
muscle alpha–actinin binding
telethonin binding
alpha–actinin binding
titin binding
structural molecule activity conferring ...
actinin binding
I band
Z disc
actin cytoskeleton
myosin II complex
striated muscle thin filament
muscle myosin complex
myosin complex
actomyosin
actin filament bundle
cytoskeleton
GO Categories
BP
MF
CC
0
5
10
15
20
25
log pvalues (correction=none)

Module:darkseagreen4 (Embryo 8-36h/Skin/Cirri - lipid membranes)

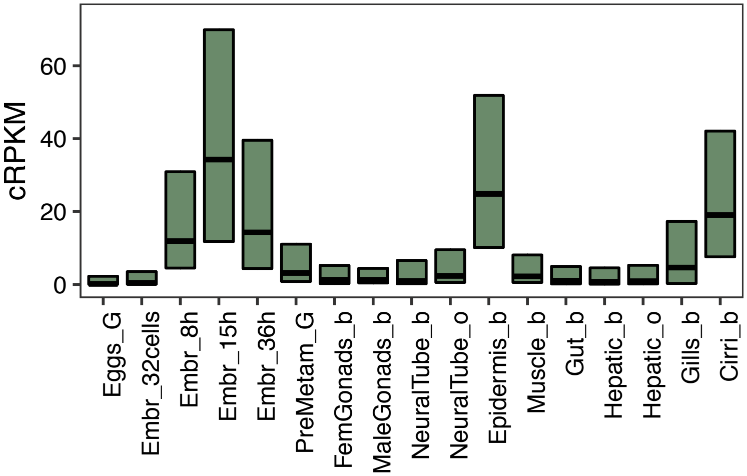

TopGO results

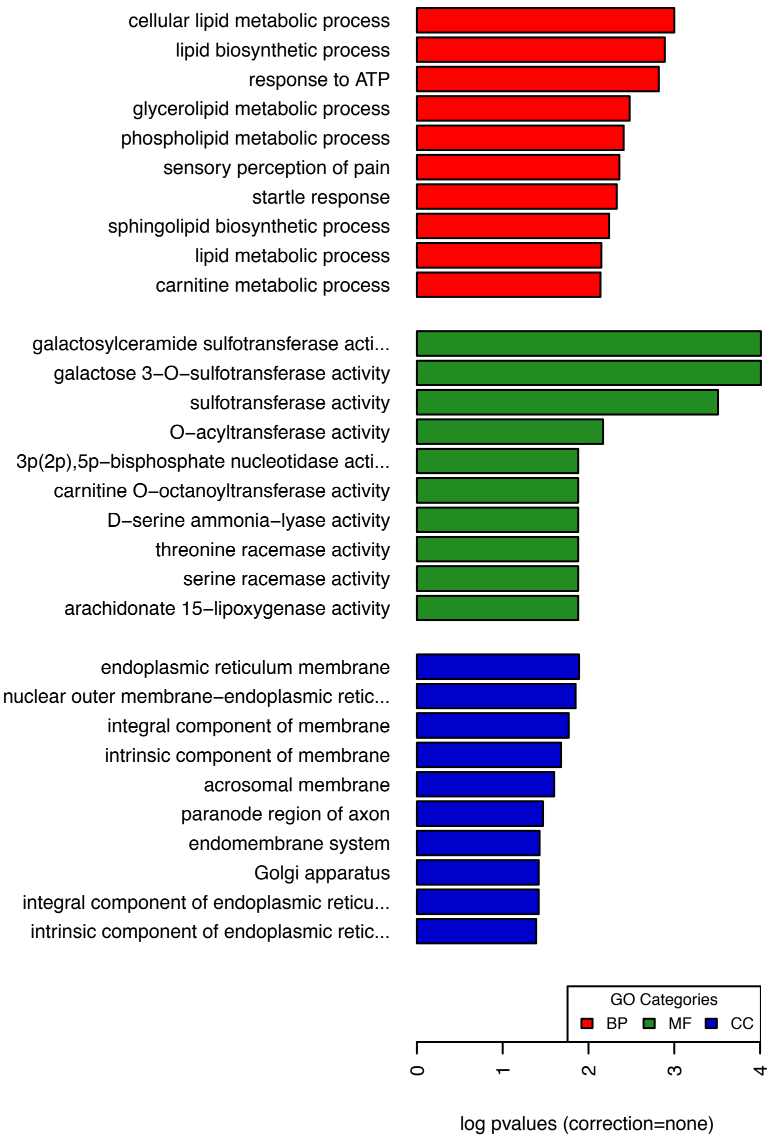

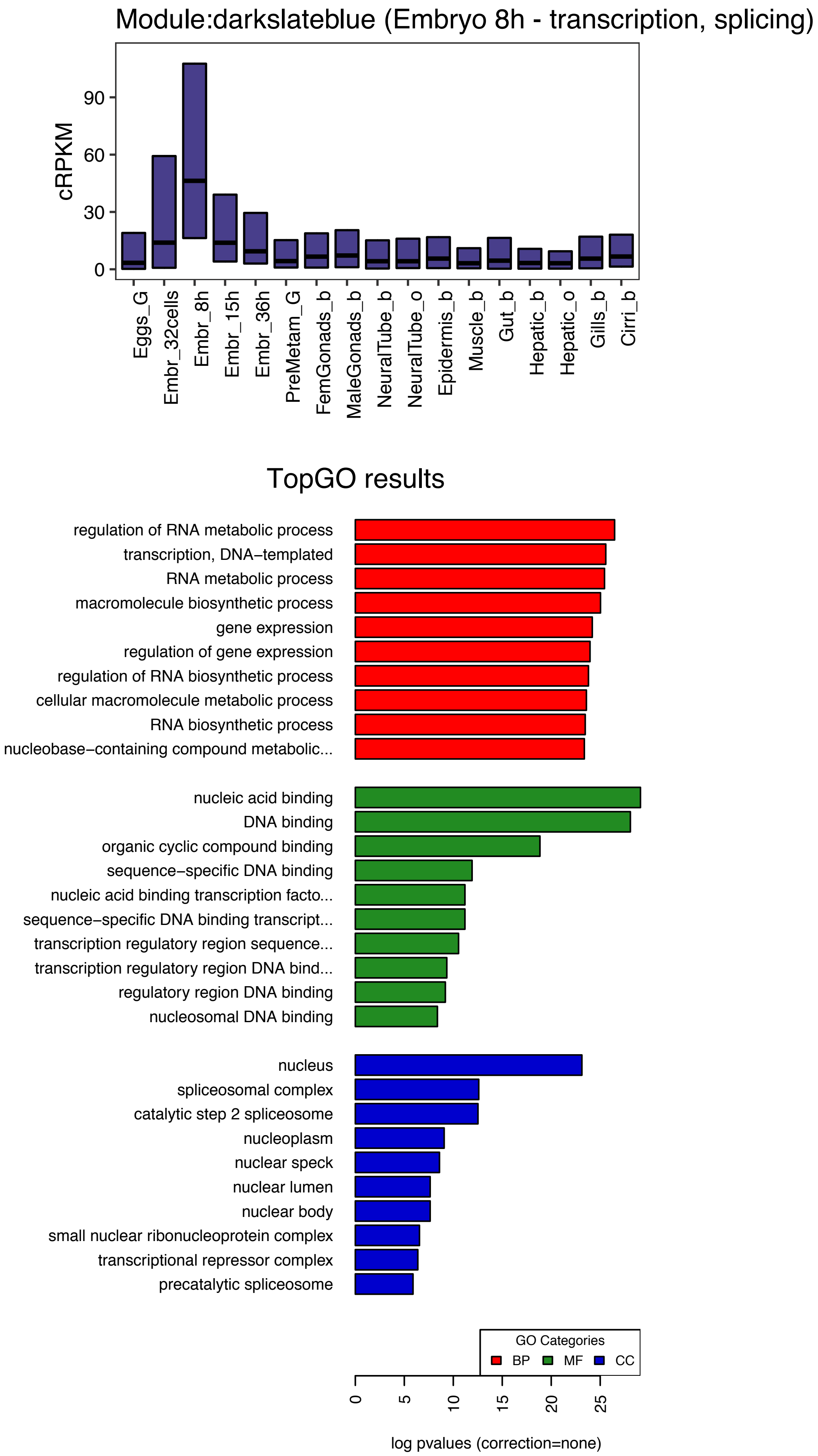

Module:darkslateblue (Embryo 8h - transcription, splicing)
cRPKM
0
30
60
90
Eggs_G
Embr_32cells
Embr_8h
Embr_15h
Embr_36h
PreMetam_G
FemGonads_b
MaleGonads_b
NeuralTube_b
NeuralTube_o
Epidermis_b
Muscle_b
Gut_b
Hepatic_b
Hepatic_o
Gills_b
Cirri_b
TopGO results
regulation of RNA metabolic process
transcription, DNA−templated
RNA metabolic process
macromolecule biosynthetic process
gene expression
regulation of gene expression
regulation of RNA biosynthetic process
cellular macromolecule metabolic process
RNA biosynthetic process
nucleobase−containing compound metabolic...
nucleic acid binding
DNA binding
organic cyclic compound binding
sequence−specific DNA binding
nucleic acid binding transcription facto...
sequence−specific DNA binding transcript...
transcription regulatory region sequence...
transcription regulatory region DNA bind...
regulatory region DNA binding
nucleosomal DNA binding
nucleus
spliceosomal complex
catalytic step 2 spliceosome
nucleoplasm
nuclear speck
nuclear lumen
nuclear body
small nuclear ribonucleoprotein complex
transcriptional repressor complex
precatalytic spliceosome
GO Categories
BP
MF
CC
0
5
10
15
20
25
log pvalues (correction=none)

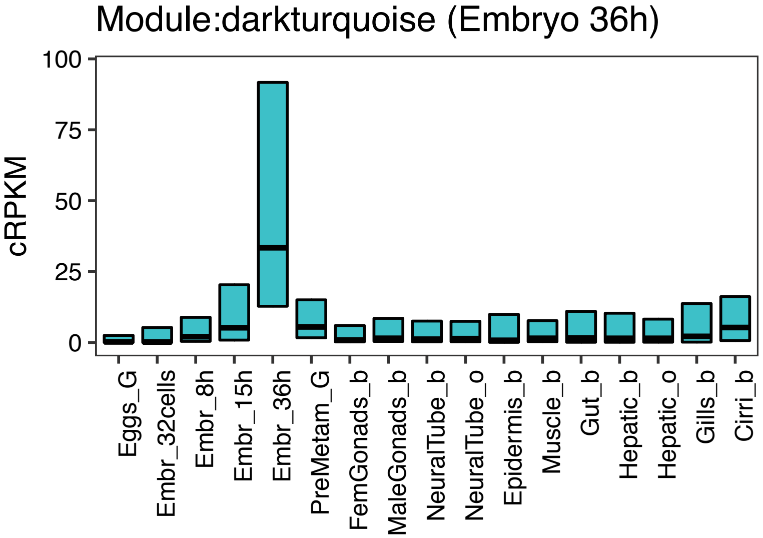
Module:darkturquoise (Embryo 36h)
cRPKM
100
75
50
25
0
Eggs_G
Embr_32cells
Embr_8h
Embr_15h
Embr_36h
PreMetam_G
FemGonads_b
MaleGonads_b
NeuralTube_b
NeuralTube_o
Epidermis_b
Muscle_b
Gut_b
Hepatic_b
Hepatic_o
Gills_b
Cirri_b

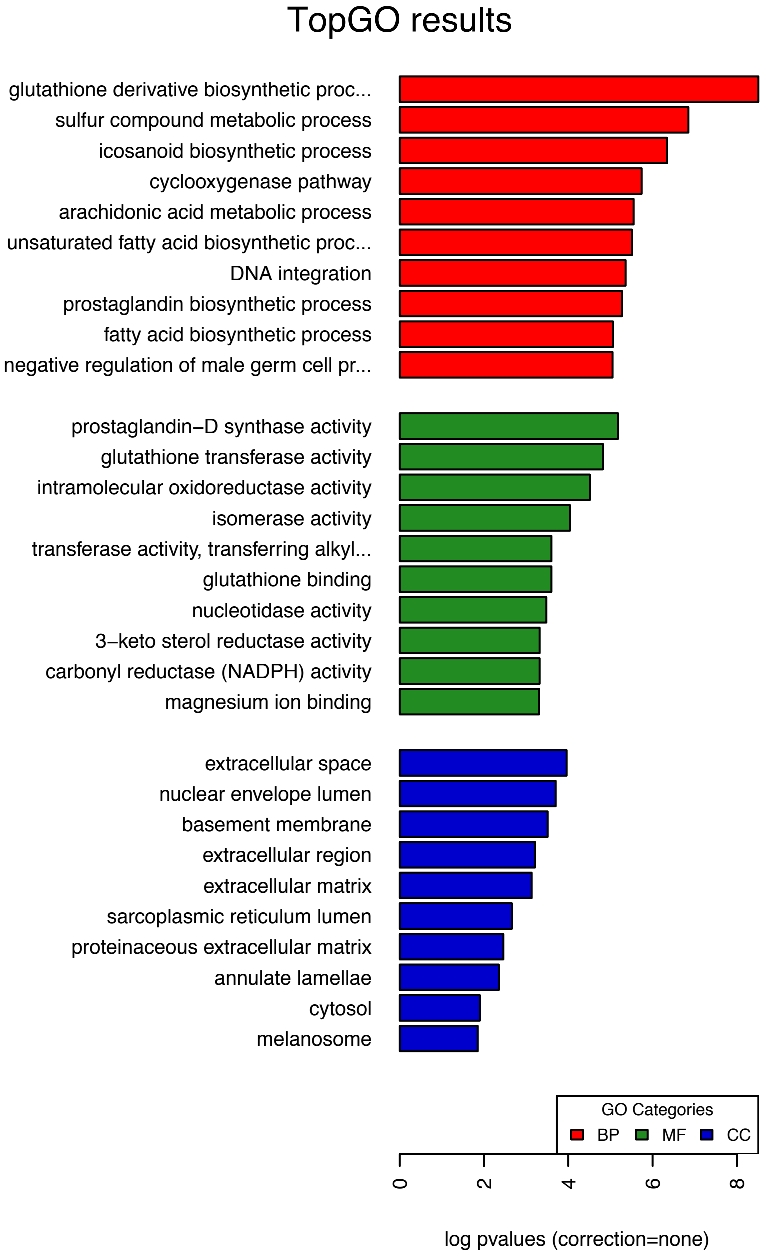
TopGO results
glutathione derivative biosynthetic proc...
sulfur compound metabolic process
icosanoid biosynthetic process
cyclooxygenase pathway
arachidonic acid metabolic process
unsaturated fatty acid biosynthetic proc...
DNA integration
prostaglandin biosynthetic process
fatty acid biosynthetic process
negative regulation of male germ cell pr...
prostaglandin−D synthase activity
glutathione transferase activity
intramolecular oxidoreductase activity
isomerase activity
transferase activity, transferring alkyl...
glutathione binding
nucleotidase activity
3−keto sterol reductase activity
carbonyl reductase (NADPH) activity
magnesium ion binding
extracellular space
nuclear envelope lumen
basement membrane
extracellular region
extracellular matrix
sarcoplasmic reticulum lumen
proteinaceous extracellular matrix
annulate lamellae
cytosol
melanosome
GO Categories
BP
MF
CC
0
2
4
6
8
log pvalues (correction=none)

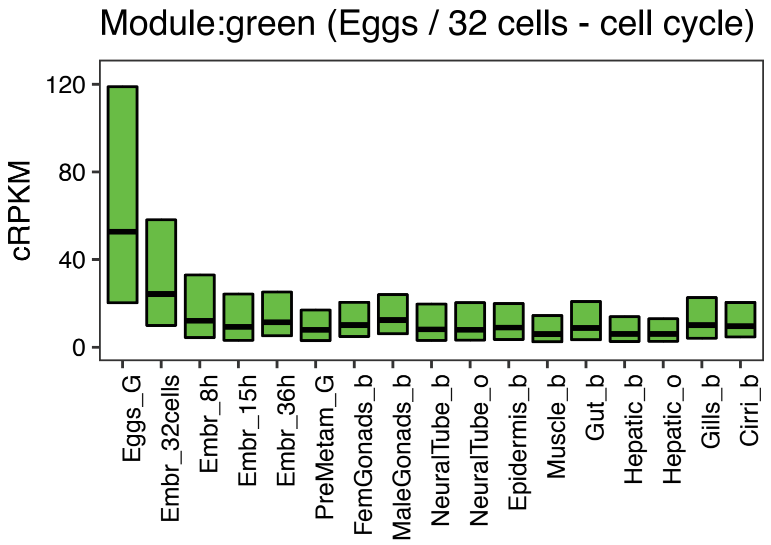
Module:green (Eggs / 32 cells - cell cycle)
cRPKM
120
80
40
0
Eggs_G
Embr_32cells
Embr_8h
Embr_15h
Embr_36h
PreMetam_G
FemGonads_b
MaleGonads_b
NeuralTube_b
NeuralTube_o
Epidermis_b
Muscle_b
Gut_b
Hepatic_b
Hepatic_o
Gills_b
Cirri_b

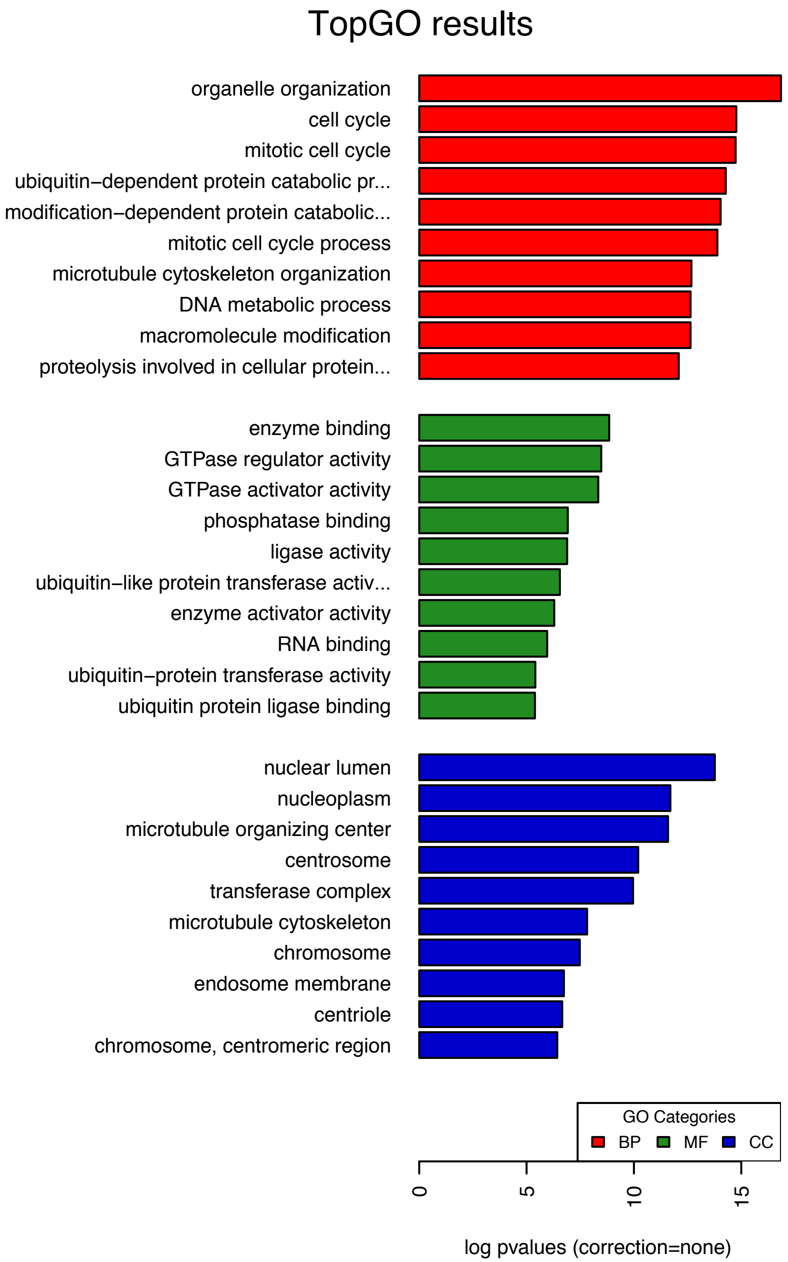
TopGO results
organelle organization
cell cycle
mitotic cell cycle
ubiquitin−dependent protein catabolic pr...
modification−dependent protein catabolic...
mitotic cell cycle process
microtubule cytoskeleton organization
DNA metabolic process
macromolecule modification
proteolysis involved in cellular protein...
enzyme binding
GTPase regulator activity
GTPase activator activity
phosphatase binding
ligase activity
ubiquitin−like protein transferase activ...
enzyme activator activity
RNA binding
ubiquitin−protein transferase activity
ubiquitin protein ligase binding
nuclear lumen
nucleoplasm
microtubule organizing center
centrosome
transferase complex
microtubule cytoskeleton
chromosome
endosome membrane
centriole
chromosome, centromeric region
GO Categories
BP
MF
CC
0
5
10
15
log pvalues (correction=none)

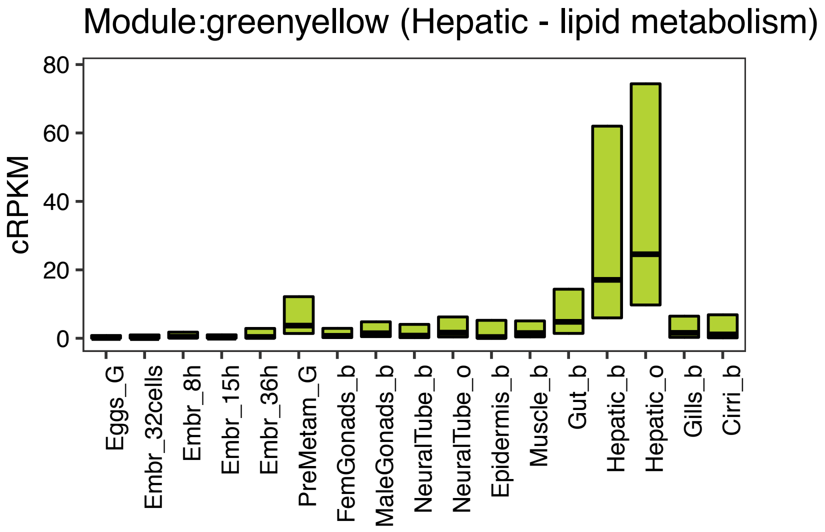
Module:greenyellow (Hepatic - lipid metabolism)
cRPKM
80
60
40
20
0
Eggs_G
Embr_32cells
Embr_8h
Embr_15h
Embr_36h
PreMetam_G
FemGonads_b
MaleGonads_b
NeuralTube_b
NeuralTube_o
Epidermis_b
Muscle_b
Gut_b
Hepatic_b
Hepatic_o
Gills_b
Cirri_b

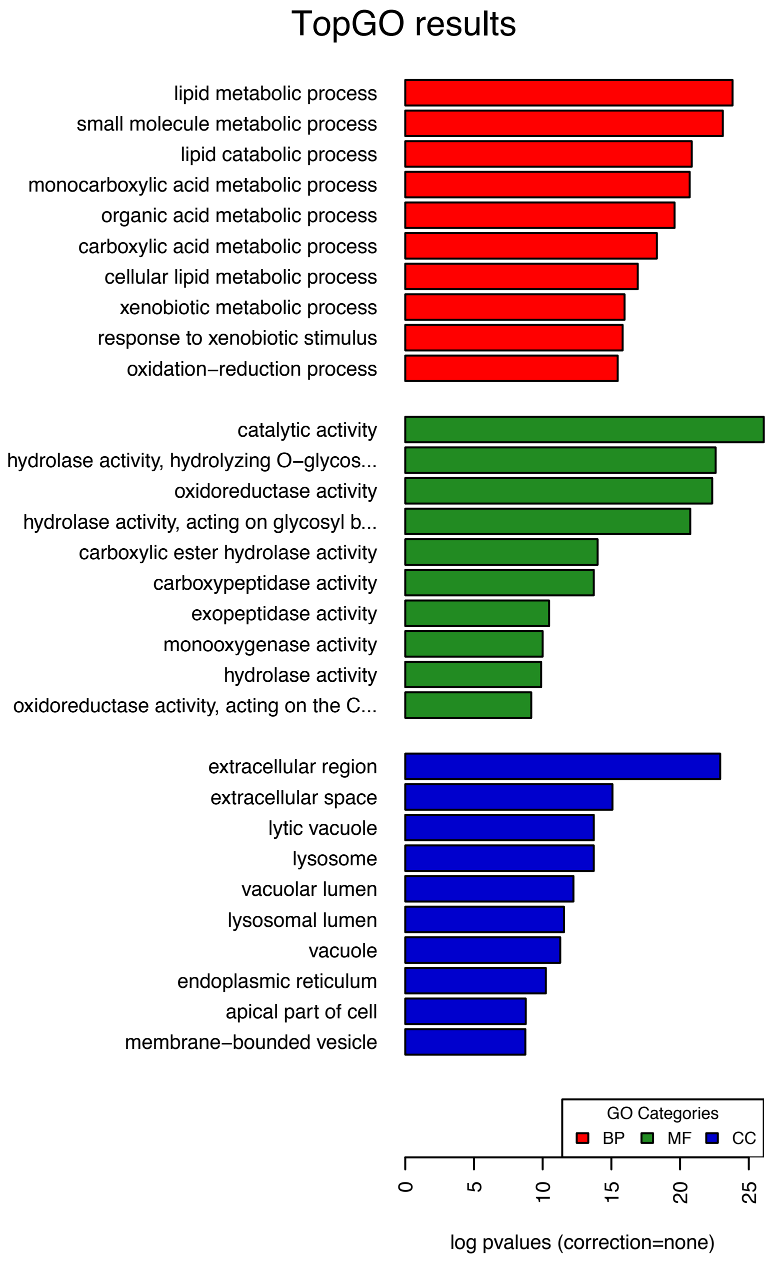
TopGO results
lipid metabolic process
small molecule metabolic process
lipid catabolic process
monocarboxylic acid metabolic process
organic acid metabolic process
carboxylic acid metabolic process
cellular lipid metabolic process
xenobiotic metabolic process
response to xenobiotic stimulus
oxidation−reduction process
catalytic activity
hydrolase activity, hydrolyzing O−glycos...
oxidoreductase activity
hydrolase activity, acting on glycosyl b...
carboxylic ester hydrolase activity
carboxypeptidase activity
exopeptidase activity
monooxygenase activity
hydrolase activity
oxidoreductase activity, acting on the C...
extracellular region
extracellular space
lytic vacuole
lysosome
vacuolar lumen
lysosomal lumen
vacuole
endoplasmic reticulum
apical part of cell
membrane−bounded vesicle
GO Categories
BP
MF
CC
0
5
10
15
20
25
log pvalues (correction=none)

Module:lavenderblush3 (Cirri / PreMet. / Muscle - actomyosin)

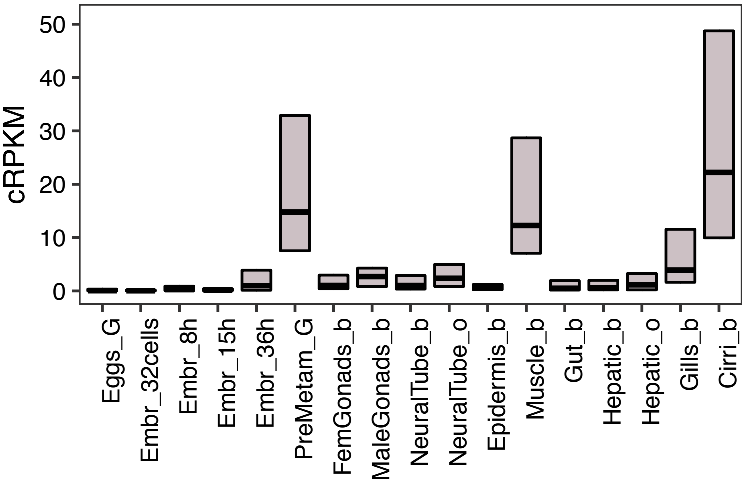

TopGO results

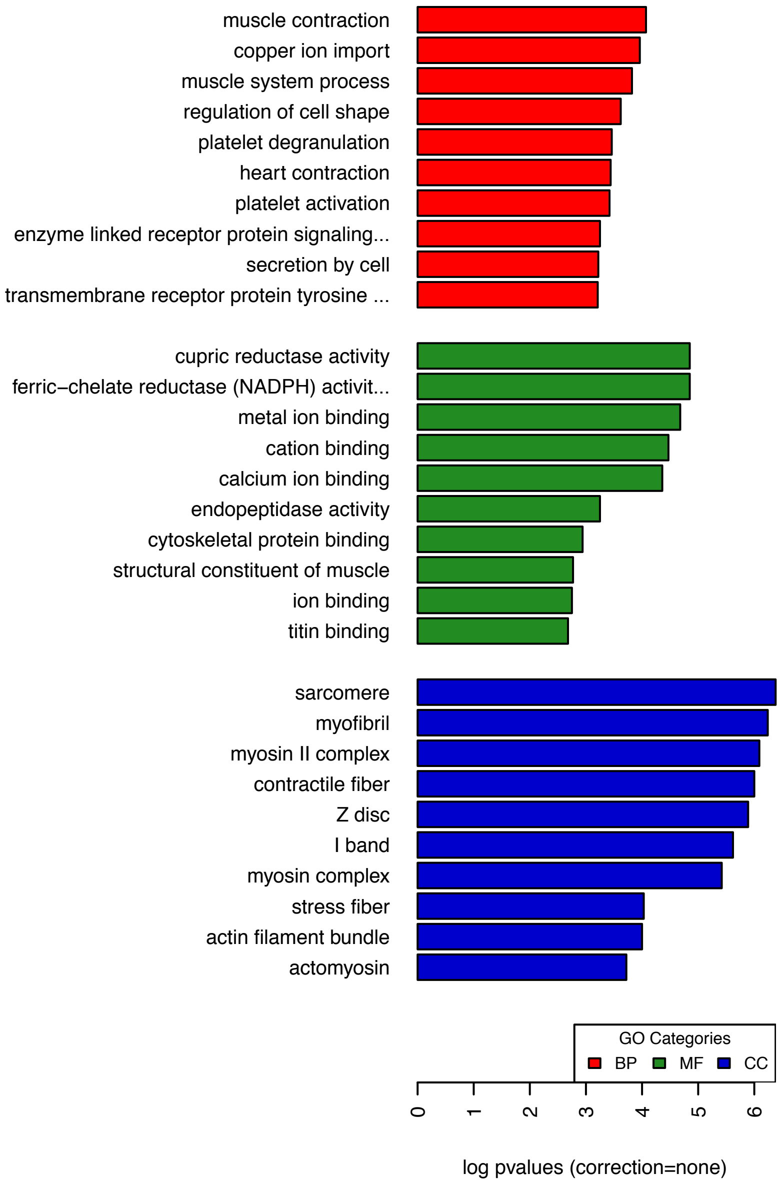

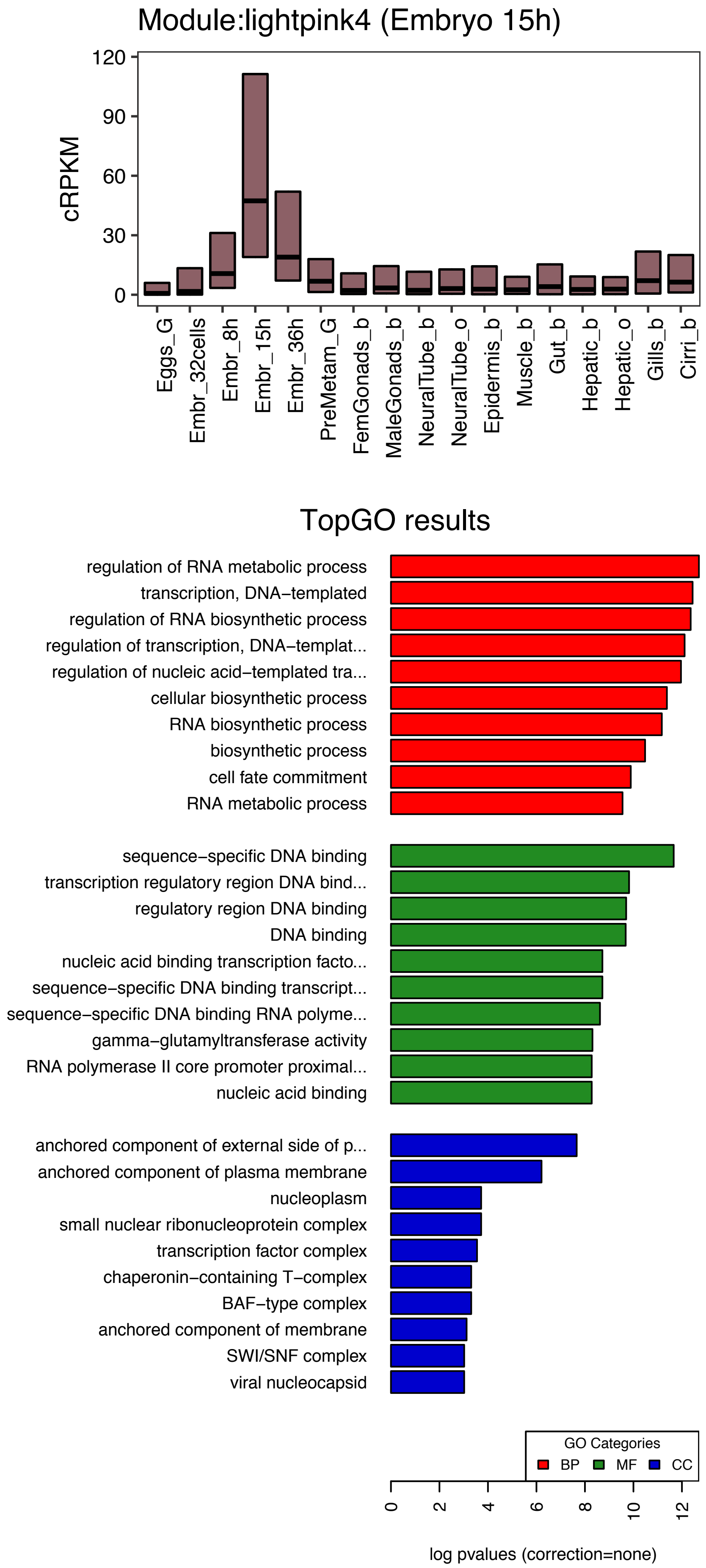

Module:lightpink4 (Embryo 15h)
cRPKM
120
90
60
30
0
Eggs_G
Embr_32cells
Embr_8h
Embr_15h
Embr_36h
PreMetam_G
FemGonads_b
MaleGonads_b
NeuralTube_b
NeuralTube_o
Epidermis_b
Muscle_b
Gut_b
Hepatic_b
Hepatic_o
Gills_b
Cirri_b
TopGO results
regulation of RNA metabolic process
transcription, DNA−templated
regulation of RNA biosynthetic process
regulation of transcription, DNA−templat...
regulation of nucleic acid−templated tra...
cellular biosynthetic process
RNA biosynthetic process
biosynthetic process
cell fate commitment
RNA metabolic process
sequence−specific DNA binding
transcription regulatory region DNA bind...
regulatory region DNA binding
DNA binding
nucleic acid binding transcription facto...
sequence−specific DNA binding transcript...
sequence−specific DNA binding RNA polyme...
gamma−glutamyltransferase activity
RNA polymerase II core promoter proximal...
nucleic acid binding
anchored component of external side of p...
anchored component of plasma membrane
nucleoplasm
small nuclear ribonucleoprotein complex
transcription factor complex
chaperonin−containing T−complex
BAF−type complex
anchored component of membrane
SWI/SNF complex
viral nucleocapsid
GO Categories
BP
MF
CC
0
2
4
6
8
10
12
log pvalues (correction=none)

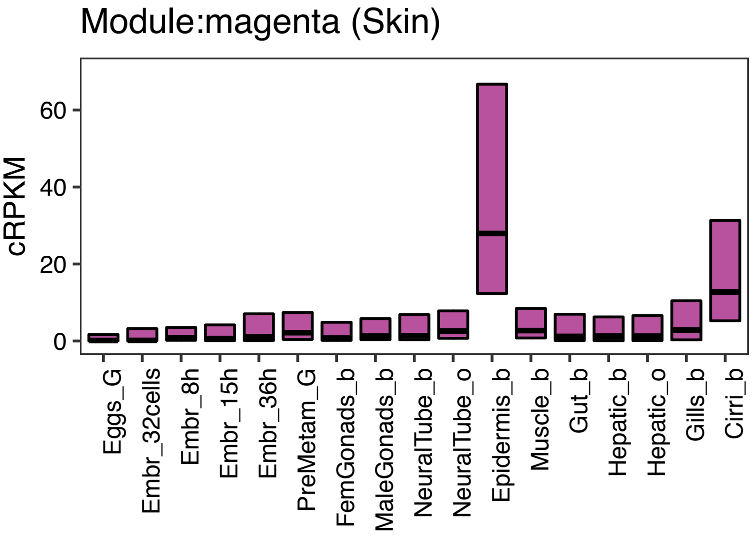

Module:magenta (Skin)
cRPKM
60
40
20
0
Eggs_G
Embr_32cells
Embr_8h
Embr_15h
Embr_36h
PreMetam_G
FemGonads_b
MaleGonads_b
NeuralTube_b
NeuralTube_o
Epidermis_b
Muscle_b
Gut_b
Hepatic_b
Hepatic_o
Gills_b
Cirri_b

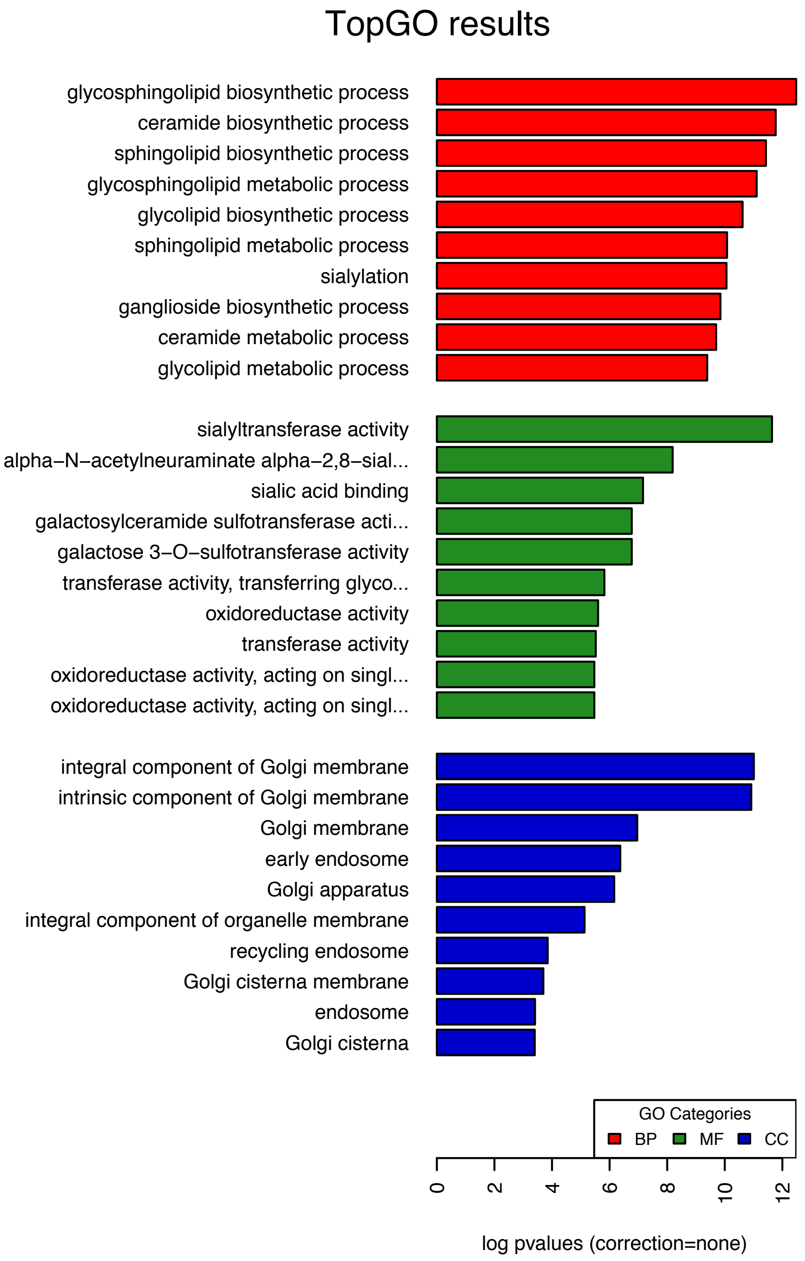

TopGO results
glycosphingolipid biosynthetic process
ceramide biosynthetic process
sphingolipid biosynthetic process
glycosphingolipid metabolic process
glycolipid biosynthetic process
sphingolipid metabolic process
sialylation
ganglioside biosynthetic process
ceramide metabolic process
glycolipid metabolic process
sialyltransferase activity
alpha−N−acetylneuraminate alpha−2,8−sial...
sialic acid binding
galactosylceramide sulfotransferase acti...
galactose 3−O−sulfotransferase activity
transferase activity, transferring glyco...
oxidoreductase activity
transferase activity
oxidoreductase activity, acting on singl...
oxidoreductase activity, acting on singl...
integral component of Golgi membrane
intrinsic component of Golgi membrane
Golgi membrane
early endosome
Golgi apparatus
integral component of organelle membrane
recycling endosome
Golgi cisterna membrane
endosome
Golgi cisterna
GO Categories
BP
MF
CC
0
2
4
6
8
10
12
log pvalues (correction=none)

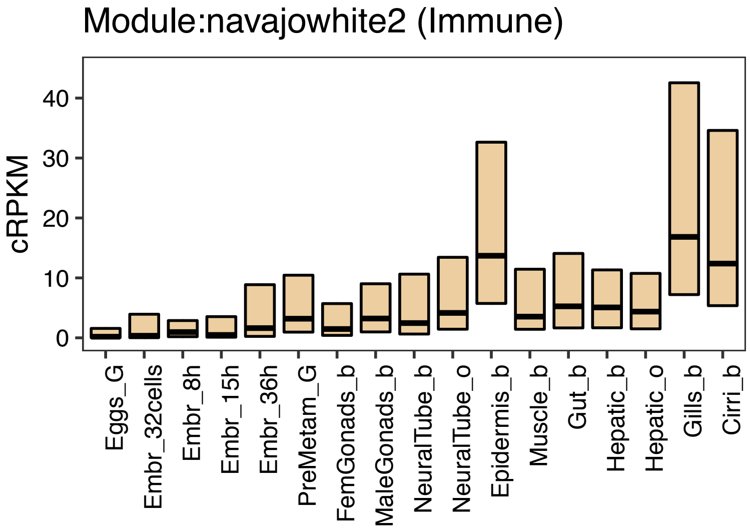
Module:navajowhite2 (Immune)
cRPKM
0
10
20
30
40
Eggs_G
Embr_32cells
Embr_8h
Embr_15h
Embr_36h
PreMetam_G
FemGonads_b
MaleGonads_b
NeuralTube_b
NeuralTube_o
Epidermis_b
Muscle_b
Gut_b
Hepatic_b
Hepatic_o
Gills_b
Cirri_b

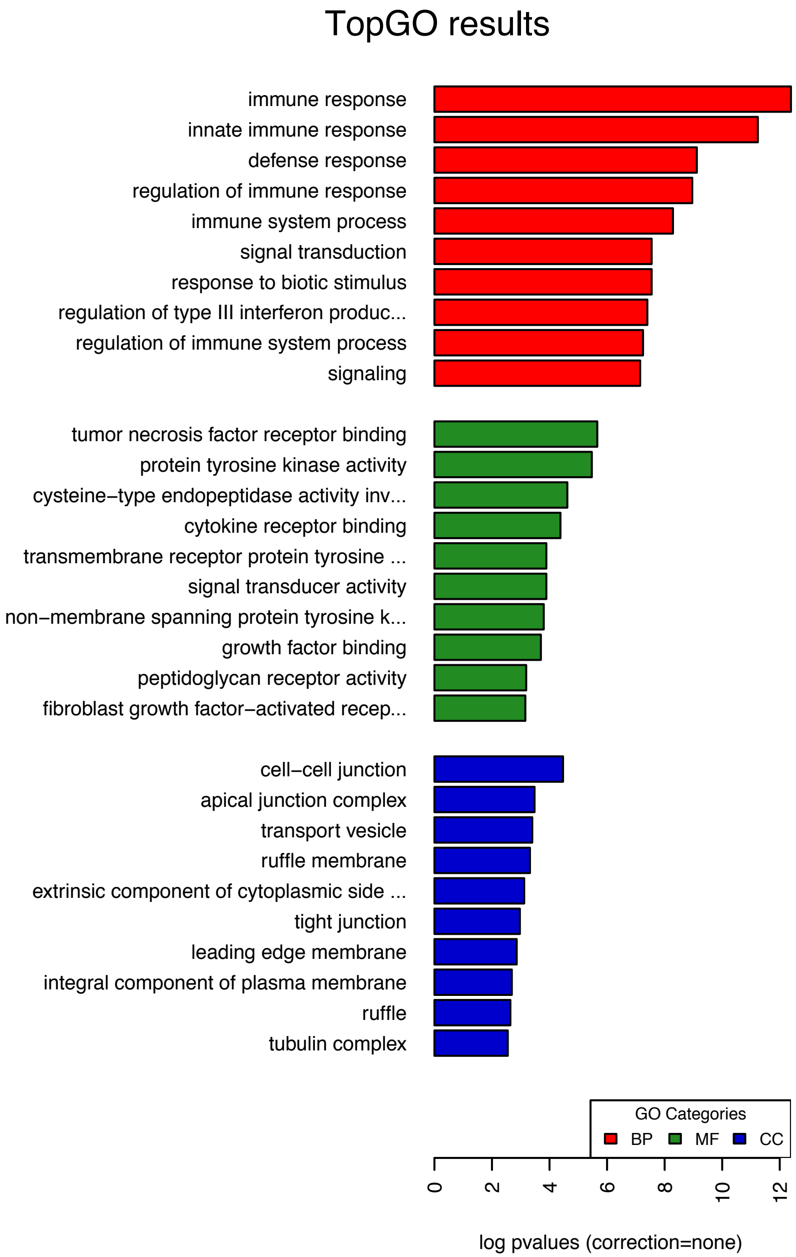
TopGO results
immune response
innate immune response
defense response
regulation of immune response
immune system process
signal transduction
response to biotic stimulus
regulation of type III interferon produc...
regulation of immune system process
signaling
tumor necrosis factor receptor binding
protein tyrosine kinase activity
cysteine−type endopeptidase activity inv...
cytokine receptor binding
transmembrane receptor protein tyrosine ...
signal transducer activity
non−membrane spanning protein tyrosine k...
growth factor binding
peptidoglycan receptor activity
fibroblast growth factor−activated recep...
cell−cell junction
apical junction complex
transport vesicle
ruffle membrane
extrinsic component of cytoplasmic side ...
tight junction
leading edge membrane
integral component of plasma membrane
ruffle
tubulin complex
GO Categories
BP
MF
CC
0
2
4
6
8
10
12
log pvalues (correction=none)

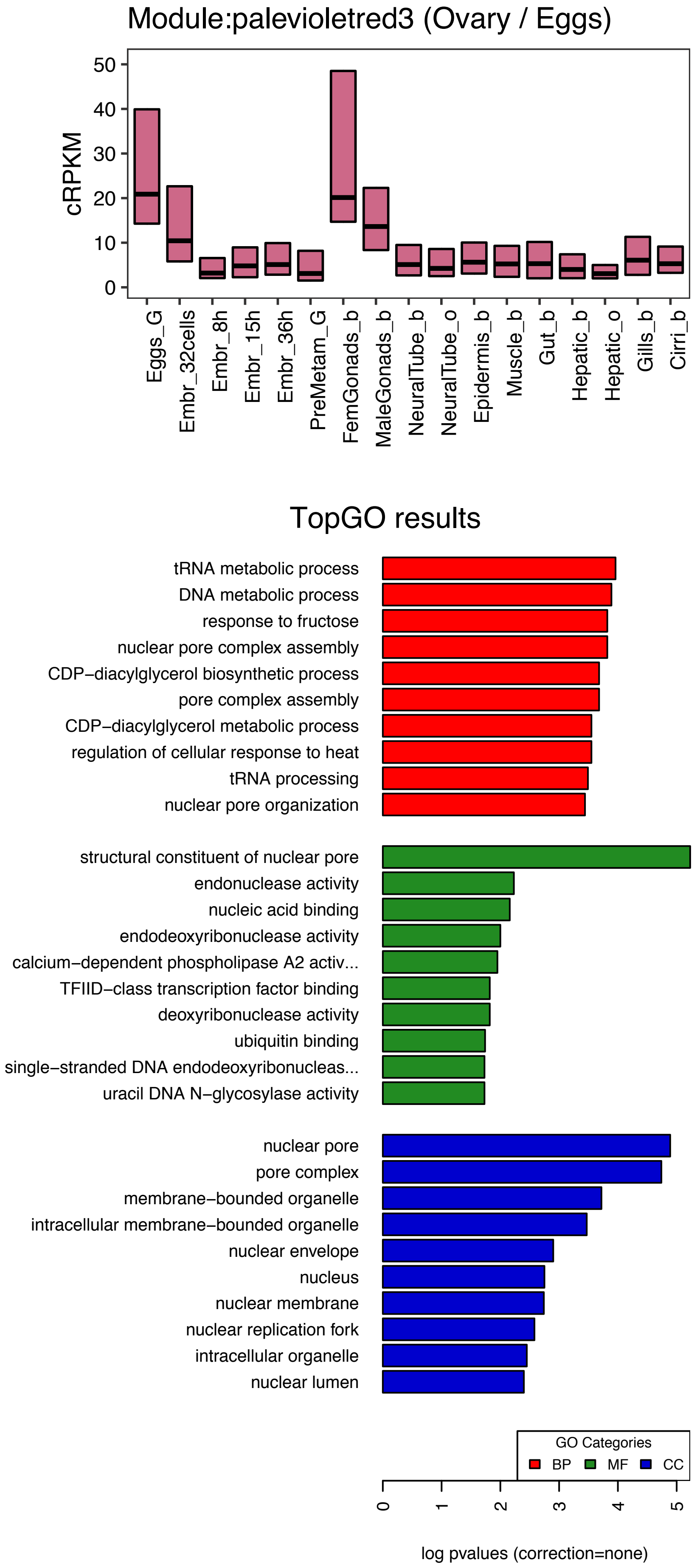

Module:palevioletred3 (Ovary / Eggs)
cRPKM
0
10
20
30
40
50
Eggs_G
Embr_32cells
Embr_8h
Embr_15h
Embr_36h
PreMetam_G
FemGonads_b
MaleGonads_b
NeuralTube_b
NeuralTube_o
Epidermis_b
Muscle_b
Gut_b
Hepatic_b
Hepatic_o
Gills_b
Cirri_b
TopGO results
tRNA metabolic process
DNA metabolic process
response to fructose
nuclear pore complex assembly
CDP−diacylglycerol biosynthetic process
pore complex assembly
CDP−diacylglycerol metabolic process
regulation of cellular response to heat
tRNA processing
nuclear pore organization
structural constituent of nuclear pore
endonuclease activity
nucleic acid binding
endodeoxyribonuclease activity
calcium−dependent phospholipase A2 activ...
TFIID−class transcription factor binding
deoxyribonuclease activity
ubiquitin binding
single−stranded DNA endodeoxyribonucleas...
uracil DNA N−glycosylase activity
nuclear pore
pore complex
membrane−bounded organelle
intracellular membrane−bounded organelle
nuclear envelope
nucleus
nuclear membrane
nuclear replication fork
intracellular organelle
nuclear lumen
GO Categories
BP
MF
CC
0
1
2
3
4
5
log pvalues (correction=none)

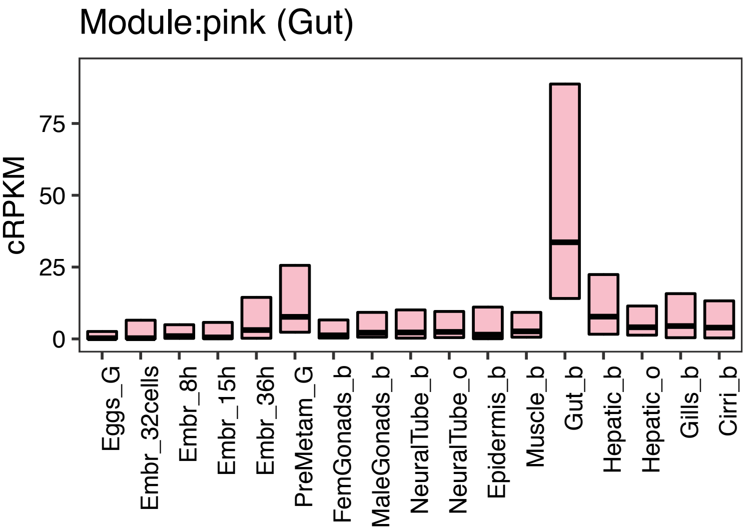
Module:pink (Gut)
cRPKM
0
25
50
75
Eggs_G
Embr_32cells
Embr_8h
Embr_15h
Embr_36h
PreMetam_G
FemGonads_b
MaleGonads_b
NeuralTube_b
NeuralTube_o
Epidermis_b
Muscle_b
Gut_b
Hepatic_b
Hepatic_o
Gills_b
Cirri_b

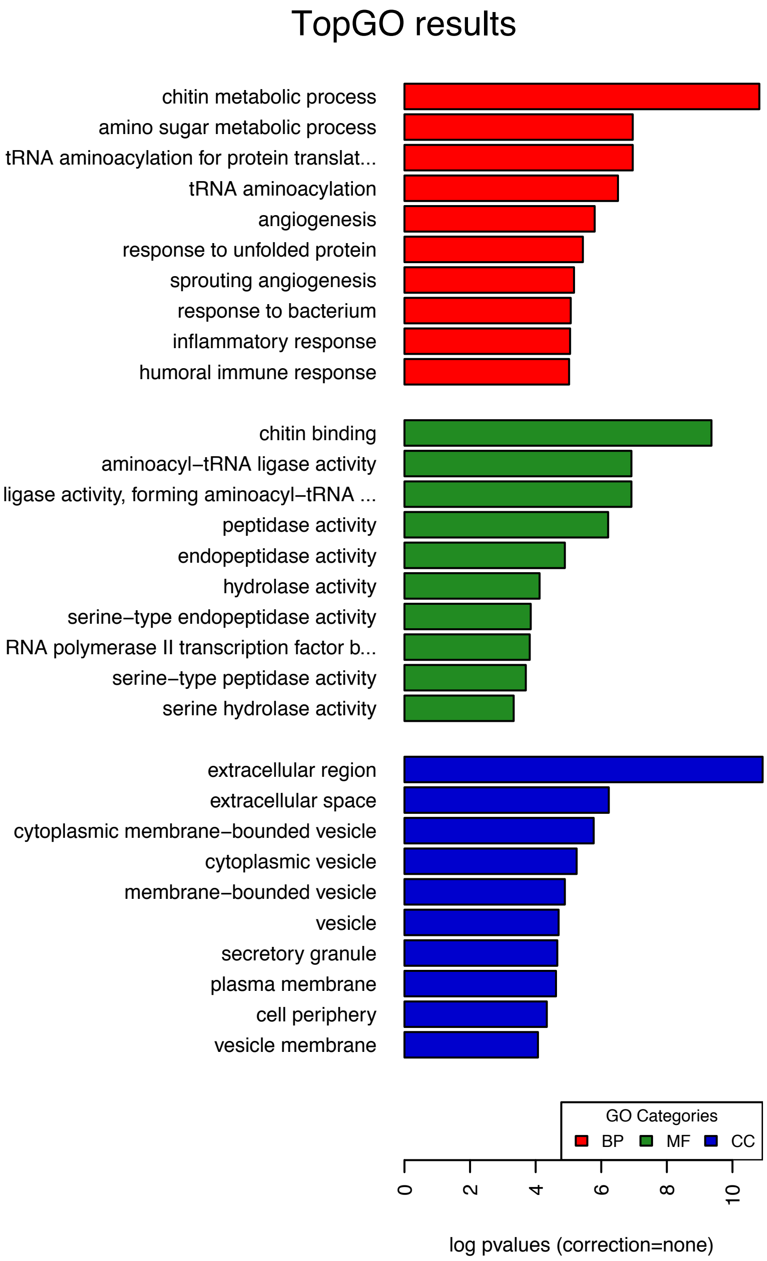
TopGO results
chitin metabolic process
amino sugar metabolic process
tRNA aminoacylation for protein translat...
tRNA aminoacylation
angiogenesis
response to unfolded protein
sprouting angiogenesis
response to bacterium
inflammatory response
humoral immune response
chitin binding
aminoacyl−tRNA ligase activity
ligase activity, forming aminoacyl−tRNA ...
peptidase activity
endopeptidase activity
hydrolase activity
serine−type endopeptidase activity
RNA polymerase II transcription factor b...
serine−type peptidase activity
serine hydrolase activity
extracellular region
extracellular space
cytoplasmic membrane−bounded vesicle
cytoplasmic vesicle
membrane−bounded vesicle
vesicle
secretory granule
plasma membrane
cell periphery
vesicle membrane
GO Categories
BP
MF
CC
0
2
4
6
8
10
log pvalues (correction=none)

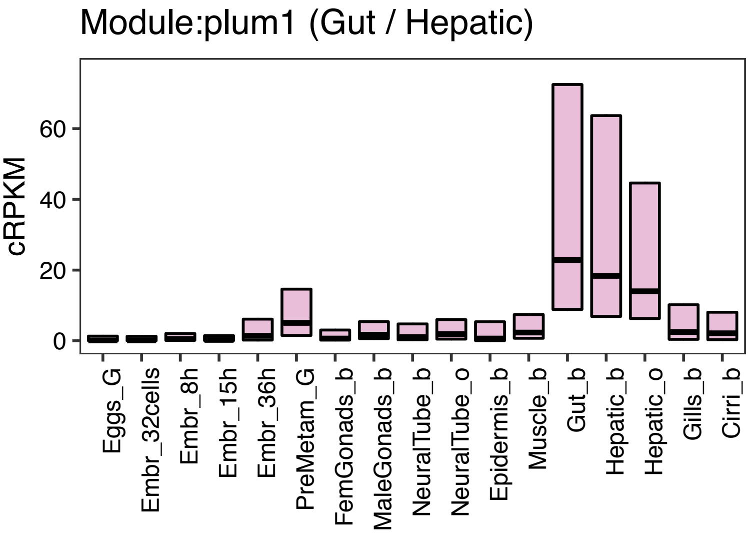
Module:plum1 (Gut / Hepatic)
cRPKM
0
20
40
60
Eggs_G
Embr_32cells
Embr_8h
Embr_15h
Embr_36h
PreMetam_G
FemGonads_b
MaleGonads_b
NeuralTube_b
NeuralTube_o
Epidermis_b
Muscle_b
Gut_b
Hepatic_b
Hepatic_o
Gills_b
Cirri_b

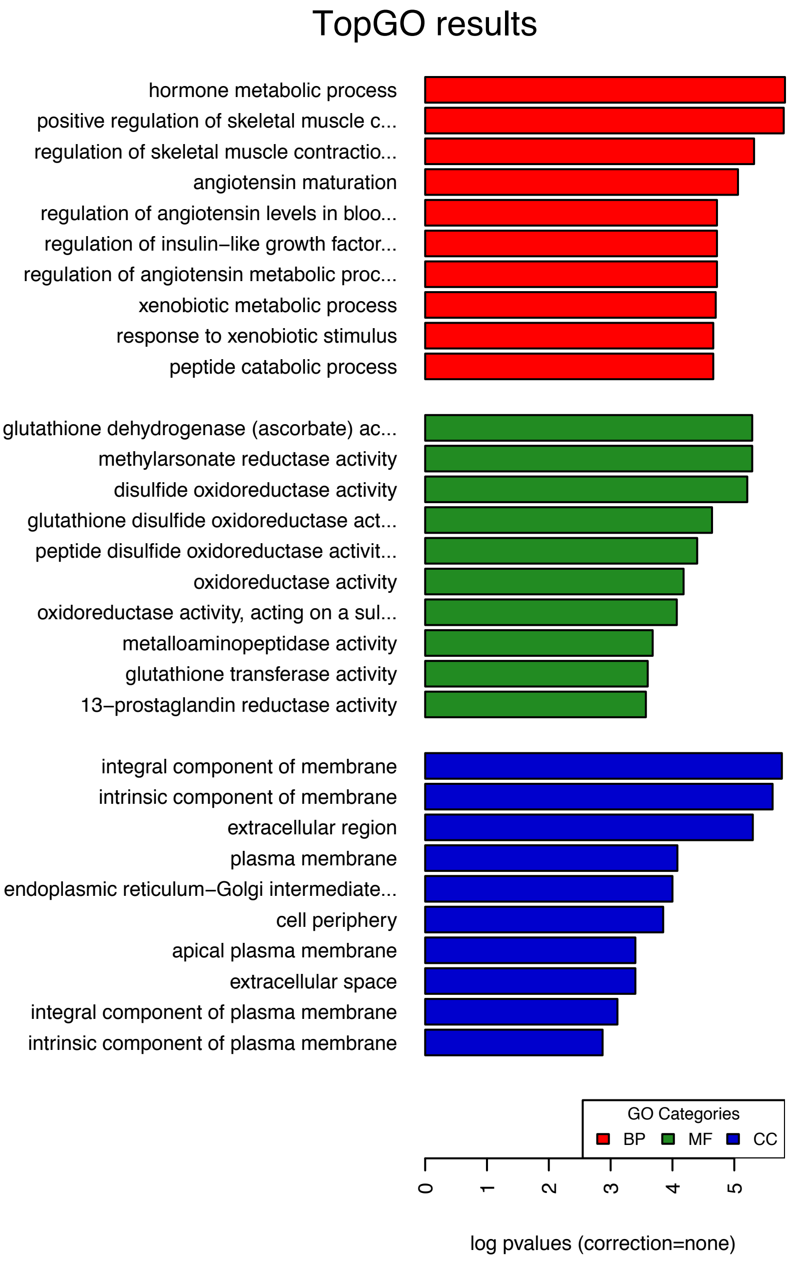
TopGO results
hormone metabolic process
positive regulation of skeletal muscle c...
regulation of skeletal muscle contractio...
angiotensin maturation
regulation of angiotensin levels in bloo...
regulation of insulin−like growth factor...
regulation of angiotensin metabolic proc...
xenobiotic metabolic process
response to xenobiotic stimulus
peptide catabolic process
glutathione dehydrogenase (ascorbate) ac...
methylarsonate reductase activity
disulfide oxidoreductase activity
glutathione disulfide oxidoreductase act...
peptide disulfide oxidoreductase activit...
oxidoreductase activity
oxidoreductase activity, acting on a sul...
metalloaminopeptidase activity
glutathione transferase activity
13−prostaglandin reductase activity
integral component of membrane
intrinsic component of membrane
extracellular region
plasma membrane
endoplasmic reticulum−Golgi intermediate...
cell periphery
apical plasma membrane
extracellular space
integral component of plasma membrane
intrinsic component of plasma membrane
GO Categories
BP
MF
CC
0
1
2
3
4
5
log pvalues (correction=none)

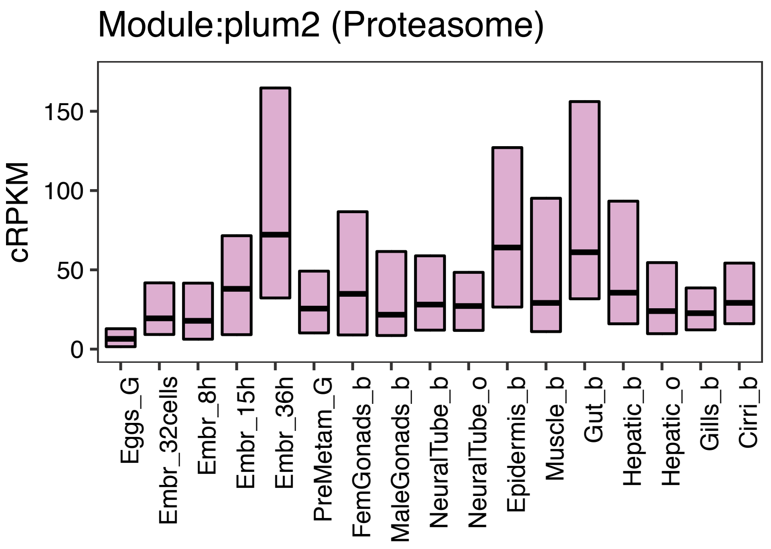
Module:plum2 (Proteasome)
cRPKM
0
50
100
150
Eggs_G
Embr_32cells
Embr_8h
Embr_15h
Embr_36h
PreMetam_G
FemGonads_b
MaleGonads_b
NeuralTube_b
NeuralTube_o
Epidermis_b
Muscle_b
Gut_b
Hepatic_b
Hepatic_o
Gills_b
Cirri_b

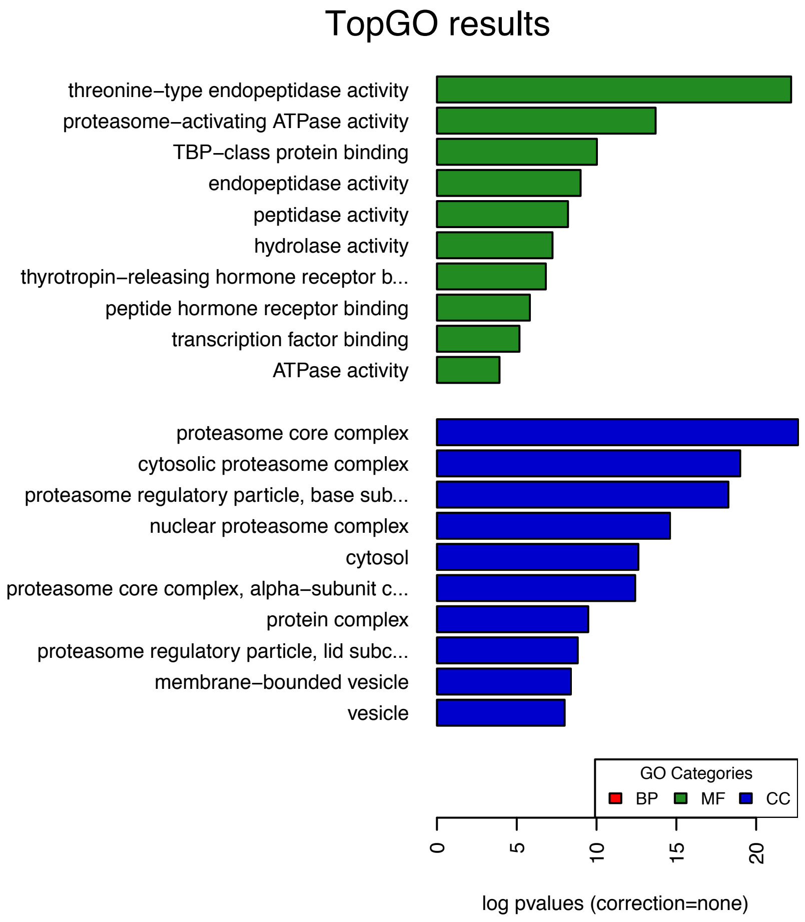
TopGO results
threonine−type endopeptidase activity
proteasome−activating ATPase activity
TBP−class protein binding
endopeptidase activity
peptidase activity
hydrolase activity
thyrotropin−releasing hormone receptor b...
peptide hormone receptor binding
transcription factor binding
ATPase activity
proteasome core complex
cytosolic proteasome complex
proteasome regulatory particle, base sub...
nuclear proteasome complex
cytosol
proteasome core complex, alpha−subunit c...
protein complex
proteasome regulatory particle, lid subc...
membrane−bounded vesicle
vesicle
GO Categories
BP
MF
CC
0
5
10
15
20
log pvalues (correction=none)

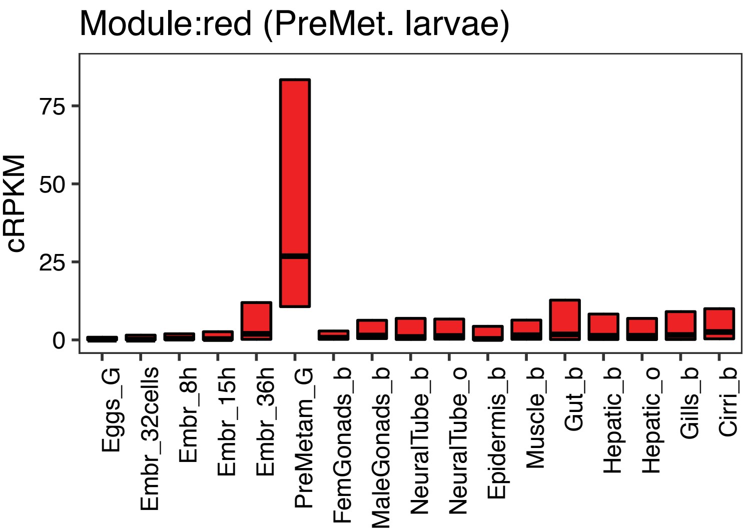
Module:red (PreMet. larvae)
cRPKM
75
50
25
0
Eggs_G
Embr_32cells
Embr_8h
Embr_15h
Embr_36h
PreMetam_G
FemGonads_b
MaleGonads_b
NeuralTube_b
NeuralTube_o
Epidermis_b
Muscle_b
Gut_b
Hepatic_b
Hepatic_o
Gills_b
Cirri_b

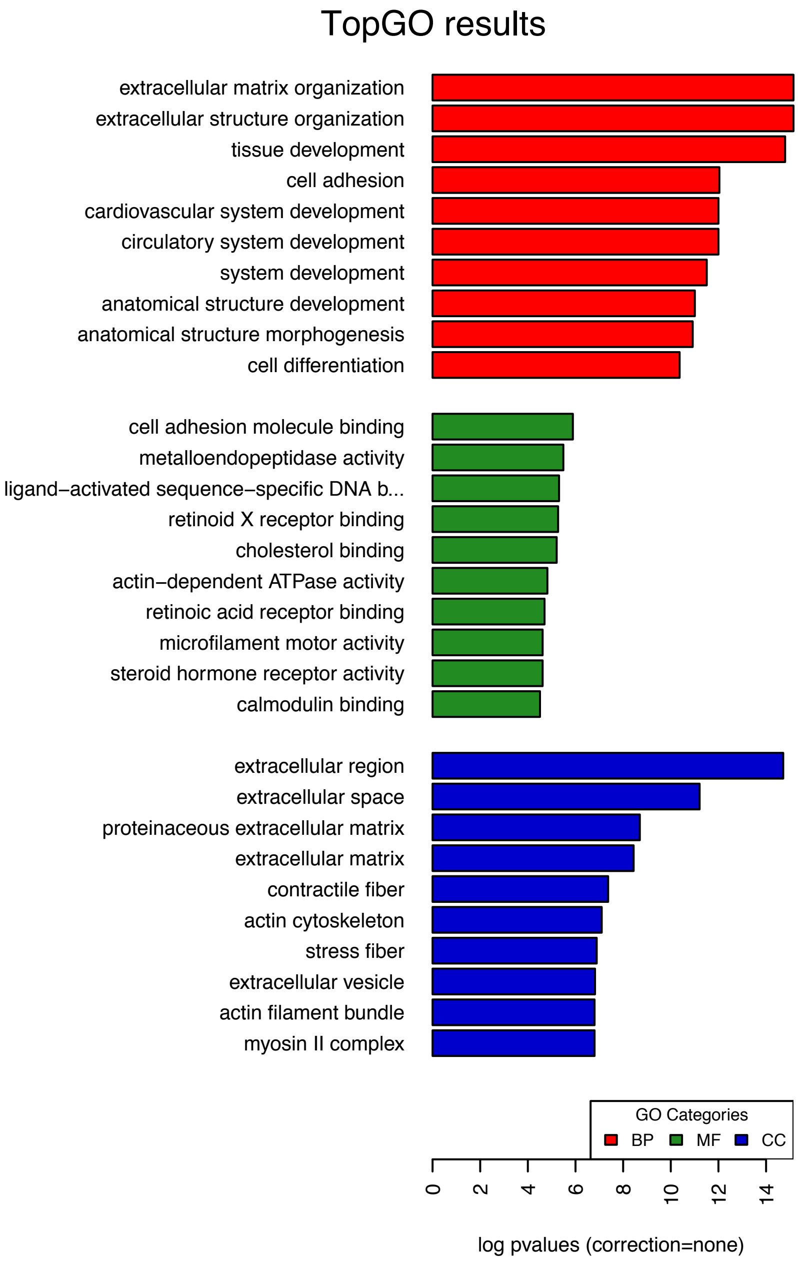
TopGO results
extracellular matrix organization
extracellular structure organization
tissue development
cell adhesion
cardiovascular system development
circulatory system development
system development
anatomical structure development
anatomical structure morphogenesis
cell differentiation
cell adhesion molecule binding
metalloendopeptidase activity
ligand−activated sequence−specific DNA b...
retinoid X receptor binding
cholesterol binding
actin−dependent ATPase activity
retinoic acid receptor binding
microfilament motor activity
steroid hormone receptor activity
calmodulin binding
extracellular region
extracellular space
proteinaceous extracellular matrix
extracellular matrix
contractile fiber
actin cytoskeleton
stress fiber
extracellular vesicle
actin filament bundle
myosin II complex
GO Categories
BP
MF
CC
0
2
4
6
8
10
12
14
log pvalues (correction=none)

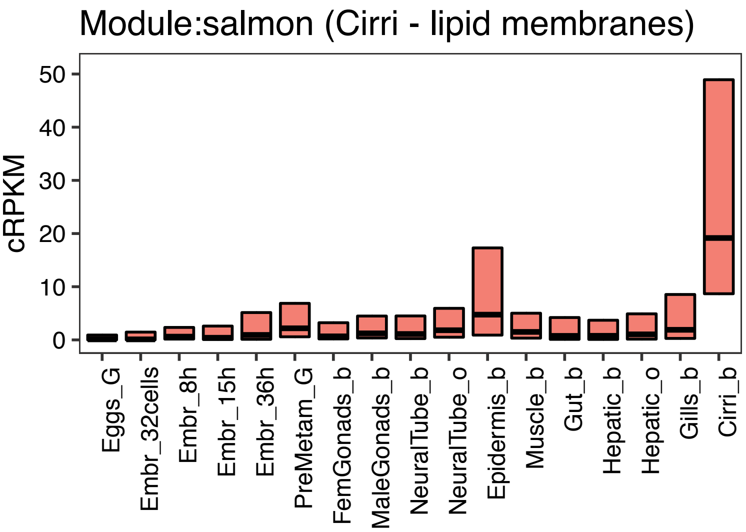
Module:salmon (Cirri - lipid membranes)
cRPKM
0
10
20
30
40
50
Eggs_G
Embr_32cells
Embr_8h
Embr_15h
Embr_36h
PreMetam_G
FemGonads_b
MaleGonads_b
NeuralTube_b
NeuralTube_o
Epidermis_b
Muscle_b
Gut_b
Hepatic_b
Hepatic_o
Gills_b
Cirri_b

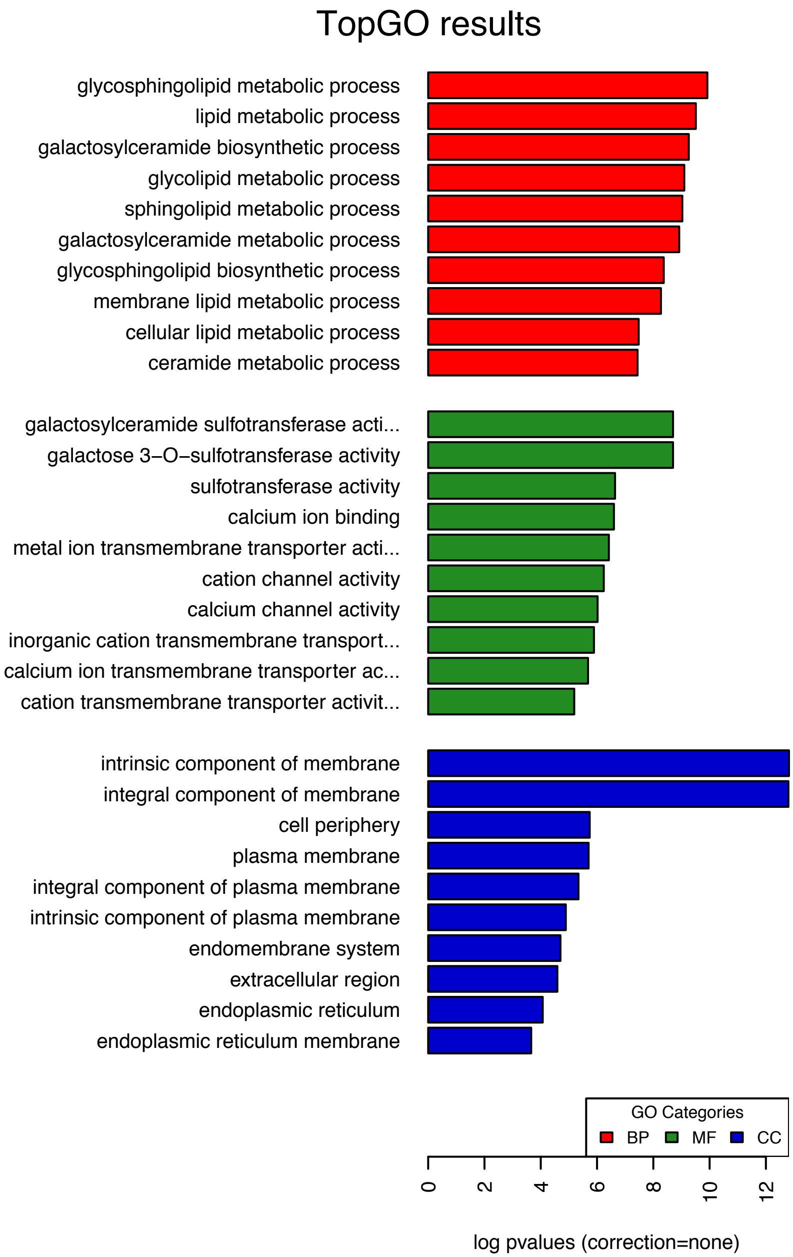
TopGO results
glycosphingolipid metabolic process
lipid metabolic process
galactosylceramide biosynthetic process
glycolipid metabolic process
sphingolipid metabolic process
galactosylceramide metabolic process
glycosphingolipid biosynthetic process
membrane lipid metabolic process
cellular lipid metabolic process
ceramide metabolic process
galactosylceramide sulfotransferase acti...
galactose 3−O−sulfotransferase activity
sulfotransferase activity
calcium ion binding
metal ion transmembrane transporter acti...
cation channel activity
calcium channel activity
inorganic cation transmembrane transport...
calcium ion transmembrane transporter ac...
cation transmembrane transporter activit...
intrinsic component of membrane
integral component of membrane
cell periphery
plasma membrane
integral component of plasma membrane
intrinsic component of plasma membrane
endomembrane system
extracellular region
endoplasmic reticulum
endoplasmic reticulum membrane
GO Categories
BP
MF
CC
0
2
4
6
8
10
12
log pvalues (correction=none)

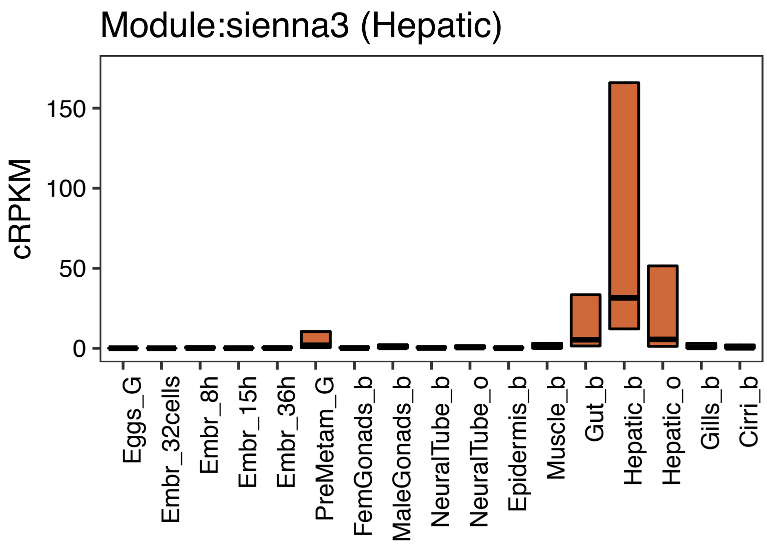

Module:sienna3 (Hepatic)
cRPKM
150
100
50
0
Eggs_G
Embr_32cells
Embr_8h
Embr_15h
Embr_36h
PreMetam_G
FemGonads_b
MaleGonads_b
NeuralTube_b
NeuralTube_o
Epidermis_b
Muscle_b
Gut_b
Hepatic_b
Hepatic_o
Gills_b
Cirri_b

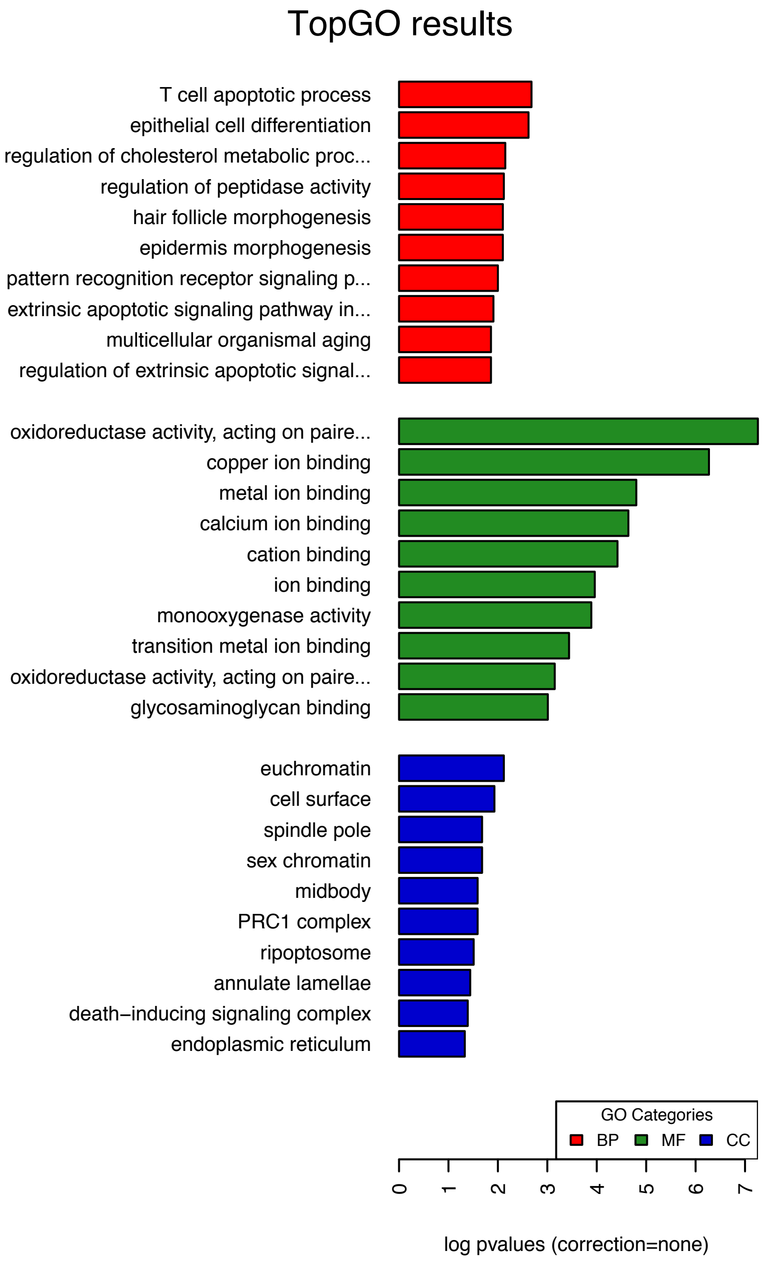

TopGO results
T cell apoptotic process
epithelial cell differentiation
regulation of cholesterol metabolic proc...
regulation of peptidase activity
hair follicle morphogenesis
epidermis morphogenesis
pattern recognition receptor signaling p...
extrinsic apoptotic signaling pathway in...
multicellular organismal aging
regulation of extrinsic apoptotic signal...
oxidoreductase activity, acting on paire...
copper ion binding
metal ion binding
calcium ion binding
cation binding
ion binding
monooxygenase activity
transition metal ion binding
oxidoreductase activity, acting on paire...
glycosaminoglycan binding
euchromatin
cell surface
spindle pole
sex chromatin
midbody
PRC1 complex
ripoptosome
annulate lamellae
death−inducing signaling complex
endoplasmic reticulum
GO Categories
BP
MF
CC
0
1
2
3
4
5
6
7
log pvalues (correction=none)

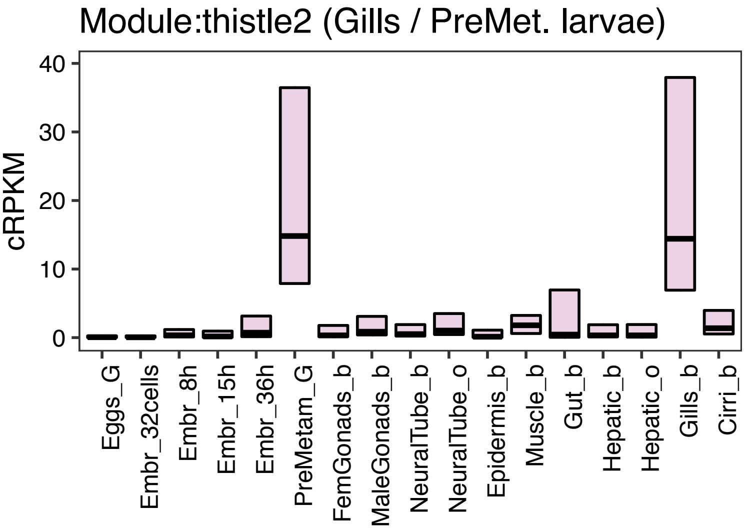
Module:thistle2 (Gills / PreMet. larvae)
cRPKM
0
10
20
30
40
Eggs_G
Embr_32cells
Embr_8h
Embr_15h
Embr_36h
PreMetam_G
FemGonads_b
MaleGonads_b
NeuralTube_b
NeuralTube_o
Epidermis_b
Muscle_b
Gut_b
Hepatic_b
Hepatic_o
Gills_b
Cirri_b

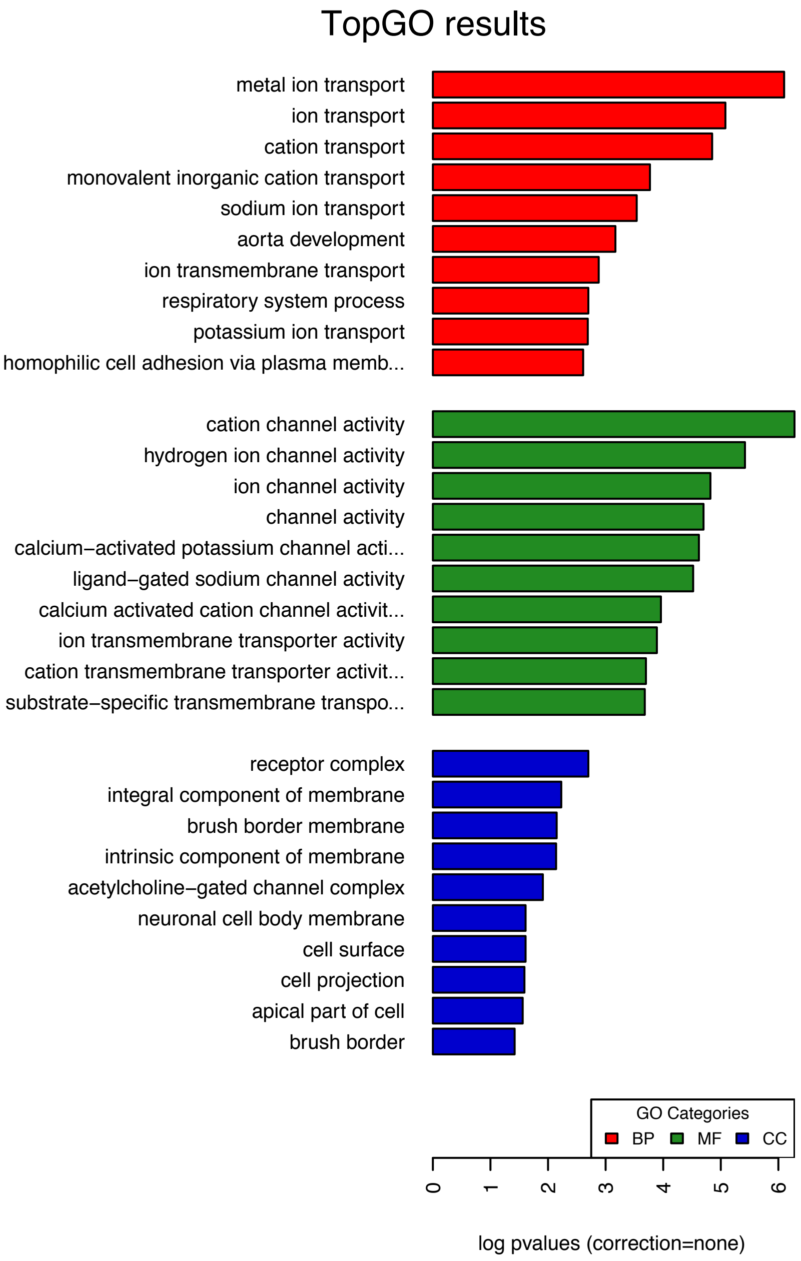
TopGO results
metal ion transport
ion transport
cation transport
monovalent inorganic cation transport
sodium ion transport
aorta development
ion transmembrane transport
respiratory system process
potassium ion transport
homophilic cell adhesion via plasma memb...
cation channel activity
hydrogen ion channel activity
ion channel activity
channel activity
calcium−activated potassium channel acti...
ligand−gated sodium channel activity
calcium activated cation channel activit...
ion transmembrane transporter activity
cation transmembrane transporter activit...
substrate−specific transmembrane transpo...
receptor complex
integral component of membrane
brush border membrane
intrinsic component of membrane
acetylcholine−gated channel complex
neuronal cell body membrane
cell surface
cell projection
apical part of cell
brush border
GO Categories
BP
MF
CC
0
1
2
3
4
5
6
log pvalues (correction=none)

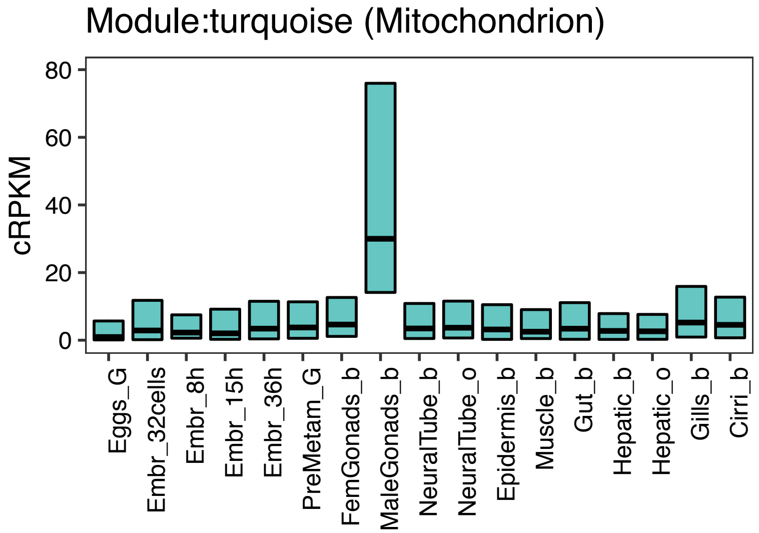
Module:turquoise (Mitochondrion)
cRPKM
0
20
40
60
80
Eggs_G
Embr_32cells
Embr_8h
Embr_15h
Embr_36h
PreMetam_G
FemGonads_b
MaleGonads_b
NeuralTube_b
NeuralTube_o
Epidermis_b
Muscle_b
Gut_b
Hepatic_b
Hepatic_o
Gills_b
Cirri_b

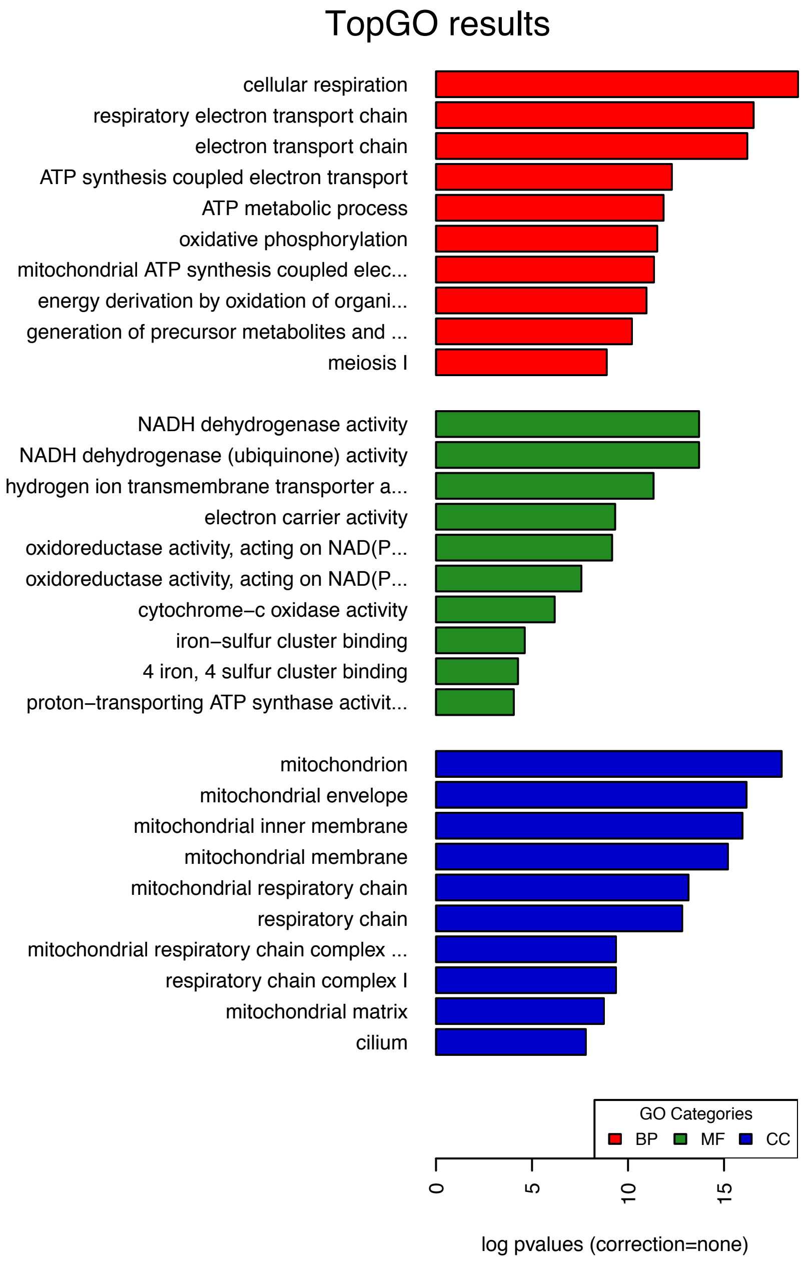
TopGO results
cellular respiration
respiratory electron transport chain
electron transport chain
ATP synthesis coupled electron transport
ATP metabolic process
oxidative phosphorylation
mitochondrial ATP synthesis coupled elec...
energy derivation by oxidation of organi...
generation of precursor metabolites and ...
meiosis I
NADH dehydrogenase activity
NADH dehydrogenase (ubiquinone) activity
hydrogen ion transmembrane transporter a...
electron carrier activity
oxidoreductase activity, acting on NAD(P...
oxidoreductase activity, acting on NAD(P...
cytochrome−c oxidase activity
iron−sulfur cluster binding
4 iron, 4 sulfur cluster binding
proton−transporting ATP synthase activit...
mitochondrion
mitochondrial envelope
mitochondrial inner membrane
mitochondrial membrane
mitochondrial respiratory chain
respiratory chain
mitochondrial respiratory chain complex ...
respiratory chain complex I
mitochondrial matrix
cilium
GO Categories
BP
MF
CC
0
5
10
15
log pvalues (correction=none)

D. rerio modules

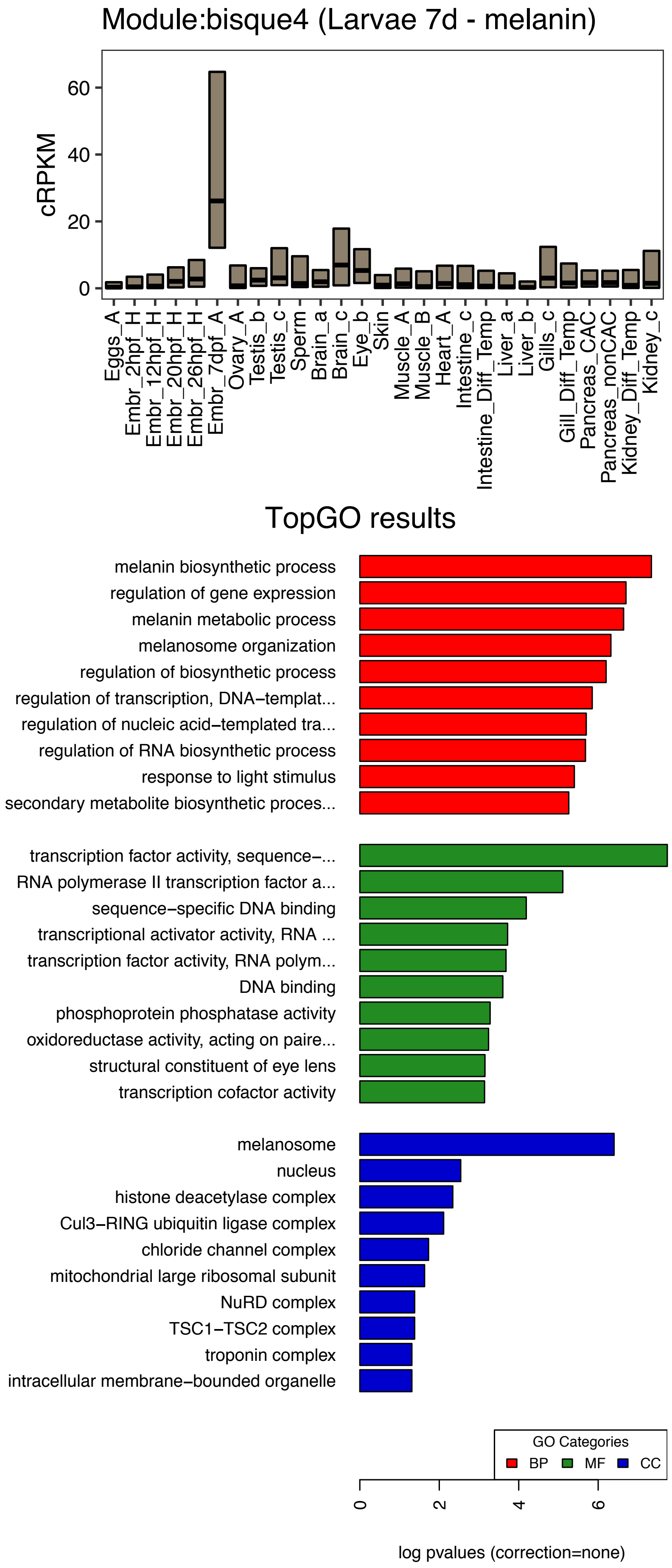

Module:bisque4 (Larvae 7d - melanin)
cRPKM
0
20
40
60
Eggs_A
Embr_2hpf_H
Embr_12hpf_H
Embr_20hpf_H
Embr_26hpf_H
Embr_7dpf_A
Ovary_A
Testis_b
Testis_c
Sperm
Brain_a
Brain_c
Eye_b
Skin
Muscle_A
Muscle_B
Heart_A
Intestine_c
Intestine_Diff_Temp
Liver_a
Liver_b
Gills_c
Gill_Diff_Temp
Pancreas_CAC
Pancreas_nonCAC
Kidney_Diff_Temp
Kidney_c
TopGO results
melanin biosynthetic process
regulation of gene expression
melanin metabolic process
melanosome organization
regulation of biosynthetic process
regulation of transcription, DNA−templat...
regulation of nucleic acid−templated tra...
regulation of RNA biosynthetic process
response to light stimulus
secondary metabolite biosynthetic proces...
transcription factor activity, sequence−...
RNA polymerase II transcription factor a...
sequence−specific DNA binding
transcriptional activator activity, RNA ...
transcription factor activity, RNA polym...
DNA binding
phosphoprotein phosphatase activity
oxidoreductase activity, acting on paire...
structural constituent of eye lens
transcription cofactor activity
melanosome
nucleus
histone deacetylase complex
Cul3−RING ubiquitin ligase complex
chloride channel complex
mitochondrial large ribosomal subunit
NuRD complex
TSC1−TSC2 complex
troponin complex
intracellular membrane−bounded organelle
GO Categories
BP
MF
CC
0
2
4
6
log pvalues (correction=none)

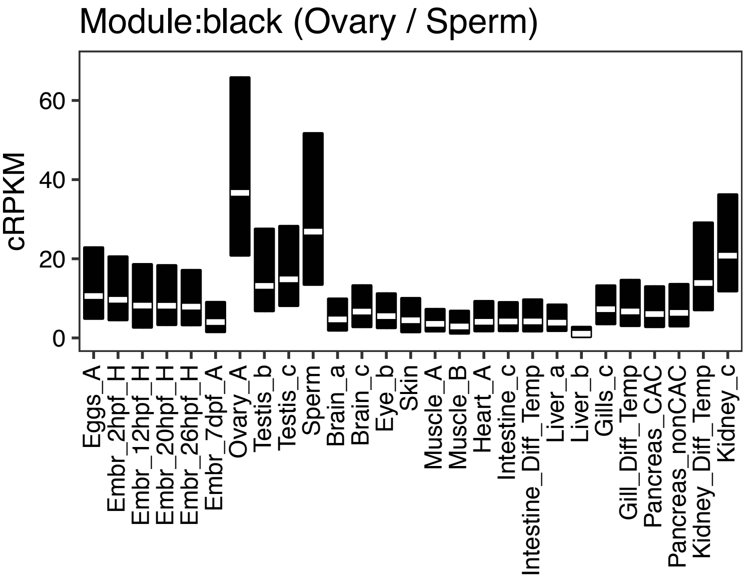
Module:black (Ovary / Sperm)
cRPKM
0
20
40
60
Eggs_A
Embr_2hpf_H
Embr_12hpf_H
Embr_20hpf_H
Embr_26hpf_H
Embr_7dpf_A
Ovary_A
Testis_b
Testis_c
Sperm
Brain_a
Brain_c
Eye_b
Skin
Muscle_A
Muscle_B
Heart_A
Intestine_c
Intestine_Diff_Temp
Liver_a
Liver_b
Gills_c
Gill_Diff_Temp
Pancreas_CAC
Pancreas_nonCAC
Kidney_Diff_Temp
Kidney_c

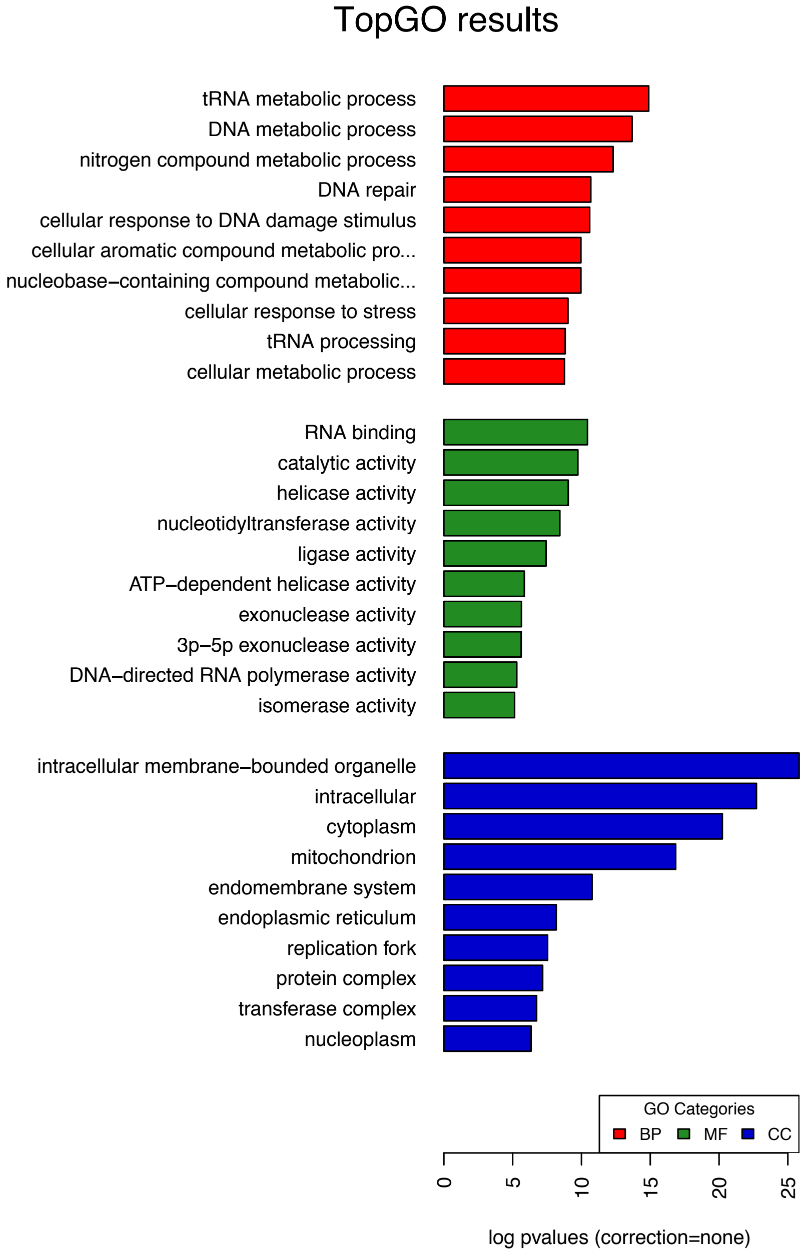
TopGO results
tRNA metabolic process
DNA metabolic process
nitrogen compound metabolic process
DNA repair
cellular response to DNA damage stimulus
cellular aromatic compound metabolic pro...
nucleobase−containing compound metabolic...
cellular response to stress
tRNA processing
cellular metabolic process
RNA binding
catalytic activity
helicase activity
nucleotidyltransferase activity
ligase activity
ATP−dependent helicase activity
exonuclease activity
3p−5p exonuclease activity
DNA−directed RNA polymerase activity
isomerase activity
intracellular membrane−bounded organelle
intracellular
cytoplasm
mitochondrion
endomembrane system
endoplasmic reticulum
replication fork
protein complex
transferase complex
nucleoplasm
GO Categories
BP
MF
CC
0
5
10
15
20
25
log pvalues (correction=none)

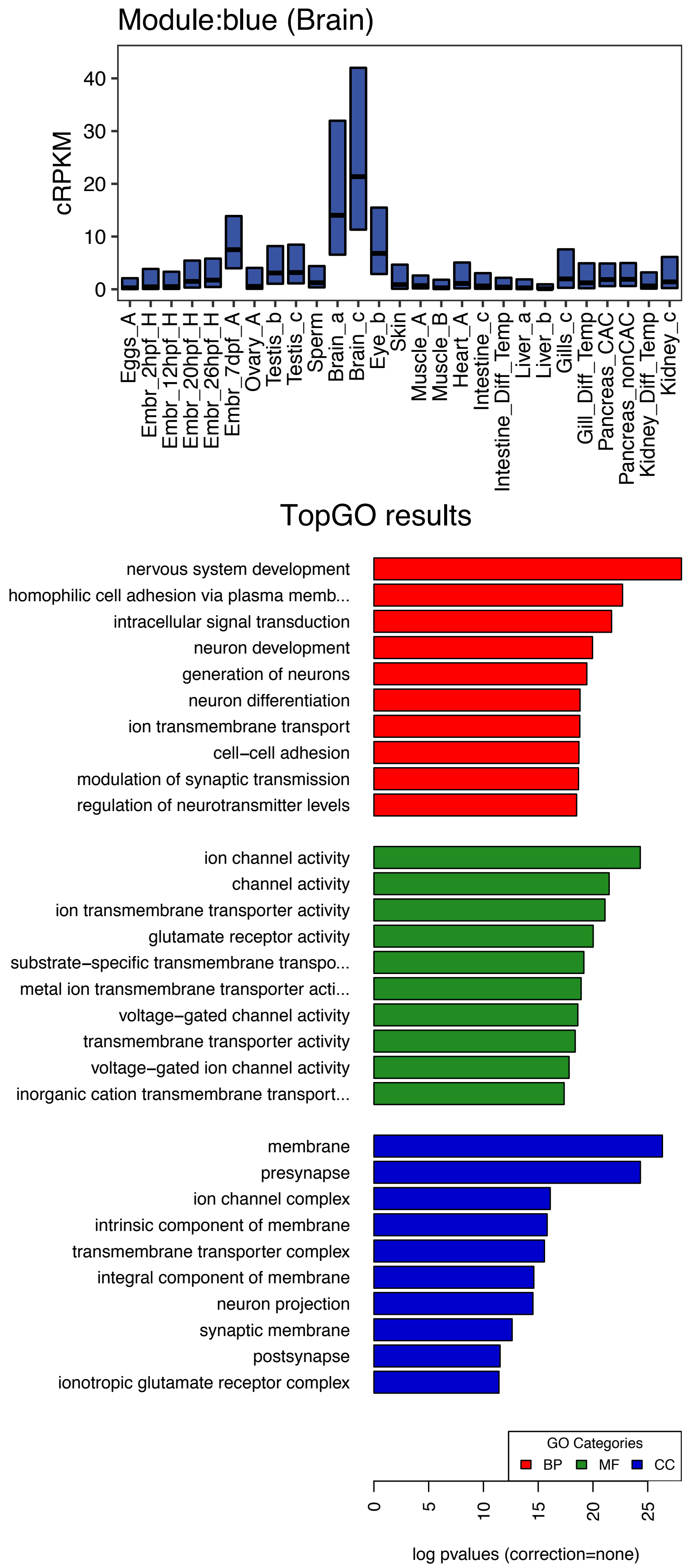
Module:blue (Brain)
cRPKM
0
10
20
30
40
Eggs_A
Embr_2hpf_H
Embr_12hpf_H
Embr_20hpf_H
Embr_26hpf_H
Embr_7dpf_A
Ovary_A
Testis_b
Testis_c
Sperm
Brain_a
Brain_c
Eye_b
Skin
Muscle_A
Muscle_B
Heart_A
Intestine_c
Intestine_Diff_Temp
Liver_a
Liver_b
Gills_c
Gill_Diff_Temp
Pancreas_CAC
Pancreas_nonCAC
Kidney_Diff_Temp
Kidney_c
TopGO results
nervous system development
homophilic cell adhesion via plasma memb...
intracellular signal transduction
neuron development
generation of neurons
neuron differentiation
ion transmembrane transport
cell−cell adhesion
modulation of synaptic transmission
regulation of neurotransmitter levels
ion channel activity
channel activity
ion transmembrane transporter activity
glutamate receptor activity
substrate−specific transmembrane transpo...
metal ion transmembrane transporter acti...
voltage−gated channel activity
transmembrane transporter activity
voltage−gated ion channel activity
inorganic cation transmembrane transport...
membrane
presynapse
ion channel complex
intrinsic component of membrane
transmembrane transporter complex
integral component of membrane
neuron projection
synaptic membrane
postsynapse
ionotropic glutamate receptor complex
GO Categories
BP
MF
CC
0
5
10
15
20
25
log pvalues (correction=none)

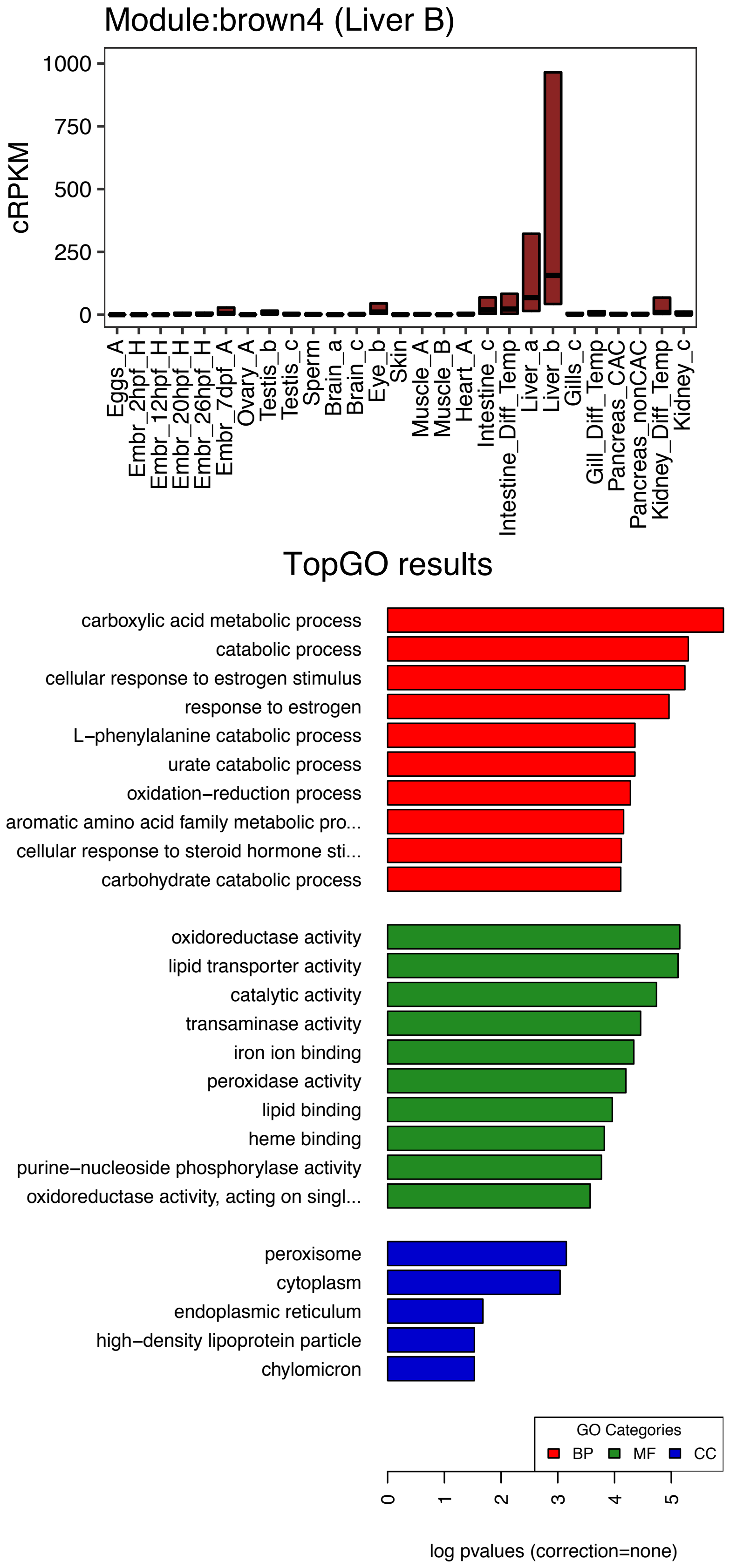
Module:brown4 (Liver B)
cRPKM
1000
750
500
250
0
Eggs_A
Embr_2hpf_H
Embr_12hpf_H
Embr_20hpf_H
Embr_26hpf_H
Embr_7dpf_A
Ovary_A
Testis_b
Testis_c
Sperm
Brain_a
Brain_c
Eye_b
Skin
Muscle_A
Muscle_B
Heart_A
Intestine_c
Intestine_Diff_Temp
Liver_a
Liver_b
Gills_c
Gill_Diff_Temp
Pancreas_CAC
Pancreas_nonCAC
Kidney_Diff_Temp
Kidney_c
TopGO results
carboxylic acid metabolic process
catabolic process
cellular response to estrogen stimulus
response to estrogen
L−phenylalanine catabolic process
urate catabolic process
oxidation−reduction process
aromatic amino acid family metabolic pro...
cellular response to steroid hormone sti...
carbohydrate catabolic process
oxidoreductase activity
lipid transporter activity
catalytic activity
transaminase activity
iron ion binding
peroxidase activity
lipid binding
heme binding
purine−nucleoside phosphorylase activity
oxidoreductase activity, acting on singl...
peroxisome
cytoplasm
endoplasmic reticulum
high−density lipoprotein particle
chylomicron
GO Categories
BP
MF
CC
0
1
2
3
4
5
log pvalues (correction=none)

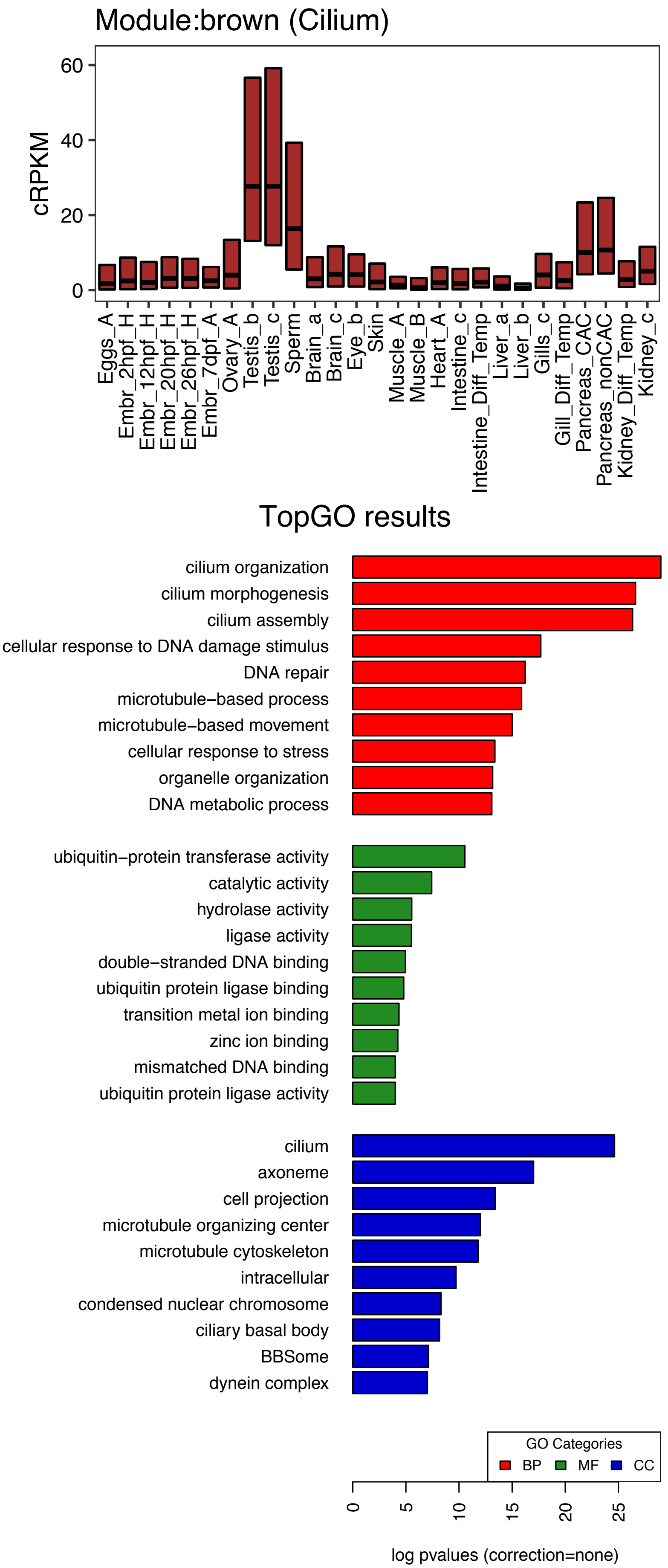

Module:brown (Cilium)
cRPKM
60
40
20
0
Eggs_A
Embr_2hpf_H
Embr_12hpf_H
Embr_20hpf_H
Embr_26hpf_H
Embr_7dpf_A
Ovary_A
Testis_b
Testis_c
Sperm
Brain_a
Brain_c
Eye_b
Skin
Muscle_A
Muscle_B
Heart_A
Intestine_c
Intestine_Diff_Temp
Liver_a
Liver_b
Gills_c
Gill_Diff_Temp
Pancreas_CAC
Pancreas_nonCAC
Kidney_Diff_Temp
Kidney_c
TopGO results
cilium organization
cilium morphogenesis
cilium assembly
cellular response to DNA damage stimulus
DNA repair
microtubule−based process
microtubule−based movement
cellular response to stress
organelle organization
DNA metabolic process
ubiquitin−protein transferase activity
catalytic activity
hydrolase activity
ligase activity
double−stranded DNA binding
ubiquitin protein ligase binding
transition metal ion binding
zinc ion binding
mismatched DNA binding
ubiquitin protein ligase activity
cilium
axoneme
cell projection
microtubule organizing center
microtubule cytoskeleton
intracellular
condensed nuclear chromosome
ciliary basal body
BBSome
dynein complex
GO Categories
BP
MF
CC
0
5
10
15
20
25
log pvalues (correction=none)

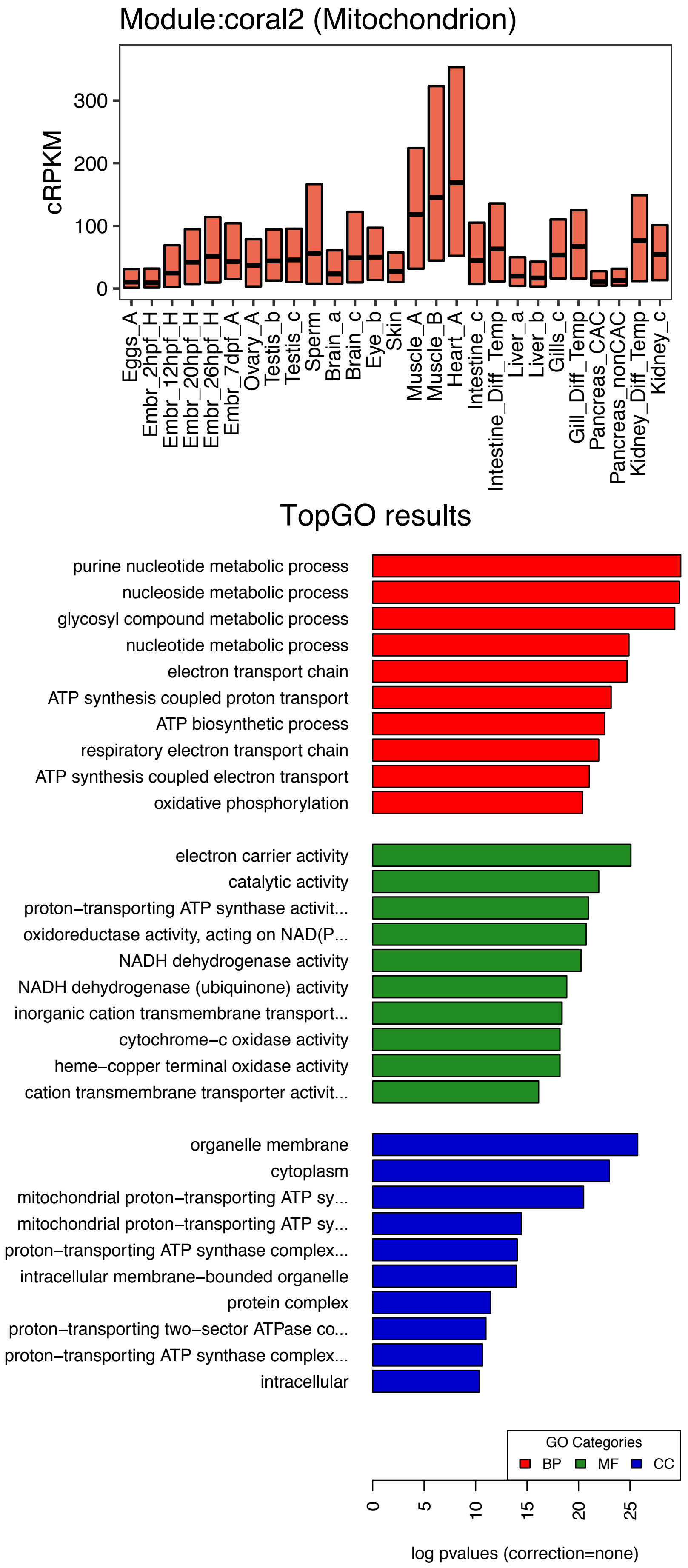

Module:coral2 (Mitochondrion)
cRPKM
0
100
200
300
Eggs_A
Embr_2hpf_H
Embr_12hpf_H
Embr_20hpf_H
Embr_26hpf_H
Embr_7dpf_A
Ovary_A
Testis_b
Testis_c
Sperm
Brain_a
Brain_c
Eye_b
Skin
Muscle_A
Muscle_B
Heart_A
Intestine_c
Intestine_Diff_Temp
Liver_a
Liver_b
Gills_c
Gill_Diff_Temp
Pancreas_CAC
Pancreas_nonCAC
Kidney_Diff_Temp
Kidney_c
TopGO results
purine nucleotide metabolic process
nucleoside metabolic process
glycosyl compound metabolic process
nucleotide metabolic process
electron transport chain
ATP synthesis coupled proton transport
ATP biosynthetic process
respiratory electron transport chain
ATP synthesis coupled electron transport
oxidative phosphorylation
electron carrier activity
catalytic activity
proton−transporting ATP synthase activit...
oxidoreductase activity, acting on NAD(P...
NADH dehydrogenase activity
NADH dehydrogenase (ubiquinone) activity
inorganic cation transmembrane transport...
cytochrome−c oxidase activity
heme−copper terminal oxidase activity
cation transmembrane transporter activit...
organelle membrane
cytoplasm
mitochondrial proton−transporting ATP sy...
mitochondrial proton−transporting ATP sy...
proton−transporting ATP synthase complex...
intracellular membrane−bounded organelle
protein complex
proton−transporting two−sector ATPase co...
proton−transporting ATP synthase complex...
intracellular
GO Categories
BP
MF
CC
0
5
10
15
20
25
log pvalues (correction=none)

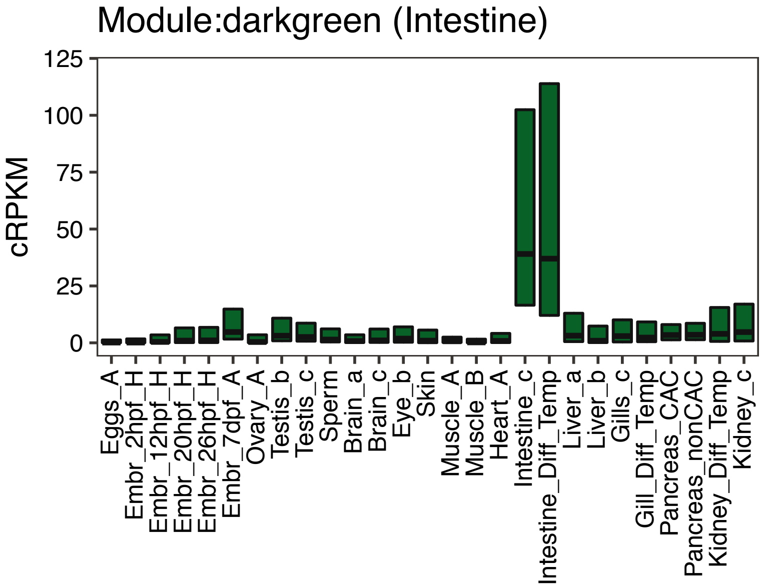
Module:darkgreen (Intestine)
cRPKM
125
100
75
50
25
0
Eggs_A
Embr_2hpf_H
Embr_12hpf_H
Embr_20hpf_H
Embr_26hpf_H
Embr_7dpf_A
Ovary_A
Testis_b
Testis_c
Sperm
Brain_a
Brain_c
Eye_b
Skin
Muscle_A
Muscle_B
Heart_A
Intestine_c
Intestine_Diff_Temp
Liver_a
Liver_b
Gills_c
Gill_Diff_Temp
Pancreas_CAC
Pancreas_nonCAC
Kidney_Diff_Temp
Kidney_c

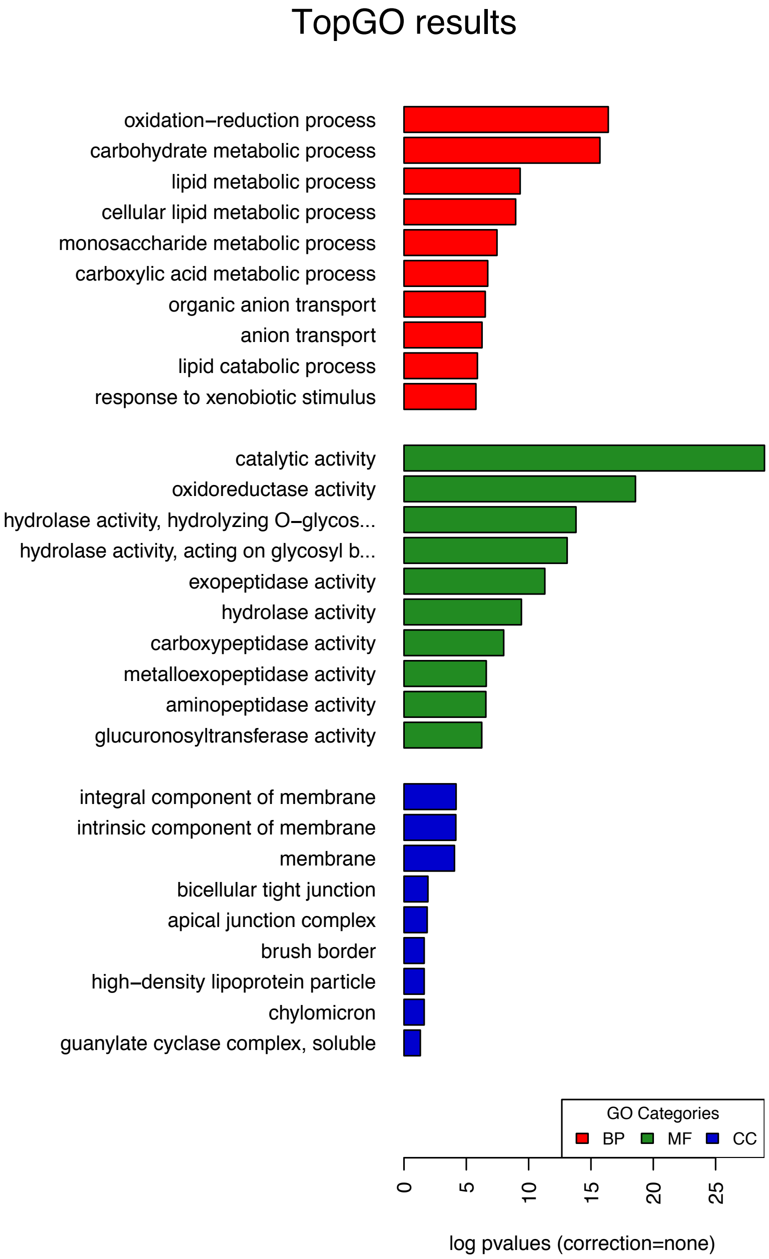
TopGO results
oxidation−reduction process
carbohydrate metabolic process
lipid metabolic process
cellular lipid metabolic process
monosaccharide metabolic process
carboxylic acid metabolic process
organic anion transport
anion transport
lipid catabolic process
response to xenobiotic stimulus
catalytic activity
oxidoreductase activity
hydrolase activity, hydrolyzing O−glycos...
hydrolase activity, acting on glycosyl b...
exopeptidase activity
hydrolase activity
carboxypeptidase activity
metalloexopeptidase activity
aminopeptidase activity
glucuronosyltransferase activity
integral component of membrane
intrinsic component of membrane
membrane
bicellular tight junction
apical junction complex
brush border
high−density lipoprotein particle
chylomicron
guanylate cyclase complex, soluble
GO Categories
BP
MF
CC
0
5
10
15
20
25
log pvalues (correction=none)

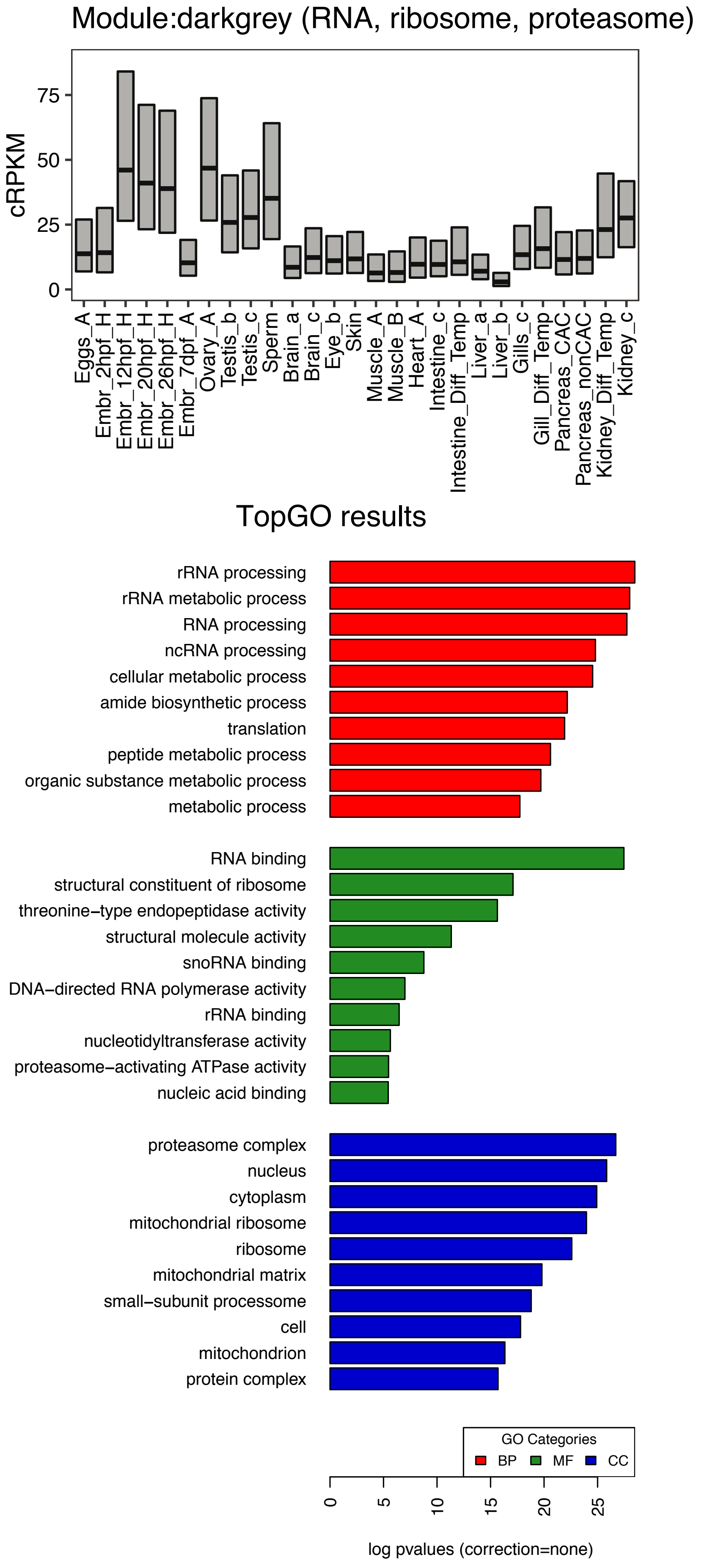
Module:darkgrey (RNA, ribosome, proteasome)
cRPKM
0
25
50
75
Eggs_A
Embr_2hpf_H
Embr_12hpf_H
Embr_20hpf_H
Embr_26hpf_H
Embr_7dpf_A
Ovary_A
Testis_b
Testis_c
Sperm
Brain_a
Brain_c
Eye_b
Skin
Muscle_A
Muscle_B
Heart_A
Intestine_c
Intestine_Diff_Temp
Liver_a
Liver_b
Gills_c
Gill_Diff_Temp
Pancreas_CAC
Pancreas_nonCAC
Kidney_Diff_Temp
Kidney_c
TopGO results
rRNA processing
rRNA metabolic process
RNA processing
ncRNA processing
cellular metabolic process
amide biosynthetic process
translation
peptide metabolic process
organic substance metabolic process
metabolic process
RNA binding
structural constituent of ribosome
threonine−type endopeptidase activity
structural molecule activity
snoRNA binding
DNA−directed RNA polymerase activity
rRNA binding
nucleotidyltransferase activity
proteasome−activating ATPase activity
nucleic acid binding
proteasome complex
nucleus
cytoplasm
mitochondrial ribosome
ribosome
mitochondrial matrix
small−subunit processome
cell
mitochondrion
protein complex
GO Categories
BP
MF
CC
0
5
10
15
20
25
log pvalues (correction=none)

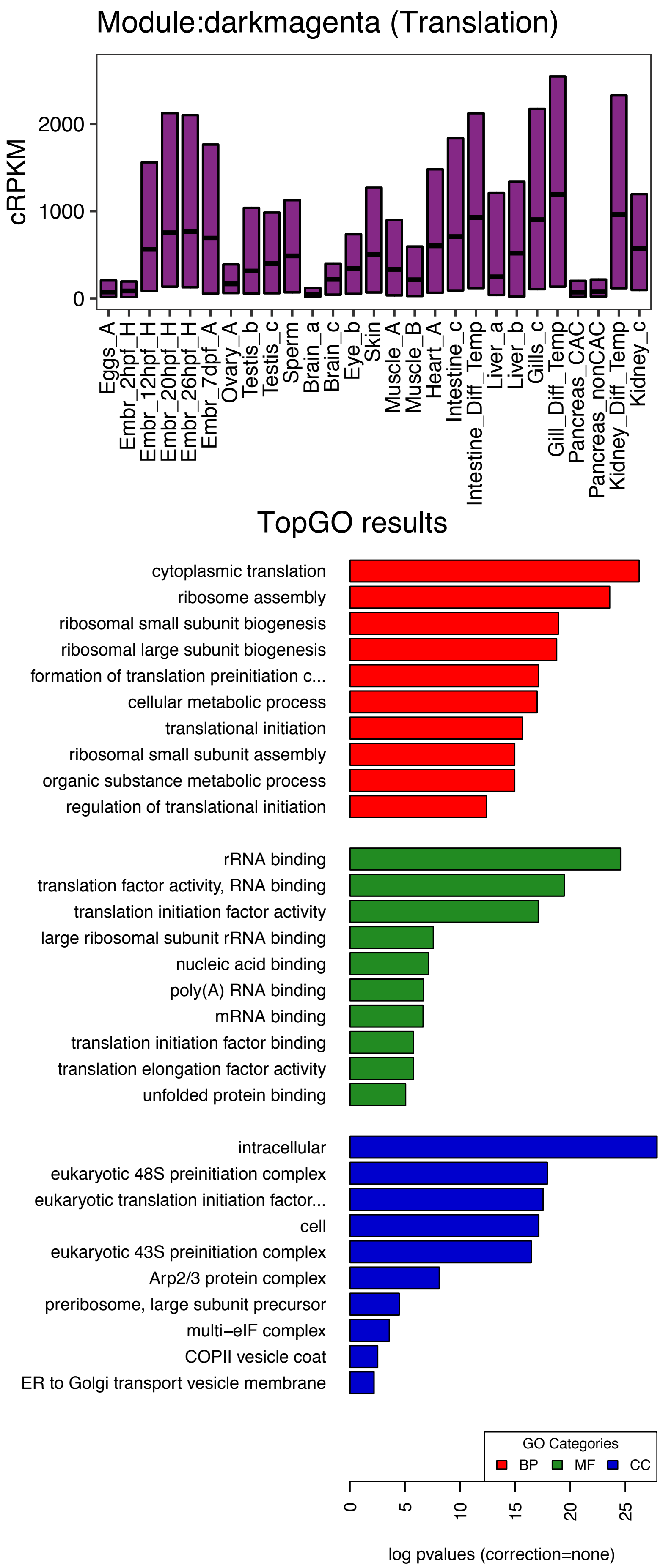
Module:darkmagenta (Translation)
cRPKM
0
1000
2000
Eggs_A
Embr_2hpf_H
Embr_12hpf_H
Embr_20hpf_H
Embr_26hpf_H
Embr_7dpf_A
Ovary_A
Testis_b
Testis_c
Sperm
Brain_a
Brain_c
Eye_b
Skin
Muscle_A
Muscle_B
Heart_A
Intestine_c
Intestine_Diff_Temp
Liver_a
Liver_b
Gills_c
Gill_Diff_Temp
Pancreas_CAC
Pancreas_nonCAC
Kidney_Diff_Temp
Kidney_c
TopGO results
cytoplasmic translation
ribosome assembly
ribosomal small subunit biogenesis
ribosomal large subunit biogenesis
formation of translation preinitiation c...
cellular metabolic process
translational initiation
ribosomal small subunit assembly
organic substance metabolic process
regulation of translational initiation
rRNA binding
translation factor activity, RNA binding
translation initiation factor activity
large ribosomal subunit rRNA binding
nucleic acid binding
poly(A) RNA binding
mRNA binding
translation initiation factor binding
translation elongation factor activity
unfolded protein binding
intracellular
eukaryotic 48S preinitiation complex
eukaryotic translation initiation factor...
cell
eukaryotic 43S preinitiation complex
Arp2/3 protein complex
preribosome, large subunit precursor
multi−eIF complex
COPII vesicle coat
ER to Golgi transport vesicle membrane
GO Categories
BP
MF
CC
0
5
10
15
20
25
log pvalues (correction=none)

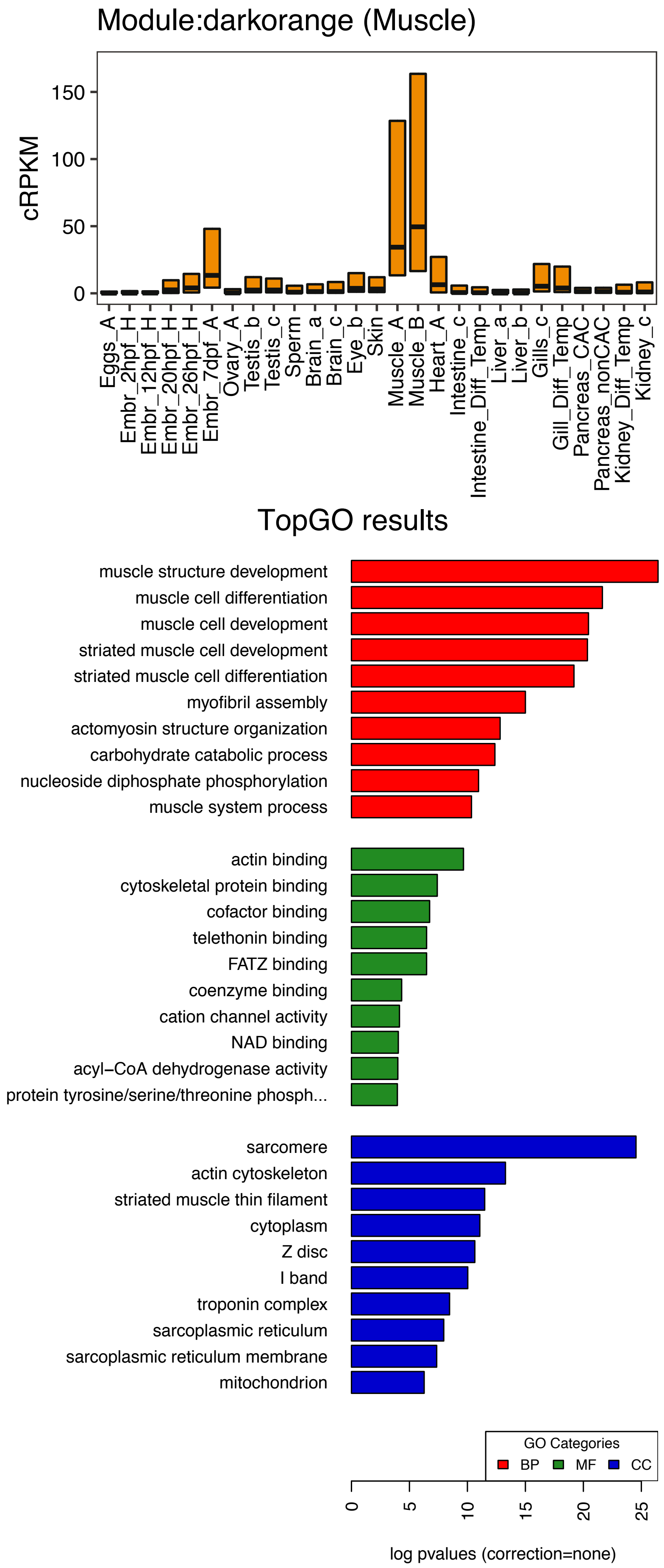

Module:darkorange (Muscle)
cRPKM
150
100
50
0
Eggs_A
Embr_2hpf_H
Embr_12hpf_H
Embr_20hpf_H
Embr_26hpf_H
Embr_7dpf_A
Ovary_A
Testis_b
Testis_c
Sperm
Brain_a
Brain_c
Eye_b
Skin
Muscle_A
Muscle_B
Heart_A
Intestine_c
Intestine_Diff_Temp
Liver_a
Liver_b
Gills_c
Gill_Diff_Temp
Pancreas_CAC
Pancreas_nonCAC
Kidney_Diff_Temp
Kidney_c
TopGO results
muscle structure development
muscle cell differentiation
muscle cell development
striated muscle cell development
striated muscle cell differentiation
myofibril assembly
actomyosin structure organization
carbohydrate catabolic process
nucleoside diphosphate phosphorylation
muscle system process
actin binding
cytoskeletal protein binding
cofactor binding
telethonin binding
FATZ binding
coenzyme binding
cation channel activity
NAD binding
acyl−CoA dehydrogenase activity
protein tyrosine/serine/threonine phosph...
sarcomere
actin cytoskeleton
striated muscle thin filament
cytoplasm
Z disc
I band
troponin complex
sarcoplasmic reticulum
sarcoplasmic reticulum membrane
mitochondrion
GO Categories
BP
MF
CC
0
5
10
15
20
25
log pvalues (correction=none)

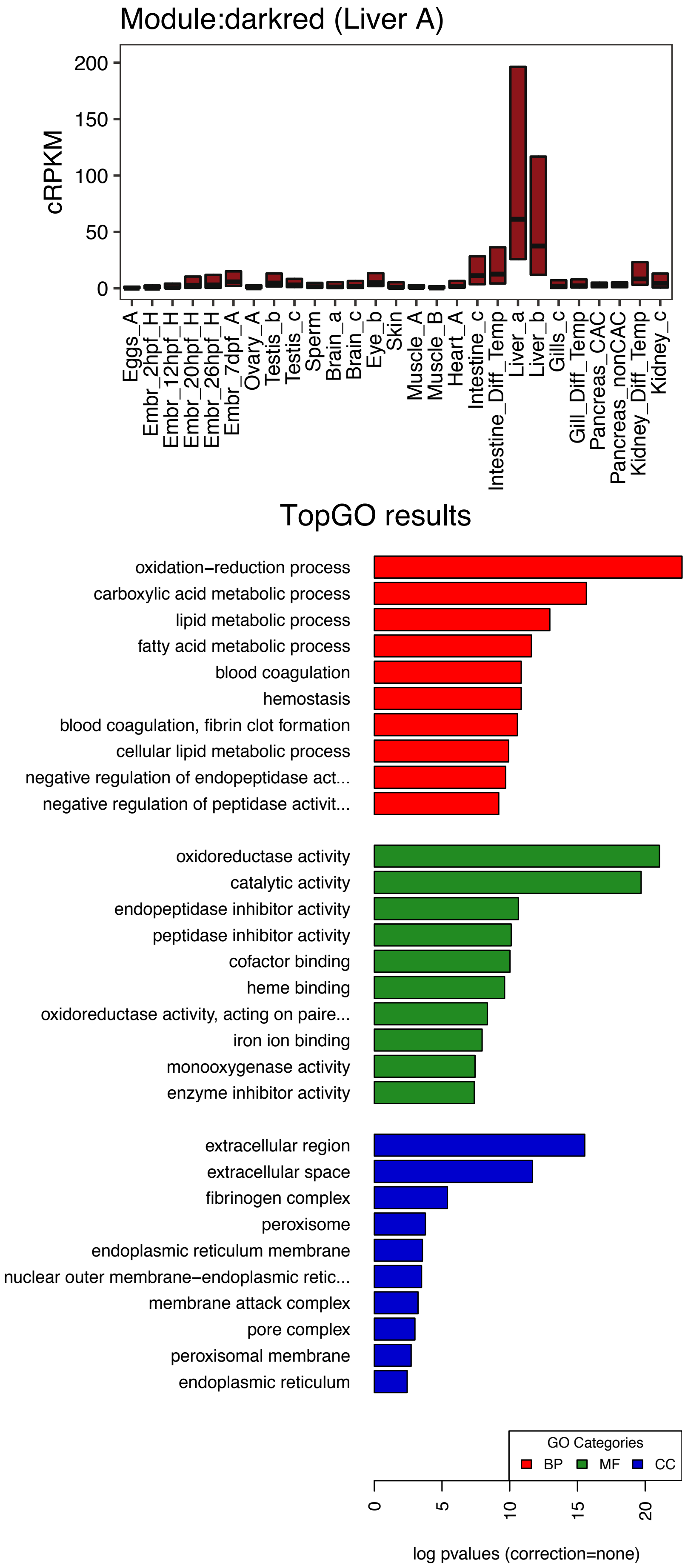

Module:darkred (Liver A)
cRPKM
0
50
100
150
200
Eggs_A
Embr_2hpf_H
Embr_12hpf_H
Embr_20hpf_H
Embr_26hpf_H
Embr_7dpf_A
Ovary_A
Testis_b
Testis_c
Sperm
Brain_a
Brain_c
Eye_b
Skin
Muscle_A
Muscle_B
Heart_A
Intestine_c
Intestine_Diff_Temp
Liver_a
Liver_b
Gills_c
Gill_Diff_Temp
Pancreas_CAC
Pancreas_nonCAC
Kidney_Diff_Temp
Kidney_c
TopGO results
oxidation−reduction process
carboxylic acid metabolic process
lipid metabolic process
fatty acid metabolic process
blood coagulation
hemostasis
blood coagulation, fibrin clot formation
cellular lipid metabolic process
negative regulation of endopeptidase act...
negative regulation of peptidase activit...
oxidoreductase activity
catalytic activity
endopeptidase inhibitor activity
peptidase inhibitor activity
cofactor binding
heme binding
oxidoreductase activity, acting on paire...
iron ion binding
monooxygenase activity
enzyme inhibitor activity
extracellular region
extracellular space
fibrinogen complex
peroxisome
endoplasmic reticulum membrane
nuclear outer membrane−endoplasmic retic...
membrane attack complex
pore complex
peroxisomal membrane
endoplasmic reticulum
GO Categories
BP
MF
CC
0
5
10
15
20
log pvalues (correction=none)

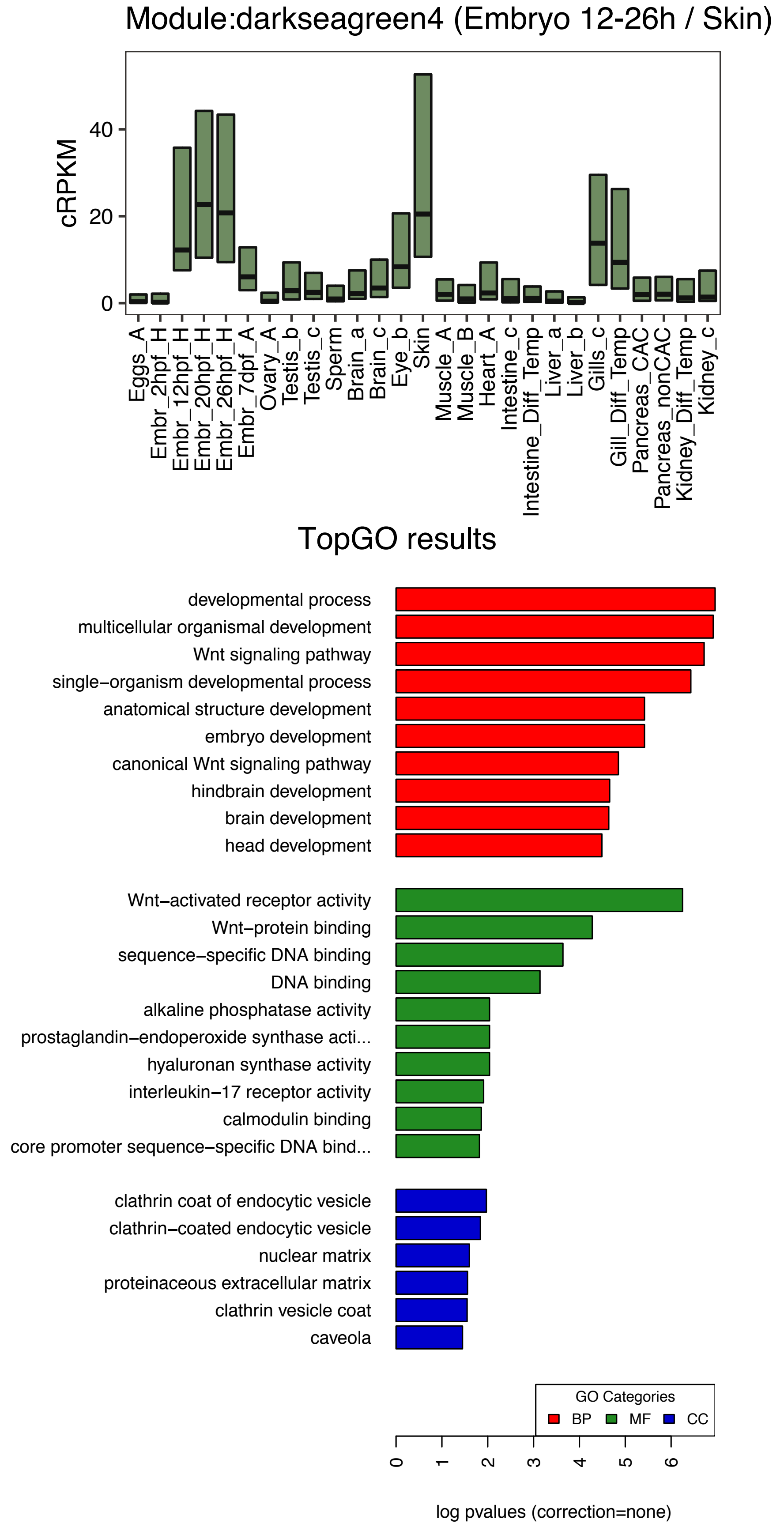
Module:darkseagreen4 (Embryo 12-26h / Skin)
cRPKM
0
20
40
Eggs_A
Embr_2hpf_H
Embr_12hpf_H
Embr_20hpf_H
Embr_26hpf_H
Embr_7dpf_A
Ovary_A
Testis_b
Testis_c
Sperm
Brain_a
Brain_c
Eye_b
Skin
Muscle_A
Muscle_B
Heart_A
Intestine_c
Intestine_Diff_Temp
Liver_a
Liver_b
Gills_c
Gill_Diff_Temp
Pancreas_CAC
Pancreas_nonCAC
Kidney_Diff_Temp
Kidney_c
TopGO results
developmental process
multicellular organismal development
Wnt signaling pathway
single−organism developmental process
anatomical structure development
embryo development
canonical Wnt signaling pathway
hindbrain development
brain development
head development
Wnt−activated receptor activity
Wnt−protein binding
sequence−specific DNA binding
DNA binding
alkaline phosphatase activity
prostaglandin−endoperoxide synthase acti...
hyaluronan synthase activity
interleukin−17 receptor activity
calmodulin binding
core promoter sequence−specific DNA bind...
clathrin coat of endocytic vesicle
clathrin−coated endocytic vesicle
nuclear matrix
proteinaceous extracellular matrix
clathrin vesicle coat
caveola
GO Categories
BP
MF
CC
0
1
2
3
4
5
6
log pvalues (correction=none)

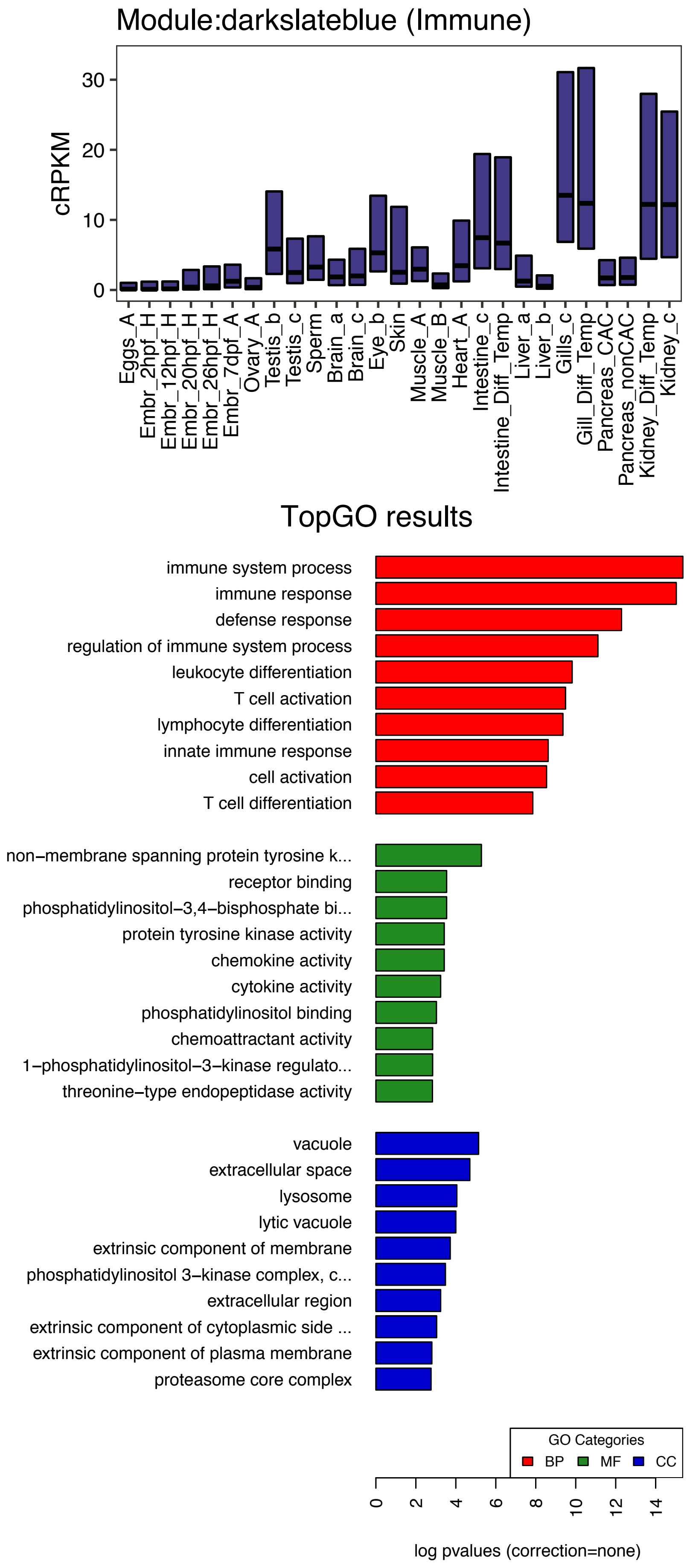

Module:darkslateblue (Immune)
cRPKM
0
10
20
30
Eggs_A
Embr_2hpf_H
Embr_12hpf_H
Embr_20hpf_H
Embr_26hpf_H
Embr_7dpf_A
Ovary_A
Testis_b
Testis_c
Sperm
Brain_a
Brain_c
Eye_b
Skin
Muscle_A
Muscle_B
Heart_A
Intestine_c
Intestine_Diff_Temp
Liver_a
Liver_b
Gills_c
Gill_Diff_Temp
Pancreas_CAC
Pancreas_nonCAC
Kidney_Diff_Temp
Kidney_c
TopGO results
immune system process
immune response
defense response
regulation of immune system process
leukocyte differentiation
T cell activation
lymphocyte differentiation
innate immune response
cell activation
T cell differentiation
non−membrane spanning protein tyrosine k...
receptor binding
phosphatidylinositol−3,4−bisphosphate bi...
protein tyrosine kinase activity
chemokine activity
cytokine activity
phosphatidylinositol binding
chemoattractant activity
1−phosphatidylinositol−3−kinase regulato...
threonine−type endopeptidase activity
vacuole
extracellular space
lysosome
lytic vacuole
extrinsic component of membrane
phosphatidylinositol 3−kinase complex, c...
extracellular region
extrinsic component of cytoplasmic side ...
extrinsic component of plasma membrane
proteasome core complex
GO Categories
BP
MF
CC
0
2
4
6
8
10
12
14
log pvalues (correction=none)

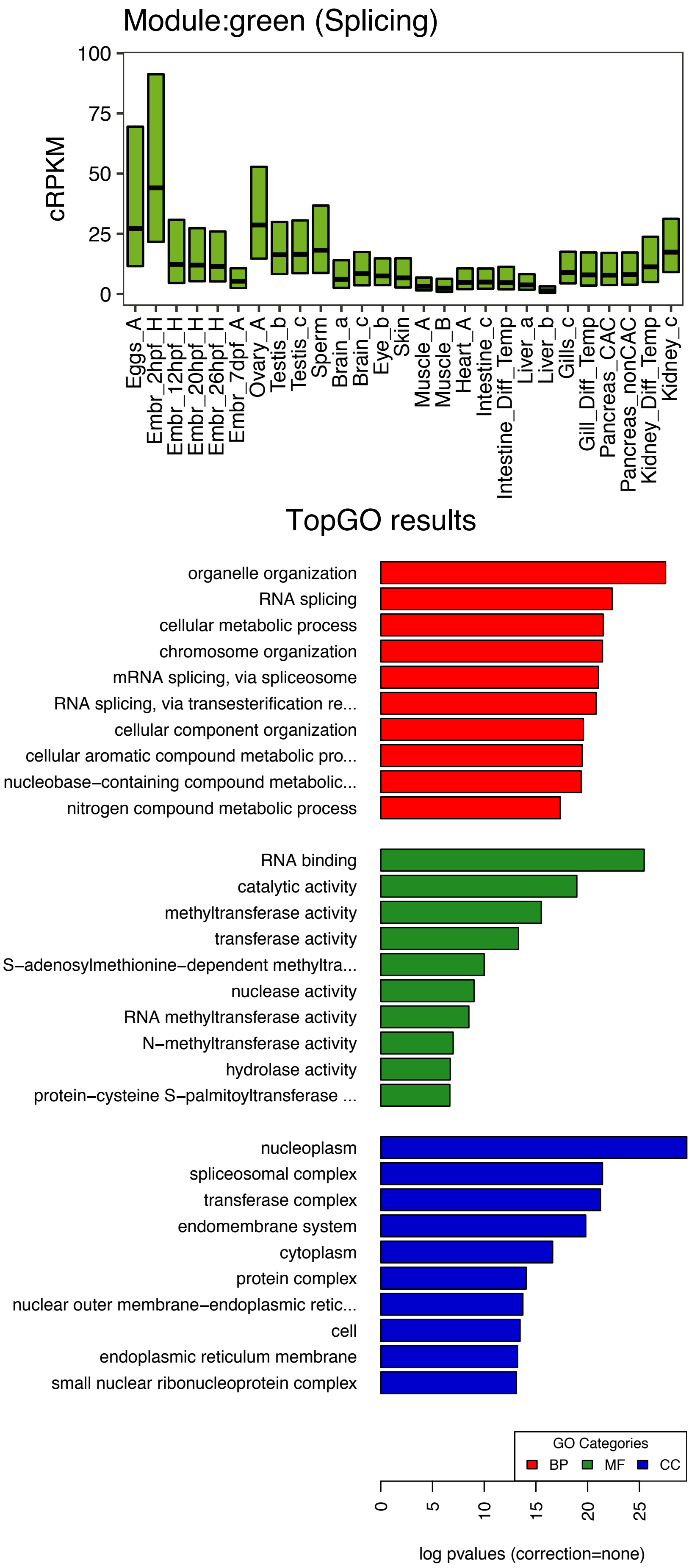

Module:green (Splicing)
cRPKM
100
75
50
25
0
Eggs_A
Embr_2hpf_H
Embr_12hpf_H
Embr_20hpf_H
Embr_26hpf_H
Embr_7dpf_A
Ovary_A
Testis_b
Testis_c
Sperm
Brain_a
Brain_c
Eye_b
Skin
Muscle_A
Muscle_B
Heart_A
Intestine_c
Intestine_Diff_Temp
Liver_a
Liver_b
Gills_c
Gill_Diff_Temp
Pancreas_CAC
Pancreas_nonCAC
Kidney_Diff_Temp
Kidney_c
TopGO results
organelle organization
RNA splicing
cellular metabolic process
chromosome organization
mRNA splicing, via spliceosome
RNA splicing, via transesterification re...
cellular component organization
cellular aromatic compound metabolic pro...
nucleobase−containing compound metabolic...
nitrogen compound metabolic process
RNA binding
catalytic activity
methyltransferase activity
transferase activity
S−adenosylmethionine−dependent methyltra...
nuclease activity
RNA methyltransferase activity
N−methyltransferase activity
hydrolase activity
protein−cysteine S−palmitoyltransferase ...
nucleoplasm
spliceosomal complex
transferase complex
endomembrane system
cytoplasm
protein complex
nuclear outer membrane−endoplasmic retic...
cell
endoplasmic reticulum membrane
small nuclear ribonucleoprotein complex
GO Categories
BP
MF
CC
0
5
10
15
20
25
log pvalues (correction=none)

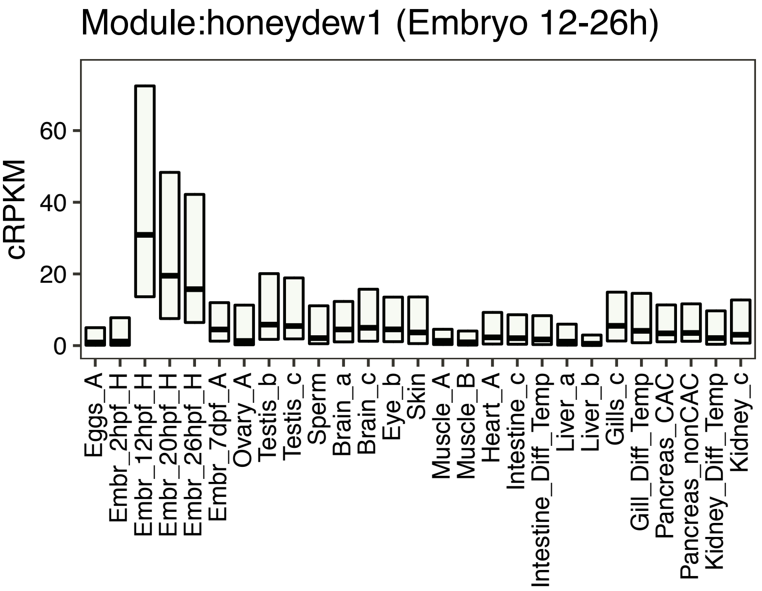
Module:honeydew1 (Embryo 12-26h)
cRPKM
0
20
40
60
Eggs_A
Embr_2hpf_H
Embr_12hpf_H
Embr_20hpf_H
Embr_26hpf_H
Embr_7dpf_A
Ovary_A
Testis_b
Testis_c
Sperm
Brain_a
Brain_c
Eye_b
Skin
Muscle_A
Muscle_B
Heart_A
Intestine_c
Intestine_Diff_Temp
Liver_a
Liver_b
Gills_c
Gill_Diff_Temp
Pancreas_CAC
Pancreas_nonCAC
Kidney_Diff_Temp
Kidney_c

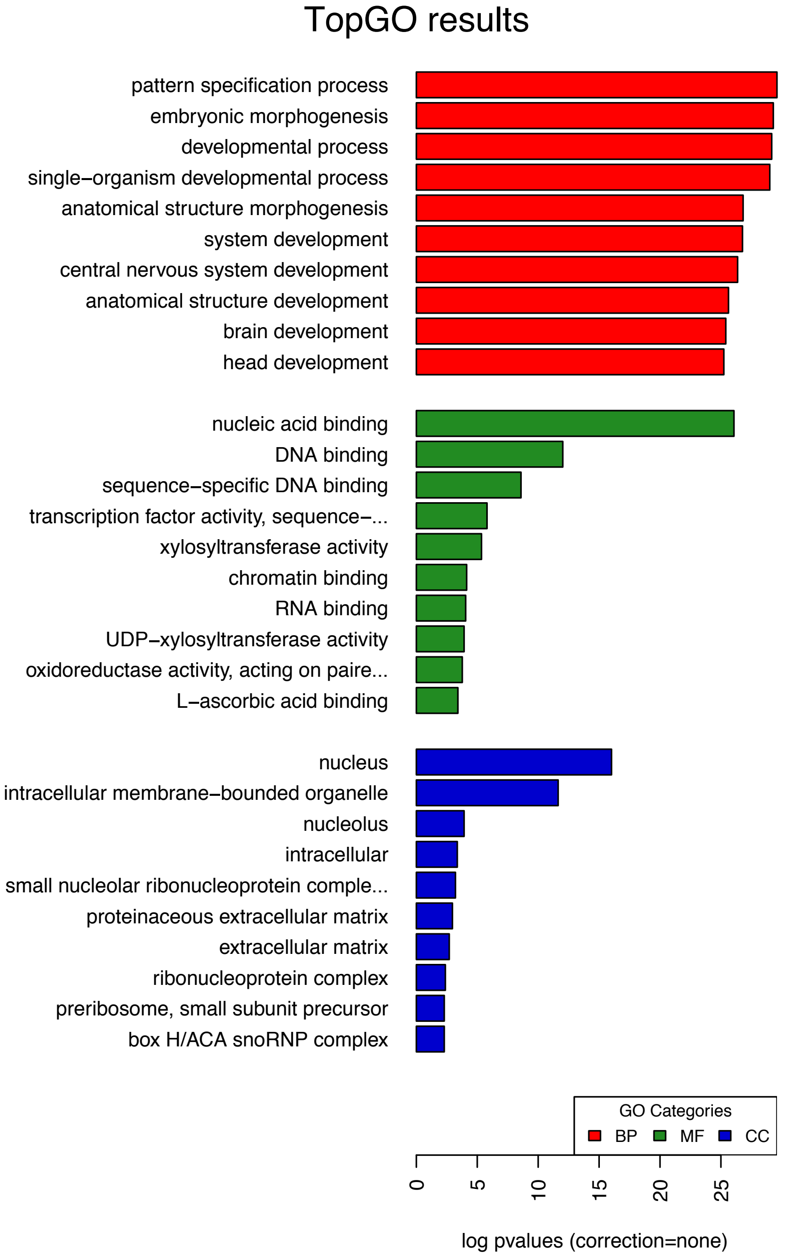
TopGO results
pattern specification process
embryonic morphogenesis
developmental process
single−organism developmental process
anatomical structure morphogenesis
system development
central nervous system development
anatomical structure development
brain development
head development
nucleic acid binding
DNA binding
sequence−specific DNA binding
transcription factor activity, sequence−...
xylosyltransferase activity
chromatin binding
RNA binding
UDP−xylosyltransferase activity
oxidoreductase activity, acting on paire...
L−ascorbic acid binding
nucleus
intracellular membrane−bounded organelle
nucleolus
intracellular
small nucleolar ribonucleoprotein comple...
proteinaceous extracellular matrix
extracellular matrix
ribonucleoprotein complex
preribosome, small subunit precursor
box H/ACA snoRNP complex
GO Categories
BP
MF
CC
0
5
10
15
20
25
log pvalues (correction=none)

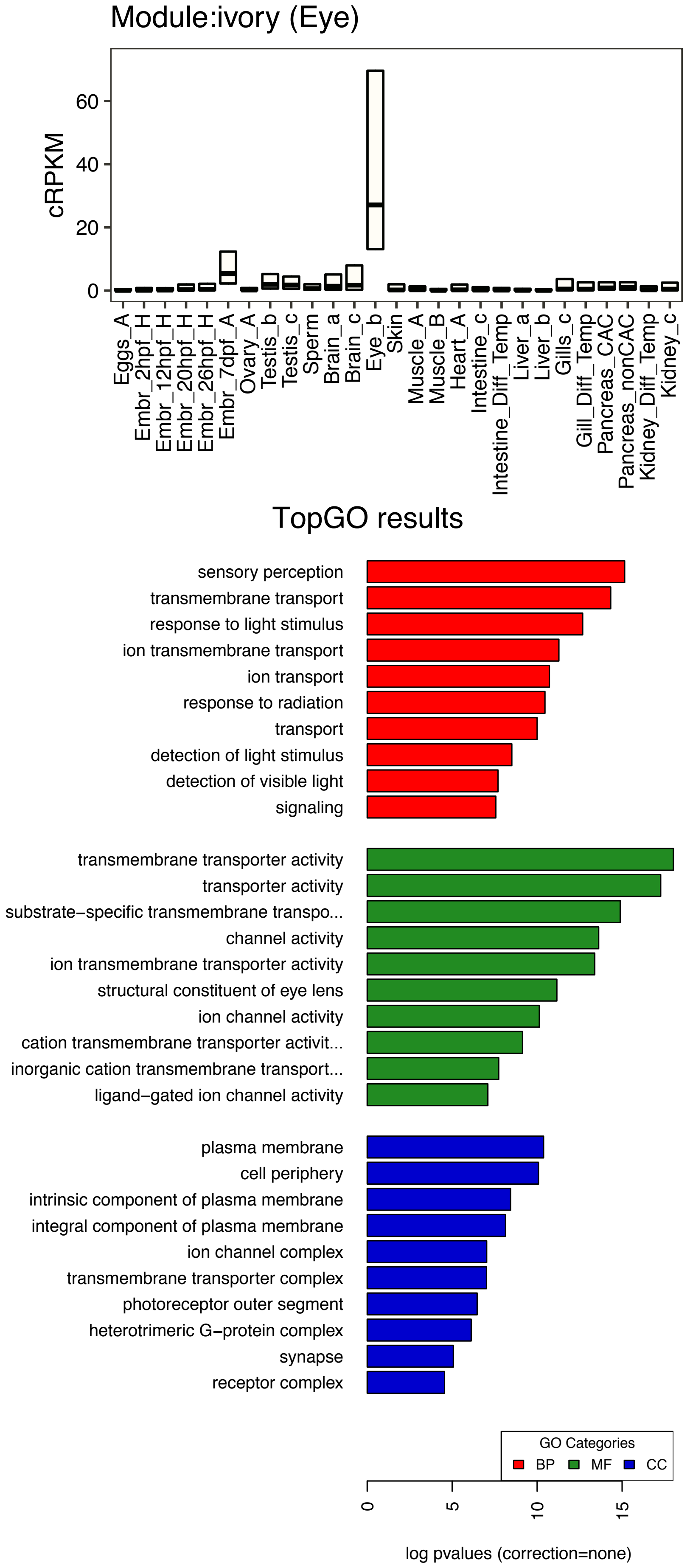
Module:ivory (Eye)
cRPKM
0
20
40
60
Eggs_A
Embr_2hpf_H
Embr_12hpf_H
Embr_20hpf_H
Embr_26hpf_H
Embr_7dpf_A
Ovary_A
Testis_b
Testis_c
Sperm
Brain_a
Brain_c
Eye_b
Skin
Muscle_A
Muscle_B
Heart_A
Intestine_c
Intestine_Diff_Temp
Liver_a
Liver_b
Gills_c
Gill_Diff_Temp
Pancreas_CAC
Pancreas_nonCAC
Kidney_Diff_Temp
Kidney_c
TopGO results
sensory perception
transmembrane transport
response to light stimulus
ion transmembrane transport
ion transport
response to radiation
transport
detection of light stimulus
detection of visible light
signaling
transmembrane transporter activity
transporter activity
substrate−specific transmembrane transpo...
channel activity
ion transmembrane transporter activity
structural constituent of eye lens
ion channel activity
cation transmembrane transporter activit...
inorganic cation transmembrane transport...
ligand−gated ion channel activity
plasma membrane
cell periphery
intrinsic component of plasma membrane
integral component of plasma membrane
ion channel complex
transmembrane transporter complex
photoreceptor outer segment
heterotrimeric G−protein complex
synapse
receptor complex
GO Categories
BP
MF
CC
0
5
10
15
log pvalues (correction=none)

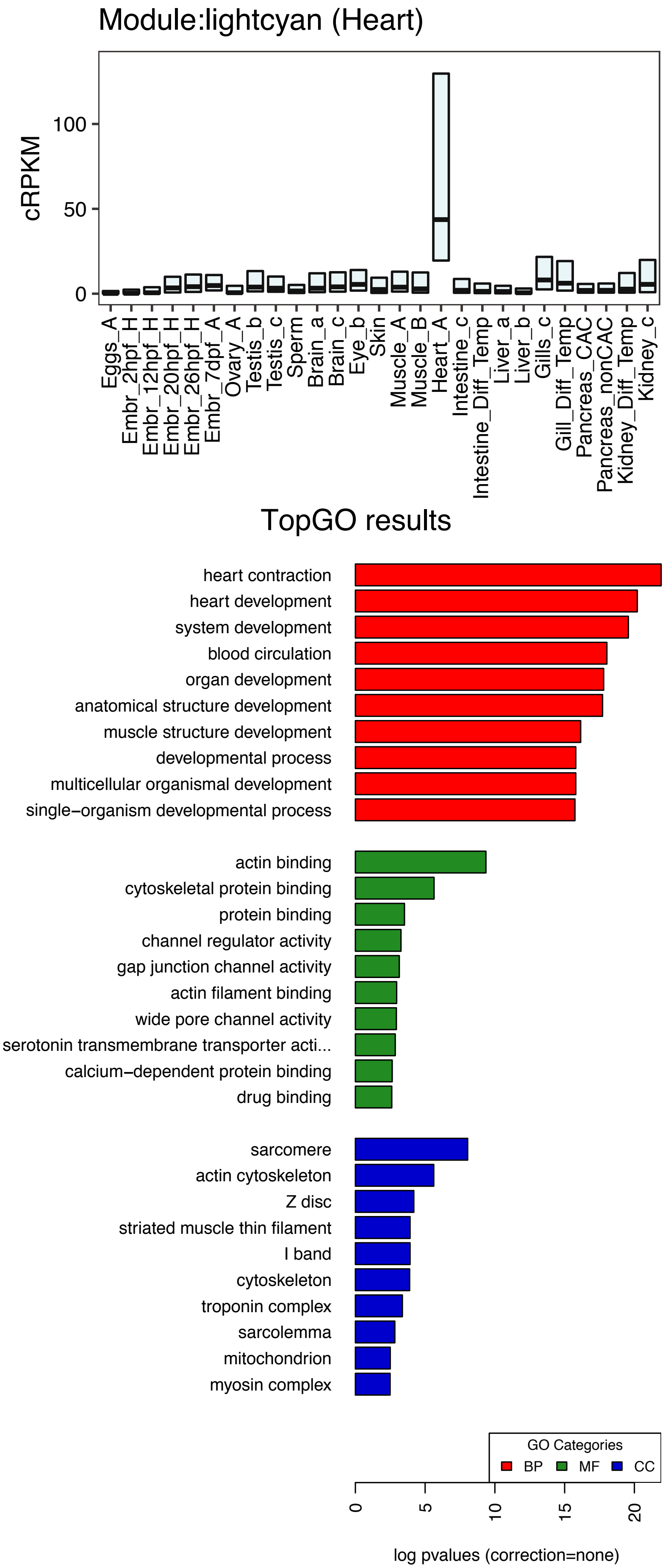
Module:lightcyan (Heart)
cRPKM
100
50
0
Eggs_A
Embr_2hpf_H
Embr_12hpf_H
Embr_20hpf_H
Embr_26hpf_H
Embr_7dpf_A
Ovary_A
Testis_b
Testis_c
Sperm
Brain_a
Brain_c
Eye_b
Skin
Muscle_A
Muscle_B
Heart_A
Intestine_c
Intestine_Diff_Temp
Liver_a
Liver_b
Gills_c
Gill_Diff_Temp
Pancreas_CAC
Pancreas_nonCAC
Kidney_Diff_Temp
Kidney_c
TopGO results
heart contraction
heart development
system development
blood circulation
organ development
anatomical structure development
muscle structure development
developmental process
multicellular organismal development
single−organism developmental process
actin binding
cytoskeletal protein binding
protein binding
channel regulator activity
gap junction channel activity
actin filament binding
wide pore channel activity
serotonin transmembrane transporter acti...
calcium−dependent protein binding
drug binding
sarcomere
actin cytoskeleton
Z disc
striated muscle thin filament
I band
cytoskeleton
troponin complex
sarcolemma
mitochondrion
myosin complex
GO Categories
BP
MF
CC
0
5
10
15
20
log pvalues (correction=none)

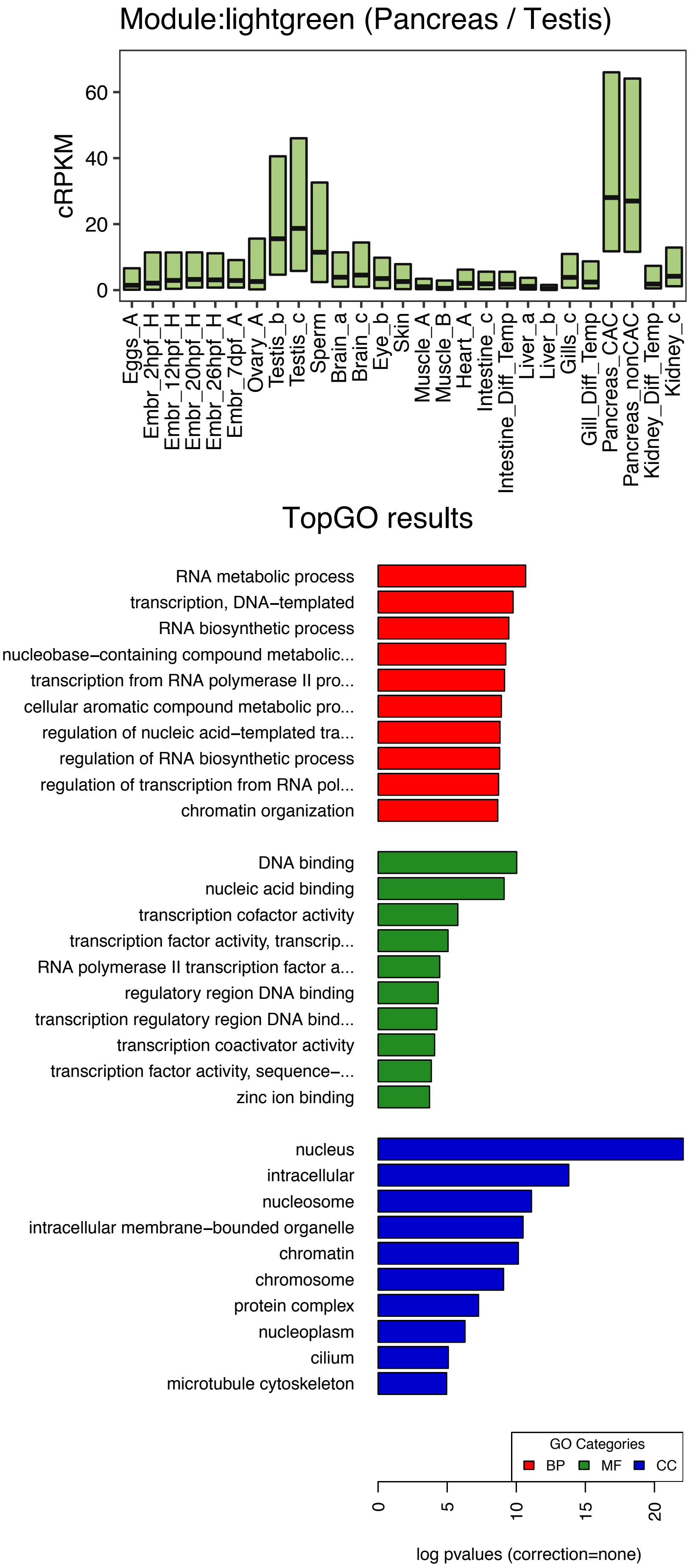
Module:lightgreen (Pancreas / Testis)
cRPKM
0
20
40
60
Eggs_A
Embr_2hpf_H
Embr_12hpf_H
Embr_20hpf_H
Embr_26hpf_H
Embr_7dpf_A
Ovary_A
Testis_b
Testis_c
Sperm
Brain_a
Brain_c
Eye_b
Skin
Muscle_A
Muscle_B
Heart_A
Intestine_c
Intestine_Diff_Temp
Liver_a
Liver_b
Gills_c
Gill_Diff_Temp
Pancreas_CAC
Pancreas_nonCAC
Kidney_Diff_Temp
Kidney_c
TopGO results
RNA metabolic process
transcription, DNA−templated
RNA biosynthetic process
nucleobase−containing compound metabolic...
transcription from RNA polymerase II pro...
cellular aromatic compound metabolic pro...
regulation of nucleic acid−templated tra...
regulation of RNA biosynthetic process
regulation of transcription from RNA pol...
chromatin organization
DNA binding
nucleic acid binding
transcription cofactor activity
transcription factor activity, transcrip...
RNA polymerase II transcription factor a...
regulatory region DNA binding
transcription regulatory region DNA bind...
transcription coactivator activity
transcription factor activity, sequence−...
zinc ion binding
nucleus
intracellular
nucleosome
intracellular membrane−bounded organelle
chromatin
chromosome
protein complex
nucleoplasm
cilium
microtubule cytoskeleton
GO Categories
BP
MF
CC
0
5
10
15
20
log pvalues (correction=none)

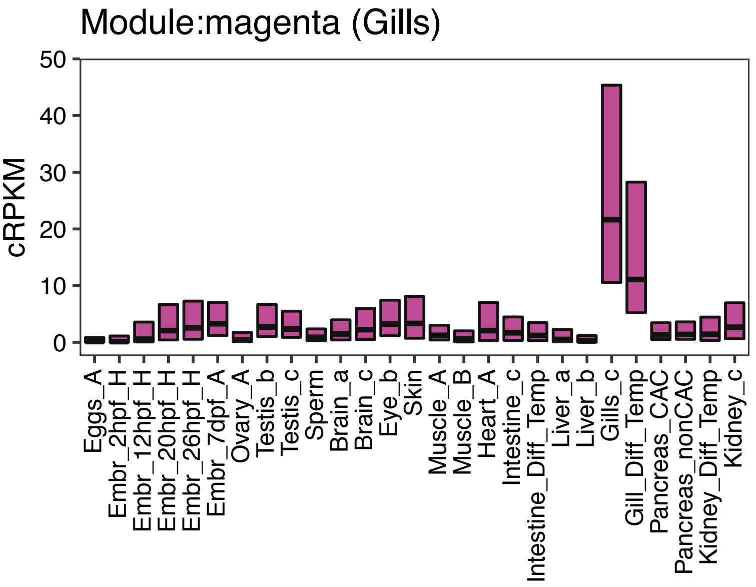
Module:magenta (Gills)
cRPKM
50
40
30
20
10
0
Eggs_A
Embr_2hpf_H
Embr_12hpf_H
Embr_20hpf_H
Embr_26hpf_H
Embr_7dpf_A
Ovary_A
Testis_b
Testis_c
Sperm
Brain_a
Brain_c
Eye_b
Skin
Muscle_A
Muscle_B
Heart_A
Intestine_c
Intestine_Diff_Temp
Liver_a
Liver_b
Gills_c
Gill_Diff_Temp
Pancreas_CAC
Pancreas_nonCAC
Kidney_Diff_Temp
Kidney_c

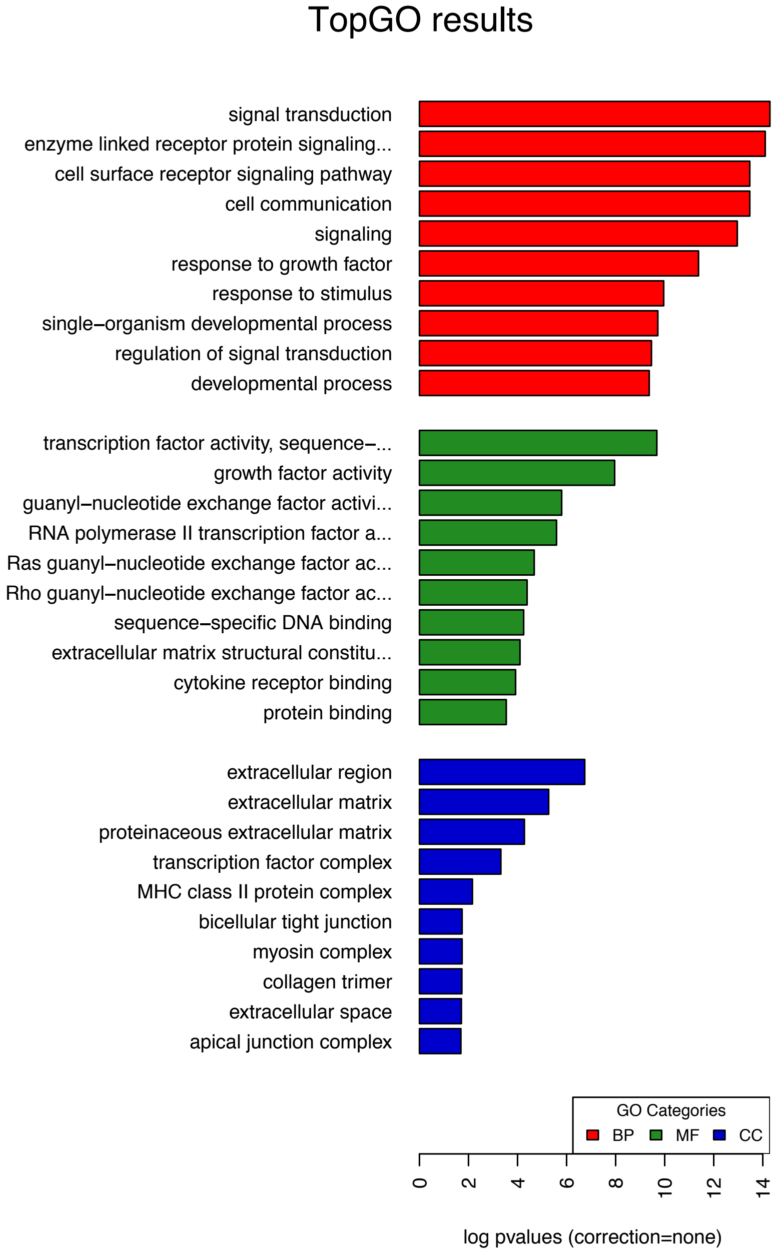
TopGO results
signal transduction
enzyme linked receptor protein signaling...
cell surface receptor signaling pathway
cell communication
signaling
response to growth factor
response to stimulus
single−organism developmental process
regulation of signal transduction
developmental process
transcription factor activity, sequence−...
growth factor activity
guanyl−nucleotide exchange factor activi...
RNA polymerase II transcription factor a...
Ras guanyl−nucleotide exchange factor ac...
Rho guanyl−nucleotide exchange factor ac...
sequence−specific DNA binding
extracellular matrix structural constitu...
cytokine receptor binding
protein binding
extracellular region
extracellular matrix
proteinaceous extracellular matrix
transcription factor complex
MHC class II protein complex
bicellular tight junction
myosin complex
collagen trimer
extracellular space
apical junction complex
GO Categories
BP
MF
CC
0
2
4
6
8
10
12
14
log pvalues (correction=none)

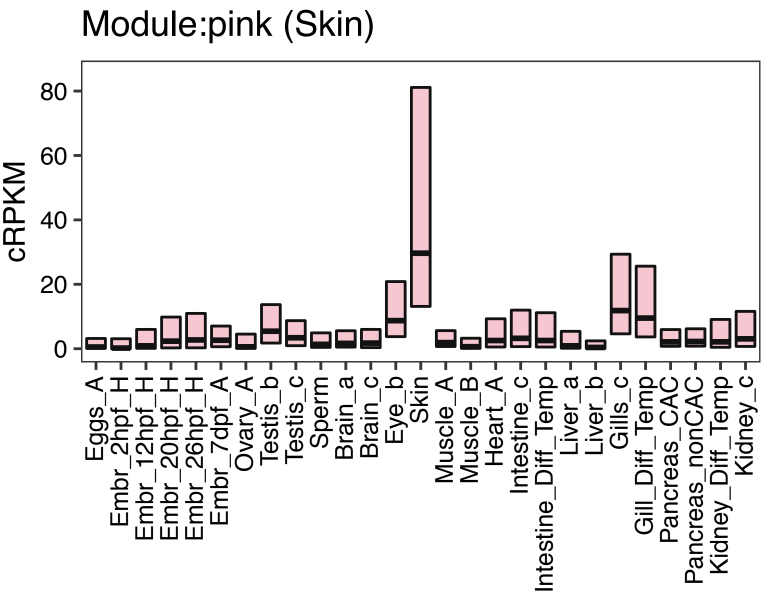
Module:pink (Skin)
cRPKM
80
60
40
20
0
Eggs_A
Embr_2hpf_H
Embr_12hpf_H
Embr_20hpf_H
Embr_26hpf_H
Embr_7dpf_A
Ovary_A
Testis_b
Testis_c
Sperm
Brain_a
Brain_c
Eye_b
Skin
Muscle_A
Muscle_B
Heart_A
Intestine_c
Intestine_Diff_Temp
Liver_a
Liver_b
Gills_c
Gill_Diff_Temp
Pancreas_CAC
Pancreas_nonCAC
Kidney_Diff_Temp
Kidney_c

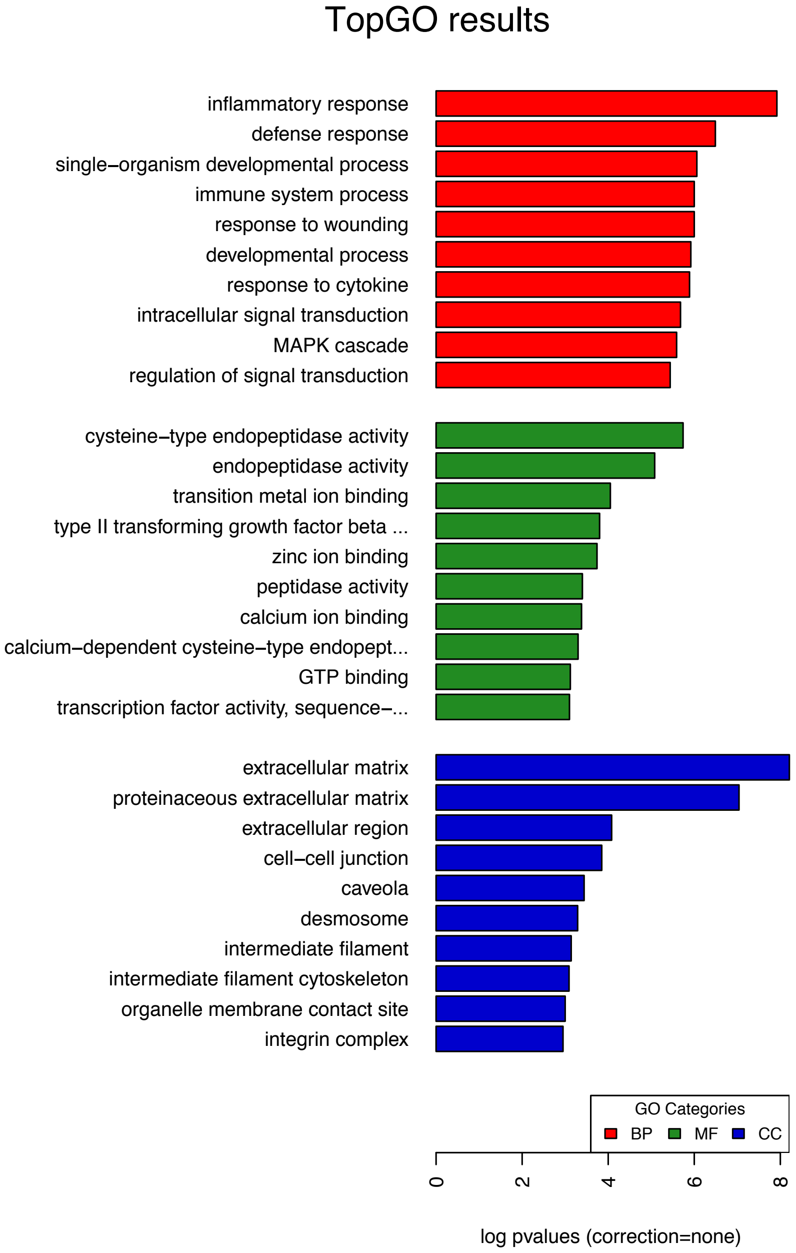
TopGO results
inflammatory response
defense response
single−organism developmental process
immune system process
response to wounding
developmental process
response to cytokine
intracellular signal transduction
MAPK cascade
regulation of signal transduction
cysteine−type endopeptidase activity
endopeptidase activity
transition metal ion binding
type II transforming growth factor beta ...
zinc ion binding
peptidase activity
calcium ion binding
calcium−dependent cysteine−type endopept...
GTP binding
transcription factor activity, sequence−...
extracellular matrix
proteinaceous extracellular matrix
extracellular region
cell−cell junction
caveola
desmosome
intermediate filament
intermediate filament cytoskeleton
organelle membrane contact site
integrin complex
GO Categories
BP
MF
CC
0
2
4
6
8
log pvalues (correction=none)

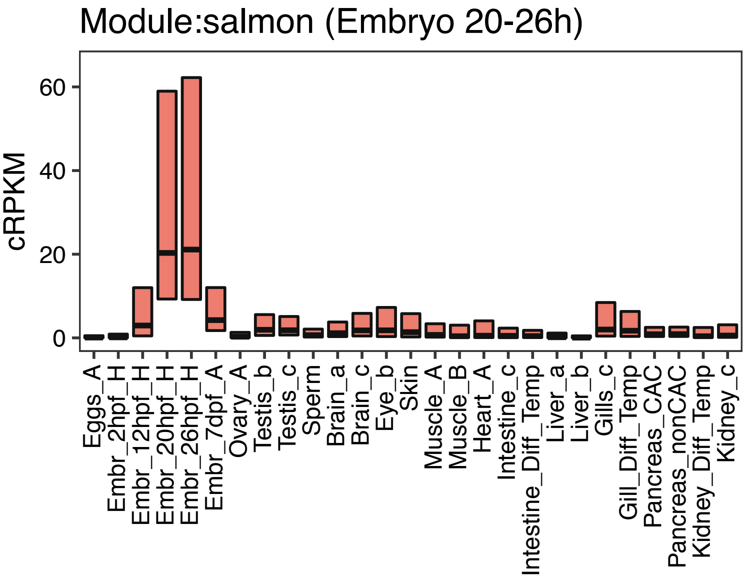
Module:salmon (Embryo 20-26h)
cRPKM
0
20
40
60
Eggs_A
Embr_2hpf_H
Embr_12hpf_H
Embr_20hpf_H
Embr_26hpf_H
Embr_7dpf_A
Ovary_A
Testis_b
Testis_c
Sperm
Brain_a
Brain_c
Eye_b
Skin
Muscle_A
Muscle_B
Heart_A
Intestine_c
Intestine_Diff_Temp
Liver_a
Liver_b
Gills_c
Gill_Diff_Temp
Pancreas_CAC
Pancreas_nonCAC
Kidney_Diff_Temp
Kidney_c

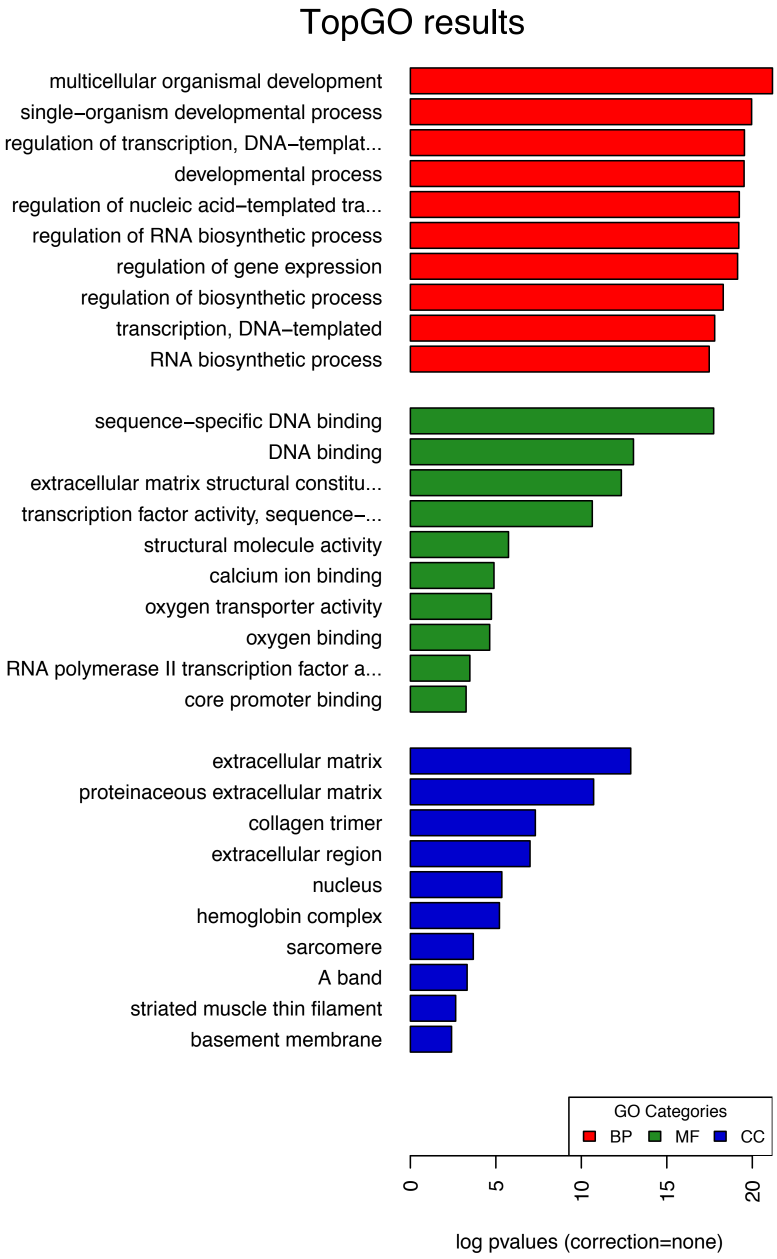
TopGO results
multicellular organismal development
single−organism developmental process
regulation of transcription, DNA−templat...
developmental process
regulation of nucleic acid−templated tra...
regulation of RNA biosynthetic process
regulation of gene expression
regulation of biosynthetic process
transcription, DNA−templated
RNA biosynthetic process
sequence−specific DNA binding
DNA binding
extracellular matrix structural constitu...
transcription factor activity, sequence−...
structural molecule activity
calcium ion binding
oxygen transporter activity
oxygen binding
RNA polymerase II transcription factor a...
core promoter binding
extracellular matrix
proteinaceous extracellular matrix
collagen trimer
extracellular region
nucleus
hemoglobin complex
sarcomere
A band
striated muscle thin filament
basement membrane
GO Categories
BP
MF
CC
0
5
10
15
20
log pvalues (correction=none)

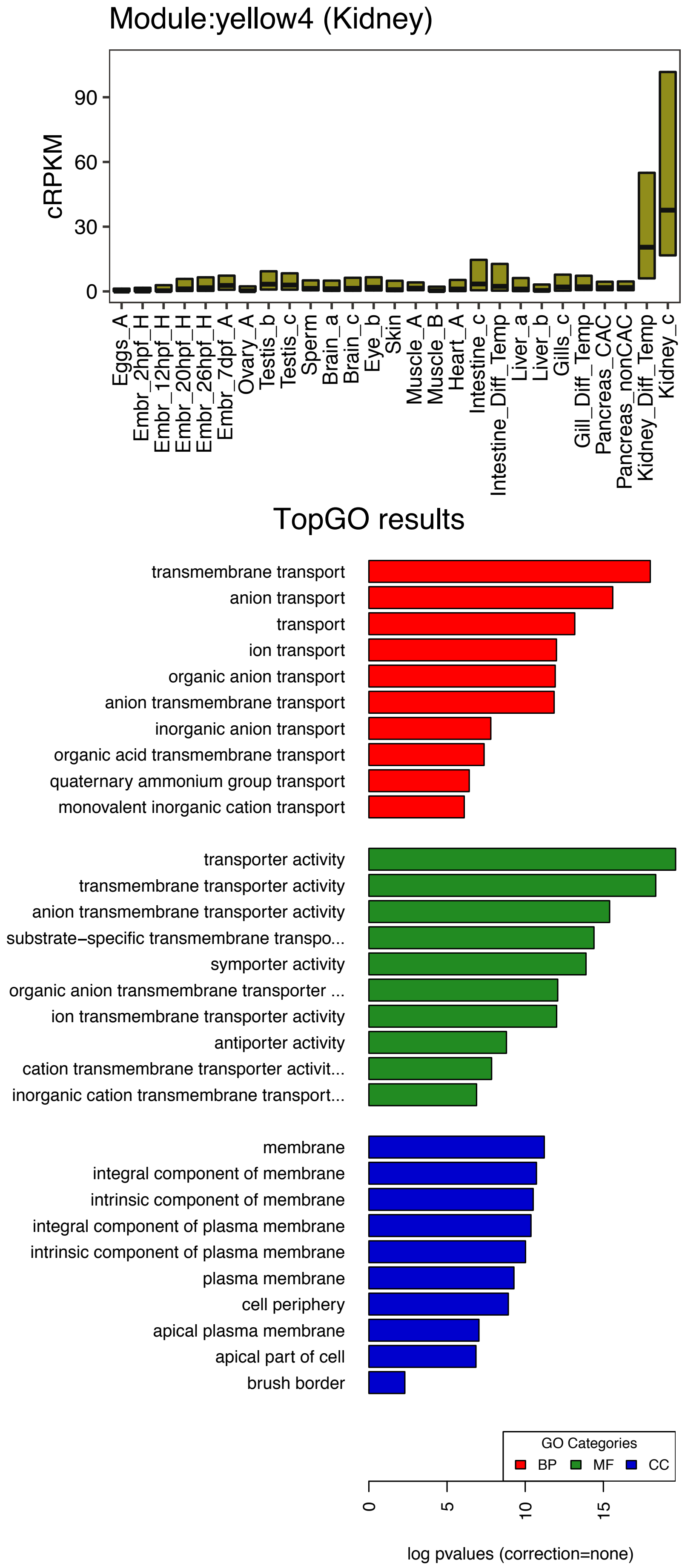

Module:yellow4 (Kidney)
cRPKM
90
60
30
0
Eggs_A
Embr_2hpf_H
Embr_12hpf_H
Embr_20hpf_H
Embr_26hpf_H
Embr_7dpf_A
Ovary_A
Testis_b
Testis_c
Sperm
Brain_a
Brain_c
Eye_b
Skin
Muscle_A
Muscle_B
Heart_A
Intestine_c
Intestine_Diff_Temp
Liver_a
Liver_b
Gills_c
Gill_Diff_Temp
Pancreas_CAC
Pancreas_nonCAC
Kidney_Diff_Temp
Kidney_c
TopGO results
transmembrane transport
anion transport
transport
ion transport
organic anion transport
anion transmembrane transport
inorganic anion transport
organic acid transmembrane transport
quaternary ammonium group transport
monovalent inorganic cation transport
transporter activity
transmembrane transporter activity
anion transmembrane transporter activity
substrate−specific transmembrane transpo...
symporter activity
organic anion transmembrane transporter ...
ion transmembrane transporter activity
antiporter activity
cation transmembrane transporter activit...
inorganic cation transmembrane transport...
membrane
integral component of membrane
intrinsic component of membrane
integral component of plasma membrane
intrinsic component of plasma membrane
plasma membrane
cell periphery
apical plasma membrane
apical part of cell
brush border
GO Categories
BP
MF
CC
0
5
10
15
log pvalues (correction=none)

Module:yellowgreen (Gills early)

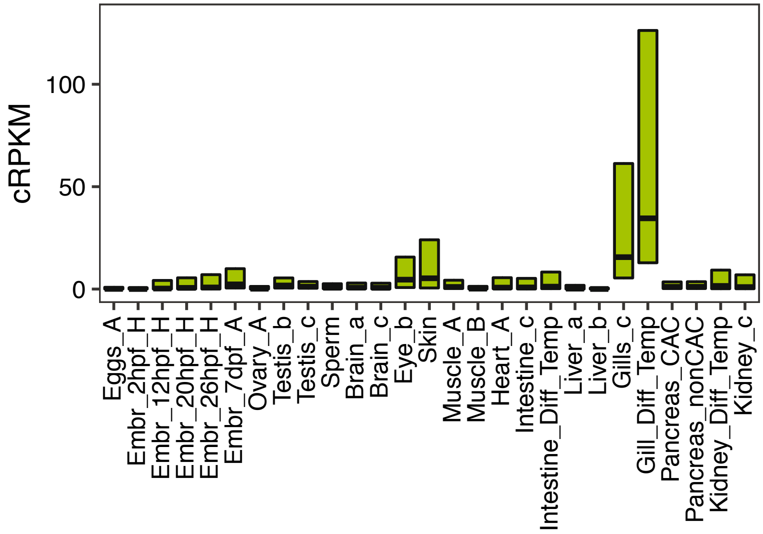

TopGO results

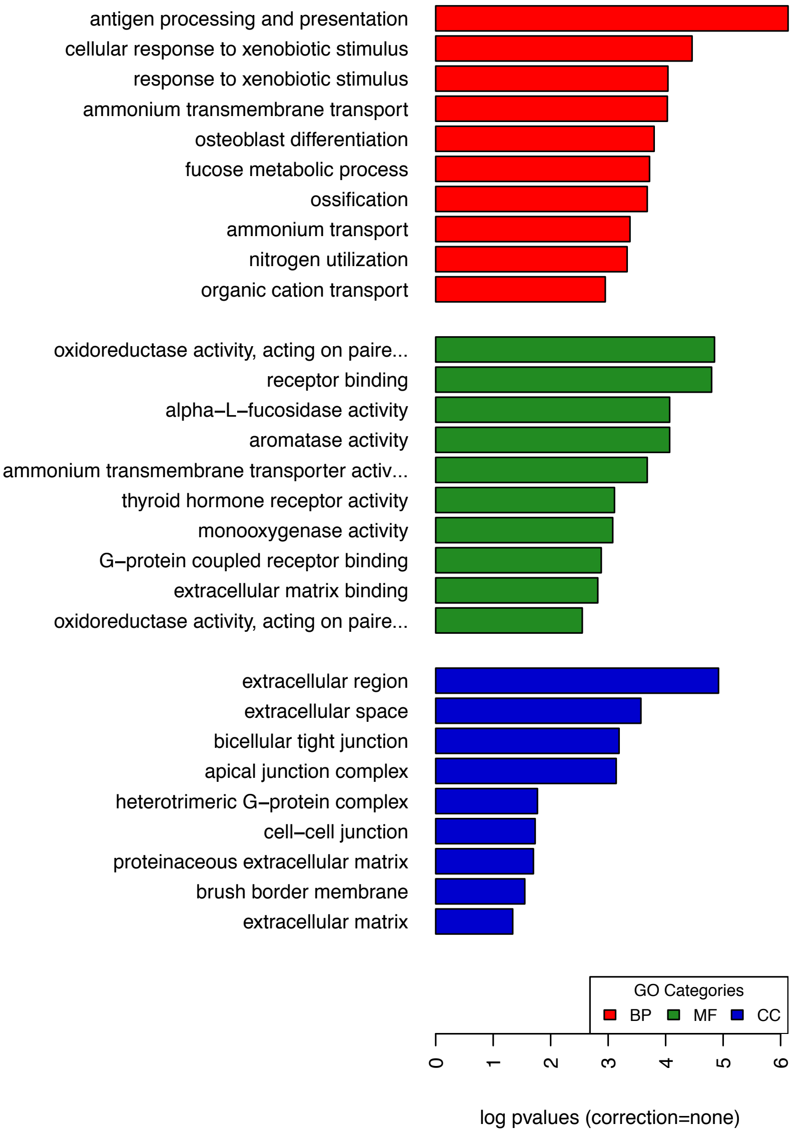

Supplement: Supplementary file 3 — This file contains the detailed annotation and expression patterns for each module of co-regulated genes identified by WGCNA in amphioxus and zebrafish. Section descriptions: 1 - Amphioxus and zebrafish module annotation and comparisons. a, Name assigned to each module (color) based on gene expression and/or GO enrichment. b, Same heatmaps as in Fig. 4a,b including the names of each module. 2 - Clustered heatmap of TF-motifs vs amphioxus & zebrafish modules. a, WGCNA modules from the two species are plotted against all motif clusters. The values visualized are the z-scores of each motif in each module. Modules and motifs are clustered based on the correlation of the visualized z-scores. b, Key for TF motif super-families in a, ordered by size of the motif name. 3 - Amphioxus module RNA-seq expression and GO terms. For each amphioxus module, boxplots without whiskers showing the median and interquartile range of gene expression levels (using the cRPKM metrics) across RNA-seq samples (top), and significantly enriched GO categories (bottom). P-values correspond to uncorrected p-values from two-sided Fisher's exact tests as calculated by topGO. Number of genes per module is provided in SI Dataset 8. 4 - Zebrafish module RNA-seq expression and GO terms. For each zebrafish module, boxplots without whiskers showing the median and interquartile range of gene expression levels (using the cRPKM metrics) across RNA-seq samples (top), and significantly enriched GO categories (bottom). P-values correspond to uncorrected p-values from two-sided Fisher's exact tests as calculated by topGO. Number of genes per module is provided in SI Dataset 8. [file 41586_2018_734_MOESM3_ESM.pdf]
